# (2*R*,1'*S*,2'*R*)- and (2*S*,1'*S*,2'*R*)-3-[2-Mono(di,tri)fluoromethylcyclopropyl]alanines and their incorporation into hormaomycin analogues

**DOI:** 10.3762/bjoc.10.302

**Published:** 2014-12-03

**Authors:** Armin de Meijere, Sergei I Kozhushkov, Dmitrii S Yufit, Christian Grosse, Marcel Kaiser, Vitaly A Raev

**Affiliations:** 1 Institut für Organische und Biomolekulare Chemie der Georg-August-Universität Göttingen, Tammannstrasse 2, 37077 Göttingen, Germany; 2 Department of Chemistry, University of Durham, South Rd., Durham DH1 3L, UK; 3 Institut für Anorganische Chemie der Georg-August-Universität Göttingen, Tammannstrasse 4, 37077 Göttingen, Germany; 4 Swiss Tropical and Public Health Institute, Parasite Chemotherapy, Socinstrasse 57, CH-4002 Basel, Switzerland; 5 University of Basel, Petersplatz, 1 CH-4003 Basel Switzerland; 6 Institut für Organische Chemie der TU Carolo-Wilhelmina zu Braunschweig, Hagenring 30, 38106 Braunschweig, Germany

**Keywords:** amino acids, biosynthesis, building blocks, cyclopropanes, natural products, synthetic methods

## Abstract

Efficient and scalable syntheses of enantiomerically pure (2*R*,1'*S*,2'*R*)- and (2*S*,1'*S*,2'*R*)-3-[2-mono(di,tri)fluoromethylcyclopropyl]alanines **9a**–**c**, as well as *allo*-D-threonine (**4**) and (2*S*,3*R*)-β-methylphenylalanine (**3**), using the Belokon' approach with (*S*)- and (*R*)-2-[(*N*-benzylprolyl)amino]benzophenone [(*S*)- and (*R*)-**10**] as reusable chiral auxiliaries have been developed. Three new fluoromethyl analogues of the naturally occurring octadepsipeptide hormaomycin (**1**) with (fluoromethylcyclopropyl)alanine moieties have been synthesized and subjected to preliminary tests of their antibiotic activity.

## Introduction

**Figure 1:**
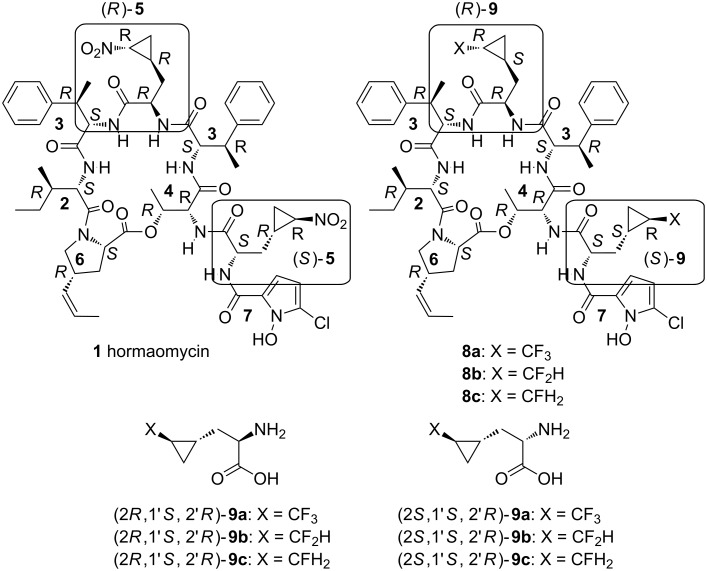
Structure and absolute configuration of hormaomycin (**1**), its fluoromethyl-substituted analogues **8a**–**c** and 3-(2-fluoromethylcyclopropyl]alanine building blocks **9a**–**c**. **2**: (*S*)-Ile; **3**: (2*S*,3*R*)-(β-Me)Phe; **4**: (2*R*)-α-Thr; **5**: (1'*R*,2'*R*)-(3-Ncp)Ala; **6**: (2*S*,4*R*)-4-(*Z*)-(4-PE)Pro; **7**: Chpca; **9**: (1'*S*,2'*R*)-(3-FMcp)Ala.

The intermolecular signal metabolite hormaomycin (**1**, Figure 1) was first isolated from a *Streptomyces griseoflavus* (strain W-384) fermentation broth by Zähner et al. in Tübingen, Germany and structurally identified by Zeeck et al. in Göttingen, Germany in 1989–1990 [3–4]. Once the absolute configuration of all the previously unassigned stereogenic centers in the octapeptidolactone had been established [5–6] and feasible enantioselective syntheses of all the amino acid building blocks had been developed [7–8], a total synthesis of **1** could be embarked on and eventually completed [9]. Several precursor-induced biosyntheses [10–11] and chemical syntheses [11–12] have provided close to 20 hormaomycin analogues that have contributed to an understanding of the biosynthetic pathways, the conformational behavior in solution and the structure–activity relationship.

After the initial observation that hormaomycin (**1**) has a marked influence on the secondary metabolite production of various streptomycetes including the producing strain itself, and an exceptionally selective inhibitory effect on coryneform bacteria [3–4], an interesting antimalarial activity was discovered [13]. It was this property that led us to consider the synthesis of even more analogues of **1**. Bearing in mind that tri(di,mono)fluoromethyl substituents often enhance the activity of pharmacologically relevant compounds [14–17], we embarked on the project to synthesize analogues **8** of hormaomycin (**1**), in which the (2-nitrocyclopropyl)alanine moieties would be replaced by [2-tri(di,mono)fluoromethylcyclopropyl]alanine residues (Figure 1).

## Results and Discussion

**Figure 2:**
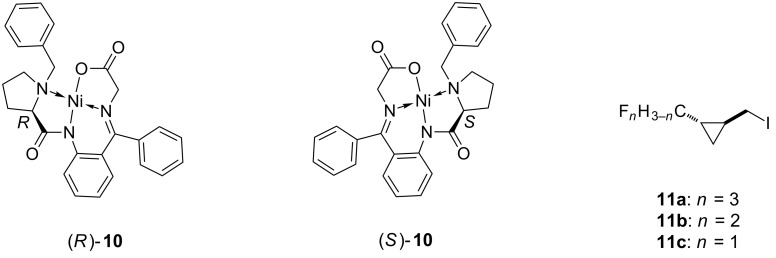
Structures of the Belokon'-type glycine complexes (BGC) (*R*)- and (*S*)-**10**.

As prerequisites for the hormaomycin analogues **8a**–**c**, the correctly configured two diastereomers each of the [2-tri(di,mono)fluoromethylcyclopropyl]alanines **9a**–**c** had to be synthesized in enantiomerically pure form. In view of the successful employment of the enantiomeric nickel(II) complexes (*R*)-**10** and (*S*)-**10** [18–20] in the synthesis of (2*R*,1*R*',2*R*')- and (2*S*,1*R*',2*R*')-3-(2-nitrocyclopropyl)alanine (**5**) for the native hormaomycin (**1**) [7,9], a completely analogous route was chosen towards (2*R*,1'*S*,2'*R*)- and (2*S*,1'*S*,2'*R*)-**9a**–**c**. This required the racemic *trans*-(2-fluoromethylcyclopropyl) iodides **11a**–**c** as alkylating agents for the enolates of (*R*)-**10** and (*S*)-**10** (Figure 2).

**Scheme 1:**
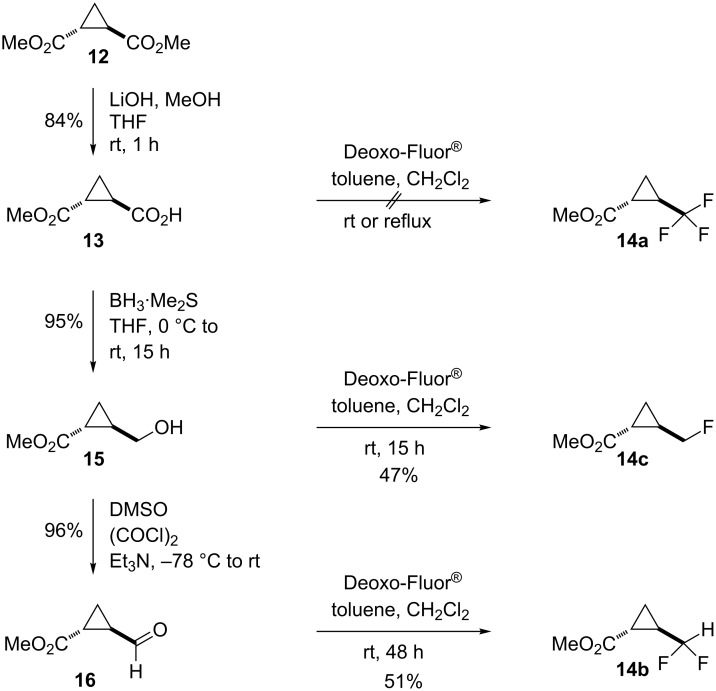
Intended routes to methyl *trans*-2-(fluormethyl)cyclopropanecarboxylates **14a**–**c**.

The initially intended preparations of the three precursors **14a**–**c** to the iodides **11a**–**c** all starting from the known dimethyl *trans*-cyclopropane-1,2-dicarboxylate (**12**) [21] through the monomethyl ester **13** [22], the hydroxymethyl **15** [22] and the formyl derivative **16** [23–24] as outlined in Scheme 1, were only partially successful.

As expected, the hydroxymethyl (**15**) and formyl derivative **16** underwent smooth conversion into the monofluoro- and difluoromethyl derivatives **14c** and **14b**, respectively upon treatment with a solution of Deoxo-Fluor in toluene at ambient temperature. Unfortunately, conducting the reaction with carboxylic acid **13** under the same conditions only provided the acid fluoride and not the trifluormethylated compound **14a**.

**Scheme 2:**
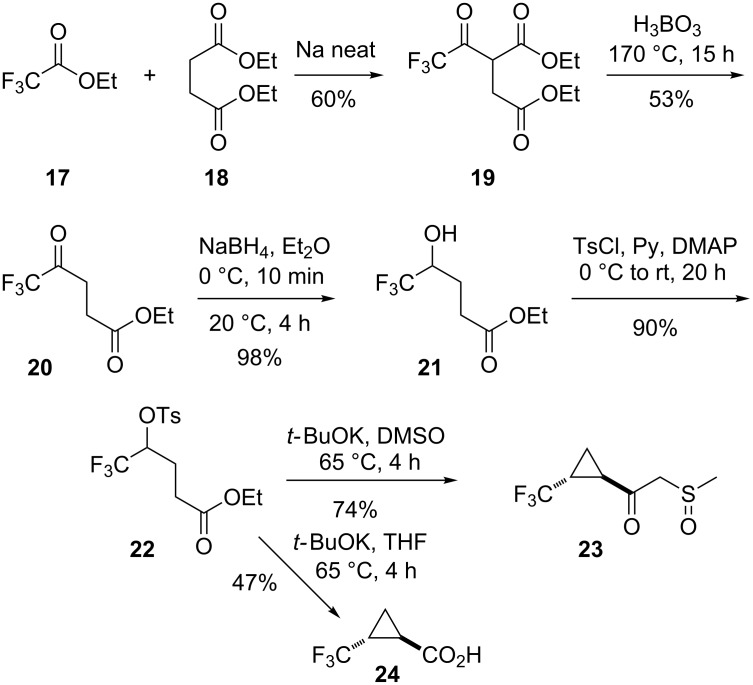
Synthesis of *trans*-(2-trifluoromethyl)cyclopropanecarboxylic acid (**24**).

Therefore, an alternative approach to *trans*-(2-trifluoromethyl)cyclopropanecarboxylic acid **23** by way of the Claisen condensation product of ethyl trifluoroacetate (**17**) with diethyl succinate (**18**) [25] was used. The conditions for two of the known further steps [26–27] (Scheme 2) had to be modified to achieve acceptable yields. The reduction of the ketoester **20** to the hydroxyester **21** under the previously described conditions (H_2_/PtO_2_) proceeded very slowly, and in several runs (2 h, 24 h, 72 h) the yield of **21** was never better than 60% with up to 25% recovered ketoester **20**. However, with powdered sodium borohydride in diethyl ether, the conversion of **20** was quantitative, and the yield of **21** was excellent (98%). In the final step, the attempted 1,3-dehydrotosylation of **22** with potassium *tert*-butoxide, when carried out in dimethyl sulfoxide as reported [26–27], compound **23** as an intermolecular condensation product of the expected ethyl *trans*-(2-trifluoromethyl)cyclopropanecarboxylate with DMSO was obtained in 74% yield. Among several other base/solvent combinations tested – NaOEt/EtOH, NaOMe/MeOH, *t*-BuOK/*t*-BuOH, NaH/THF, *t*-BuOK/THF under reflux – the last one gave the best results with up to 47% yield of the *trans*-(2-trifluoromethyl)cyclopropanecarboxylic acid (**24**).

The conversion of the carboxylic acid **24** and esters **14b**,**c** to the corresponding cyclopropylmethyl alcohols was attempted according to the standard protocol by adding the respective substrate to a twofold excess of LiAlH_4_ in diethyl ether under reflux. (2-Trifluoromethylcyclopropyl)methanol (**25a**) thus was obtained in excellent yield (88%), but the difluoromethyl- (**25b**) and especially monofluoromethylcyclopropylmethanol **25c**, respectively, were obtained from the corresponding methyl cyclopropanecarboxylates **14b** and **14c**, respectively, in very poor yields (3% and 4%, respectively). In the case of monofluoro derivative **14c** the main product (38%) was *trans*-(2-methylcyclopropyl)methanol. In the case of the difluoro compound **14b**, a mixture of the mono- (**25c**) and difluoromethylcyclopropylmethanol **25b** along with the non-fluorinated alcohol was obtained in a ratio of approximately 1:1:1.

**Scheme 3:**
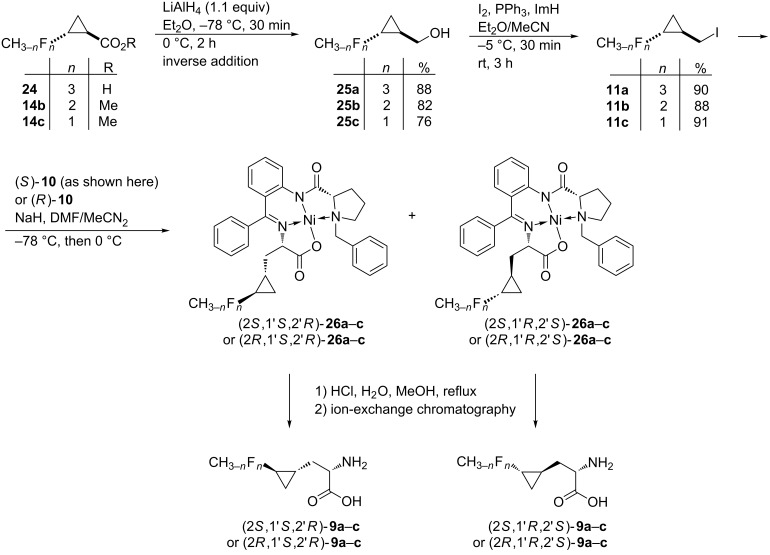
Preparation of racemic *trans*-2-(fluoromethyl)cyclopropylmethyl iodides **11a**–**c** and their conversion to (2*S*,1'*S*,2'*R*)- and (2*R*,1'*S*,2'*R*)-3-(*trans*-2'-fluoromethylcyclopropyl)alanines **9a**–**c** (only (2*S*)-enantiomers are shown in the Scheme). Compounds (*S*)-**10** and (*R*)-**10** are shown in Figure 2. For details see Table 1.

To avoid this overreduction, inverse addition of 1.1 equiv of LiAlH_4_ in diethyl ether solution (ca. 1 M) to a solution of the acid **24** or the respective ester **14b**,**c** in diethyl ether (ca. 1 M) was practiced. This way, the desired alcohols **25a**–**c** were obtained in good yields (88, 82 and 76%, respectively). Upon treatment with the iodine/triphenylphosphine reagent in the presence of imidazole, the racemic *trans*-(2-fluoromethylcyclopropyl)methanols **25a**–**c** were smoothly converted to the corresponding iodides **11a–c** in very good yields (Scheme 3).

**Table 1:** Alkylation of the enolates of the Belokon'-type glycine equivalents (*S*)- and (*R*)-**10** with the racemic *trans*-2-(fluoromethyl)cyclopropylmethyl iodides *rac*-**11a**–**c** (see Scheme 3). Yields in % based on converted (*S*)- and (*R*)-**10**.

	from (*S*)-**10**		from (*R*)-**10**	

Iodide	2*S*,1'*S*,2'*R*	2*S*,1'*R*,2'*S*	2*R*,1'*S*,2'*R*	2*R*,1'*R*,2'*S*

*rac*-**11a**	46	49	44	42
*rac*-**11b**	45	48	47	45
*rac*-**11c**	44	45	47	42

**Figure 3:**
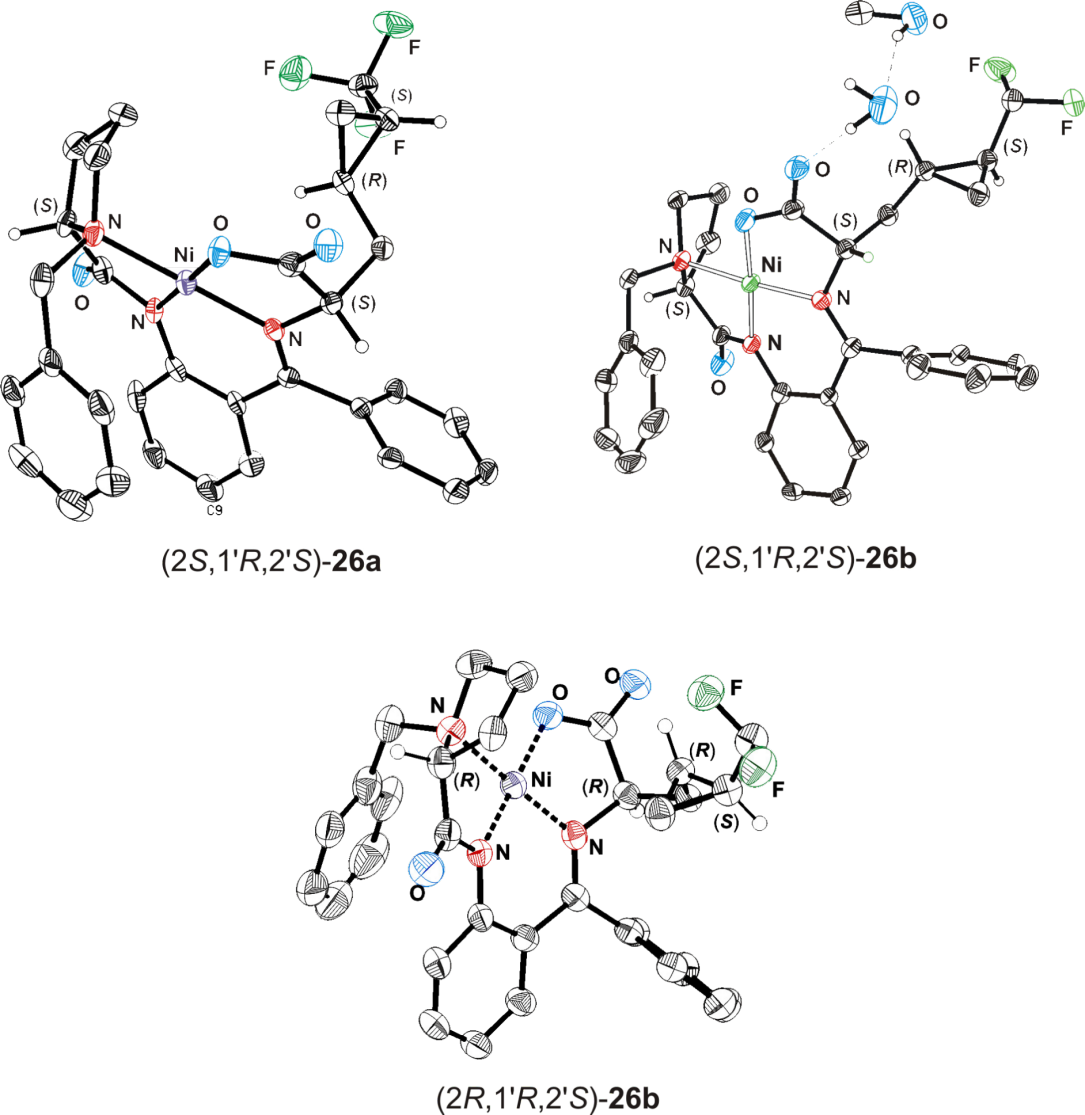
Structure and absolute configurations of the nickel(II) complexes (2*S*,1'*R*,2'*S*)-**26a**, (2*S*,1'*R*,2'*S*)-**26b** and (2*R*,1'*R*,2'*S*)-**26b** in the crystals. Less important hydrogen atoms are omitted for clarity.

Alkylation of the glycine equivalent enolates derived from (*S*)- and (*R*)-2-[(*N*-benzylprolyl)amino]benzophenone [(*S*)- and (*R*)-**10**] as reusable chiral auxiliaries with the racemic iodides **11a**–**c**, employing the protocol of Larionov and de Meijere [7], in each case led to a mixture of diastereomeric products, which were separated by column chromatography. Unfortunately, the diastereomers could not be separated by fractional crystallization as easily as was previously reported for the corresponding 3-(*trans*-2-nitrocyclopropyl)alanine derivatives [7]. The absolute configuration of the arbitrarily selected nickel(II) complexes (2*S*,1'*R*,2'*S*)-**26a**, (2*S*,1'*R*,2'*S*)-**26b** and (2*R*,1'*R*,2'*S*)-**26b** were determined by a single crystal X-ray structure analysis (see Figure 3 and Supporting Information File 1) [28].

The isolated nickel complexes **26a**–**c** were decomposed by treatment with refluxing aqueous methanolic hydrogen chloride to give, after ion-exchange chromatography, the corresponding (2*S*,1'*S*,2'*R*)- [see Scheme 3, derived from (*S*)-**10**] and (2*R*,1'*S*,2'*R*)-3-(2'-fluoromethylcyclopropyl)alanines [derived from (*R*)-**10**] in good to excellent yields. The chiral auxiliary was recovered as the hydrochloride of 2-[(*N*-benzylprolyl)amino]benzophenone (~95%).

(*R*)-*allo-*Threonine (**4**) is commercially available, but extremely expensive (from 77.80 € for 250 mg to 60.80 € for 25 mg). Therefore a simple and inexpensive access to (*R*)-*allo*-threonine was desirable.

The synthesis of **4** from (*R*)-threonine as a chiral precursor was performed according to the known protocol reported by Tanner et al [29]. Although (*R*)-threonine is less expensive (21–37 € for 5 g) than the target amino acid, the conversion requires five steps, and the overall yield is not better than 72%.

The Belokon' protocols are among the best to access enantiomerically pure non-proteogenic amino acids. Nickel(II) or copper(II) complexes of Schiff bases derived from glycine and (*S*)- or (*R*)-2-*N*-(*N*'-benzylprolyl)aminobenzophenone (BPB) [30–31], aminoacetophenone (BPA) [32] or aminobenzaldehyde (BPH) [33] can be used as chiral nucleophilic glycine equivalents in reactions with alkyl halides or carbonyl compounds. The most versatile one is the nickel(II) aminobenzophenone derivative.

It is interesting that nickel(II) complexes of Schiff bases derived from 2-bromoglycine and (*S*)-BPB can be used as electrophilic glycine equivalents [34]. Alkylations of the nickel(II) complexes of Schiff bases derived from glycine and (*S*)- or (*R*)-BPB with alkyl halides virtually yield single stereoisomers, in which the configuration of the newly formed stereogenic center at C-2 of the amino acid moiety is the same as that in the proline moiety of the chiral auxiliary in the starting material.

In reactions of the enolate of this chiral glycine equivalent with aldehydes the situation is more complicated. The reaction of (*S*)-**10** with acetaldehyde under strongly basic conditions led to the (*R*)-threonine complex **29** (inverse configuration relative to that of the proline moiety of (*S*)-**10** due to epimerization on C-2), but when a weaker base such as triethylamine was employed, a mixture of (*R*)-threonine **29** and (*S*)-*allo*-threonine **31** complexes [35] was obtained.

**Figure 4:**
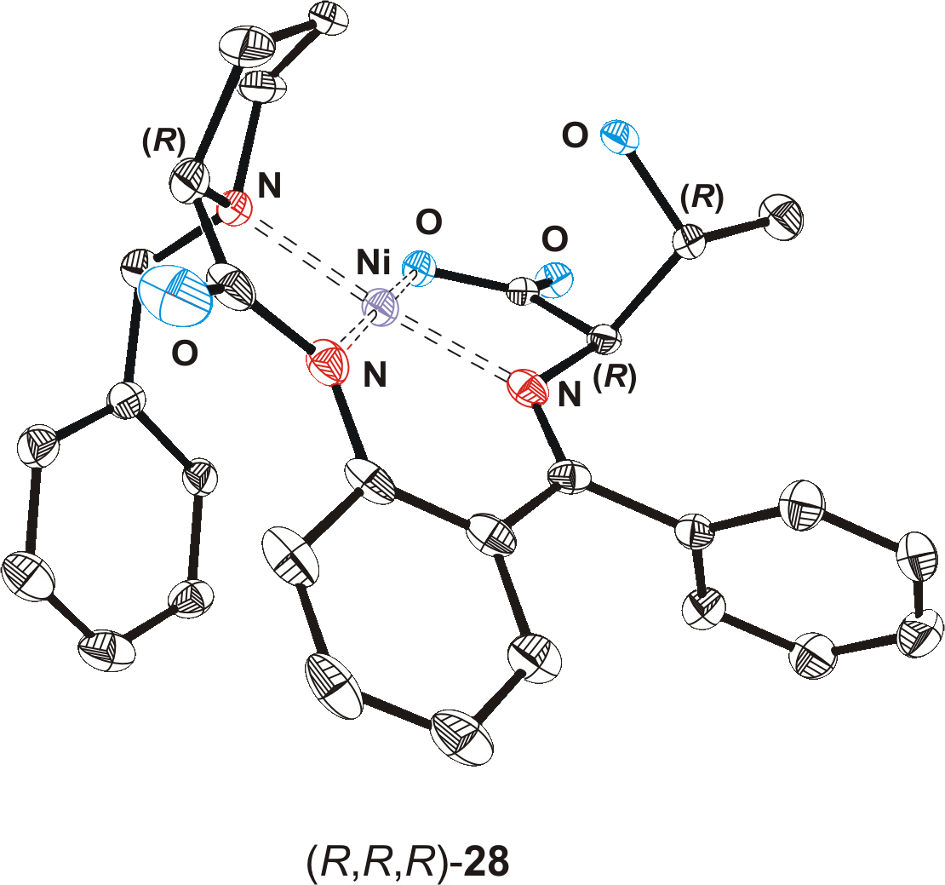
Structure and absolute configuration of nickel(II) complex (*R*,*R*,*R*)-**28** in the crystal. Hydrogen atoms are omitted for clarity.

**Scheme 4:**
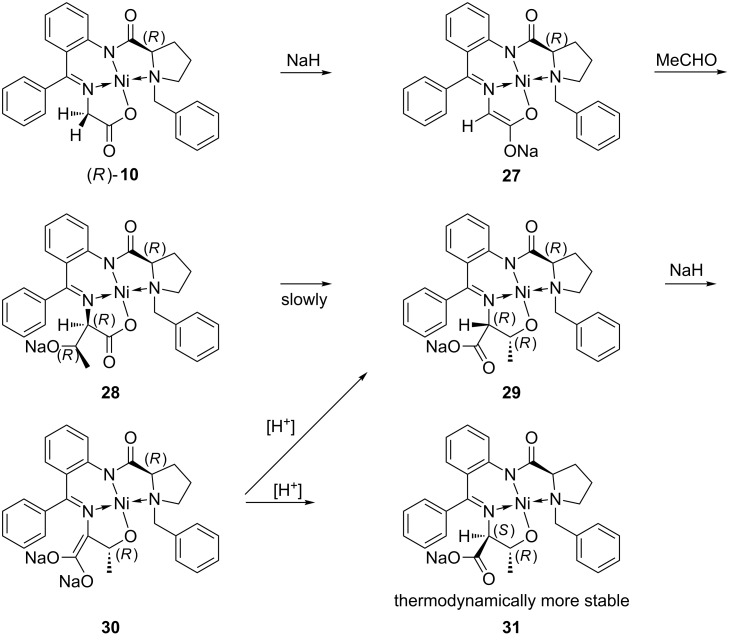
Mechanism of epimerization of the threonine nickel(II) complex **29**.

The hypothesis, that the reaction of the Belokon' glycine complex (BGC) **10** with aldehydes under strongly basic conditions proceeds in two steps and is thermodynamically controlled, was corroborated by experimental tests [36]. The initially formed main product (*R*,*R*,*R*)-**28** in the aldol reaction of acetaldehyde with **10** had the same configuration at C-2 as the proline unit in **10**. The absolute configuration of this nickel(II) complex was determined by a single crystal X-ray structure analysis (see Figure 4 and Supporting Information File 1) [28]. However, the product ratio changed in time from 95:5 after 30 s through 70:18 after 10 min to 5:95 after 24 h at ambient temperature. This epimerization comes along with a possible rearrangement in the Ni complex. The newly formed hydroxide group of the product **28** can coordinate the Ni atom liberating the carboxylate moiety and thus making the proton at C-2 accessible to base attack (Scheme 4). In order to obtain (*R*)-*allo*-threonine (**4**), it is necessary to carry out the aldol reaction of (*R*)-**10** with an excess of acetaldehyde under strongly basic conditions at low temperature and to quench the reaction after a short time to avoid epimerization of **28**.

This modified protocol indeed gave the (*R*)-*allo*-threonine (**4**) in relatively poor yield [7.5% for the Ni complex, 91% (7% overall) for the amino acid], but with high enantiomeric purity in two steps. Bearing in mind that the starting materials are inexpensive and the chiral auxiliary is reusable (≥95% recovery), this protocol represents one of the best routes to the rather expensive (*R*)-*allo*-threonine (**4**).

It is also possible to obtain (*R*)-*allo*-threonine starting from (*R*)-**10** and acetaldehyde under thermodynamic control (Et_3_N as the base, (*S*)-threonine:(*R*)-*allo*-threonine = 1:7), but it is necessary to leave the reaction mixture for two months for the reaction to go to completion [37].

(2*S*,3*R*)-3-Methylphenylalanine (L-β-methylphenylalanine, (β-Me)Phe, MeF, **3**) also is a constituent of the peptidolactone hormaomycin (**1**) and is contained in the molecule twice. Thus it is required for the synthesis of hormaomycin and the analogues envisaged here. In addition, a versatile protocol for the preparation of other β-alkylarylalanines for incorporation into hormaomycin analogues as well as into other peptides would be desirable as the incorporation of conformationally constrained α-amino acids such as **3** into peptides is frequently used to study structure–activity relationships [38–40].

Several methods have been developed for the preparation of analogues of β-methylphenylalanine in enantiopure form. These include classical resolution [41], enzymatic resolution in conjunction with HPLC [42], or HPLC separation of derived peptides [43], preparative HPLC separation on a chiral phase column [44], asymmetric synthesis from chiral precursors [45–46] including the stereoselective alkylation of aromatic compounds with triflates of threonine stereoisomers [47], the chiral auxiliary approach [48–51] and enantioselective hydrogenation over a chiral catalyst [52–53].

All these approaches ought to be applicable to prepare unsubstituted β-methylphenylalanine, but most if not all of them have severe drawbacks. Among the chiral auxiliary approaches to β-branched arylalanines, including all four stereoisomers of β-methylphenylalanine, the one employing the "Evans amide" method with a 4-benzyl- or 4-phenyl-2-oxazolidinone moiety, has been used most frequently. Along this route, which requires eight procedural steps (including a transmetallation), the (2*S*,3*R*)-3-methyl-3-phenylalanine required for hormaomycin and its analogues, has previously been utilized [9,54–55].

**Scheme 5:**
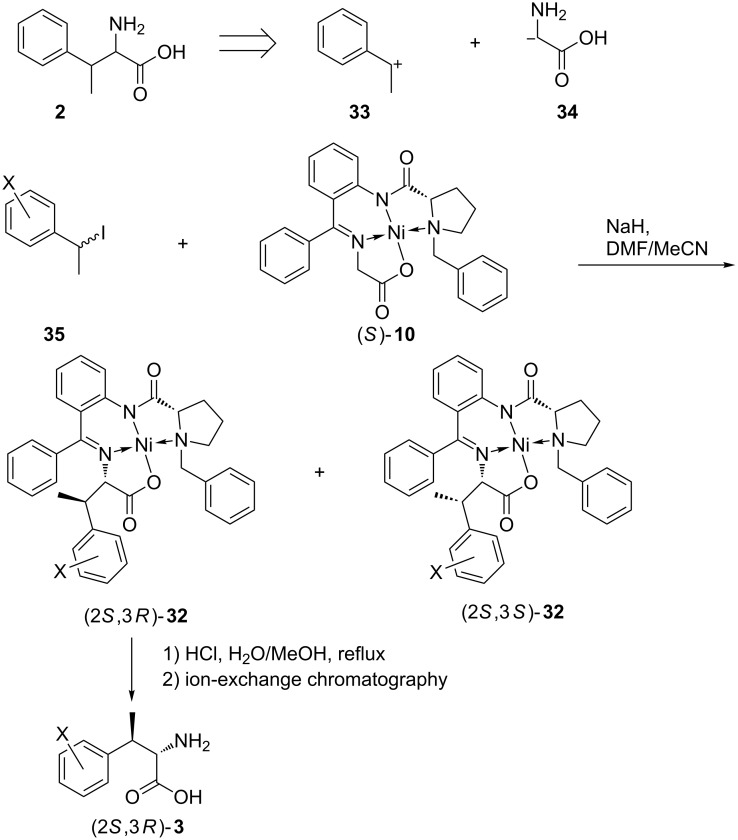
A new general approach to (2*S*,3*R*)-β-methylarylalanines **3** by alkylation of the glycine nickel(II) complex (*S*)-**10** with 1-arylethyl iodides **35**. For details see Table 2.

In view of the good performance of the Belokoń protocol for various electrophilic reagents it appeared attractive to apply it for the synthesis of β-methylphenylalanines as well (Scheme 5). Towards this, the (*S*)-configured glycine nickel(II) complex (*S*)-**10** was alkylated with 1-phenylethyl iodide and some analogues with substituents in the aryl moiety, all in racemic form. The diastereomeric product Ni(II) complexes obtained in each case, could be separated by column chromatography. The pure diastereomers with (2*S*,3*R*) configuration were decomposed with an aqueous methanolic HCl solution to furnish the target amino acids, which were purified by ion-exchange chromatography, in good yields (Table 2). (2*S*,3*R*)-β-Methylphenylalanine was thus prepared from acetophenone in only four steps in an overall yield of 30%.

**Table 2:** Substituted β-methylphenylalanines by alkylation of the glycine nickel(II) complex (*S*)-**10** with 1-arylethyl iodides (yields based on converted **10**, d.e. ≥98%). See Scheme 5. The yields of the liberated amino acids **3** based on the respective Ni complexes **32** are in parentheses.

X	Product	Yield (%)	Product	Yield (%)

H	(2*S*,3*S*)-**32**^a^	35	(2*S*,3*R*)-**32**	38 (59)
*o*-Cl	(2*S*,3*S*)-**32**-*o*-Cl	38	(2*S*,3*R*)-**32**-*o*-Cl	42
*m*-Cl	(2*S*,3*S*)-**32**-*m*-Cl	37	(2*S*,3*R*)-**32**-*m*-Cl	42 (96)
*p*-Cl	(2*S*,3*S*)-**32**-*p*-Cl	42	(2*S*,3*R*)-**32**-*p*-Cl	40 (89)
*p*-F	(2*S*,3*S*)-**32**-*p*-F	46	(2*S*,3*R*)-**32**-*p*-F	43

^a^The absolute configurations of the arbitrarily selected nickel(II) complexes (2*S*,3*S*)-**32**, (2*S*,3*R*)-**32**-*m*-Cl and (2*S*,3*R*)-**32**-*p*-F were determined by single crystal X-ray structure analyses (see Figure 5 and CCDC-deposited material) [28].

**Figure 5:**
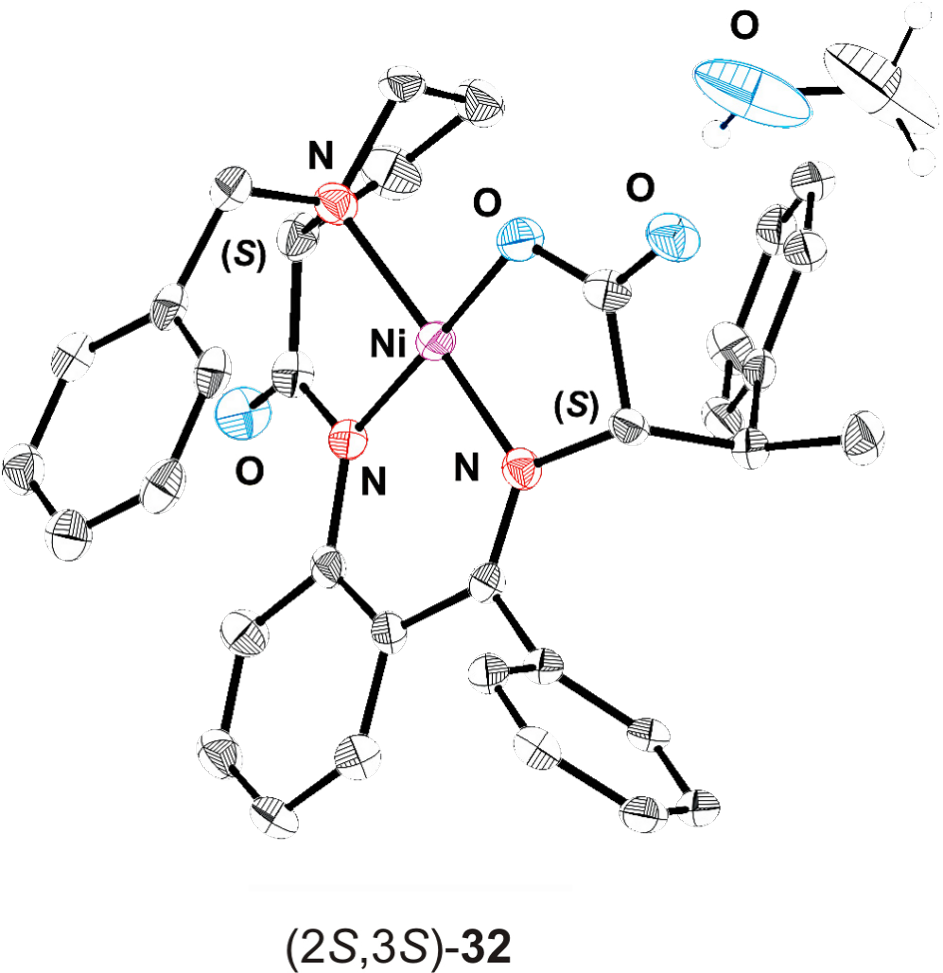
Structure and absolute configuration of nickel(II) complex (2*S*,3*S*)-**32** in the crystal. Hydrogen atoms are omitted for clarity.

A similar protocol for the synthesis of 3-alkylphenylalanines was independently developed by Soloshonok et al. [56]. These authors used sodium hydroxide for the deprotonation of **10** and 1-phenylalkyl bromides for the alkylation of the enolate.

Once sufficient quantities of (2*S,*1'*S,*2'*R*)-**9a**–**c** and (2*R,*1'*S,*2'*R*)-**9a**–**c** (FmcpA), as well as the *N*-Boc-protected (2*S,*4*R*)-4-(*Z*)-propenylproline [(4-Pe)Pro, **6**] [8], the *O*-MOM-protected 5-chloro-1-hydroxypyrrole-2-carboxylic acid (Chpca, **7**) [8], (*R*)-*allo*-threonine (*allo*-Thr, **4**) and (2*S*, 3*R*)-β-methylphenylalanine [(β-Me)Phe, **3**] had been prepared. The assembly of the hormaomycin analogues **8a**–**c** with 3-(2'-fluoromethylcyclopropyl)alanine residues was initiated, employing the same sequence as developed by Zlatopolskiy for the synthesis of hormaomycin **1** [9] and its aza-analogue [12]. To start with, the dicyclopropylmethyl (DCPM) ester of *N*-Fmoc-protected Ile **37**, was condensed with *N*-Z-protected (βMe)Phe-OH **39**. After removal of the Z group from the *N*-terminus of the resulting dipeptide **42** by catalytic hydrogenation, the product was coupled with *N*-Fmoc-protected (2*R*,1'*R*,2'*R*)-[3-(mono-, di- or tri-)fluoromethylcyclopropyl]alanines **41a**–**c** to yield tripeptides **47a**–**c**, which, in turn, after deprotection with Et_2_NH/THF, were coupled with *N*-Fmoc-protected (βMe)Phe-OH **46** to give *N*,*C*-protected tetrapeptides **49a**–**c**.

The *N*-Boc-protected (4-Pe)Pro-OH **43** and *N*,*C*-protected *allo*-Thr **40** were condensed under 4-pyrrolidinopyridine catalysis to give the ester **45**, which, after deallylation under palladium catalysis, was coupled with the tetrapeptides employing the HATU reagent in the presence of HOAt to give the corresponding hexadepsipeptides **51a**–**c**.

**Scheme 6:**
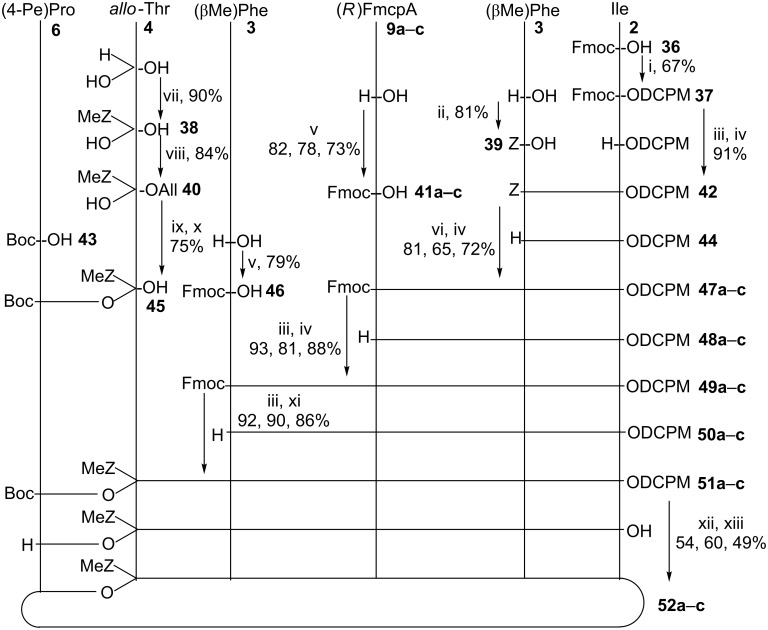
Synthesis of the cyclohexadepsipeptides **52a**–**c** for the hormaomycin analogues **8a–c** with 3-(2'-fluoromethylcyclopropyl)alanine residues. **a**: trifluoromethyl-, **b**: difluoromethyl-, **c**: monofluoromethylcyclopropylalanine. Reagents and conditions: i) oxalyl chloride, pyridine/dicyclopropylmethanol, DMAP, CH_2_Cl_2_, 0*→*20 °C, 20 h; ii) ZOSu, NaHCO_3_, acetone/water, 2 h; iii) 50% Et_2_NH/THF, 20 °C, 1 h; iv) EDC, HOAt, 2,4,6-collidine, CH_2_Cl_2_, 4*→*20 °C, 14 h; v) FmocOSu, NaHCO_3_, acetone/water, 3 h; vi) H_2_, Pd/C, EtOAc, 20 °C, 2 h; vii) MeZOSu, NaHCO_3_, water/dioxane, 20 °C, 3 h; viii) All-Br, K_2_CO_3_, MeCN, 85 °C, 3 h, 60 °C, 16 h; ix) EDC, 4-pyrrolidinopyridine, CH_2_Cl_2_, 4*→*20 °C, 16 h; x) [Pd(PPh_3_)_4_], *N*-methylaniline, DME, 20 °C, 1 h; xi) HATU, HOAt, DIEA, 2,4,6-collidine, CH_2_Cl_2_, 4*→*20 °C, 15 h; xii) 2 M HCl in EtOAc, 20 °C, 20 min; xii) HATU, HOAt, DIEA, 2,4,6-collidine, CH_2_Cl_2_, 4*→*20 °C, 22 h. Boc = *tert*-butyloxycarbonyl, DCPM = dicyclopropylmethyl, Fmoc = 9-fluorenylmethyloxycarbonyl, DIEA = *N*,*N*-diisopropylethylamine, DMAP = 4-dimethylaminopyridine, EDC = *N*'-(3-dimethylaminopropyl)-*N*-ethylcarbodiimide hydrochloride, HATU = *O*-(7-azabenzotriazole-1-yl)-*N*,*N*,*N*',*N*'-tetramethyluronium hexafluorophosphate, HOAt = 7-aza-1-hydroxybenzotriazole, MeZ = (4-methylbenzyl)oxycarbonyl, Z = benzyloxycarbonyl.

From the latter, the DCPM and Boc groups were cleaved off from both termini, leaving the MeZ group intact as proved by an ESIMS spectrum, and the cyclizing peptide condensation was achieved under high dilution conditions with the HATU reagent. The cyclohexadepsipeptides **52a**–**c** were obtained in 54, 60 and 53%, respectively, yield over 8 steps, after HPLC purification (Scheme 6).

**Scheme 7:**
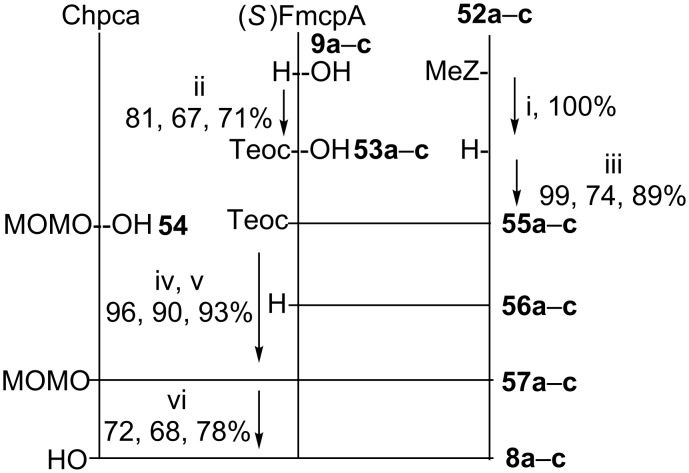
Synthesis of hormaomycin analogues with **a**: trifluoromethyl-, **b**: difluoromethyl-, **c**: monofluoromethylcyclopropylalanine residues. Reagents and conditions: i) anisole, TFA, 20 °C, 2 h; ii) TeocOSu, NaHCO_3_, *N*,*N*-dimethylaminopropylamine, water/acetone, 20 °C, 2 h; iii) HATU, HOAt, DIEA, 2,4,6-collidine, CH_2_Cl_2_, 20 °C, 15 h; iv) TFA, 20 °C, 1 h; v) HATU, DIEA, 2,4,6-collidine, CH_2_Cl_2_, 20 °C, 4 h; vi) MgBr_2_·Et_2_O, EtSH, CH_2_Cl_2_, 20 °C, 3.5 h. DIEA = *N*,*N*-diisopropylethylamine, HATU = *O*-(7-azabenzotriazole-1-yl)-*N*,*N*,*N*',*N*'-tetramethyluronium hexafluorophosphate, HOAt = 7-aza-1-hydroxybenzotriazole, MeZ = (4-methylbenzyl)oxycarbonyl, MOM = methoxymethyl, Teoc = (2-trimethylsilylethyl)oxycarbonyl.

The assemblies of the corresponding hormaomycin analogues were completed after removal of the *N*-MeZ groups from the cyclic intermediates **52a–c**, subsequent coupling with the corresponding *N*-Teoc-protected (2*S*,1'*R*,2'*R*)-[3-(mono-, di- or tri-)fluoromethylcyclopropyl]alanines **53a–c** and, after removal of the Teoc groups, the intermediates **56a**–**c** were in turn coupled with the 1-*O*-MOM-protected 5-chloro-1-hydroxypyrrole-2-carboxylic acid **54**. Eventually, the MOM group was cleaved off by treatment with MgBr_2_∙Et_2_O and EtSH in dichloromethane to give, after HPLC purification, the target compounds **8a**–**c** in 72, 82 and 84% yield, respectively (Scheme 7).

**Figure 6:**
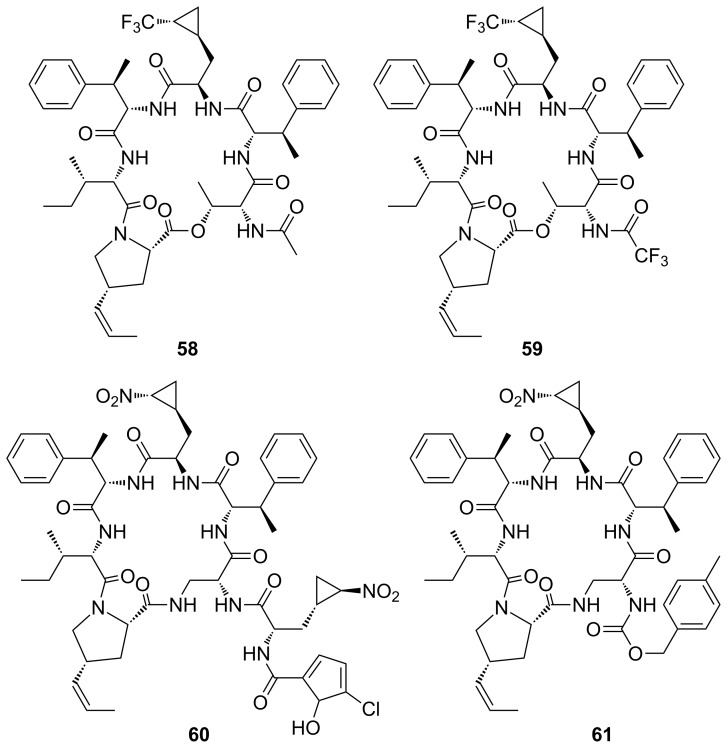
Two derivatives **58** and **59** of cyclohexadepsipeptide **52a** containing the (trifluoromethylcyclopropyl)alanine moiety, the aza-analogue **60** of hormamycin (**1**) itself as well as a simplified analogue **61** for biological testing.

Since the MeZ-protected cyclohexadepsipeptide core of the native hormaomycin was found to have a significant antiparasitic activity, *N*-acetylated **58** and *N*-trifluoroacetylated **59** derivatives were prepared by coupling the deprotected cyclic intermediate **52a** with acetic and trifluoroacetic acid (Figure 6).

### Some antiparasitic activities of hormaomycin, its all-peptide analogue and the new analogues

The syntheses of hormaomycin (**1**) itself and of its all-peptide aza-analogues **60** and **61** (Figure 6) as developed by Zlatopolskiy et al. [9,12], were reproduced in order to provide large enough quantities for biological tests of their antimalarial activities. Results of the in vitro tests of the antiparasitic activities of hormaomycin (**1**) [57], its analogues **8a**, **52a**, **58** and **59**, as well as the aza-analogues **60** and **61** are presented in Table 3.

**Table 3:** In vitro activities of some hormaomycin-derived compounds [58] and established reference drugs against *L. donovani* (axenic amastigotes), *P. falciparum* and L6 cells (IC_50_, concentration in µg/mL).^a^

Compound	*Leishmania donovani* strain MHOM-ET-67/L82	*Plasmodium falciparum* strain K1	L6 cells

Miltefosine	0.143	–	–
Chloroquine	–	0.089	–
Podophyllotoxin	–	–	0.006
**1**	0.15	0.129	17.20
**52a**	2.13	0.042	>90
**58**	1.73	0.151	>90
**8a**	0.205	0.183	40
**59**	2.37	0.265	>90
**60**	4.8	0.023	?
**61**	–	0.061	?

^a^IC_50_ values reported are the averages of two independent assays which varied less than ±50%.

All the newly prepared and tested hormaomycin analogues and the native hormaomycin (**1**) [57] showed good and selective antimalarial activities. Compounds **8a** and **1** additionally showed an activity against *L. donovani*. However, the activities of the (trifluoromethylcyclopropyl)alanine-containing hormaomycin analogue **8a** against *L. donovani* and *P. falciparum* were 37 and 45% lower than those of hormaomycin (**1**) itself. Further exploration of structure–activity relationships of the hormaomycins would be required to prepare new antiparasitic lead compounds.

## Conclusion

At first sight, the oligopeptide assembly leading to hormaomycin does not appear to be a very complicated problem. "State of the art" peptide coupling methodology [59–60] allows one to prepare almost any peptides, that do not contain extremely sterically congested fragments such as α,α-dialkyl-substituted amino acids, *N*-alkyl amino acids or even the more challenging *N*-aryl amino acids. With a proper choice of the coupling reagent, solvent and other experimental conditions, the oligopeptides in this study were obtained in high yields and with high optical purities. As almost all amino acids, which comprise hormaomycin (**1**) itself and its anticipated analogues, are β-branched with the exception of 3-(2'-nitrocyclopropyl)alanine and the 3-(2'-fluoromethylcyclopropyl)alanines, HATU as well as the combination of EDC and 7-aza-1-hydroxybenzotriazole (HOAt) [61] were used for each condensation step to ensure high yields. The most unusual fragment in hormaomycin (**1**) and its analogues is the ester bond between the secondary (4-Pe)Pro moiety and the hydroxy group of *allo*-Thr. Among several methods described in the literature for the creation of such bonds, the dialkylaminopyridine-promoted carbodiimide-mediated esterification was successfully employed here [62].

As far as the biological activities against *L. donovani* and *P. falciparum* are concerned, a certain degree of lowering was observed, but by far no complete loss. Thus, further modifications would be desirable to eventually arrive at a new lead compound.

## Experimental

For detailed experimental procedures of all the described syntheses see the Supporting Information File 1.

In vitro antiprotozoal activity assays: The in vitro activities against the protozoan parasites *L. donovani* and *P. falciparum* as well as cytotoxicity assessments against L6 cells were determined as reported elsewhere [58]. The following strains, parasite forms and positive controls were used: *L. donovani*, MHOM/ET/67/L82, axenic amastigote forms, miltefosine, IC_50_ of 0.143 μg/mL; *P. falciparum*, K1 (chloroquine and pyrimethamine resistant), erythrocytic stages, chloroquine, IC_50_ of 0.089 μg/mL and L6 cells, rat skeletal myoblasts, podophyllotoxin, IC_50_ of 0.006 μg/mL.

## Supporting Information

File 1 Experimental procedures and analytical data.
