# Supplementary material for: (2R,1'S,2'R)- and (2S,1'S,2'R)-3-[2-Mono(di,tri)fluoromethylcyclopropyl]alanines and their incorporation into hormaomycin analogues
Source: Beilstein J Org Chem. 2014 Dec 3;10:2844–57. doi: 10.3762/bjoc.10.302 (PMC4273223; doi:10.3762/bjoc.10.302)
Supplement: File 1 — Experimental procedures and analytical data. [file Beilstein_J_Org_Chem-10-2844-s001.pdf]

Supporting Information

for

(2*R*,1'*S*,2'*R*)- and (2*S*,1'*S*,2'*R*)-3-[2-Mono(di,tri)fluoromethylcyclopropyl]alanines and their incorporation into hormaomycin analogues

Armin de Meijere*[1], Sergei I. Kozhushkov[1], Dmitrii S. Yufit[2], Christian Grosse[3], Marcel Kaiser[4,5] and Vitaly A. Raev*[1,6]

Address: [1]Institut für Organische und Biomolekulare Chemie der Georg-August-Universität Göttingen, Tammannstrasse 2, 37077 Göttingen, Germany, [2]Department of Chemistry, University of Durham, South Rd., Durham DH1 3L, UK, [3]Institut für Anorganische Chemie der Georg-August-Universität Göttingen, Tammannstrasse 4, 37077 Göttingen, Germany, [4]Swiss Tropical and Public Health Institute, Parasite Chemotherapy, Socinstrasse 57, CH-4002 Basel, Switzerland, [5]University of Basel, Petersplatz, 1 CH-4003 Basel Switzerland and [6]Current address: Institut für Organische Chemie der TU Carolo-Wilhelmina zu Braunschweig, Hagenring 30, 38106 Braunschweig, Germany.

Email: Armin de Meijere - ameijer1@uni-goettingen.de; Vitaly A. Raev - v.raev@tu-braunschweig.de

*Corresponding author

This article is part 163 of the Series "Cyclopropyl Building Blocks for Organic Synthesis". For parts 162 and 161 see [1] and [2], respectively. Dedicated to Professor Max Malacria on the occasion of his 65th birthday

Experimental procedures and analytical data

Contents:

1. General remarks and procedures S2
2. Experimental procedures S4
3. X-ray Crystallography S51
4. ^{1}H-, ^{13}C-NMR, MS- and (in part) IR-spectra as well as specific rotations of representative compounds S56
5. References S108

General remarks

^{1}H NMR: Bruker AM 250 (250 MHz), Varian Unity 300 (300 MHz), Inova 500 (500 MHz), Inova 600 (600 MHz). ^{1}H chemical shifts are reported in ppm relative to residual peaks of deuterated solvents: δ (ppm) = 2.49 for [D_5]-DMSO, 4.65 for HOD in D_2O, 7.26 for $CHCl_3$, 1.73 and 3.55 for [D_7]-THF, 3.35 for CHD_2OD. Higher-order NMR spectra were approximately interpreted as first-order spectra, if possible. The observed signal multiplicities are listed as: s = singlet, d = doublet, t = triplet, q = quartet, quin = quintet, m = multiplet, as well as b = broad.

^{13}C NMR [additional DEPT (Distortionless Enhancement by Polarization Transfer) or APT (Attached Proton Test)]: Bruker AM 250 (62.9 MHz), AMX 300 (75.5 MHz) or Varian Unity 300 (75.5 MHz), Inova 500 (125.7 MHz), Inova 600 (125.7 MHz) instruments. ^{13}C chemical shifts are reported relative to peaks of deuterated solvents: δ (ppm) = 39.5 for [D_6]-DMSO, 77.0 for $CDCl_3$, 25.5 and 3.55 for [D_8]-THF, 3.35 for CD_3OD or to methanol in D_2O (δ = 49.5 ppm). The following abbreviations are applied: DEPT: "+" = primary or tertiary (positive signal in DEPT), "–" = secondary (negative signal in DEPT), Cquat = quaternary (no signal in DEPT); APT: "+" = primary or tertiary (positive signal in DEPT), "–" = secondary or quaternary (negative signal in APT).

IR spectra were recorded for KBr pellets or thin films between KBr plates on a Bruker IFS 66 (FT-IR) spectrometer.

MS: EI-MS: Finnigan MAT 95, 70 eV, high resolution EI-MS spectra with perfluorokerosene as reference substance; DCI-MS: Finnigan MAT 95, 200 eV, reactant gas NH_3; ESI-MS: Finnigan LCQ. HPLC-MS: pump: Flux Instruments Rheos 4000; degasser: Flux Instruments ERC 3415α; detector: Linear UVIS-205; data system: Flux Instruments Janeiro; ESI: Finnigan LCQ, positive and negative ion mode; data system: Finnigan LCQ Xcalibur; column: Crom Superspher 100 RP-18 endcapped (4 µm, 2 × 100 mm); HPLC conditions: eluent A: H_2O (0.1% TFA), eluent B: MeCN (0.1% TFA). Analytical HPLC: instrument Instrumentelle Analytik Goebel GmbH, autosampler SA 360, pump 420, detector Celeno DAD UV, software Geminyx Version 1.91, column Nucleodur® C18 (250 mm × 3 mm, 5 µm, 100 Å), flow rate 0.5 mL/min. Preparative HPLC: instrument Jasco, pump Jasco PU-1587, detector Jasco UV-1575, Software Jasco-BORWIN HSS-2000, column Nucleodur® C18 (250 mm × 20 mm, 5 µm, 100 Å), flow rate 18.0 mL/min.

Optical rotations: Perkin-Elmer 241 digital polarimeter, 1-dm cell; optical rotation values are given in 10^{-1} deg cm^2 g^{-1}; concentrations (c) are given in g/100 mL.

M. p.: Büchi 510 capillary melting point apparatus, uncorrected values.

TLC: Macherey-Nagel precoated sheets, 0.25 mm Sil G/UV254. The chromatograms were viewed under UV light and/or made visible by treatment with phosphomolybdic acid (10% in ethanol), or ninhydrine (0.2% in ethanol), or I_2 vapor.

Column chromatography: Merck silica gel, grade 60, 230–400 mesh and Baker silica gel, 40–140 mesh.

Elemental analyses: Mikroanalytisches Laboratorium des Instituts für Organische und Biomolekulare Chemie der Universität Göttingen.

Starting materials: Ethyl 5,5,5-trifluoro-4-oxovalerate **20** [3], ethyl 5,5,5-trifluoro-4-tosyloxyvalerate **22** [3]], *N*-Boc-protected (2*S*,4*R*)-4-(*Z*)-propenylproline [(4-Pe)Pro] **43** [4], *O*-MOM-protected 5-chloro-1-hydroxypyrrole-2-carboxylic acid (Chpca) **54** [4], dicyclopropylmethyl ester of *N*-Fmoc-protected Ile **37** [5], *N*-Z-protected (βMe)Phe-OH **39** [5], dipeptide **42** [5], *N,C*-protected *a*-Thr **40** [5] and ester **45** [5]] were prepared as described elsewhere. Anhydrous solvents were prepared according to standard methods by distillation over drying agents and were stored under nitrogen. All other solvents were distilled before use. All reactions were carried out with magnetic stirring and, when employing air- or moisture- sensitive materials, in flame-dried glassware under argon or nitrogen atmosphere.

List of used abbreviations: DCPM = dicyclopropylmethyl, Fmoc = 9-fluorenylmethyl-oxycarbonyl, DIEA=*N*,*N*-diisopropylethylamine, DMF = *N*,*N*-dimethylformamide, EDC = *N*'-(3-dimethylaminopropyl)-*N*-ethylcarbodiimide hydrochloride, HATU = *O*-(7-azabenzotriazole-1-yl)-*N*,*N*,*N*',*N*'-tetramethyluronium hexafluorophosphate, HOAt = 7-aza-1-hydroxybenzotriazole, MeZ = *p*-methylbenzyloxycarbonyl, MOM = methoxymethyl, Teoc = (2-trimethylsilylethyl)-oxycarbonyl, TFA = trifluoroacetic acid, TMP = 2,4,6-collidine, Z = benzyloxycarbonyl, ZOSu = *O*-benzyloxycarbonyl-1-hydroxypyrrolidine-2,5-dione.

General synthetic protocols

Deprotection of N-Fmoc-protected peptides (GP 1)

Similar as in [5], the respective protected peptide (1 mmol) was taken up with acetonitrile or THF (2 mL); diethylamine (2 mL) was added, and the resulting mixture left at ambient temperature for 40 min. All volatiles were evaporated under reduced pressure; the residue was taken up with toluene (2 × 5 mL), which was evaporated under reduced pressure to remove the last traces of diethylamine. The obtained crude *N*-deprotected peptide was directly used in the next condensation step.

Peptide condensation using EDC/HOAt (GP 2)

Similar as in [5], EDC (1.03 mmol) and HOAt (1.05 mmol) were added to a cooled (4 °C) solution of the respective *N*-protected amino acid (1 mmol) in anhydrous CH_2Cl_2 (3 mL). After 20 min, a solution of the respective crude *N*-deprotected peptide (0.97 mmol) and TMP (3 mmol) in anhydrous CH_2Cl_2 (1 mL) was added at the same temperature. The temperature was allowed to reach 20 °C, and stirring was continued for 15 h. Then the reaction mixture was diluted with diethyl ether or ethyl acetate (30 mL) and washed with water (2 × 5 mL), 1 M $KHSO_4$ (3 × 5 mL), water (2 × 5 mL), 5% aqueous $NaHCO_3$ solution (3 × 5 mL), water (3 × 5 mL), brine (2 × 5 mL), dried over $MgSO_4$, filtered and concentrated under reduced pressure. The residue was purified by column chromatography or recrystallization.

Preparation of hexadepsipeptides and hexapeptides using HATU/HOAt mediated coupling (GP 3)

The respective deprotected (according to GP 1) tetrapeptide (0.100 mmol) was dissolved in anhydrous CH_2Cl_2 (3 mL), the ester acid / dipeptide acid (0.110 mmol), HATU (0.107 mmol) and HOAt (0.110 mmol) were added, and the mixture was cooled to 4 °C. DIEA (0.110 mmol) and TMP (0.300 mmol) were then added, the mixture was allowed to warm to 20 °C and stirring continued for an additional 15 h. The mixture was then taken up with Et_2O (40 mL), and after the usual aqueous work-up (GP 2), the organic layer was concentrated to leave crude hexadepsipeptide / hexapeptide, which was purified by recrystallization and/or column chromatography.

Preparation of cyclohexadepsipeptides (GP 4)

The respective acyclic hexadepsipeptide (105 µmol) was deprotected by stirring with a 2 M HCl solution in ethyl acetate (2 mL) at 20 °C for 1 h in a dark place and subsequent concentration under reduced pressure to leave a solid residue. The latter was taken up in CH_2Cl_2 (1.0 L). The solution was cooled to 4 °C (internal temperature), HATU (122 µmol) and HOAt (104 µmol) were added, the mixture was stirred for 30 min, and then a solution of

DIEA (305 µmol) in CH_2Cl_2 (50 mL) was added over a period of 30 min. The cooling bath was removed, and stirring was continued for an additional 2 h at ambient temperature. Then the reaction mixture was cooled again to 4 °C and second portions of HATU (122 µmol) and HOAt (104 µmol) were added, followed by a solution of DIEA (305 µmol) in CH_2Cl_2 (50 mL) was added over a period of 30 min. The temperature was allowed to reach 20 °C, and stirring was continued for 15 h. After this, the solvent was removed under reduced pressure, the residue was taken up with diethyl ether (50 mL), subjected to the usual aqueous work-up (see GP 2) and concentrated under reduced pressure to give the crude product, which was finally purified by preparative HPLC.

Deprotection of N-MeZ protected cyclohexadepsipeptides (GP 5)

The respective *N*-MeZ protected cyclohexadepsipeptide (10 µmol) was treated with 10% anisole in TFA (1 mL) in the dark for 2 h. All volatiles were then removed under reduced pressure at 20 °C. The solid residue was taken up with toluene (2 × 10 mL), which was distilled off under reduced pressure to remove the last traces of anisole and TFA. The resulting crude deprotected depsipeptide was directly used for the subsequent coupling reaction.

Preparation of heptadepsipeptides and hormaomycines by HATU/HOAt mediated coupling (GP 6)

The respective deprotected (according to GP 5) depsidipeptide (0.100 mmol) was dissolved in anhydrous CH_2Cl_2 (4 mL), the *N*-protected amino acid (0.320 mmol), HATU (0.300 mmol) and HOAt (0.300 mmol) were added, and the mixture was cooled to 4 °C. DIEA (0.102 mmol) and TMP (0.900 mmol) were then added as a solution in anhydrous CH_2Cl_2 (2 mL), the mixture was allowed to warm to 20 °C, and stirring was continued for an additional 15 h. The mixture was then taken up with EtOAc (100 mL), and after the usual aqueous work-up (GP 2) the organic layer was concentrated to leave the crude depsipeptide, which was purified by recrystallization and/or chromatography.

Removal of the MOM ether group using $MgBr_2$•Et_2O and EtSH (GP 7)

$MgBr_2$•Et_2O (1 mmol) and EtSH (0.5 mmol) were added to a vigorously stirred solution of the respective *O*-MOM protected derivative (0.1 mmol) in CH_2Cl_2 (15 mL), and stirring was continued for an additional 3.5 h (TLC control was impossible as the starting material and the product in all cases showed exactly the same R_f in all tested solvent systems). The mixture was then taken up with EtOAc (40 mL), and the solution was washed with 1 M $KHSO_4$ (3 × 10 mL), water (5 × 10 mL), brine (2 × 5 mL), dried over $MgSO_4$, filtered and

concentrated under reduced pressure. The residue was purified first by crystallization, and the crude product was finally purified by HPLC.

Reduction with $LiAlH_4$ (inverse addition) (GP 8)

A solution of $LiAlH_4$ in diethyl ether (1 M, 6 mL, 6 mmol) was added dropwise to the cooled (dry ice/acetone bath) solution of the respective carbonyl compound (20 mmol for ketones, 10 mmol for esters, 7.5 mmol for carboxylic acids) in diethyl ether (20 mL), and the mixture was stirred at –78 °C for an additional 30 min. The flask was immersed in an ice/water bath, the mixture was stirred for an additional 2 h, and a saturated aqueous NH_4Cl solution was added dropwise under vigorous stirring (careful – foam!) until the H_2 gas evolution ceased. The mixture was stirred for an additional 15 min, filtered with suction through a pad of Celite®, the filter cake was washed with diethyl ether (3 × 50 mL), and the combined filtrates were concentrated under reduced pressure to give the target alcohol.

Conversion of alcohols to iodides (GP 9)

The respective racemic alcohol (12 mmol) was added to a solution of triphenylphosphine (5.5 g, 21 mmol) and imidazole (1.5 g, 22 mmol) in the respective solvent mixture, and the solution was cooled down to –5 °C (internal temperature, ice/salt bath). Solid iodine (6.0 g, 24 mmol) was added in one portion, and the mixture was stirred at this temperature for an additional 30 min, then the bath was removed, and the mixture was stirred at ambient temperature for 3 h. The mixture was poured into pentane (200 mL) under vigorous stirring, and the resulting mixture was washed with 20% (w/w) aqueous $Na_2S_2O_3 \times 5\,H_2O$ (100 mL), the upper pentane layer was separated, the water layer was extracted with pentane (2 × 50 mL) and discarded. The combined pentane solution (washings and extracts) was washed with 20% (w/w) aqueous $Na_2S_2O_3 \times 5\,H_2O$ (100 mL), brine (100 mL), dried over $MgSO_4$, filtered and concentrated under reduced pressure to give the crude product as a colorless liquid containing a solid. This was purified by column chromatography (silica gel, eluting with pentane), to give the pure corresponding target iodide.

*Alkylation of the Ni(II)-complexes of Schiff bases, derived from glycine and (S)- or (R)-2-[(N-benzylprolyl)amino]benzophenone (glycine nickel(II) complexes; (S)- or (R)-**10**) (GP 10)*

A suspension of the respective glycine nickel(II) complex **10** (2.00 g, 4.02 mmol) in a DMF/MeCN mixture (2 + 4 mL) was degassed in two freeze-pump-thaw cycles (dry ice/acetone bath) under stirring, then NaH (60% in mineral oil, 193 mg, 4.8 mmol) was added to the cold mixture, and the system was warmed up to 0 °C under stirring until the color changed from orange to dark-brown. The mixture was frozen, the respective racemic

iodide **11** (4.22 mmol) was added with stirring, the bath was removed, and the mixture was left to warm to 0 °C with stirring. When the ice cover on the flask started to thaw, the flask was immersed in an ice/water bath, and stirring was continued until all the starting material **4** had been consumed (TLC monitoring, chloroform/acetone 7:1, R_f = 0.12). After about 1 h, 60% aqueous acetic acid (2 mL) was added dropwise. After an additional 10 min of stirring, the mixture was poured into vigorously stirred H_2O (100 mL). The resulting suspension was stirred for about 1 h, and the crude product (diastereomeric mixture) was filtered off, the filter cake was washed with H_2O (3 × 10 mL) and dried overnight in a dessicator over P_2O_5 under reduced pressure. The diastereomers were separated by column chromatography (silica gel, eluting with ethyl acetate).

Decomposition of amino acid Belokon'-type complexes to obtain enantiomerically pure amino acids (GP 11)

6 M HCl (50 mL) was added to a refluxing solution of the respective amino acid nickel(II) complex (1 mmol) in methanol (25 mL), the mixture was heated under reflux for an additional 10 min and concentrated under reduced pressure to leave behind a wet hydrochloride salt. The residue was treated with H_2O (100 mL), the precipitated ligand (2-[(*N*-benzylprolyl)amino]benzophenone) as a hydrochloride salt was filtered off, washed with H_2O (3 × 30 mL), dried and collected for recycling. The filtrate was combined with the washings, neutralized to pH = 6.0 with 5% aqueous ammonia, and the solution was extracted with $CHCl_3$ (3 × 30 mL). The aqueous fraction was concentrated to approx. 10 mL and neutralized with 5% aqueous ammonia to pH = 6.5. The amino acid was separated from the nickel salts by elution of the neutralized concentrate through an H^+-form DOWEX ion-exchange resin column (ca. 150 g of resin) with 5–7% aqueous ammonia. The fraction of the eluate that showed red pigmentation on developing with ninhydrin, was collected. This solution was concentrated under reduced pressure at 40–45 °C. The crude amino acid was dissolved in a minimum volume of hot water; the hot turbid solution was filtered and diluted with an equal volume of ethanol. The precipitate, formed after storing at –20 °C for 1 h, was filtered off, washed with cold ethanol (10 mL), and dried *in vacuo* at 40 °C to give the target amino acid.

Experimental procedures

β-Methylphenylalanine (2*S*,3*R*)-**3**

Nickel(II) (S)-complex with (2S,3R)-β-methylphenylalanine [(2*S*,3*R*)-**32**]: Complex (*S*)-**10** (10.0 g, 20 mmol) was alkylated with racemic 1-iodo-1-phenylethane *rac*-**35** (4.9 g, 21 mmol) according to GP 10 using NaH (60% in oil, 1.0 g, 25 mmol) in a DMF/MeCN mixture

(10 + 20 mL), giving the (2*S*,3*R*) diastereomer (3.78 g, 6.3 mmol, 32% based on (*S*)-**10**), the (2*S*,3*S*) diastereomer (3.13 g, 5.2 mmol, 26% based on (*S*)-**10**] and mixed fractions [3.87 g, 6.4 mmol, 32% based on (*S*)-**10**] as well as products from an oxidation of the anion (0.94 g). The mixed fraction was repeatedly separated by column chromatography to give more 0.80 g of (2*S*,3*R*) diastereomer (1.3 mmol) and 1.09 g of (2*S*,3*S*) diastereomer (1.8 mmol). Total yield is 4.58 g [7.6 mmol, 38% based on (*S*)-**10**] of (2*S*,3*R*) diastereomer and 4.22 g (7.0 mmol, 35% based on (*S*)-**10**) of (2*S*,3*S*) diastereomer. Further separation of the mixed fraction was not performed. (2*S*,3*R*)-**32**: $[\alpha]_D^{20}$ =+2190.0 (*c* = 0.2 in $CHCl_3$); ^{1}H NMR (500 MHz, $CDCl_3$): δ = 1,13 (d, *J* = 7.34 Hz, 3 H), 1.34–1.51 (m, 2 H), 1.69–2.01 (m, 2 H), 2.22 (q, *J* = 7.90 Hz, 2 H), 2.71–2.95 (m, 2 H), 3.25 (t, *J* = 8.72 Hz, 1 H), 3.39 (d, *J* = 12.62 Hz, 1 H), 3.49 (d, *J* = 5.49 Hz, 3 H), 4.12 (d, *J* = 3.17 Hz, 1 H), 4.23 (d, *J* = 12.56 Hz, 1 H), 6.62–6.78 (m, 2 H), 6.99–7.07 (m, 1 H), 7.08–7.70 (m, 13 H), 7.98 (d, *J* = 8.28 Hz, 2 H), 8.26 (d, *J* = 8.56 Hz, 1 H); ^{13}C NMR (125.7 MHz, $CDCl_3$): δ = 18.3 (+), 22.9 (–), 30.7 (–), 44.8 (+), 57.2 (–), 63.5 (–), 70.3 (+), 75.5 (+), 120.4 (+), 123.1 (+), 125.9 (–), 127.1 (+), 127.5 (+), 127.9 (+), 128.4 (+), 128.5 (+), 128.6 (+), 128.61 (+), 128.7 (+), 128.8 (+) 129.1 (+), 129.4 (+), 129.6 (+), 131.5 (+), 131.8 (+), 131.9 (+), 131.95 + 132.03 (+), 132.3 (+), 132.8 (–), 133.2 (–), 133.5 (+), 134.3 (–), 141.0 (–), 142.9 (–), 171.0 (–), 177.4 (–), 180.4 (–); MS-ESI (MeOH): 1827.6 (85, 3M+Na]$^+$), 1225.1 (70, 2M+Na]$^+$), 1205.0 (20, 2M+H]$^+$), 624.3 (100, M+Na]$^+$), 602.3 (48, M+H]$^+$).

(2S,3R)-β-Methylphenylalanine [*MeF,* (2*S*,3*R)*-**3**]: (2*S*,3*R*)-**32** (3.78 g, 6.3 mmol) was decomposed, and the amino acid was separated and purified according to GP 11 to give the pure target amino acid (0.67 g, 3.7 mmol, 59%); ^{1}H NMR (300 MHz, D_2O): δ = 1.43 (d, *J* = 7.3 Hz, 3 H), 3.57 (qd, *J* = 7.3 Hz, 5.0 Hz, 1 H), 3.98 (d, *J* = 5.0 Hz, 1 H), 4.72 (bs, 3 H), 7.36–7.53 (m, 5 H); ^{13}C NMR (125.7 MHz, D_2O): δ = 13.81 (+), 39.46 (+), 60.75 (+), 127.81 (+), 127.86 (+), 129.17 (+), 140.38 (–), 173.24 (–); $[\alpha]_D^{20}$ = –7.4 (*c* = 0.5 in H_2O); MS-ESI (MeOH): (positive) *m/z* (%) 180 (100, M+H]$^+$), (negative) *m/z* (%) 178 (100, M–H]$^-$).

Nickel(II) complexes of core-substituted β-methylphenylalanines **32**

Racemic 1-(p-chlorophenyl)ethanol: *p*-Chloroacetophenone (3.10 g, 20 mmol) was reduced with a $LiAlH_4$ solution according to GP 8, giving the target racemic alcohol as a colorless liquid (3.07 g, 19.6 mmol, 98%); ^{1}H NMR (250 MHz, $CDCl_3$): δ = 1.42 (d, *J* = 6.5 Hz, 3 H), 2.65 (bs, 1 H), 5.36 (dq, *J* = 3.0 Hz, 6.5 Hz, 1 H), 7.20–7.35 (m, 4 H), ^{13}C NMR (62.9 MHz, $CDCl_3$): δ = 25.12, 69.50, 126.70, 128.43, 132.85, 144.14.

Racemic 1-iodo-1-(p-chlorophenyl)ethane (*rac*-**35**-*p*-Cl): Racemic 1-(*p*-chlorophenyl)ethanol (3.0 g, 19.2 mmol) was transformed to the respective iodide according to GP 9 using triphenylphosphine (6.7 g, 25.5 mmol), imidazole (2.0 g, 30 mmol) and iodine (9.4 g, 37.0 mmol) in a toluene/acetonitrile mixture (100 + 20 ml). The mixture was heated under reflux for 30 min before work-up, diluted with *tert*-buthyl methyl ether (50 mL), washed with 10% w/w aqueous $NaHSO_3$ (3 × 50 mL), H_2O (50 mL) and brine (50 mL), dried over $MgSO_4$, and concentrated under reduced pressure. The crude iodide was purified by column chromatography (silica gel, eluted with pentane), giving the target iodide *rac*-**35**-*p*-Cl as a yellowish liquid (5.01 g, 18.8 mmol, 98%); TLC: pentane, R_f = 0.28; ^{1}H NMR (250 MHz, $CDCl_3$): δ = 2.19 (d, *J* = 7.1 Hz, 3 H), 5.36 (q, *J* = 7.1 Hz, 1 H), 7.23–7.41 (m, 4H), ^{13}C NMR (62.9 MHz, $CDCl_3$): δ = 24.35, 28.81, 127.85, 128.82, 133.38, 143.88; MS-EI (70 eV): m/z (%) 141 (30, M(^{37}Cl)–I $]^{+}$), 139 (100, M(^{35}Cl)–I $]^{+}$), 103 (50, C_8H_7 $]^{+}$), 77 (18, C_6H_5 $]^{+}$).

Belokon'-type (S)-complex with (2S,3R)-β-methyl(p-chlorophenyl)alanine [(2*S*,3*R*)-**32**-*p*-Cl]: Complex (*S*)-**10** (3.45 g, 6.9 mmol) was alkylated with the racemic 1-iodo-1-(*p*-chlorophenyl)ethane *rac*-**35**-*p*-Cl (1.95 g,7.3 mmol) according to GP 10 using NaH (60% in oil, 0.33 g, 8.3 mmol) in a DMF/MeCN mixture (3.5 + 7.0 mL) giving, after chromatography, the (2*S*,3*R*) component [1.77 g, 2.8 mmol, 40% on (*S*)-**10**, d.e.≥98%], the (2*S*,3*S*) component [1.83 g, 2.9 mmol, 42% on (*S*)-**10**, d.e.≥98%] and mixed fractions [0.67 g, 1.1 mmol, 15.2% on (*S*)-**10**] as well as oxidation products of the anion (0.11 g); (2*S*,3*R*)-component: TLC: R_f = 0.17 (EtOAc); ^{1}H NMR (250 MHz, $CDCl_3$): δ = 1.07 (d, 7.3 Hz), 1.45–1.65 (m, 1 H), 1.80–2.06 (m, 2 H), 2.14–2.36 (m, 3 H), 2.70–2.90 (m, 2 H), 3.29 (t, *J* = 8.6 Hz, 1 H), 3.40 (d, *J*=12.5 Hz, 1 H), 4.10 (d, *J*=3.1 Hz, 1 H), 4.24 (d, *J*=12.5 Hz, 1 H), 6.62–6.74 (m, 2 H), 7.00–7.06 (m, 1 H), 7.08–7.19 (m, 2 H), 7.22–7.37 (m, 5 H), 7.42–7.63 (m, 5 H), 7.94–8.03 (m, 2 H), 8.22–8.30 (m, 1 H); ^{13}C NMR (62.9 MHz, $CDCl_3$): 18.02, 22.69, 30.67, 44.18, 50.38, 57.40, 63.53, 70.18, 75.05, 106.97, 113.70, 120.39, 122.98, 125.66, 126.94, 127.64,

128.52, 128.76, 129.09, 129.62, 130.61, 131.32, 132.32, 133.12, 133.41, 133.51, 134.04, 139.37, 142.69, 171.12, 177.14, 180.32.

(2S,3R)-β-Methyl(p-chlorophenyl)alanine [(2*S*,3*R*)-**3**-*p*-Cl]: (2*S*,3*R*)-**32**-*p*-Cl (1.70 g, 2.7 mmol) was decomposed and the amino acid was separated and purified according to GP 11 to give the pure target amino acid (512 mg, 2.4 mmol, 89%); ^{1}H NMR (300 MHz, D_2O): δ = 1.43 (d, *J* = 7.3 Hz, 3 H), 3.57 (qd, *J* = 7.3 Hz, 5.0 Hz, 1 H), 3.98 (d, *J* = 5.0 Hz, 1 H), 4.72 (bs, 3 H), 7.36–7.53 (m, 5 H); ^{13}C NMR (125.7 MHz, D_2O): δ = 13.81 (+), 39.46 (+), 60.75 (+), 127.81 (+), 127.86 (+), 129.17 (+), 140.38 (–), 173.24 (–); $[\alpha]_D^{20}$ –7.4 (c=0.5 in H_2O); MS-ESI (MeOH): positive 180.0 (100%), negative 178.2 (100%).

Racemic 1-(m-chlorophenyl)ethanol): *m*-Chloroacetophenone (3.10 g, 20 mmol) was reduced with $LiAlH_4$ solution according to GP 8, giving the target racemic alcohol as a colorless liquid (3.09 g, 19.7 mmol, 98%); ^{1}H NMR (250 MHz, $CDCl_3$): δ = 1.44 (d, *J* = 6.4 Hz, 3 H), 2.48 (bs, 1 H), 4.81 (dq, *J*=6.4 Hz, 3.3 Hz, 1 H), 7.16–7.30 (m, 3 H), 7.32–7.36 (m, 1 H); ^{13}C NMR (62.9 MHz, $CDCl_3$): δ = 25.12, 59.59, 123.48, 125.54, 127.41, 129.70, 134.22, 147.77; MS-EI (70 eV): m/z (%) 158 (10%, M(^{37}Cl)⌉$^{+}$), 156 (30%, M(^{35}Cl)⌉$^{+}$), 143 (25%, M(^{37}Cl)–O+H⌉$^{+}$), 141 (100%, M(^{35}Cl)–O+H⌉$^{+}$, M(^{37}Cl)–OH⌉$^{+}$), 139.1 (45%, M(^{35}Cl)–OH⌉$^{+}$), 121.1 (15%, M–Cl⌉$^{+}$), 115.1 (13%, $C_6H_6{}^{37}Cl$⌉$^{+}$), 113.1 (40%, $C_6H_6{}^{35}Cl$⌉$^{+}$), 77.1 (75%, C_6H_5⌉$^{+}$).

Racemic 1-iodo-1-(m-chlorophenyl)ethane (*rac*-**35**-*m*-Cl): Racemic 1-(*m*-chlorophenyl)ethanol (3.0 g, 19.2 mmol) was converted to the respective iodide according to GP 9 using triphenylphosphine (6.7 g, 25.5 mmol), imidazole (2.0 g, 30 mmol) and iodine (9.4 g, 37.0 mmol) in a toluene/acetonitrile mixture (100 + 20 ml). The mixture was heated under reflux for 30 min before work-up, diluted with *tert*-buthyl methyl ether (50 mL), washed with 10% w/w aqueous $NaHSO_3$ (3 × 50 mL), H_2O (50 mL) and brine (50 mL), dried over $MgSO_4$,

and concentrated under reduced pressure. The crude iodide was purified by column chromatography (silica, eluted with pentane), giving the target iodide *rac*-**42**-*m*-Cl as a yellowish liquid (5.04 g, 18.9 mmol, 98%); TLC: $R_f = 0.28$, pentane; ^{1}H NMR (250 MHz, $CDCl_3$): δ = 2.19 (d, *J* = 7.1 Hz, 3 H), 5.31 (q, *J* = 7.1 Hz, 1 H), 7.20–7.35 (m, 3 H), 7.41–7.44 (m, 1 H); ^{13}C NMR (62.9 MHz, $CDCl_3$): δ = 23.72, 28,67, 124.80, 126.67, 128.01, 129.92, 134.28, 147.21.

Belokon'-type (S)-complex with (2S,3R)-β-methyl(m-chlorophenyl)alanine [(2*S*,3*R*)-**32**-*m*-Cl]: Complex (*S*)-**10** (3.45 g, 6.9 mmol) was alkylated with the racemic 1-iodo-1-(*m*-chlorophenyl)ethane *rac*-**35**-*m*-Cl (1.95 g,7.3 mmol) according to GP 10 using NaH (60% in oil, 0.33 g, 8.3 mmol) in a DMF/MeCN mixture (3.5 + 7.0 mL), giving the (2*S*,3*R*) component [1.83 g, 2.9 mmol, 42% on (*S*)-**10**, d.e.≥98%], the (2*S*,3*S*)-component (1.62 g, 2.5 mmol, 37% on (*S*)-**10**, d.e.≥98%) and mixed fractions (0.87 g, 1.4 mmol, 19.8% on (*S*)-**10**) as well as oxidation products of the anion (0.18 g). (2*S*,3*R*)-Component: ^{1}H NMR (250 MHz, $CDCl_3$): δ = 1.11 (d, *J* = 7.3 Hz, 3 H), 1.43–1.64 (m, 1 H), 1.73–2.02 (m, 2 H), 2.27 (q, *J* = 7.6 Hz, 2 H), 2.75–2.94 (m, 2 H), 3.27 (t, *J* = 8.6 Hz, 1 H), 3.42 (d, *J* = 12.6 Hz, 1 H), 4.05–4.16 (m, 1 H), 4.25 (d, *J* = 12.6 Hz, 1 H), 6.62–6.74 (m, 2 H), 6.98–7.06 (m, 1 H), 7.08–7.19 (m, 2 H), 7.19–7.35 (m, 5 H), 7.37–7.46 (m, 3 H), 7.46–7.66 (m, 3 H), 7.93–8.00 (m, 2 H), 8.26–8.32 (m, 1 H); ^{13}C NMR (62.9 MHz, $CDCl_3$): 30.84, 44.62, 57.25, 63.45, 70.30, 75.18, 120.46, 123.17, 125.81, 127.86, 128.68, 128.91, 129.25, 131.52, 143.05, 143.34, 177.12, 180.39, 209,51.

(2S,3R)-β-Methyl(m-chlorophenyl)alanine [(2*S*,3*R*)-**3**-*m*-Cl]: Complex (2*S*,3*R*)-**32**-*m*-Cl (1.80 g, 2.8 mmol) was decomposed, and the amino acid was separated and purified according to GP 11 to give the pure target amino acid (573 mg, 2,7 mmol, 96%); ^{1}H NMR (300 MHz, D_2O): δ = 1.43 (d, *J* = 7.3 Hz, 3 H), 3.57 (qd, *J* = 7.3 Hz, 5.0 Hz, 1 H), 3.98 (d, *J* = 5.0 Hz, 1 H), 4.72 (bs, 3 H), 7.36–7.53 (m, 5 H); ^{13}C NMR (125.7 MHz, D_2O):

δ = 13.81 (+), 39.46 (+), 60.75 (+), 127.81 (+), 127.86 (+), 129.17 (+), 140.38 (–), 173.24 (–); $[\alpha]_D^{20}$ –7.4 (c=0.5 in H_2O); MS-ESI (MeOH): positive 180.0 (100%), negative 178.2 (100%).

Racemic 1-(o-chlorophenyl)ethanol: *o*-Chloroacetophenone (3.10 g, 20 mmol) was reduced with $LiAlH_4$ solution according to GP 8, giving the target racemic alcohol as a colorless liquid (2.98 g, 19.0 mmol, 95%); ^{1}H NMR (250 MHz, $CDCl_3$): δ = 1.46 (d, *J* = 6.3 Hz, 3 H), 2.50 (bs, 1 H), 5.26 (q, *J* = 6.3 Hz, 1 H), 7.12–7.34 (m, 3 H), 7.53–7.60 (m, 1 H); ^{13}C NMR (62.9 MHz, $CDCl_3$): δ = 23.43, 66.81, 126.33, 127.12, 128.29, 129.28, 131.48, 142.99; MS-EI (70 eV): m/z (%) 158.2 (4%, M(^{37}Cl)$\rceil^{+}$), 156.2 (12%, M(^{35}Cl)$\rceil^{+}$), 143.1 (18%, M(^{37}Cl)–O+H$\rceil^{+}$), 141.1 (55%, M(^{35}Cl)–O+H$\rceil^{+}$), 115.1 (10%, $C_6H_6{}^{37}Cl\rceil^{+}$), 113.1 (30%, $C_6H_6{}^{35}Cl\rceil^{+}$), 77.1 (100%, $C_6H_5\rceil^{+}$).

Racemic 1-iodo-1-(o-chlorophenyl)ethane (*rac*-**35**-*o*-Cl): Racemic 1-(*o*-chlorophenyl)ethanol (2.9 g, 18.5 mmol) was converted to the respective iodide according to GP 7 using triphenylphosphine (6.7 g, 25.5 mmol), imidazole (2.0 g, 30.0 mmol) and iodine (9.4 g, 37.0 mmol) in a toluene/acetonitrile mixture (100 + 20 ml). The mixture was heated under reflux for 30 min before work-up, diluted with *tert*-buthyl methyl ether (50 mL), washed with 10% w/w aqueous $NaHSO_3$ (3 × 50 mL), H_2O (50 mL) and brine (50 mL), dried over $MgSO_4$, and concentrated under reduced pressure. The crude iodide was purified by column chromatography (silica gel, eluted with pentane), to give the target iodide *rac*-**35**-*o*-Cl as a yellowish liquid (4.82 g, 18.1 mmol, 98%); TLC: pentane, R_f = 0.28; ^{1}H NMR (250 MHz, $CDCl_3$): δ = 2.25 (d, *J* = 7.1 Hz, 3 H), 5.72 (q, *J* = 7.1 Hz, 1 H), 7.15–7.35 (m, 3 H), 7.60–7.67 (m, 1 H); ^{13}C NMR (62.9 MHz, $CDCl_3$): δ = 20.54, 27.75, 127.50, 127.57, 128.93, 129.89, 132.05, 142.11; MS-EI (70 eV): m/z (%) 141.1 (33%, M(^{37}Cl)–I$\rceil^{+}$), 139.1 (100%, M(^{35}Cl)–I$\rceil^{+}$), 103.1 (100%, M–(H+Cl+I)$\rceil^{+}$), 77.1 (75%, $C_6H_5\rceil^{+}$).

Belokon'-type (S)-complex with (2S,3R)-β-methyl(o-chlorophenyl)alanine [(2*S*,3*R*)-**32**-*o*-Cl]: Complex (*S*)-**10** (3.08 g, 6.2 mmol) was alkylated with racemic 1-iodo-1-(*o*-chlorophenyl)ethane *rac*-**35**-*o*-Cl (1.74 g, 6.5 mmol) according to GP 10 using NaH (60% in oil, 0.30 g, 7.4 mmol) in a DMF/MeCN mixture (3.0 + 6.0 mL), giving the (2*S*,3*R*) component [1.64 g, 2.6 mmol, 42% on (*S*)-**10**, d.e.≥98%], the (2*S*,3*S*) component [1.49 g, 2.3 mmol, 38% on (*S*)-**10**, d.e.≥98%] and mixed fractions [0.76 g, 1.2 mmol, 19.2% on (*S*)-**10**] as well as oxidation products of the anion (0.12 g).

Racemic 1-(p-fluorophenyl)ethanol): *p*-Fluoroacetophenone (2.76 g, 20.0 mmol) was reduced with $LiAlH_4$ solution according to GP 8, giving the target racemic alcohol as a colorless liquid (2.72 g, 19.4 mmol, 97%); TLC: R_f = 0.27, pentane; ^{1}H NMR (250 MHz, $CDCl_3$): δ = 1.45 (d, *J* = 6.5 Hz, 3 H), 2.30 (bs, 1 H), 4.85 (q, *J* = 6.5 Hz, 1 H), 6.96–7.08 (m, 2 H), 7.25–7.37 (m, 2 H); ^{13}C NMR (62.9 MHz, $CDCl_3$): δ = 25.20 (+), 69.65 (+), 115.16 (+, d, *J* = 21.3 Hz), 126.98 (+, d, *J* = 8.1 Hz), 141.45 (C_{quat}, d, *J* = 3.1 Hz), 162.00 (C_{quat}, d, *J* = 245.1 Hz); MS-EI (70 eV): m/z (%) 140.1 (22%, M^{+}), 125.1 (100%, $C_8H_{10}F^{+}$), 97.1 (60%, $C_6H_6F^{+}$), 77.1 (20%, $C_6H_5^{+}$), 43.2 (24%, $C_2H_3O^{+}$).

Racemic 1-iodo-1-(p-fluorophenyl)ethane (*rac*-**35**-*p*-F): Racemic 1-(*p*-fluorophenyl)ethanol (2.5 g, 17.8 mmol) was converted to the respective iodide according to GP 9 using triphenylphosphine (8.1 g, 30.7 mmol), imidazole (2.2 g, 32.6 mmol) and iodine (9.0 g, 35.6 mmol) in a diethyl ether/acetonitrile mixture (50 + 35 ml), giving the target iodide *rac*-**35**-*p*-F as a yellowish liquid (4.31 g, 17.2 mmol, 96,8%); TLC: R_f = 0.37 pentane; ^{1}H NMR (250 MHz, $CDCl_3$): δ = 2.20 (d, *J* = 7.1, 3 H), 5.40 (q, *J* = 7.1 Hz, 1 H), 6.92–7.06 (m, 2 H), 7.36–7.48 (m, 2 H); ^{13}C NMR (62.9 MHz, $CDCl_3$): δ = 24.85 (+), 29.13 (+), 115.54 (+, d, *J* = 21.6 Hz), 128.20 (+, d, *J* = 8.3 Hz), 141.24 (C_{quat}), 161.86 (C_{quat}, d, *J* = 247.2 Hz).

Belokon'-type (S)-complex with (2S,3R)-β-methyl(p-fluorophenyl)alanine [(2*S*,3*R*)-**32**-*p*-F]: Complex (*S*)-**10** (2.84 g, 5.7 mmol) was alkylated with the racemic 1-iodo-1-(*p*-fluorophenyl)ethane *rac*-**35**-*p*-F (1.50 g, 6.0 mmol) according to GP 10 using NaH (60% in oil, 0.28 g, 6.8 mmol) in a DMF/MeCN mixture (3.0 + 6.0 mL), giving the (2*S*,3*R*) component [1.53 g, 2.5 mmol, 43% on (*S*)-**10**, d.e.≥98%], the (2*S*,3*S*) component [1.63 g, 2.6 mmol, 46% on (*S*)-**10**, d.e.≥98%] and mixed fractions [0.33 g, 0.52 mmol, 9.2% on (*S*)-**10**] as well as oxidation products of the anion (0.13 g) after chromatography (silica gel, eluted with ethyl acetate). ^{1}H NMR (250 MHz, $CDCl_3$): δ = 1.85–2.02 (m, 1 H), 2.03–2.21 (m, 1 H), 2.06 (d, *J* = 7.1 Hz, 3 H) 2.50–2.75 (m, 1 H), 2.80–2.98 (m, 1 H), 3.31–3.52 (m, 4 H), 3.59 (d, *J* = 12.7 Hz, 1 H), 4.09 (d, *J* = 5.0 Hz, 1 H), 4.39 (d, *J* = 2.6 Hz, 1 H), 6.50–6.71 (m, 5 H), 6.75 (t, *J* = 8.6 Hz, 2 H), 7.01–7.17 (m, 2 H), 7.22–7.34 (m, 4 H), 7.47–7.58 (m, 2 H), 8.04 (d, *J* = 7.3 Hz, 2 H), 8.20 (d, *J* = 8.6 Hz, 1 H); ^{13}C NMR (62.9 MHz, $CDCl_3$): δ = 16.12, 23.27, 30.71, 43.94, 46.16, 56.71, 63.19, 70.39, 75.66, 114.83 (d, *J* = 21.0 Hz), 120.63, 123.13, 126.31, 127.46, 128.20, 128.75, 128.88, 128.94, 129.09 (d, *J* = 7.9 Hz), 129.58, 131.43, 133.04, 133.52, 133.75, 134.48 (d, *J* = 269.7 Hz), 142.39, 170.75, 176.73, 180.14.

(*R*)-*allo*-Threonine (2*R*,3*R*)-**4**

(R)-Allo-threonine nickel(II) complex (*R,R,R*-**28**): Complex (*R*)-**10** (21.4 g, 43.0 mmol) was suspended in DMF/MeCN mixture (20 + 40 mL) and degassed with two freeze-pump-thaw cycles (dry ice/acetone bath) under stirring, then NaH (60% in oil, 2.0 g, 50 mmol) was added to the cold mixture, and the system was warmed up to 0 °C under stirring until the H_2 gas evolution ceased. The mixture was frozen, acetaldehyde (2.0 g, 45 mmol) was added under stirring, and the system was left to warm to 0 °C under air with stirring. When the ice cover on the flask started to thaw, 60% aqueous acetic acid (4.5 mL) was added. After an additional 10 min of stirring the mixture was concentrated under reduced pressure (bath temp ~50 °C), and the liquid residue was poured into a vigorously stirred mixture of H_2O (0.5 L) and $CHCl_3$ (100 mL). The organic layer was separated and washed with H_2O (2 × 100 mL), the combined

water phases were extracted with $CHCl_3$ (3 × 50 mL), the combined organic phases were concentrated under reduced pressure to ~20 mL volume and left to crystallize at ambient temperature overnight. The crystals were filtered off, washed with cold $CHCl_3$ (~10 mL) and recrystallized from $CHCl_3$, giving orange crystals, uniform by TLC (1.76 g, 3.2 mmol, 7.5%); ^{1}H NMR (250 MHz, $CDCl_3$): δ = 1.08 (d, *J* = 6.5 Hz, 3 H), 1.94–2.12 (m, 3 H), 2.25–2.59 (m, 2 H), 2.67–2.84 (m, 1 H), 3.38–3.61 (m, 1 H), 3.45 (dd, *J* = 11.1 Hz, 5.8 Hz, 1 H), 3.55 (d, 12.6 Hz, 1 H), 3.75–3.97 (m, 3 H), 4.42 (d, *J* = 12.6 Hz, 1 H), 6.65 (d, *J* = 3.87 Hz, 2 H), 6.95 (m, 1 H), 7.09–7.23 (m, 2 H), 7.35 (t, *J* = 7.52 Hz, 2 H), 7.41–7.56 (m, 3 H), 8.06 (d, *J* = 7.02 Hz, 2 H), 8.14 (d, *J* = 8.61 Hz, 1 H); ^{1}H NMR (250 MHz, [D$_6$]-DMSO): δ = 0.88 (d, *J* = 6.3 Hz, 3 H), 1.81–2.18 (m, 2 H), 2.29–2.40 (m, 2 H), 3.45–3.58 (m, 3 H), 3.61–3.87 (m, 2 H), 4.04 (d, *J* = 12.2 Hz, 1 H), 6.12 (d, *J* = 5.1 Hz, 1 H), 6.54 (d, *J* = 7.8 Hz, 1 H), 6.64 (t, *J* = 7.1 Hz, 1 H), 7.02–7.19 (m, 3 H), 7.30–7.62 (m, 6 H), 7.99 (d, *J* = 8.6 Hz, 1 H), 8.35 (d, *J* = 7.4 Hz, 2 H), hydroxyl signal is masked with H_2O absorption; ^{13}C NMR (62.9 MHz, [D$_6$]-DMSO): δ = 20.19, 22.61, 30.24, 57.43, 62.38, 69.27, 69.71, 75.82, 119.78, 123.20, 126.05, 127.24, 127.75, 128.20, 128.31, 128.46, 128.86, 128.96, 129.38, 130.96, 131.45, 131.55, 132.48, 134.01, 134.76, 142.61, 169.08, 174.67, 180.18.

*(2R,3R)-2-Amino-3-hydroxybutyric acid [R-allo-threonine, (2R,3R)-***4***]*: (*R*)-*allo*-Threonine nickel(II) complex *R*,*R*,*R*-**28** (1.76 g, 3.2 mmol) was decomposed, and the amino acid was separated according to GP 11 to give the target amino acid (351 mg, 2.9 mmol, 91%).

(Trifluoromethylcyclopropyl)alanine **9a**

Racemic ethyl 5,5,5-trifluoro-4-oxyvalerate (*rac*-**21**): To a solution of ethyl 5,5,5-trifluoro-4-oxovalerate (**20)** (15.8 g, 79.7 mmol) in anhydrous diethyl ether (160 mL), cooled in an ice/salt bath, was added powdered sodium borohydride (1.51 g, 40.0 mmol) in one portion. The cold mixture was stirred for 10 min, the bath was removed, and the mixture was stirred for 4 h. 1 M aq. $KHSO_4$ (50 mL) was added slowly (careful – foam!), the organic layer was separated, and the water layer was extracted with diethyl ether (3 × 30 mL). The combined

organic layers were dried over $MgSO_4$, filtered and concentrated under reduced pressure to give the target hydroxyester *rac*-**21** (15.6 g, 98%) as a yellowish liquid. ^{1}H NMR (250 MHz, $CDCl_3$): δ = 1,25 (t, *J* = 7.1 Hz, 3 H), 1.80–2.12 (m, 2 H), 2.54 (t, *J* = 7.1 Hz, 2 H), 3.38–3.76 (bs, 1 H), 3.91–4.07 (m, 1 H), 4.14 (q, *J* = 7.1 Hz, 2 H); ^{13}C NMR (62.9 MHz, $CDCl_3$): δ = 13.9 (+), 24.5 (–), 29.6 (–), 61.0 (–), 69.5 (+, q, *J* = 31.2 Hz), 124.90 (–, q, *J* = 281.9 Hz), 173.9 (–); IR (film): 3460, 2989, 1724, 1373, 1278, 1131, 1021, 880 cm^{-1}.

Racemic trans-2-trifluoromethylcyclopropanecarboxylic acid (*rac*-**24**): A solution of ethyl 5,5,5-trifluoro-4-tosyloxyvalerate **(22)** (6.4 g, 18 mmol) in anhydrous THF (10 mL) was added to a vigorously stirred solution of potassium *tert*-butoxide (9.0 g, 80 mmol) in anhydrous THF (50 mL) under an N_2 flow at 65 °C during 3 h using a syringe pump, and the solution was heated under reflux for 4 h. After cooling, the resulting mixture was diluted with water (100 mL), and the organics were distilled off under reduced pressure. The alkaline water phase was washed with diethyl ether (3 × 30 mL), acidified with aqueous 6 M HCl to pH~1 and extracted with diethyl ether (5 × 50 mL). The combined extracts were dried over $MgSO_4$, filtered and concentrated under reduced pressure, to give the crude product as a black tar. Kugelrohr distillation of this crude product gave the target acid *rac*-**24** as a colorless liquid (1.3 g, 47%). ^{1}H NMR (250 MHz, $CDCl_3$): δ = 1.25–1.48 (m, 2 H), 1.97–2.11 (m, 1 H), 2.12–2.29 (m, 1 H), 10.36 (bs, 1 H); ^{13}C NMR (62.9 MHz, $CDCl_3$): δ = 10.9 (–, q, *J* = 3.2 Hz), 16.7 (+, q, *J* = 2.6 Hz), 22.6 (+, q, *J* = 38.3 Hz), 124.5 (–, q, *J* = 271.2 Hz), 177.8.

Racemic (2-trifluoromethylcyclopropyl)methanol (*rac*-**25a**): Racemic *trans*-2-trifluoromethyl-cyclopropanecarboxylic acid *rac*-**24** (3.4 g, 18.6 mmol) was reduced with lithium aluminum hydride in diethyl ether (1.13 M, 8.3 mL, 9.4 mmol) according to GP 8 to give the target alcohol *rac*-**25a** as a colorless liquid (2.3 g, 88%). ^{1}H NMR (250 MHz, $CDCl_3$): 0.70–0.83 (m, 1 H), 0.94–1.06 (m, 1 H), 1.39–1.58 (m, 2 H), 1.95 (bd, *J* = 19.7 Hz, 1 H), 3.40–

3.70 (m, 2 H); ^{13}C NMR (62.9 MHz, $CDCl_3$): 6.3 (–), 17.4 (+), 17.9 (+, q, *J*=37.0 Hz), 63.6 (–), 126.1 (+, q, *J*=270.3 Hz).

Racemic trans-(2-trifluoromethylcyclopropyl)methyl iodide (**11a**): Racemic *trans*-(2-trifluoromethylcyclopropyl)methanol (**25a)** (1.7 g, 12 mmol) was treated according to GP 9 with triphenylphosphine (5.5 g, 21 mmol), imidazole (1.5 g, 22 mmol) and solid iodine (6.0 g, 24 mmol) in a diethyl ether/acetonitrile mixture (36 + 24 mL), giving the target iodide as a slightly yellowish liquid (2.7 g, 90%). TLC: R_f = 0.36, pentane; ^{1}H NMR (250 MHz, $CDCl_3$): 0.75–0.87 (m, 1 H), 1.21–1.33 (m, 1 H), 1.41–1.57 (m, 1 H), 1.63–1.79 (m, 1 H), 3.03–3.21 (m, 2 H); ^{13}C NMR (62.9 MHz, $CDCl_3$): 6.8, 13.5 (q, *J*=3.0 Hz), 19.4 (q, *J*=2.7 Hz), 25.1, 130.0 (q, *J*=280.5 Hz). Product contains ~25% w/w of pentane.

Nickel (II) (R)-complex of 3-(2-trifluoromethylcyclopropyl)alanine [*(R)-B(tFmcpA)C,* (2*R*,1'*S*,2'*R*)-**26a**]: (*R*)-**10** (3.17 g, 6.4 mmol) was alkylated with racemic *trans*-(2-trifluoromethylcyclopropyl)methyl iodide (**11a)** (1.67 g, 6.7 mmol) according to GP 10 using NaH (60% in oil, 305 mg, 7.6 mmol) in a DMF/MeCN mixture (3 + 6 mL) during 4 h to give, after chromatographic separation (silica gel, eluting with EtOAc), the (2*R*,1'*S*,2'*R*) diastereomer [1.73 g, 44% based on (*R*)-**10**, d.e.≥98%], and the (2*R*,1'*R*,2'*S*) diastereomer [1.68 g, 42% based on (*R*)-**10**, d.e.≥98%] and mixed fractions [0.17 g, 4% based on (*R*)-**10**] as well as oxidation products of the anion (0.12 g). For the (2*R*,1'*S*,2'*R*) diastereomer: ^{1}H NMR (250 MHz, $CDCl_3$): δ = –0.09 to –0.15 (m, 1 H), 0.80–0.90 (m, 1 H), 0.86–0.98 (m, 1 H), 1.10–1.21 (m, 1 H), 1.76–1.84 (m, 1 H), 2.04–2.21 (m, 2 H), 2.43–2.52 (m, 1 H), 2.57–2.71 (m, 2 H), 3.46–3.68 (m, 4 H), 3.92 (dd, *J* = 3.5 Hz, 9.1 Hz, 1 H), 4.42 (d, *J* = 12.7 Hz, 1 H), 6.56–6.65 (m, 2 H), 6.86 (d, *J* = 7.6 Hz, 1 H), 7.04–7.23 (m, 2 H), 7.25–7.41 (m, 3 H), 7.41–7.54 (m, 3 H), 8.05 (d, *J* = 7.1 Hz, 2 H), 8.09 (d, *J* = 8.7 Hz, 1 H); ^{13}C NMR (125.7 MHz, $CDCl_3$): δ=8.4, 11.7, 11.8, 20.1 (q, *J* = 36.9 Hz,), 23.9, 30.7, 38.4, 57.2, 63.2, 69.5, 70.1, 120.7, 123.7, 125.9 (q, *J* = 270.3 Hz,), 126.2, 127.3, 127.4, 128.8, 128.89, 128.90,

129.1, 129.2, 129.8, 131.5, 132.3, 133.1, 133.3, 133.7, 142.4, 170.8, 178.7, 180.5; MS-ESI: (positive) *m/z* (%) 1881.6 (100, 3M+H+Na]$^+$), 1263.1 (66, 2M+2H+Na]$^+$), 809.8 (25, 2M+Na–$C_{13}H_{12}$]$^+$), 642.2 (15, M+Na]$^+$), 620.2 (3, M+H]$^+$), (negative) *m/z* (%) 618.2 (100, M–H]$^-$), 528.1 (40, M–C_7H_7]$^-$).

*(2R,1'S,2'R)-3-(2-Trifluoromethylcyclopropyl)alanine [(R)tFmcpA, (2R,1'S,2'R)-***9a***]*: The complex (2*R*,1'*S*,2'*R*)-**26a** (980 mg, 1.58 mmol) was decomposed and the amino acid was purified according to GP 11 to give the pure target amino acid *(2R,1'S,2'R)*-**9a** (275 mg, 1.39 mmol, 88%): $[\alpha]_D^{20}$ = –20.0 (*c* = 0.2 in MeOH); ^{1}H NMR (250 MHz, CD_3OD): δ = 0.75–0.87 (m, 1 H), 0.94–1.07 (m, 1 H), 1.31–1.44 (m, 1 H), 1.47–1.67 (m, 2 H), 2.06–2.22 (m, 1 H), 3.54–3.63 (m, 1 H), 4.94 (bs, 3 H); ^{13}C NMR (62.9 MHz, CD_3OD): δ = 9.64, 13.10, 20.12 (q, *J* = 36.9 Hz), 34.88, 55.99, 127.80 (q, *J* = 269.6 Hz), 173.72; MS-EI (70 eV): *m/z* (%) 152 (100, M–CO_2H]$^+$), 74 (75, $C_2H_4NO_2$]$^+$)); MS-ESI: (positive) *m/z* (%) 198 (100, M+H]$^+$), (negative) *m/z* (%) 196 (60, M–H]$^-$).

Nickel(II) (S)-complex of 3-(2-trifluoromethylcyclopropyl)alanine [*(S)-B(tFmcpA)C,* (2*S*,1'*S*,2'*R*)-**26a**]: (*S*)-**10** (645 mg, 1.29 mmol) was alkylated with racemic (2-trifluoromethyl-cyclopropyl)methyl iodide **11a** (340 mg, 1.36 mmol) according to GP 10 using NaH (60% in oil, 62 mg, 1.55 mmol) in a DMF/MeCN mixture (1 + 2 mL) during 4 h to give, after chromatographic separation (silica gel, eluting with EtOAc), the (2*S*,1'*R*,2'*S*) diastereomer [393 mg, 49% based on (*S*)-**10**, d.e.≥98%], the (2*S*,1'*S*,2'*R*) diastereomer [364 mg, 46% based on (*S*)-**10**, d.e.≥98%] and mixed fractions [33 mg, 4% based on (*S*)-**10**]. For the (2*S*,1'*S*,2'*R*) diastereomer: ^{1}H NMR (250 MHz, $CDCl_3$): δ = 0.43–0.61 (m, 2 H), 1.00–1.13 (m, 1 H), 1.40–1.70 (m, 2 H), 1.99–2.26 (m, 3 H), 2.40–2.78 (m, 2 H), 3.33–3.65 (m, 4 H), 3.99 (dd, *J* = 8.9 Hz, 3.3 Hz, 1 H), 4.46 (d, *J* = 12.6 Hz, 1 H), 6.58–6.71 (m, 2 H), 6.84(d, *J* = 7.5 Hz, 1 H), 7.09–7.23 (m, 2 H), 7.29–7.40 (m, 3 H), 7.41–7.63 (m, 3 H), 8.04–8.12 (m, 3 H); ^{13}C NMR (62.9 MHz, $CDCl_3$): δ = 23.89 (q, *J* = 1.3 Hz), 30.67, 38.77, 57.24, 60.33, 63.21,

69.68, 70.14, 120.74, 125.58 (q, J = 237.9 Hz), 126.24, 127.28, 128.35, 128.54, 128.86, 128.89, 129.25, 129.90, 131.45, 131.93, 132.24, 133.34, 142.27, 170.47, 178.68, 180.43. The cyclopropyl CH_2 signals are close to coalescence and are not observable.[6]

*(2S,1'S,2'R)-3-(2-Trifluoromethylcyclopropyl)alanine [(S)tFmcpA, (2S,1'S,2'R)-***9a***]*: The complex (2*S*,1'*S*,2'*R*)-**26a** (210 mg, 339 µmol) was decomposed and the amino acid was purified according to GP 11 to give the pure target amino acid *(2S,1'S,2'R)*-**9a** (61 mg, 92%).

(Difluoromethylcyclopropyl)alanine (**9b**)

Racemic trans-2-(methoxycarbonyl)cyclopropanecarboxylic acid (**13**) [7]: A solution of LiOH•H_2O (4.2 g, 100 mmol) in methanol (100 mL) was added dropwise within 1 h to a vigorously stirred solution of racemic dimethyl *trans*-cyclopropane-1,2-dicarboxylate (**12**) (15.8 g, 100 mmol) in THF (400 mL) under an N_2 flow, and the resulting mixture was stirred for an additional 1 h. The solvents were evaporated under reduced pressure at ambient temperature, the residue was diluted with water (80 mL), and the solution was washed with diethyl ether. The organic phases were discarded, the aqueous phase was acidified with concentrated aqueous HCl (37%, 10 mL), the solution was saturated with solid NaCl and extracted with diethyl ether (3 × 50 mL). The combined organic phases were dried over $MgSO_4$, filtered and concentrated under reduced pressure to give a clear oil, which solidified when dried under reduced pressure overnight (12.1 g, 84%). ^{1}H NMR (250 MHz, $CDCl_3$): δ = 1.42–1.57 (m, 2 H), 2.11–2.27 (m, 2 H), 3.71 (s, 3 H), 9.76 (bs, 1 H); ^{13}C NMR (62.9 MHz, $CDCl_3$): δ = 15.87, 22.07, 22.75, 52.31, 171.86, 178.13.

Racemic methyl trans-2-(hydroxymethyl)cyclopropane carboxylate (**15**) [8]: Borane-dimethyl sulfide complex (10 M in Me_2S, 10.8 mL, 108 mmol) was added dropwise within 30 min to a cold (ice/water bath) solution of the racemic *trans*-2-(methoxycarbonyl)-cyclopropanecarboxylic acid (**13)** (12.9 g, 90 mmol) in THF (40 mL), and the resulting

mixture was left with stirring in the melting ice/water bath overnight. The mixture was re-cooled (ice/water bath), and methanol (5 mL) was added dropwise with stirring. After the H_2 gas evolution had ceased, the mixture was diluted with methanol (100 mL) and concentrated under reduced pressure, this dilution-concentration process was repeated three times to give the crude product as a clear oil (11.9 g), which was purified by chromatography (silica gel, eluting with Et_2O) to give the pure hydroxyester **15** as a colorless clear oil (11.1 g, 85 mmol, 95%). TLC: R_f=0.26 (Et_2O); ^{1}H NMR (250 MHz, $CDCl_3$): 1.06–1.29 (m, 2 H), 1.36–1.50 (m, 2 H), 1.99–2.25 (m, 2 H), 3.65 (s, 3 H), 4.58 (bs, 1 H); ^{13}C NMR (62.9 MHz, $CDCl_3$): 12.7, 18.1, 24.2, 51.8, 64.3, 174.5.

Racemic methyl trans-2-formylcyclopropane carboxylate (**16**): To a vigorously stirred solution of oxalyl chloride (5.23 g, 3.5 mL, 41.2 mmol) in anhydrous CH_2Cl_2 (70 mL) cooled to –78 °C (dry ice/acetone bath) under nitrogen flow, was added a solution of anhydrous DMSO (6.89 g, 6.3 mL, 88.2 mmol) in anhydrous CH_2Cl_2 (3 mL) at such a rate that the temperature of the reaction mixture did not exceed –70 °C (about 40 min). After the mixture had been stirred at –78 °C for an additional 30 min, a solution of the racemic methyl *trans*-2-(hydroxymethyl)cyclopropanecarboxylate **(15)** (4.44 g, 34.1 mmol) was added dropwise under vigorous stirring, keeping the temperature of the reaction mixture under –70 °C. The mixture was stirred at this temperature for an additional 1 h, and anhydrous triethylamine (17.2 g, 24 mL, 170 mmol) was gradually added at –78 °C. After the addition was complete, the cooling bath was removed, and the stirred mixture was allowed to reach room temperature. Then water (20 mL) was added, and the mixture was acidified with aq. 12 M HCl (15 mL) at 0 °C (ice/salt bath). The organic layer was separated, and the aqueous phase was extracted with diethyl ether (3 × 20 mL). The combined organic layers were washed with water (20 mL), brine (2 × 20 mL), dried over $MgSO_4$, filtered and concentrated under reduced pressure to give the target aldehyde as a colorless clear oil (4.19 g, 96%). ^{1}H NMR (250 MHz, $CDCl_3$): 1.38-1.66 (m, 2H), 2.08-2.53 (m, 2H), 3.68 (s, 3H), 9.27 (d, 4.2 Hz, 1H); ^{13}C NMR

(62.9 MHz, $CDCl_3$): 14.8, 21.9, 30.5, 52.2, 171.5, 198.1. Other signals correspond to the traces of solvents (DCM and pentane).

Racemic methyl trans-2-(difluoromethyl)cyclopropane carboxylate (*rac*-**14b**): The reaction was carried out in a PTFE flask. A Deoxo-Fluor® solution in toluene (50% w/w, 26.4 g, 59.7 mmol) was added with stirring under an N_2 flow to a solution of racemic methyl *trans*-2-formylcyclopropane carboxylate (**16**) (4.5 g, 35.1 mmol) in anhydrous CH_2Cl_2 (6 mL). Ethanol (0.1 mL) was added, and the mixture was stirred at ambient temperature for 48 h. The resulting solution was poured into a vigorously stirred sat. aq. $NaHCO_3$ solution (150 mL), stirred until the CO_2 gas evolution had ceased, the organic phase was separated, the aqueous phase was extracted with CH_2Cl_2 (3 × 50 mL); the combined organic phases were dried over $MgSO_4$, filtered and concentrated under reduced pressure. The crude product was purified by column chromatography (silica gel, eluting with pentane/diethyl ether 4:1) to give the target *rac*-**14b** as a colorless liquid (2.7 g, 51%). ^{1}H NMR (250 MHz, $CDCl_3$): δ = 1.09–1.21 (m, 1 H), 1.21–1.33 (m, 1 H), 1.82–2.00 (m, 2 H), 3.69 (s, 3 H), 5.76 (td, *J* = 57.3 Hz, 3.4 Hz, 1 H); ^{13}C NMR (62.9 MHz, $CDCl_3$): δ = 9.7 (–, t, *J*=4.3 Hz), 15.5 (+, t, *J*=4.4 Hz), 22.8 (+, t, *J*=27.0 Hz), 51.9 (+), 114.6 (+, t, *J*=239.0 Hz), 172.5 (–); IR (film): 2959, 1735, 1441, 1382, 1322, 1293, 1213, 1152, 1069, 996 cm^{-1}; MS-EI (70eV): *m/z* (%) 150.1 (10, M^{+}), 149.1 (9, $M–H^{+}$), 119.1 (100, $M–OMe^{+}$), 99.1 (29, $C_5H_4FO^{+}$), 91.1 (30, $M–CO_2Me^{+}$), 59.0 (28, $C_3H_4F^{+}$).

Racemic trans-(2-difluoromethylcyclopropyl)methanol (*rac*-**25b**): Racemic methyl *trans*-2-(difluoromethyl)cyclopropane carboxylate **14b** (2.52 g, 16.8 mmol) was reduced with lithium aluminum hydride in diethyl ether (1.13 M, 7.4 mL, 8.4 mmol) according to GP 8. The crude product was purified by column chromatography (silica gel, eluting with pentane/diethyl ether 1:1) to give the target *rac*-**25b** as a colorless liquid (1.68 g, 82%). TLC: R_f = 0.17 (pentane/Et_2O=4:1); ^{1}H NMR (600 MHz, $CDCl_3$): δ = 0.58–0.63 (m, 1 H), 0.76–0.80 (m, 1 H), 1.15–1.24 (m, 1 H), 1.26–1.32 (m, 1 H), 2.57 (bs, 1 H), 3.40–3.53 (m, 2 H), 5.56 (td,

J = 57.4 Hz, 4.5 Hz, 1 H); ^{13}C NMR (125.7 MHz, $CDCl_3$): δ = 5.48 (–, t, J=4.5 Hz), 16.58 (+, t, J=4.0 Hz), 18.59 (+, t, J=27.1 Hz), 64.42 (–), 116.78 (+, t, J=237.5 Hz); IR (film): 3347, 3014, 2980, 1383, 1199, 1154, 1097 cm^{-1}; MS-EI (70eV): *m/z* (%) 119.1 (5, M–3H$]^+$), 72.0 (20, M–$CF_2]^+$), 59.1 (100, $C_3H_4F]^+$).

Racemic trans-(2-difluoromethylcyclopropyl)methyl iodide (*rac*-**11b**): Racemic *trans*-(2-difluoromethylcyclopropyl)methanol (**25b)** (1.68 g, 13.8 mmol) was converted according to GP 9 with triphenylphosphine (6.26 g, 23.9 mmol), imidazole (1.71 g, 25.1 mmol) and solid iodine (6.75 g, 26.5 mmol) in a diethyl ether/acetonitrile mixture (41 + 27 mL) to give the target iodide *rac*-**11b** as a slightly yellowish liquid (2.81 g, 88%). TLC: R_f = 0.17, pentane; ^{1}H NMR (300 MHz, $CDCl_3$): δ = 0.63–0.74 (m, 1 H), 1.06–1.16 (m, 1 H), 1.16–1.33 (m, 1 H), 1.48–1.61 (m, 1 H), 3.04–3.17 (m, 2 H), 5.63 (td, J = 57.4 Hz, 3.9 Hz, 1 H); ^{13}C NMR (125.7 MHz, $CDCl_3$): δ = 8.60 (–), 12.95 (–, t, J = 4.5 Hz), 18.85 (+, t, J=4.6 Hz), 25.73 (+, t, J=26.7 Hz), 115.70 (+, t, J=238.4 Hz); IR (film): 2966, 2361, 1466, 1436, 1411, 1378, 1179, 1099, 1033 cm^{-1}; MS–EI (70 eV): *m/z* (%) 105,1 (100, M-I$]^+$), 85.1 (20, $C_5H_6F]^+$), 77.0 (30, $C_4H_{10}F]^+$), 59.1 (95, $C_3H_4F]^+$), 41.2 (31, $C_3H_5]^+$).

Nickel(II) (R)-complex of 3-(2-difluoromethylcyclopropyl)alanine [(*R*)-B(dFmcpA)C, (2*R*,1'*S*,2'*R*)-**26b**]: (*R*)-**10** (2.00 g, 4.02 mmol) was alkylated with racemic *trans*-(2-difluoromethylcyclopropyl)methyl iodide **(11b)** (980 mg, 4.22 mmol) according to GP 10 using NaH (60% in oil, 193 mg, 4.8 mmol) in a DMF/MeCN mixture (2 + 4 mL) within 1 h to give the (2*R*,1'*S*,2'*R*) diastereomer [1.14 g, 47% based on (*R*)-**10**, d.e.≥98%], the (2*R*,1'*R*,2'*S*)-diastereomer [1.10 g, 45% based on (*R*)-**10**, d.e.≥98%] and a mixed fraction [0.107 g, 4.4% based on (*R*)-**10**]. For the (2*R*,1'*S*,2'*R*)-diastereomer: $[\alpha]_D^{20}$ = –2830.0 (c = 0.2, $CHCl_3$); ^{1}H NMR (600 MHz, $CDCl_3$): δ= –0.3 to –0.2 (m, 1 H), 0.6–0.7 (m, 1 H), 0.9–1.0 (m, 1 H), 1.0–1.1 (m, 1 H), 1.4–1.5 (m, 1 H), 2.0–2.1 (m, 1 H), 2.1–2.2 (m, 1 H), 2.4–2.5 (m, 1 H), 2.55–2.7 (m, 2 H), 3.4–3.5 (m, 1 H), 3.5–3.6 (m, 2 H), 3.55 (d, J = 12.7 Hz, 1 H), 3.90 (dd, J = 3.5 Hz, 9.6 Hz, 1 H), 4.43 (d, J = 12.7 Hz, 1 H), 5.51 (td, J = 57.4 Hz, 4.4 Hz, 1 H), 6.55–

6.65 (m, 2 H), 6.87 (d, J = 7.2 Hz, 1 H), 7.07–7.12 (m, 1 H), 7.12–7.20 (m, 1 H), 7.22–7.28 (m, 1 H), 7.28–7.36 (m, 2 H), 7.38–7.44 (m, 1 H), 7.46–7.54 (m, 2 H), 8.00–8.50 (m, 3 H); ^{13}C NMR (125.7 MHz, $CDCl_3$): δ=7.2 (–), 11.0 (+, t, J = 4.5Hz), 20.9 (+, t, J = 26.9 Hz), 24.0 (–), 30.7 (–), 39.1 (–), 57.2 (–), 63.1 (–), 69.9 (+), 70.1 (+), 116.7 (+, t, J = 238.0 Hz), 120.7 (+), 123.7 (+), 126.2 (–), 127.3 (+), 127.4(+), 128.1 (+), 128.7 (+), 128.8 (+), 128.9 (+) 129.0 (+), 129.8 (+), 129.8 (+), 131.5 (+), 132.2 (+), 133.1 (+), 133.2 (–), 133.6 (–), 142.2 (–), 170.5 (–), 178.9 (–), 180.5 (–); MS-ESI: (positive) *m/z* (%) 624.2 (100, M+Na]$^+$), 1225.0 (90, 2M+Na]$^+$), 1827.4 (55, 3M+Na]$^+$), 602.2 (16,M+H]$^+$).

(2R,1'S,2'R)-3-(2-Difluoromethylcyclopropyl)alanine [(2*R*,1'*S*,2'*R*)-**9b**]: The complex (2*R*,1'*S*,2'*R*)-**26b** (730 mg, 1.21 mmol) was decomposed, and the amino acid was purified according to GP 11 to give the target amino acid (210 mg, 97%); $[\alpha]_D^{20}$ = +30.4 (c = 0.5, H_2O); ^{1}H NMR (600 MHz, D_2O): δ = 1.21–1.30 (m, 1 H), 1.63–1.75 (m, 1 H), 1.98–2.08 (m, 1 H), 3.81 (t, J = 6.0 Hz, 1 H), 5.67 (td, J = 57.1 Hz, 4.8 Hz, 1 H); MS-ESI: (positive) *m/z* (%) 180 (100, M+H]$^+$), (negative) *m/z* (%) 357 (100, 2M–H]$^-$), 178 (55, M–H]$^-$).

Nickel(II) (S)-complex of 3-(2-difluoromethylcyclopropyl)alanine [(*S*)-B(dFmcpA)C, (2*S*,1'*S*,2'*R*)-**26b**]: (*S*)-**10** (756 mg, 1.52 mmol) was alkylated with racemic *trans*-(2-difluoromethylcyclopropyl)methyl iodide (**11b)** (370 mg, 1.60 mmol) according to GP 10 using NaH (60% in oil, 73 mg, 1.82 mmol) in a DMF/MeCN mixture (1 + 2 mL) within 1 h to give the (2*S*,1'*R*,2'*S*)-diastereomer [442 mg, 48% based on (*S*)-**10**, d.e.≥98%], the (2*S*,1'*S*,2'*R*)-diastereomer [409 mg, 45% based on (*S*)-**10**, d.e.≥98%] and a mixed fraction (40 mg, 4% based on (*S*)-**10**). For the (2*S*,1'*S*,2'*R*)-diastereomer: $[\alpha]_D^{20}$ = +2200 (c = 0.2, $CHCl_3$); ^{1}H NMR (600 MHz, $CDCl_3$): δ = 0.25–0.37 (m, 1 H), 0.37–0.41 (m, 1 H), 0.85–0.95 (m, 1 H), 1.30–1.45 (m, 2 H), 2.00–2.10 (m, 1 H), 2.10–2.19 (m, 1 H), 2.19–2.23 (m, 1 H), 2.43–2.53 (m, 1 H), 2.60–2.70 (m, 1 H), 3.40–3.47 (m, 1 H), 3.47–3.6 (m, 2 H), 3.53 (d, J = 12.7 Hz, 1 H), 3.90–3.97 (m, 1 H), 4.41 (d, J = 12.7 Hz, 1 H), 5.41 (td, J = 57.2 Hz, 4.1 Hz, 1 H), 6.55–6.60 (m, 1 H), 6.60–6.65 (m, 1 H), 6.78–6.83 (m, 1 H), 7.08–7.12 (m,

1 H), 7.12–7.17 (m, 1 H), 7.25–7.38 (m, 3 H), 7.39–7.46 (m, 1 H), 7.46–7.57 (m, 2 H), 8.00–8.10 (m, 3 H); ^{13}C NMR (125.7 MHz, $CDCl_3$): δ = 8.38 (–, t, *J* = 4.2 Hz), 11.38 (+, dd, *J*=4 Hz, 5 Hz), 20.10 (+, dd, 26.1 Hz, 28.0 Hz), 23.85 (–), 30.67 (–), 39.18 (–), 57.19 (–), 63.16 (–), 69.96 (+), 70.15 (+), 116.44 (+, t, *J*=32.0 Hz), 120.66 (+), 123.66 (+), 126.25 (–), 127.33 (+), 127.44 (+), 128.13 (+), 128.81 (+), 128.85 (+), 129.04 (+), 129.12 (+), 129.83 (+), 131.29 (+), 131.45 (+), 132.15 (+), 133,11 (+), 133.28 (–), 133.62 (–), 142.29 (–), 170.33 (–), 178.76 (–), 180.36 (–); MS-ESI: (positive) *m/z* (%) 1828 (55, 3M+Na]$^+$), 1225 (100, 2M+Na]$^+$), 624 (70, M+Na]$^+$), 602 (14, M+H]$^+$), (negative) *m/z* (%) 600 (100, M–H]$^-$).

(2S,1'S,2'R)-3-(2-Difluoromethylcyclopropyl)alanine [(2*S*,1'*S*,2'*R*)-**9b**]: The complex (2*S*,1'*S*,2'*R*)-**26b** (180 mg, 299 μmol) was decomposed, and the amino acid was purified according to GP 11 to give the target amino acid (50 mg, 93%). $[\alpha]_D^{20}$ = –16.0 (*c* = 0.3 in H_2O); ^{1}H NMR (600 MHz, D_2O):δ = 0.65–0.75 (m, 1 H), 0.90–1.00 (m, 1 H), 1.10–1.20 (m, 1 H), 1.30–1.41 (m, 1 H), 1.80–1.88 (m, 1 H), 2.01–2.08 (m, 1 H), 3.87 (dd, *J* = 6.6 Hz, 5.4 Hz, 1 H), 4.72 (bs, 3 H), 5.76 (td, *J* = 57.2 Hz, 4.8 Hz, 1 H); ^{13}C NMR (125.7 MHz, D_2O): δ = 6.93 (–, dd, *J* = 5.6 Hz, 3.3 Hz), 10.10 (+, dd, *J* = 5.9 Hz, 3.5 Hz), 19.92 (+, t, *J* = 27.1 Hz), 33.30 (–), 54.84 (–), 117.99 (+, t, *J* = 235.4 Hz), 174.20 (–); MS-ESI: (positive) *m/z* (%) 180.0 (100, M+H]$^+$).

Monofluoromethylcyclopropylalanine (**9c)**

Racemic methyl trans-2-(fluoromethyl)cyclopropane carboxylate (**14c**): The reaction was performed in a PTFE flask. A Deoxo-Fluor® solution in toluene (50% w/w, 24.3 g, 55.0 mmol) was added under N_2 flow with stirring to a solution of racemic methyl *trans*-2-(hydroxymethyl)cyclopropane carboxylate (**15)** (6.5 g, 50.0 mmol) in anhydrous CH_2Cl_2 (5 mL), and the mixture was stirred overnight at ambient temperature. The resulting solution was poured into a vigorously stirred sat. aq. $NaHCO_3$ solution (150 mL), and the mixture was stirred until the CO_2 gas evolution had ceased; the organic phase was separated, the water

phase was extracted with CH_2Cl_2 (3 × 50 mL), the combined organic phases were dried over $MgSO_4$, filtered and concentrated under reduced pressure. The crude product was purified by column chromatography (silica gel, eluting with pentane/diethyl ether 4:1) to give the target monofluoroester **14c** as a colorless liquid (3.1 g, 47%). ^{1}H NMR (250 MHz, $CDCl_3$): δ = 0.80–0.98 (m, 1 H), 1.15–1.37 (m, 1 H), 1.60–1.72 (m, 1 H), 1.73–1.92 (m, 1 H), 3.67 (s, 3 H), 4.18 (ddd, *J* = 7.2 Hz, 9.8 Hz, 48.2 Hz, 1 H), 4.40 (ddd, *J* = 6.0 Hz, 9.8 Hz, 48.2 Hz, 1 H); ^{13}C NMR (62.9 MHz, $CDCl_3$): δ = 12.1 (–, d, *J* = 7.0 Hz), 18.0 (+, d, *J* = 5.8 Hz), 21.3 (+, d, *J* = 24.7 Hz), 51.8 (+), 84.7 (–, d, *J* = 168.7 Hz), 173.46 (–, d, *J* = 1.7 Hz); IR (film): 2958, 2904, 1733, 1439, 1373, 1327, 1275, 1208, 1095, 979 cm^{-1}; MS-EI (70 eV): *m/z* (%) 132.1 (25, M$^+$), 131.1 (20, M–H$^+$), 101.0 (100, M–OMe$^+$), 71.0 (30, $C_4H_4F^+$), 47.1 (80, $C_2H_4F^+$).

Racemic trans-(2-monofluoromethylcyclopropyl)methanol (*rac*-**25c**): The racemic methyl *trans*-2-(fluoromethyl)cyclopropanecarboxylate (**14c**) (2.2 g, 16.7 mmol) was reduced with lithium aluminum hydride in diethyl ether (1.13 M, 7.4 mL, 8.4 mmol) according to GP 8. The crude product was purified by column chromatography (silica gel, eluting with pentane/diethyl ether 1:1) to give the target monofluoroalcohol *rac*-**25c** as a colorless liquid (1.33 g, 76%). ^{1}H NMR (600 MHz, $CDCl_3$): 0.47–0.55 (m, 2 H), 1.00–1.11 (m, 2 H), 2.64–2.74 (bs, 1 H), 3.33–3.49 (m, 2 H), 4.07–4.31 (m, 2 H); ^{13}C NMR (125.7 MHz, $CDCl_3$): 7.6 (–, d, *J* = 7.3 Hz), 16.5 (+, d, *J* = 25.1 Hz), 19.2 (+, d, *J* = 5.7 Hz), 65.3 (–, d, *J* = 1.3 Hz), 86.9 (–, d, *J* = 165.8 Hz); IR (film): 3354, 3007, 2957, 2897, 1471, 1387, 1176, 1067, 1024, 966 cm^{-1}; MS-EI (70 eV): *m/z* (%) 103.1 (1, M–H$^+$), 87.1 (12, M–OH$^+$), 85.0 (38, M–F$^+$), 83.0 (40, M–F–2H$^+$), 67.1 (36), 43.1 (85), 41.2 (100, $C_3H_5^+$).

Racemic trans-(2-monofluoromethylcyclopropyl)methyl iodide (*rac*-**11c**): The racemic *trans*-(2-monofluoromethylcyclopropyl)methanol (**25c**) (1.33 g, 12.8 mmol) was converted into *rac*-**11c** according to GP 9 using triphenylphosphine (5.81 g, 22.2 mmol), imidazole (1.58 g, 23.3 mmol) and solid iodine (6.25 g, 24.6 mmol) in a diethyl ether/acetonitrile mixture

(38 + 25 mL) to give the target iodide as a light yellow liquid (2.50 g, 91%). TLC: R_f = 0.11, pentane; ^{1}H NMR (300 MHz, $CDCl_3$): 0.59–0.68 (m, 1 H), 0.82–0.91 (m, 1 H), 1.11–1.25 (m, 1 H), 1.26–1.38 (m, 1 H), 3.13 (d, J = 7.6 Hz, 2 H), 4.22 (dd, J = 7.0 Hz, 48.4 Hz, 2 H); ^{13}C NMR (125.7 MHz, $CDCl_3$): 10.54 (–, d, J = 0.9 Hz) 15.40 (–, d, J = 7.0 Hz), 21.38 (+, d, J = 6.6 Hz), 24.06 (+, d, J = 25.2 Hz), 85.96 (–, d, J = 167.6 Hz); IR (film): 2958, 2895, 1433, 1412, 1266, 1175, 1038, 994 cm^{-1}; MS-EI (70 eV): *m/z* (%) 41.2 (100, $C_3H_5^+$), 87.1 (38, M–I$^+$), 67.1 (36, $C_5H_7^+$).

Nickel(II) (R)-complex of 3-(2-fluoromethylcyclopropyl)alanine [(*R*)-B(mFmcpA)C, (2*R*,1'*S*,2'*R*)-**26c**]: Complex (*R*)-**10** (2.40 g, 4.8 mmol) was alkylated with racemic *trans*-(2-fluoromethylcyclopropyl)methyl iodide (**11c**) (1.07 g, 5.0 mmol) according to GP 10, using NaH (60% in oil, 230 mg, 5.7 mmol) in a DMF/MeCN mixture (2.5 + 5 mL) within 3 h to give, after chromatographic separation (silica gel, eluting with EtOAc), the (2*R*,1'*S*,2'*R*)-diastereomer [1.31 g, 47% based on (*R*)-**10**, d.e.≥98%], the (2*R*,1'*R*,2'*S*)-diastereomer [1.19 g, 42% based on (*R*)-**10**, d.e.≥98%] and a mixed fraction [0.102 g, 3.6% based on (*R*)-**10**].

*(2R,1'S,2'R)-3-(2-Fluoromethylcyclopropyl)alanine [(2R,1'S,2'R)-***9c***]*: The complex (2*R*,1'*S*,2'*R*)-**26c** (1.11 g, 1.90 mmol) was decomposed, and the amino acid was purified according to GP 11 to give the target amino acid (172 mg, 96%).

Nickel(II) (S)-complex of 3-(2'-fluoromethylcyclopropyl)alanine [(*S*)-B(mFmcpA)C, (2*S*,1'*S*,2'*R*)-**26c**]: Complex (*S*)-**10** (2.40 g, 4.8 mmol) was alkylated with racemic *trans*-(2-fluoromethylcyclopropyl)methyl iodide (**11c**) (1.07 mg, 5.0 mmol) according to GP 10, using NaH (60% in oil, 230 mg, 5.7 mmol) in a DMF/MeCN mixture (2.5 + 5 mL) within 3 h to give, after chromatographic separation (silica gel, eluting with EtOAc), the (2*S*,1'*R*,2'*S*)-diastereomer [1.25 g, 45% based on (*S*)-**10**, d.e.≥98%], the (2*S*,1'*S*,2'*R*)-diastereomer [1.23 g, 44% based on (*S*)-**10**, d.e.≥98%] and a mixed fraction [143 mg, 5% based on (*S*)-**10**].

(2S,1'S,2'R)-3-(2-Fluoromethylcyclopropyl)alanine [(2*S*,1'*S*,2'*R*)-**9c**]: The complex (2*S*,1'*S*,2'*R*)-**25c** (1.20 g, 2.05 mmol) was decomposed, and the amino acid was purified according to GP 11 to give the target amino acid (311 mg, 94%).

Hormaomycin analogues containing (fluoromethylcyclopropyl)alanine moieties

[(Trifluoromethylcyclopropyl)alanyl]hormaomycin analogue **8a**

N-Fmoc-(2R,1'R,2'R)-3-(2'-Trifluoromethylcyclopropyl)alanine (Fmoc-(R)tFmcpA-OH), [(2*R*,1'*R*,2'*R*)-**41a**]: Analogous to a procedure in [5] a solution of Fmoc-OSu (459 mg, 1.36 mmol) in acetone (7 mL) was added to a vigorously stirred solution of (2*R*,1'*R*,2'*R*)-3-(2'-trifluoromethylcyclopropyl)alanine (**9a**) (225 mg, 1.14 mmol) and $NaHCO_3$ (0.202 g, 2.40 mmol) in water (5 mL) (if a precipitate formed, acetone and/or water was added to obtain a homogeneous solution), and stirring was continued for an additional 3 h. Acetone was then removed under reduced pressure, and the pH of the residual aqueous solution was adjusted to 1 with aq. 1 M $KHSO_4$. The resulting emulsion was extracted with diethyl ether (30 mL), and the ethereal layer was back-extracted with 3% aq. $NaHCO_3$ (5 × 10 mL). The combined aqueous fractions were washed with diethyl ether (2 × 10 mL), acidified to pH~2 with aq. 1 M $KHSO_4$, and the resulting emulsion was extracted with diethyl ether (4 × 10 mL). The organic phase was washed with aq. 1 M $KHSO_4$ (2 × 10 mL), water (3 × 10 mL), brine (2 × 5 mL), dried, filtered and concentrated under reduced pressure. The residue was triturated with cold pentane and filtered. The resulting extremely viscous oil was dried at 0.02 Torr for a prolonged time to give the target protected amino acid **41a** (390 mg, 82%) as a colorless foam. R_f = 0.08 (EtOAc/hexane 1:1); m. p. (softening) 50–57 °C; $[\alpha]_D^{20}$ = –56.7 (*c* = 0.36, $CHCl_3$); ^{1}H NMR (600 MHz, $CDCl_3$): 0.38–0.44 + 0.57–0.64 + 0.79–0.86 (3 × m, 1 H), 0.91–0.97 + 1.00–1.09 (2 × m, 1 H), 1.14–1.22 + 1.26–1.34 (2 × m, 1 H), 1.35–1.53 + 1.85–1.88 (2 × m, 1 H), 1.80–1.85 (m, 1 H), 3.75–3.79 + 4.53–4.67 (2 × m, 1 H), 3.95–4.01 + 4.47–4.52 (2 × m, 1 H), 4.16–4.23 (m, 1 H), 4.36–4.47 (m, 1 H), 5.52 (d, *J* = 7.9 Hz, 0.7 H), 6.76 (d, *J* = 5.9 Hz, 0.3 H), 7.27–7.31 (m, 2 H), 7.35–7.41 (m, 2 H), 7.51 (t, J = 8.1 Hz, 0.6 H), 7.58 (t, J = 8.4 Hz, 1.4 H), 7.74 (d, *J* = 7.4 Hz, 2 H), 7.85–8.65 (bs, 1 H); ^{13}C NMR (125.7 MHz, $CDCl_3$, mixture of two rotamers): δ = 7.9 (–), 11.5 (+), 19.7 (+, q, *J* = 37.0 Hz), 34.7 (–), 47.1 (+), 53.5 and 53.8 (+), 67.1 and 67.9 (–), 120.0 (+), 125.0 (+), 126.0 (–, q, *J* = 272.4 Hz), 127.07 (+), 127.8 (+), 141.3 (–), 143.1 (–), 143.8 (–), 155.8 and 156.71 (–), 174.96 and 175.79 (–); MS-ESI: (positive) *m/z* (%) 1302 (35, 3M–H+2Na]$^+$), 861 (100, 2M+Na]$^+$), 442 (M+Na]$^+$),

(negative) *m/z* (%) 837 (100, 2M–H]$^{-}$), 418 (16, M–H]$^{-}$), 222 (14, M–FmOH–H]$^{-}$), 196 (15, FmOH]$^{-}$).

Dicyclopropylmethyl (5R,8S,11S)-11-sec-butyl-1-(9H-fluoren-9-yl)-3,6,9-trioxo-8-[(R)-1-phenylethyl]-5-{[(1S,2R)-2-(trifluoromethyl)cyclopropyl]methyl}-2-oxa-4,7,10-triazadode-can-12-oate [Fmoc-(R)tFmcpA-(βMe)Phe-Ile-ODCPM] (**47a**): Dipeptide **42** (434 mg, 834 µmol) was taken up with EtOAc (20 mL) and hydrogenated over 10% Pd/C (250 mg) under ambient pressure of hydrogen for 2 h. The reaction mixture was filtered through a pad of Celite® and concentrated under reduced pressure to give the deprotected dipeptide, which was directly used for the coupling with **41a** (360 mg, 860 µmol), using EDC (172 mg, 896 µmol), HOAt (120 mg, 883 µmol) and TMP (310 µL, 2.5 mmol) according to GP 2. During the reaction, a colorless precipitate formed. The mixture was diluted with diethyl ether (50 mL), stirred for 30 min and filtered to give the crude product (1st crop, 473 mg after drying *in vacuo*). The filtrate was concentrated under reduced pressure at ambient temperature and diluted with diethyl ether (20 mL) to give more of the crude product (2nd crop, 130 mg after drying *in vacuo*). The remaining filtrate was subjected to the usual aqueous work-up according to GP 2 to give the last portion of crude product (3rd crop, 100 mg after drying *in vacuo*). The combined crude products were recrystallized from THF/hexane, the resulting off-white solid was dissolved in chloroform (50 mL) and subjected to the usual aqueous work-up according to GP 2 to give the pure tripeptide **47a** as a colorless solid (535 mg, 81%). R_f = 0.52; EtOAc/hexane 2:3; m. p. 151–155°C; $[\alpha]_D^{20}$ = –3.8 (*c* = 0.26, $CHCl_3$); ^{1}H NMR (500 MHz, $CDCl_3$): δ = 0.28–0.40 (m, 4 H), 0.43–0.53 (m, 2 H), 0.57–0.66 (m, 3 H), 0.88 (d, *J* = 6.7 Hz, 3 H), 0.90 (t, *J* = 7.4 Hz, 3 H), 0.94–1.00 (m, 1 H), 1.03–1.13 (m, 2 H), 1.13–1.22 (m, 2 H), 1.30–1.50 (m, 2 H), 1.41 (d, *J* = 6.8 Hz, 3 H), 1.58–1.70 (m, 1 H), 1.81–1.97 (m, 2 H), 3.28–3.38 (m, 1 H), 3.86 (t, *J* = 8.4 Hz, 1 H), 4.28 (t, *J* = 6.9 Hz, 1 H), 4.39–4.48 (m, 3 H), 4.48–4.57 (m, 1 H), 4.76 (t, *J* = 8.0 Hz, 1 H), 5.76 (d, *J* = 7.7 Hz, 1 H), 6.40 (d, *J* = 7.5 Hz, 1 H), 7.11 (d, *J* = 8.1 Hz, 1 H), 7.20–7.34 (m, 5 H), 7.35 (t, *J* = 7.4 Hz, 2 H), 7.46 (t, *J* = 7.4 Hz, 2 H), 7.60 (t, *J* = 8.0 Hz, 2 H), 7.79 (d, *J* = 7.5 Hz, 2 H); ^{13}C NMR (125.7 MHz, $CDCl_3$): δ = 2.6 (–), 2.9 (–), 8.2 (–), 11.6 (+), 14.4 (+), 14.7 (+), 15.1 (+), 15.2 (+), 16.9 (+), 19.7 (+, q, *J*=37.1 Hz), 25.1 (–), 35.1 (–), 38.1 (+), 42.1 (+), 47.1 (+), 54.7 (+), 56.5 (+), 58.8 (+), 67.2 (–), 83.4 (+), 119.9 (+), 125.0 (+), 127.1 (+), 127.6 (+), 127.7 (+), 128.5 (+), 141.2 (–), 141.3 (–), 141.7 (–), 143.6 (–), 143.8 (–), 156.0 (–), 169.7 (–), 170.6 (–), 170.9 (–); MS-ESI: (positive) *m/z* (%) 1597.2 (90, 2M+Na]$^{+}$), 810.4 (100, M+Na]$^{+}$), 588.3 (55).

Dicyclopropylmethyl (5S,8R,11S,14S)-14-sec-butyl-1-(9H-fluoren-9-yl)-3,6,9,12-tetraoxo-5,11-bis[(R)-1-phenylethyl]-8-{[(1S,2R)-2-(trifluoromethyl)cyclopropyl]methyl}-2-oxa-4,7,10,13-tetraazapentadecan-15-oate [Fmoc-(βMe)Phe-(R)tFmcpA-(βMe)Phe-Ile-ODCPM] (**49a**): The tripeptide **47a** (394 mg, 500 µmol) was deprotected according to GP 1, and the resulting *C*-protected tripeptide was then directly coupled with Fmoc-(βMe)Phe-OH **46** (211 mg, 525 µmol) according to GP 2 using EDC (99 mg, 518 µmol), HOAt (70 mg, 512 µmol) and TMP (175 mg, 1440 µmol) in CH_2Cl_2 (3 mL). After 16 h, the reaction mixture was diluted with chloroform (50 mL) and subjected to the usual aqueous work-up according to GP 2 to give the crude tetrapeptide, which was twice recrystallized from THF/hexane, giving the pure target tetrapeptide **49a** as an off-white solid (440 mg, 93%). R_f = 0.29; $CHCl_3$/MeOH 70:1; m. p. 210–215 °C (decomp.); $[\alpha]_D^{20}$ = –26.3 (*c* = 0.32, THF); ^{1}H NMR (250 MHz, $CDCl_3$): δ = 0.22–0.36 (m, 4 H), 0.41 (t, *J* = 8.9 Hz, 3 H), 0.48–0.61 (m, 2 H), 0.62–0.71 (m, 1 H), 0.76 (d, *J* = 6.9 Hz, 3 H), 0.82 (t, *J* = 7.3 Hz, 3 H), 0.92–1.09 (m, 4 H), 1.09–1.18 (m, 1 H), 1.22–1.46 (m, 2 H), 1.26 (d, *J* = 6.9 Hz, 3 H), 1.39 (d, *J* = 6.6 Hz, 3 H), 1.71–1.89 (m, 1 H), 3.09–3.36 (m, 3 H), 3.81 (t, *J* = 8.4 Hz, 1 H), 4.15–4.33 (m, 3 H), 4.35–4.64 (m, 3 H), 4.62 (t, *J* = 8.3 Hz, 1 H), 5.95 (d, *J* = 7.1 Hz, 1 H), 6.40 (d, *J* = 7.4 Hz, 1 H), 7.08–7.45 (m, 16 H), 7.57 (t, *J* = 8.9 Hz, 2 H), 7.76 (d, *J* = 7.3 Hz, 2 H); ^{13}C NMR (62.9 MHz, $CDCl_3$): δ = 3.3 (–), 3.5 (–), 3.6 (–), 3.7 (–), 12.2 (+), 15.6 (+), 15.9 (+), 16.1 (+), 17.7 (+), 18.7 (+), 18.9 (–), 23.2 (+), 26.2 (–), 35.0 (–), 39.1 (+), 42.8 (+), 44.1 (+), 48.5 (+), 52.8 (+), 57.4 (+), 59.3 (+), 60.2 (+), 62.1 (+), 67.8 (–), 83.4 (+), 115,1 (+, q, *J* = 291.4 Hz), 126.4 (+), 127.5 (+), 127.7 (+), 128.1 (+), 128.6 (+), 129.1 (+), 129.1 (+), 129.3 (+), 129.4 (+), 142.5 (–), 144.1 (–), 144.4 (–), 145.4 (–), 145.6 (–), 157.8 (–), 171.1 (–), 171.5 (–), 171.9 (–), 172.1 (–).

Boc-(4-Pe)Pro-[MeZ-a-Thr]-(βMe)Phe-(S)tFmcpA-(βMe)Phe-Ile-ODCPM (**51a**): The tetrapeptide **49a** (332 mg, 350 µmol) was *N*-deprotected according to GP 1 with diethylamine (5 mL) and THF (5 mL), taken up with anhydrous CH_2Cl_2 (5 mL), a solution of the ester acid **45** (194 mg, 385 µmol), HATU (160 mg, 420 µmol) and HOAt (53 mg, 385µmol) in CH_2Cl_2 (3 mL) was added, and the reaction mixture was cooled to 4 °C. After this, a solution of DIEA (65 µL, 48 mg, 368 µmol) and TMP (140 µL, 127 mg, 1050 µmol) in CH_2Cl_2 (2 mL) was added at the same temperature within 5 min. The temperature was allowed to reach 20 °C, and stirring was continued for an additional 15 h. After aqueous work-up according to GP 2 and two recrystallizations from EtOAc/hexane (1:2), the target hexadepsipeptide **51a** (390 mg, 92%) was obtained as a colorless solid. R_f = 0.46 (EtOAc/hexane 1:1); m. p. 125–127 °C; $[\alpha]_D^{20}$ = –29.0 (*c* = 0.2, THF); ^{1}H NMR (250 MHz, $CDCl_3$): δ = 0.24–0.68 (m, 12 H), 0.75 (d, *J* = 6.9 Hz, 3 H), 0.88 (t, *J* = 7.1 Hz, 3 H), 0.98–1.17 (m, 5 H), 1.18–1.46 (m, 1 H), 1.24 (d,

J = 6.6 Hz, 3 H), 1.27 (d, J = 6.7 Hz, 3 H), 1.36 (s, 9 H), 1.40 (d, J = 6.7 Hz, 3 H), 1.68 (d, J = 7.0 Hz, 3 H), 1.75–1.94 (m, 2 H), 2.29–2.46 (m, 1 H), 2.32 (s, 3 H), 3.07–3.33 (m, 4 H), 3.68 (t, J = 8.6 Hz, 1 H), 3.93 (t, J = 8.3 Hz, 1 H), 4.18–4.27 (m, 1 H), 4.32–4.54 (m, 4 H), 4.64 (t, J = 9.6 Hz, 1 H), 4.95–5.13 (m, 2 H), 5.20–5.34 (m, 1 H), 5.44–5.63 (m, 2 H), 6.60 (d, J = 7.7 Hz, 1 H), 6.70 (d, J = 8.8 Hz, 1 H), 6.82 (d, J = 8.4 Hz, 1 H), 7.05–7.37 (m, 14 H), 7.45 (d, J = 10.1 Hz, 1 H), 7.76 (d, J = 8.1 Hz, 1 H); ^{13}C NMR (62.9 MHz, $CDCl_3$): δ = 2.48 (–), 2.82 (–), 2.90 (–), 3.01 (–), 11.59 (+), 13.20 (+), 14.16 (+), 14.64 (+), 15.57 (+), 17.77 (+), 18.41 (–), 18.86 (+), 19.43 (+), 21.11 (+), 21.77 (+), 25.23 (–), 26.85 (–), 28.23 (+), 31.46 (–), 31.53 (–), 36.28 (–), 36.32 (+), 37.30 (+), 40.45 (+), 42.00 (+), 50.62 (+), 52.08 (+), 56.43 (+), 59.30 (+), 59.49 (+), 61.01 (+), 61.62 (+), 61.99 (+), 66.89 (–), 70.53 (+), 80.93 (–), 83.24 (+), 116,2 (+, q, J = 287.3 Hz), 127.00 (+), 127.06 (+), 127.10 (+), 127.60 (+), 127.69 (+), 128.47 (+), 128.63 (+), 128.68 (+), 128.82 (+), 128.93 (+), 133.21 (C_{quat}), 137.83 (–), 141.73 (–), 141.90 (–), 154.76 (–), 155.75 (–), 170.38 (–), 170.43 (–), 170.79 (–), 171.37 (–), 173.41 (–), 174.06 (–).

N-MeZ-protected cyclohexadepsipeptide **52a** *(Cyclo-F$_3$6-MeZ)*: The hexadepsipeptide Boc-(4-Pe)Pro-[MeZ-*a*-Thr]-(βMe)Phe-(*S*)tFmcpA-(βMe)Phe-Ile-ODCPM **51a** (300 mg, 247 µmol) was deprotected at the termini by treatment with a 2 M HCl solution in ethyl acetate (5 mL). The reaction mixture was stirred for 20 min in a dark place (aluminum foil jacket) at ambient temperature, and all volatiles were removed under reduced pressure without any heating. The residue was triturated with anhydrous diethyl ether (10 mL) to give the hydrochloride of the deprotected material as a colorless solid (232 mg, 220 µmol, 89%). The terminally deprotected hexadepsipeptide, HATU (110 mg, 288 µmol) and HOAt (33 mg, 244 µmol) were dissolved in cold (4 °C, internal temperature) anhydrous CH_2Cl_2 (2.5 L), and a solution of DIEA (120 µL, 93 mg, 720 µmol) in CH_2Cl_2 (100 mL) was added dropwise within 1 h, the cooling bath was removed, and the mixture was stirred at ambient temperature for 2 h. The mixture was cooled again to 4 °C (internal temperature), then second portions each of HATU (110 mg, 288 µmol) and HOAt (33 mg, 244 µmol) were added, followed by dropwise addition of a solution of DIEA (120 µL, 93 mg, 720 µmol) in CH_2Cl_2 (100 mL) within 1 h. The cooling bath was removed, and the mixture was stirred for 18 h at ambient temperature. The mixture was concentrated under reduced pressure, subjected to aqueous work-up according to GP 2 to give the crude protected cyclohexadepsipeptide (180 mg, 73%) which was finally purified by HPLC to give the pure product (132 mg, 132 µmol, 54%). Preparative HPLC: isocratic, 82% B for 25 min, t_R = 15.4 min, purity > 98%; analytical HPLC: gradient 20% → 100% B for 20 min, then isocratic 100% B for 5 min t_R = 11.9 min, purity > 98%; $[\alpha]_D^{20}$ = –15.5 (c = 0.20, $CHCl_3$); ^{1}H NMR (600 MHz, $CDCl_3$): δ = 0.61–

0.67 (m, 1 H), 0.72 (d, *J* = 6.6 Hz, 3 H), 0.71–0.77 (m, 1 H), 0.79 (t, *J* = 7.2 Hz, 3 H), 1.04–1.12 (m, 1 H), 1.23 (d, *J* = 6.6 Hz, 3 H), 1.27–1.34 (m, 1 H), 1.37 (d, *J* = 6.6 Hz, 3 H), 1.37–1.43 (m, 1 H), 1.45–1.54 (m, 1 H), 1.54–1.57 (m, 1 H), 1.57 (d, *J* = 6.6 Hz, 3 H), 1.65 (dd, *J* = 6.6 Hz, 1.5 Hz, 3 H), 1.66–1.76 (m, 2 H), 2.20–2.25 (m, 1 H), 2.35 (s, 3 H), 3.01–3.07 (m, 1 H), 3.15–3.28 (m, 2 H), 3.54 (dq, *J* = 7.2 Hz, 6.6 Hz, 1 H), 3.71 (dd, *J* = 6.0 Hz, 5.4 Hz, 1 H), 3.74–3.77 (m, 1 H), 3.98 (dd, *J* = 10.5 Hz, 6.3 Hz, 1 H), 4.01–4.08 (m, 1 H), 4.46–4.54 (m, 2 H), 4.52–4.55 (m, 1 H), 4.67–4.70 (m, 1 H), 5.03 (d, *J* = 12.0 Hz, 1 H), 5.15 (d, *J* = 12.0 Hz, 1 H), 5.19–5.25 (m, 1 H), 5.39 (qd, *J* = 6.6 Hz, 1.8 Hz, 1 H), 5.56 (dq, *J* = 10.8 Hz, 6.6 Hz, 1 H), 5.96 (d, *J* = 5.3 Hz, 1 H), 6.20 (d, *J* = 7.4 Hz, 1 H), 6.28 (d, *J* = 9.7 Hz, 1 H), 6.49 (d, *J* = 9.4 Hz, 1 H), 7.11–7.37 (m, 14 H) 7.32 (d, *J* = 7.4 Hz, 1 H), 7.45 (d, *J* = 8.7 Hz, 1 H); ^{13}C NMR (150.8 MHz, $CDCl_3$): δ = 10.3 (+), 13.3 (+), 14.6 (+), 17.3 (–), 17.7 (+), 18.4 (+), 21.2 (+), 21.3 (+), 24.7 (–), 32.0 (–), 35.4 (–), 36.6 (+), 39.4 (+), 44.5 (+), 52.5 (–), 53.3 (+), 54.6 (+), 58.6 (+), 59.0 (+), 59.4 (+), 60.1 (+), 60.7 (+), 67.2 (–), 72.6 (+), 127.1 (+), 127.2 (+), 127.5 (+), 127.6 (+), 128.3 (+), 128.6 (+), 128.8 (+), 129.2 (+), 127.8 (+), 128.0 (+), 133.2 (–), 137.9 (–), 140.9 (–), 142.6 (–), 156.3 (–), 169.0 (–), 170.3 (–), 170.6 (–), 171.1 (–), 171.4 (–), 173.1 (–).

N-Teoc-(2S,1'S,2'R)-3-(2'-Trifluoromethylcyclopropyl)alanine (Teoc-(S)tFmcA-OH, **53a***)*: A solution of TeocOSu (43 mg, 164 µmol) in acetone (1 mL) was added to a vigorously stirred solution of (2*S*,1'*S*,2'*R*)-3-(2'-trifluoromethylcyclopropyl)alanine *(2S,1'S,2'R)*-**9a** (27 mg, 137 µmol) and $NaHCO_3$ (24 mg, 286 µmol) in water (1 mL) (if an emulsion formed, acetone and/or water was added to obtain a homogeneous solution), and stirring was continued for another 2 h. *N*,*N*-Dimethylaminopropylamine (8 µL, 6,4 mg, 52 µmol) was then added. After an additional 10 min, acetone was removed under reduced pressure, and the pH of the residual aqueous solution was adjusted to 2–3 with aq. 1 M $KHSO_4$. The resulting emulsion was extracted with diethyl ether (50 mL), and the ethereal layer was washed with aq. 1 M $KHSO_4$ (2 × 10 mL), water (3 × 10 mL), brine (2 × 5 mL), dried over $MgSO_4$, filtered and concentrated under reduced pressure. The residual oil was dried overnight *in vacuo* to give a glass-like product (38 mg, 81%). R_f = 0.24 [EtOAc/hexane 1:3 (2% AcOH)]; $[\alpha]_D^{20}$ = +22.80 (*c* = 0.46, $CHCl_3$); ^{1}H NMR (250 MHz, $CDCl_3$): δ = 0.04 (s, 9H), 1.00 (dd, *J* = 9.5 Hz, 7.3 Hz, 2 H), 1.11–1.18 (m, 1 H), 1.60–1.95 (m, 2 H), 1.98–2.19 (m, 2 H), 4.14–4.23 (m, 3 H), 4.33–4.59 (m, 1 H), 5.33–5.46 (m, 1 H), 7.08–7.25 (bs, 1 H); ^{13}C NMR (62.9 MHz, $CDCl_3$): δ = –1.9 (+), 10.2 (–), 17.3 (–), 22.0 (+), 33.1 and 33.3 (–), 52.7 (+), 53.2 (+), 59.0 (+), 63.7 (+), 64.8 (–), 115.6 (q, *J* = 271.4 Hz), 157.4 (–), 174.5 and 174.8 (–).

*Teoc-(S)tFmcpA-Cyclo-$F_3$6 (**55a**)*: The *N*-MeZ-protected cyclohexadepsipeptide **52a** (25 mg, 25 µmol) was deprotected with 10% anisole in TFA (1.1 mL) in the dark at ambient temperature for 2 h, the residue was treated with toluene (5 mL), the solution was concentrated under reduced pressure and the residue was dried *in vacuo* at ambient temperature for 2 h. A solution of Teoc-(*S*)tFmcpA-OH **53a**, HATU (29 mg, 75 µmol) and HOAt (10 mg, 75 µmol) in CH_2Cl_2 (1.5 mL) was added at 4 °C, followed by a solution of DIEA (3 5 mg, 27 µmol) and TMP (27 mg, 225 µmol) in CH_2Cl_2 (1.5 mL) and the mixture was stirred at ambient temperature for 15 h. The reaction mixture was then diluted with diethyl ether (50 mL), and the crude product obtained after the usual aqueous work-up (GP 2) was purified by crystallization from CH_2Cl_2/pentane to give Teoc-(*S*)tFmcpA-Cyclo-$F_3$6 (**55a**) (29 mg, 99%) as a colorless solid (R_f = 0.43, acetone/hexane 1:2), which was used for the next step without any characterization.

*MOM-O-Protected [(trifluoromethylcyclopropyl)alanyl]hormaomycin (MOM-O-F_3Horm, **57a**)*: The Teoc group was cleaved off the compound **55a** (8.0 mg, 7.08 µmol) by treatment with TFA (0.6 mL) for 1 h. The mixture was concentrated under reduced pressure at 20 °C, and the residue then taken up with toluene (3 × 15 mL), which was distilled off to remove the last traces of TFA. The resulting deprotected depsipeptide was coupled with the *O*-MOM protected acid **54** (2.9 mg, 14.10 µmol) using HATU (5.4 mg, 14.20 µmol), DIEA (0.92 mg, 7.12 µmol) and TMP (5.14 mg, 42.42 µmol) in CH_2Cl_2 (1 mL) according to GP 6 within 2.5 h. The mixture was then taken up with Et_2O (20 mL), and the crude product obtained after the usual aqueous work-up (GP 2) was crystallized from CH_2Cl_2 / pentane to give the *O*-MOM protected trifluoromethylcyclopropylalanyl hormaomycin **57a** (8.0 mg, 96%, R_f = 0.36 acetone/hexanes 1:2) as a colorless glass which was used for the next step without any characterization.

*[(Trifluoromethylcyclopropyl)alanyl]hormaomycin (F_3Horm, **8a**)*: The *O*-MOM protected [(trifluoromethylcyclopropyl)alanyl]hormaomycin **57a** (8.0 mg, 6.82 µmol) was deprotected using $MgBr_2{\cdot}Et_2O$ (52 mg, 201 µmol) and EtSH (0.10 mL, 1.9 mmol) in CH_2Cl_2 (10 mL) according to GP 7 within 3 h. The mixture was taken up with EtOAc, and the crude product obtained after the usual aqueous work-up was crystallized from CH_2Cl_2 / pentane to give **2a** as a colorless solid, which was finally purified by preparative HPLC. Yield 5.5 mg (72%, 55% over 5 steps from **52a**). R_f = 0.24 acetone/hexanes 3:7; preparative HPLC: isocratic, 82% B for 30 min, t_R = 16.8 min, purity > 98%; analytical HPLC: gradient 20% → 100% B for

20 min, then isocratic 100% B for 5 min t_R = 15.3 min, purity > 98%; $[\alpha]_D^{20}$ = +20.0 (*c* = 0.1, MeOH); ^{1}H NMR (600 MHz, $CDCl_3$): δ = –0.71 to –0.63 (m, 1 H), –0.20 to –0.10 (m, 1 H), 0.23–0.32 (m, 1 H), 0.49–0.56 (m, 1 H), 0.88 (t, *J* = 7.4 Hz, 3 H), 0.95–1.01 (m, 1 H), 1.02 (d, *J* = 6.9 Hz, 3 H), 1.04–1.14 (m, 1 H), 1.17–1.35 (m, 1 H), 1.30 (d, *J* = 7.0 Hz, 3 H), 1.39 (d, *J* = 7.3 Hz, 3 H), 1.47–1.54 (m, 1 H), 1.53 (d, *J* = 6.9 Hz, 3 H), 1.55–1.62 (m, 1 H), 1.67 (dd, *J* = 6.9 Hz, 1.6 Hz, 3 H), 1.75–1.84 (m, 2 H), 1.85–1.94 (m, 3 H), 2.30–2.40 (m, 1 H), 2.88–2.91 (m, 1 H), 2.96–3.02 (m, 1 H), 3.22–3.31 (m, 2 H), 3.43–3.50 (m, 1 H), 3.62–3.70 (m, 1 H), 3.93–4.00 (m, 1 H), 4.03 [ddd, *J* = 6.8, 3.4, 3.4 Hz, 1 H), 4.26 (dd, *J* = 11.5 Hz, 6.0 Hz, 1 H), 4.38 (dd, J = 10.6 Hz, 10.6 Hz, 1 H), 4.46 (dd, *J* = 9.4 Hz, 4.5 Hz, 1 H), 4.57 (dd, *J* = 9.3 Hz, 2.3 Hz, 1 H), 4.66 (dd, *J* = 9.0, 9.0 Hz, 1 H), 5.08–5.14 (m, 1 H), 5.22–5.28 (m, 1 H), 5.40 (qd, *J* = 6.9 Hz, 2.4 Hz, 1 H), 5.58–5.65 (m, 1 H), 6.13 (d, *J* = 4.7 Hz, 1 H), 6.56 (d, *J* = 6.5 Hz, 1 H), 6.80 (d, *J* = 9.3 Hz, 1 H), 6.82 (d, *J* = 4.7 Hz, 1 H), 6.98–7.03 (m, 1 H), 7.09–7.18 (m, 5 H), 7.20–7.27 (m, 7 H), 8.05 (d, *J* = 9.1 Hz, 1 H), 9.06 (d, *J* = 9.3 Hz, 1 H); ^{13}C NMR (150.8 MHz, $CDCl_3$): δ = 10.50 (+), 13.24 (+), 13.33 (+), 14.94 (+), 16.99 (+), 17.41 (–), 17.74 (+), 20.00 (+), 21.66 (+), 24.90 (–), 26.88 (–), 33.02 (–), 35.03 (–), 35.51 (–), 36.66 (–), 37.97 (+), 39.24 (+) 41.75 (+), 50.99 (+), 51.79 (+), 52.78 (–), 54.61 (+), 54.93 (+), 58.11 (+), 59.12 (+), 59.86 (+), 60.04 (+), 61.37 (+), 69.07 (+), 103.59 (+), 109.85 (+), 119.86 (–), 121.55 (–), 126.98 (+), 127.17 (+), 127.44 (+), 127.47 (+), 127.67 (+), 128.33 (+), 128.49 (+), 128.64 (+), 141.55 (–), 142.11 (–), 159.27 (–), 168.54 (–), 168.73 (–), 169.75 (–), 170.74 (–), 171.26 (–), 171.55 (–), 172.86 (–), HRMS: M+H]$^+$ calculated 1175.48021; found 1175.47956; M+NH_4]$^+$: calculated 1192.50676; found 1192.50686; M+Na]$^+$: calculated 1197.46216; found 1197.46120.

[(Difluoromethylcyclopropyl)alanyl]hormaomycin analogue **8b**

(2R,1'R,2'R)-2-{[(9H-Fluoren-9-yl)methoxy]carbonylamino}-3-[2-(difluoromethyl)cyclopropyl] propanoic acid [Fmoc-(R)dFmcpA-OH], [(2*R*,1'*R*,2'*R*)-**41b**]: Analogous to a procedure in [5] a solution of Fmoc-OSu (459 mg, 1.36 mmol) in acetone (7 mL) was added to a

vigorously stirred solution of (2*R*,1'*R*,2'*R*)-3-(2'-difluoromethylcyclopropyl)alanine [(2*R*,1'*R*,2'*R*)-**9b**] (225 mg, 1.14 mmol) and $NaHCO_3$ (0.202 g, 2.40 mmol) in water (5 mL) (if a precipitate formed, acetone and/or water was added to obtain a homogeneous solution), and stirring was continued for an additional 3 h. Acetone was then removed under reduced pressure, and the pH of the residual aqueous solution was adjusted to 1 with aq. 1 M $KHSO_4$. The resulting emulsion was extracted with diethyl ether (30 mL), and the ethereal layer was back-extracted with 3% aq. $NaHCO_3$ (5 × 10 mL, TLC control for the completeness of the extraction was necessary). The combined aqueous fractions were washed with diethyl ether (2 × 10 mL), acidified to pH~2 with aq. 1 M $KHSO_4$, and the resulting emulsion was extracted with diethyl ether (4 × 10 mL). The organic phase was washed with aq. 1 M $KHSO_4$ (2 × 10 mL), water (3 × 10 mL), brine (2 × 5 mL), dried, filtered and concentrated under reduced pressure. The residue was triturated with cold pentane and filtered. The resulting extremely viscous oil was dried at 0.02 Torr for a prolonged time to give the target protected amino acid (2*R*,1'*R*,2'*R*)-**41b** (390 mg, 78%) as a colorless foam. R_f = 0.08 (EtOAc/hexane 1:1); m. p. (softening) 50–57 °C; $[\alpha]_D^{20}$ = –24.6 (*c* = 0.50, $CHCl_3$); ^{1}H NMR (500 MHz, $CDCl_3$): 0.38–0.44 + 0.57–0.64 + 0.79–0.86 (3 × m, 1 H), 0.91–0.97 + 1.00–1.09 (2 × m, 1 H), 1.14–1.22 + 1.26–1.34 (2 × m, 1 H), 1.35–1.53 + 1.85–1.88 (2 × m, 1 H), 1.80–1.85 (m, 1 H), 3.75–3.79 + 4.53–4.67 (2 × m, 1 H), 3.95–4.01 + 4.47–4.52 (2 × m, 1 H), 4.16–4.23 (m, 1 H), 4.36–4.47 (m, 1 H), 5.52 (d, *J* = 7.9 Hz, 0.7 H), 6.76 (d, *J* = 5.9 Hz, 0.3 H), 7.27–7.31 (m, 2 H), 7.35–7.41 (m, 2 H), 7.51 (t, *J* = 8.1 Hz, 0.6 H), 7.58 (t, *J* = 8.4 Hz, 1.4 H), 7.74 (d, *J* = 7.4 Hz, 2 H), 7.85–8.65 (bs, 1 H); ^{13}C NMR (125.7 MHz, $CDCl_3$, mixture of two rotamers): δ = 7.9 (–), 11.5 (+), 19.7 (+, q, *J* = 37.0 Hz), 34.7 (–), 47.1 (+), 53.5 and 53.8 (+), 67.1 and 67.9 (–), 120.00 (+), 124.97 (+), 125.96 (–,t, *J* = 272.4 Hz), 127.07 (+), 127.76 (+),141.3 (–), 143.5 (–), 143.8 (–), 155.8 and 156.71 (–), 175.0 and 175.8 (–); MS-ESI: (positive) *m/z* (%): 1225.6 (35, 3M–H+Na$]^+$), 824.8 (100, 2M–H+Na$]^+$), 424.2 (72, M+Na$]^+$); (negative) *m/z* (%): 801.1 (100, 2M–H$]^-$), 400.0 (9, M–H$]^-$), 178.2 (8).

Dicyclopropylmethyl (5R,8S,11S)-11-sec-butyl-5-{[(1S,2R)-2-(difluoromethyl)cyclopropyl]-methyl}-1-(9H-fluoren-9-yl)-3,6,9-trioxo-8-[(R)-1-phenylethyl]-2-oxa-4,7,10-triazadodecan-12-oate [Fmoc-(R)dFmcpA-(βMe)Phe-Ile-ODCPM] (**47b**): The dipeptide **42** (434 mg, 834 µmol) was taken up with EtOAc (20 mL) and hydrogenated over 10% Pd/C (250 mg) under ambient pressure of hydrogen for 2 h. The reaction mixture was filtered through a pad of Celite®, and the solution was concentrated under reduced pressure to give the deprotected dipeptide, which was directly used for the coupling with Fmoc-(*R*)dFmcpA-OH -(2*R*,1'*R*,2'*R*)-**41b** (360 mg, 860 µmol), using EDC (172 mg, 896 µmol), HOAt (120 mg, 883 µmol) and TMP (310 µL, 2.5 mmol) according to GP 2. During the reaction, a colorless precipitate appeared. The mixture was diluted with diethyl ether (50 mL), stirred for 30 min and filtered to give the crude product (1st crop, 173 mg after drying *in vacuo*). The filtrate was concentrated under reduced pressure at ambient temperature and diluted with diethyl ether (20 mL), and this solution was subjected to the usual aqueous work-up according to GP 2 to give another portion of crude product (2nd crop, 112 mg after drying *in vacuo*). The combined crude product was recrystallized from THF/hexane, the resulting off-white solid was dissolved in chloroform (50 mL), and the solution was subjected to the usual aqueous work-up according to GP 2 to give the pure tripeptide **47b** as a colorless solid (253 mg, 65%). R_f = 0.52; EtOAc/hexane 2:3; m. p. 151–155 °C; $[\alpha]_D^{20}$ = +6.8 (*c* = 0.50, DMSO)); ^{1}H NMR (500 MHz, $CDCl_3$): δ = 0.28–0.40 (m, 4 H), 0.43–0.53 (m, 2 H), 0.57–0.66 (m, 3 H), 0.88 (d, *J* = 6.7 Hz, 3 H), 0.90 (t, *J* = 7.4 Hz, 3 H), 0.94–1.00 (m, 1 H), 1.03–1.13 (m, 2 H), 1.13–1.22 (m, 2 H), 1.30–1.50 (m, 2 H), 1.41 (d, *J* = 6.8 Hz, 3 H), 1.58–1.70 (m, 1 H), 1.81–1.97 (m, 2 H), 3.28–3.38 (m, 1 H), 3.86 (t, *J* = 8.4 Hz, 1 H), 4.28 (t, *J* = 6.9 Hz, 1 H), 4.39–4.48 (m, 3 H), 4.48–4.57 (m, 1 H), 4.76 (t, *J* = 8.0 Hz, 1 H), 5.76 (d, *J* = 7.7 Hz, 1 H), 6.40 (d, *J* = 7.5 Hz, 1 H), 7.11 (d, *J* = 8.1 Hz, 1 H), 7.20–7.34 (m, 5 H), 7.35 (t, *J* = 7.4 Hz, 2 H), 7.46 (t, *J* = 7.4 Hz, 2 H), 7.60 (t, *J* = 8.0 Hz, 2 H), 7.79 (d, *J* = 7.5 Hz, 2 H); ^{13}C NMR (125.7 MHz, [D_6]-DMSO): δ = 2.6 (–), 2.9 (–), 8.2 (–), 11.6 (+), 14.4 (+), 14.7 (+), 15.1 (+), 15.2 (+), 16.9 (+), 19.70 (+, t, *J* = 37.1 Hz), 25.1 (–), 35.1 (–), 38.1 (+), 42.1 (+), 47.1 (+),

54.7 (+), 56.5 (+), 58.8 (+), 67.2 (–), 83.4 (+), 119.9 (+), 125.0 (+), 127.1 (+), 127.6 (+), 127.7 (+), 128.5 (+), 141.2 (–), 141.3 (–), 141.7 (–), 143.8 (–), 143.8 (–), 156.0 (–), 169.6 (–), 170.6 (–), 170.9 (–); HRMS: calculated for $C_{45}H_{54}F_2N_3O_6$ (M+H]$^+$) 770.3975, found 770.3976.

Dicyclopropylmethyl (5S,8R,11S,14S)-14-sec-butyl-8-{[(1S,2R)-2-(difluoromethyl)cyclopropyl]methyl}-1-(9H-fluoren-9-yl)-3,6,9,12-tetraoxo-5,11-bis[(R)-1-phenylethyl]-2-oxa-4,7,10,13-tetraazapentadecan-15-oate [Fmoc-(βMe)Phe-(R)dFmcpA-(βMe)Phe-Ile-ODCPM] (**49b**): The tripeptide **47b** (394 mg, 500 µmol) was deprotected according to GP 1, and the resulting *C*-protected tripeptide was then directly coupled with Fmoc-*(βMe)Phe*-OH **46** (211 mg, 525 µmol) according to GP 2 using EDC (99 mg, 518 µmol), HOAt (70 mg, 512 µmol) and TMP (175 mg, 1440 µmol) in CH_2Cl_2 (3 mL). After 16 h, the reaction mixture was diluted with chloroform (50 mL) and subjected to the usual aqueous work-up according to GP 2 to give the crude tetrapeptide, which was twice recrystallized from THF/hexane to give the pure target tetrapeptide **49b** as an off-white solid (440 mg, 81%). R_f = 0.29; $CHCl_3$/MeOH 70:1; m. p. 210–215 °C (decomp.); $[\alpha]_D^{20}$ = –16.0 (*c* = 0.50 in THF); ^{1}H NMR (500 MHz, [D$_8$]-THF): δ = 0.23–0.32 (m, 4 H), 0.32–0.39 (m, 2 H), 0.40–0.55 (m, 4 H), 0.82 (d, *J* = 7.0 Hz, 3 H), 0.83 (d, *J* = 7.3 Hz, 3 H), 0.82–0.91 (m, 1 H), 0.94–1.05 (m, 3 H), 1.08–1.19 (m, 2 H), 1.22 (d, *J* = 7.1 Hz, 3 H), 1.32 (d, *J* = 7.0 Hz, 3 H), 1.36–1.47 (m, 2 H), 2.63 (bs, 1 H), 3.17 (dq, *J* = 9.2 Hz, 7.2 Hz, 1 H), 3.29 (dq, *J* = 7.2 Hz, 7.1 Hz, 1 H), 3.82 (t, *J* = 8.28, 1 H), 4.18–4.25 (m, 2 H), 4.25–4.31 (m, 1 H), 4.33–4.40 (m, 2 H), 4.41–4.48 (m, 1 H), 4.65–4.71 (m, 1 H), 5.40 (td, *J* = 57.5 Hz, 4.8 Hz, 1 H), 7.02–7.08 (m, 1 H), 7.09–7.29 (m, 12 H), 7.30–7.36 (m, 3 H), 7.49 (t, *J* = 9.3 Hz, 2 H), 7.63 (d, *J* = 7.5 Hz, 1 H), 7.67 (d, *J* = 7.7 Hz, 1 H), 7.76 (d, *J* = 7.6 Hz, 2 H); ^{13}C NMR (125.7 MHz, [D$_8$]-THF): δ =2.8 (–), 2.98 (–), 3.04 (–), 3.2 (–), 7.7 (–), 11.7 (+), 12.0 (+, t, *J* = 4.2 Hz), 15.1 (+), 15.4 (+), 15.6 (+), 17.0 (+), 18.0 (+), 21.1 (+, t, *J* = 27.1 Hz), 25.8 (–), 36.0 (–), 38.5 (+), 42.0 (+), 43.3 (+), 48.1 (+), 53.4 (+), 56.9 (+), 58.8 (+), 61.6 (+), 67.3 (–), 82.8 (+), 118.3 (+, t, *J* = 236.9 Hz), 120.3 (+), 120.4 (+), 125.9 (+), 126.0 (+), 126.8 (+), 127.2 (+), 127.6 (+),

127.6 (+), 128.1 (+), 128.5 (+), 128.6 (+), 128.7 (+), 128.8 (+), 142.0 (–), 142.1 (–), 143.9 (–), 144.1 (–), 145.0 (–), 145.1 (–), 157.3 (–), 170.6 (–), 171.5 (–), 171.6 (–);MS-ESI: (positive) *m/z* (%) 1681.9 (5, 2M+H]$^+$), 953.5 (20, M+Na]$^+$), 931.5 (100, M+H]$^+$); HRMS: calculated for $C_{55}H_{65}F_2N_4O_7$ (M+H]$^+$): 931.4816; found: 931.4809.

(5R,8S,11R,14S,17S)-5-[(R)-1-{(2S,4R)-1-(tert-Butoxycarbonyl)-4-[(Z)-prop-1-enyl]pyrrolidine-2-carbonyloxy}ethyl]-17-sec-butyl-11-{[(1S,2R)-2-(difluoromethyl)cyclopropyl]methyl}-3,6,9,12,15-pentaoxo-8,14-bis[(R)-1-phenylethyl]-1-p-tolyl-2-oxa-4,7,10,13,16-pentaaza-octadecan-18-oic acid [Boc-(4-Pe)Pro-[MeZ-a-Thr]-(βMe)Phe-(S)dFmcpA*-(βMe)Phe*-Ile-*ODCPM]* (**51b**): The tetrapeptide **49b** (200 mg, 215 μmol) was *N*-deprotected according to GP 1 with diethylamine (5 mL) and THF (5 mL), the crude product taken up with anhydrous CH_2Cl_2 (5 mL), a solution of the ester acid **45** (119 mg, 235 μmol), HATU (98 mg, 256 μmol) and HOAt (32 mg, 235μmol) in CH_2Cl_2 (3 mL) were added, and the reaction mixture was cooled to 4 °C. After this, a solution of DIEA (30 mg, 225 μmol) and TMP (77 mg, 635 μmol) in CH_2Cl_2 (2 mL) was added at the same temperature within 5 min. The temperature was allowed to reach 20 °C, and stirring was continued for an additional 15 h. After aqueous work-up according to GP 2 and two recrystallizations from EtOAc/hexane (1:2), the crude hexadepsipeptide was finally purified by column chromatography (silica gel). All impurities were eluted with EtOAc/hexane (1:1), and the product was eluted with methanol. After solvent evaporation under reduced pressure the *C*-deprotected hexadepsipeptide **51b** (236 mg, 90%) was obtained as a colorless solid. R_f = 0.46 (THF); $[\alpha]_D^{20}$ = –18.5 (*c* = 0.2, THF); ^{1}H NMR (600 MHz, [D$_8$]-THF): δ = 0.23–0.30 (m, 1 H), 0.30–0.37 (m, 0.5 H), 0.37–0.43 (m, 0.5 H), 0.44–0.53 (m, 1 H), 0.79–1.02 (m, 2 H) 0.84 (d, *J* = 7.3 Hz, 3 H), 0.86 (*d*, *J* = 7.1 Hz, 3 H), 1.13 (d, *J* = 6.1 Hz, 2 H), 1.16 (d, *J* = 5.9 Hz, 2 H), 1.20 (t, *J* = 5.4 Hz, 2 H), 1.28 (t, *J* = 6.0 Hz, 2 H), 1.37 + 1.39 (2 s, 9 H), 1.42–1.53 (m, 2 H), 1.64 (t, *J* = 6.7 Hz, 2 H), 1.78–1.87 (m, 1 H), 2.26–2.33 (m, 1 H), 2.27 (s, 3 H), 2.91–3.60 (bs, 1 H), 2.93 (t, *J* = 10.3 Hz, 1 H), 2.99 (t, *J* = 10.2 Hz, 1 H), 3.05 (q, *J* = 8.6 Hz, 1 H), 3.16 (q,

J = 8.7 Hz, 1 H), 3.17–3.28 (m, 2 H), 3.35–3.46 (m, 2 H), 3.64 (dd, *J* = 10.0 Hz, 7.5 Hz, 1 H), 4.07–4.35 (m, 3 H), 4.55–4.72 (m, 3 H), 4.91 (d, *J* = 12.3 Hz, 1 H), 5.04 (dd, *J* = 24.1 Hz, 12.2 Hz, 1 H), 5.19–5.36 (m, 3 H), 5.45–5.53 (m, 1 H), 6.90 (d, *J* = 8.1 Hz, 0.6 H), 6.97 (d, *J* = 8.7 Hz, 0.4 H), 7.03–7.29 (m, 14 H), 7.40 (d, *J* = 7.0 Hz, 0.5 H), 7.45 (d, *J* = 6.4 Hz, 0.5 H), 7.53–7.64 (m, 2 H), 7.93 (d, *J* = 5.4 Hz, 0.6 H), 7.97 (d, *J* = 6.1 Hz, 0.4 H); ^{13}C NMR (125.7 MHz, [D$_8$]-THF): δ = 7.8 (–), 11.7 (+), 11.96 (+), 12.02 (+), 13.0 (+), 13.1 (+), 15.8 (+), 21.00 (+), 21.01 (+), 25.6 (+), 25.9 (–), 28.4 (+), 28.5 (+), 35.1 (–), 35.4 (–), 36.4 (+), 36.6 (–), 37.1 (+), 37.7 (–), 38.1 (+), 41.3 (+), 41.6 (+), 42.4 (+), 52.0 (–), 52.5 (–), 53.8 (+), 53.9 (+), 60.2 (+), 67.7 (+), 79.4 (–), 80.0 (–), 116.6 (+), 118.5 (+), 120.4 (+), 126.4 (+), 126.5 (+), 126.7 (+), 126.8 (+), 127.1 (+), 127.2 (+), 128.37 (+), 128.40 (+), 128.57 (+), 128.59 (+), 128.8 (+), 128.9 (+), 129.4 (+), 129.5 (+), 130.7 (+), 130.9 (+), 134.9 (–), 137.9 (–), 143.9 (–), 144.0 (–), 153.4 (–), 154.5 (–), 157.0 (–), 157.2 (–), 169.91 (–), 169.94 (–), 170.87 (–), 170.92 (–), 171.4 (–), 171.5 (–), 171.9 (–), 172.2 (–), 172.4 (–); MS-ESI: (positive) *m/z* (%) 1123.6 (100, M+Na^{+}), 1101.6 (100, M+H^{+}); HRMS: calculated for $C_{59}H_{79}F_2N_6O_{12}$ (100, M+H^{+}): 1101.5724; found: 1101.5700

4-Methylbenzyl (3R,4R,7S,10R,13S,16S,20R,21aS)-16-sec-butyl-10-{[(1S,2R)-2-(difluoromethyl)cyclopropyl]methy})-3-methyl-1,5,8,11,14,17-hexaoxo-7,13-bis[(R)-1-phenylethyl]-20-[(Z)-prop-1-enyl]icosahydropyrrolo[2,1-c][1,4,7,10,13,16]oxapentaazacyclononadecin-4-ylcarbamate [N-MeZ-protected cyclohexadepsipeptide Cyclo-F$_2$6-MeZ] (**52b**): The hexadepsipeptide **51b** (136 mg, 122 µmol) was deprotected at the termini by treatment with a 2 M HCl solution in ethyl acetate (5 mL). The reaction mixture was stirred for 20 min in the dark (aluminum foil jacket) at ambient temperature, and all volatiles were removed under reduced pressure without any heating. The residue was triturated with anhydrous diethyl ether (10 mL) to give the hydrochloride of the deprotected material as a colorless solid. HRMS [calculated for $C_{54}H_{71}F_2N_6O_{10}$ (M+H^{+}): 1001.5200; found: 1001.5186. The ends-deprotected hexadepsipeptide, HATU (54 mg, 142 µmol) and HOAt (16 mg, 121 µmol) were dissolved in cold (4 °C, internal temperature) anhydrous CH_2Cl_2 (1,5 L), and a solution of DIEA (46 mg,

354 µmol) in CH_2Cl_2 (100 mL) was added dropwise within 1 h. The cooling bath was removed, and the mixture was stirred at ambient temperature for 2 h. The mixture was cooled again to 4 °C (internal temperature), then a second portion each of HATU (54 mg, 142 µmol) and HOAt (16 mg, 121 µmol) were added, followed with dropwise addition of a solution of DIEA (46 mg, 354 µmol) in CH_2Cl_2 (100 mL) within 1 h. The cooling bath was removed, and the mixture was stirred at ambient temperature for 18 h. The mixture was concentrated under reduced pressure, subjected to aqueous work-up according to GP 2 to give the crude protected cyclohexadepsipeptide (118 mg, 98%) which was finally purified by HPLC to give pure product **52b** (72 mg, 60%). Preparative HPLC: isocratic, 60% B for 8 min, then gradient 60%→100% B for 6 min, then isocratic 100% B for 11 min, t_R = 17.2 min, purity > 98%; analytical HPLC: isocratic 60% B for 10 min, then gradient 60% → 100% B for 20 min, then isocratic 100% B for 15 min t_R = 27.8 min, purity > 98%; $[\alpha]_D^{20}$ = –20.0 (*c* = 0.15, THF); ^{1}H NMR (600 MHz, [D$_8$]-THF): δ = –0.05 to 0.02 (m, 1 H), 0.12–0.18 (m, 1 H), 0.27–0.34 (m, 1 H), 0.34–0.39 (m, 1 H), 0.74 (d, *J* = 6.6 Hz, 3 H), 0.79 (t, *J* = 7.5 Hz, 3 H), 1.10–1.24 (m, 2 H), 1.20 (d, *J* = 7.2, 3 H) 1.28 (d, *J* = 7.2 Hz, 3 H), 1.36–1.47 (m, 1 H), 1.49 (d, *J* = 6.9 Hz, 3 H), 1.60–1.70 (m, 1 H), 1.66 (dd, *J* = 1.7 Hz, 6.9 Hz, 3 H), 1.95–2.04 (m, 1 H), 2.12–2.18 (m, 1 H), 2.30 (s, 3 H), 2.88–2.96 (m, 1 H), 3.12–3.17 (m, 1 H), 3.22–3.31 (m, 1 H), 3.45–3.51 (m, 1 H), 3.70–3.76 (m, 1 H), 3.83–3.88 (m, 1 H), 4.17–4.23 (m, 1 H), 4.40–4.46 (m, 1 H), 4.52–4.70 (m, 4 H), 4.93–4.99 (m, 1 H), 4.99–5.02, 5.07–5.14 (2×m, 2 H), 5.27–5.33 (m, 1 H), 5.36–5.41 (m, 1 H), 5.51–5.58 (m, 1 H), 6.74–6.82 (m, 1 H), 7.07–7.22 (m, 9 H), 7.23–7.29 (m, 5 H), 7.30–7.40 (m, 1 H), 7.64 (d, *J* = 8.5 Hz, 1 H), 7.69 (d, *J* = 7.8 Hz, 1 H), 7.80–7.90, 8.18–8.22 (2×m, 1 H); ^{13}C NMR (125.7 MHz, [D$_8$]-THF): δ = 7.9 (–), 8.0 (–), 10.4 (+), 11.5 (+), 11.6 (+), 13.15 (+), 13.2 (+), 15.3 (+), 18.5 (+), 18.6 (+),20.5 (+), 21.1 (+), 21.4 (+, t, *J* = 27.5 Hz), 30.4 (–), 31.1 (–), 34.9 (–), 35.9 (–), 36.1 (+), 37.3 (+), 39.2 (+), 46.3 (+), 52.5 (–), 54.8 (+), 55.0 (+), 58.88 (+), 60.3 (+), 60.7 (+), 61.7 (+), 73.0 (+), 118.7 (+, t, *J* = 236.3 Hz), 126.8 (+), 126.9 (+), 127.0 (+), 127.6 (+), 128.1 (+), 128.47 (+), 128.50 (+), 128.6 (+), 128.65 (+), 128.72 (+), 128.9 (+), 129.5 (+),

129.7 (+), 130.7 (+), 135.4 (–), 137.7 (–), 143.8 (–), 144.8 (–), 156.6 (–), 169.0 (–), 170.94 (–), 171.04 (–), 171.4 (–), 172.9 (–); MS–ESI (positive): 1988.1 (23, 2M+H+Na]$^+$), 1005.6 (100, M+Na]$^+$); (negative): 981.4 (100, M–H]$^-$).

N-Teoc-(2S,1'S,2'R)-3-(2'-Difluoromethylcyclopropyl)alanine [Teoc-(S)dFmcA-OH], [(2*S*,1'*S*,2'*R*)-**53b**]: A solution of TeocOSu (59 mg, 228 µmol) in acetone (1 mL) was added to a vigorously stirred solution of (2*S*,1'*S*,2'*R*)-3-(2'-difluoromethylcyclopropyl)alanine [(2*S*,1'*S*,2'*R*)-**9b**] (34 mg, 190 µmol) and $NaHCO_3$ (34 mg, 396 µmol) in water (1 mL) (if an emulsion formed, acetone and/or water was added to obtain a homogeneous solution), and stirring was continued for another 2 h. *N,N*-Dimethylaminopropylamine (10 µL, 7.5 mg, 73 µmol) was then added. After an additional 10 min, acetone was removed under reduced pressure, and the pH of the residual aqueous solution was adjusted to 2–3 with aq. 1 M $KHSO_4$. The resulting emulsion was extracted with diethyl ether (50 mL), and the ethereal layer was washed with aq. 1 M $KHSO_4$ (2 × 10 mL), water (3 × 10 mL), brine (2 × 5 mL), dried over $MgSO_4$, filtered and concentrated under reduced pressure. The residual oil was dried overnight *in vacuo* to give (2*S*,1'*S*,2'*R*)-**53b** (41 mg, 67%) as the glass-like product, $R_f = 0.26$ (EtOAc/hexane 1:3, +2% AcOH); $[\alpha]_D^{20} = +20.2$ ($c = 0.26$, $CHCl_3$); ^{1}H NMR (250 MHz, $CDCl_3$, as the cyclohexylammonium salt): δ = 0.01 (s, 9 H), 0.38–0.57 (m, 1 H), 0.65–0.80 (m, 1 H), 0.85–1.49 (m, 9 H), 1.50–1.85 (m, 5 H), 1.85–2.03 (m, 2 H), 2.72–3.00 (m, 1 H), 3.89–4.22 (m, 3 H), 5.50 (td, *J* = 57.6 Hz, 4.1 Hz, 1 H), 5.71 (d, *J* = 7.3 Hz, 1 H), 7.35 (bs, 3 H); ^{13}C NMR (62.9 MHz, $CDCl_3$, major of two rotamers): δ = –1.6 (+), 7.2 (–), 110.6 (–), 17.6 (+), 20.4 (+, t, J = 24.0 Hz), 35.2 (–), 53.5 (+), 63.7 (–), 116.8 (+, t, J = 237.5 Hz), 156.3 (–), 176.3 (–).

2-(Trimethylsilyl)ethyl 1-[(3R,4R,7S,10R,13S,16S,20R,21aS)-16-sec-butyl-10-{[(1S,2R)-2-(difluoromethyl)cyclopropyl]methyl}-3-methyl-1,5,8,11,14,17-hexaoxo-7,13-bis[(R)-1-phenylethyl]-20-[(Z)-prop-1-enyl]icosahydropyrrolo[2,1-c][1,4,7,10,13,16]oxapentaazacyclononadecin-4-ylamino]-3-[(1S,2R)-2-(difluoromethyl)cyclopropyl]-1-oxopropan-2-ylcarba-

mate [Teoc-(S)dFmcpA-Cyclo-$F_2$6] (**55b**): *N*-MeZ-protected cyclohexadepsipeptide **60b** (62 mg, 63 µmol) was deprotected with 10% anisole in TFA (4 mL) in the dark at ambient temperature within 2 h, the residue was treated with toluene (5 mL), the solution was concentrated under reduced pressure, and the residue was dried *in vacuo* at ambient temperature for 2 h. A solution of Teoc-(*S*)dFmcpA-OH **53b** (31 mg, 96 µmol), HATU (72 mg, 189 µmol) and HOAt (26 mg, 190 µmol) in CH_2Cl_2 (3 mL) was added at 4 °C, followed by a solution of DIEA (8.4 mg, 65 µmol) and TMP (69 mg, 568 µmol) in CH_2Cl_2 (5.5 mL), and the mixture was stirred at ambient temperature for 15 h. The reaction mixture was then diluted with diethyl ether (50 mL), and the crude product obtained after the usual aqueous work-up (GP 2) was purified by column chromatography (silica gel, eluting with EtOAc/hexane 1:1) to give the Teoc-(*S*)dFmcpA-Cyclo-$F_2$6 (**55b**) (53 mg, 46.5 µmol, 74%) as a colorless solid. R_f = 0.34, EtOAc/hexane 1:1; $[\alpha]_D^{20}$ = –7.0 (*c* = 0.3, $CHCl_3$); ^{1}H NMR (500 MHz, $CDCl_3$):δ = –0.15 to –0.02 (m, 2 H), 0.01–0.02 (m, 1 H), 0.05 (s, 9 H), 0.06–0.10 (m, 1 H), 0.28–0.42 (m, 2 H), 0.43–0.58 (m, 2 H), 0.78–0.88 (m, 2 H), 0.90 (t, *J* = 7.3 Hz, 3 H), 0.96 (d, *J* = 6.8 Hz, 3 H), 1.02 (t, *J* = 8.5Hz, 2 H), 1.07–1.20 (m, 3 H), 1.26 (d, *J* = 7.1 Hz, 3 H), 1.31 (d, *J* = 7.2 Hz, 3 H), 1.54 (d, *J* = 6.8 Hz, 3 H), 1.63 (d, *J* = 6.8 Hz, 3 H), 1.71 (dd, *J* = 11.8 Hz, 23.4 Hz, 1H), 1.86–1.95 (m, 1 H), 2.15–2.35 (m, 3 H), 3.11–3.27 (m, 1 H), 3.23 (t, *J* = 9.6 Hz, 1 H), 3.62–3.69 (m, 1 H), 3.71–3.82 (m, 1 H), 4.02–4.18 (m, 3 H), 4.20–4.30 (m, 1 H), 4.34–4.41 (m, 1 H), 4.48–4.57 (m, 1 H), 4.58–4.70 (m, 2 H), 4.74 (d, *J* = 8.9 Hz, 1 H), 5.17 (td, *J* = 60 Hz, 5.0 Hz, 1H), 5.19–5.25 (m, 1 H), 5.27–5.34 (m, 1 H), 5.47 (td, *J* = 55 Hz, 4.6 Hz, 1 H), 5.53–5.61 (d, *J* = 8.9 Hz, 1 H), 6.64 (d, *J* = 8.5 Hz, 1 H), 7.00–7.40 (m, 14H), 8.16 (s, 1 H); ^{13}C NMR (125.7 MHz, $CDCl_3$): δ = –1.5 (+), 7.0 (–, t, *J* = 2.6 Hz), 7.4 (–, t, *J* = 4.2 Hz), 10.1 (+), 10.4 (+), 10.6 (+), 13.27 (+), 13.5 (+), 15.3 (+), 17.6 (–), 17.9 (+), 18.3 (+), 20.3 (+), 20.6 (+), 20.8 (+),24.8 (–), 34.7 (–), 35.5 (–), 36.51(+), 37.1 (+), 39.2 (+), 43.75 (+), 52.6 (–), 53.4 (+), 54.0 (+), 54.7 (+), 55.9 (+), 59.05 (+), 59.7 (+), 61.0 (+), 63.4 (–), 71.5 (+), 117.0 (+, t, *J* = 237.3 Hz), 117.2 (+, t, *J* = 237.7 Hz), 126.8 (+), 126.9 (+), 127.2 (+), 127.6 (+), 127.8 (+), 128.0 (+), 128.4 (+),

128.58 (+), 128.63 (+),141.8 (–),142.5 (–), 156.3 (–), 168.4 (–), 170.1 (–), 170.8 (–), 171.2 (–), 171.3 (–), 171.9 (–); MS-ESI: (positive) *m/z* (%) 1162.7 (100, M+Na]$^+$), (negative) *m/z* (%) 1138.6 (100, M–H]$^-$).

N-(1-[(3R,4R,7S,10R,13S,16S,20R,21aS)-16-sec-butyl-10-([(1S,2R)-2-(difluoromethyl)cyclopropyl]methyl}-3-methyl-1,5,8,11,14,17-hexaoxo-7,13-bis[(R)-1-phenylethyl]-20-[(Z)-prop-1-enyl]icosahydropyrrolo[2,1-c][1,4,7,10,13,16]oxapentaazacyclononadecin-4-ylamino]-3-[(1S,2R)-2-(difluoromethyl)cyclopropyl]-1-oxopropan-2-yl)-5-chloro-1-(methoxymethoxy)-1H-pyrrole-2-carboxamide [MOM-O-Protected [(difluoromethylcyclopropyl)alanyl]hormaomycin, MOM-O-F$_2$Horm], (**57b**): The Teoc group in the compound **55b** (8.0 mg, 7.08 µmol) was cleaved off by stirring with TFA (0.6 mL) for 1 h. The reaction mixture was concentrated under reduced pressure at 20 °C and then taken up with toluene (3 × 15 mL), which was distilled off to remove the last traces of TFA. The resulting deprotected depsipeptide was coupled with the *O*-MOM protected acid **22** (2.9 mg, 14.10 µmol) using HATU (5.4 mg, 14.20 µmol), DIEA (0.92 mg, 7.12 µmol) and TMP (5.14 mg, 42.42 µmol) in CH_2Cl_2 (1 mL) according to GP 6 for 2.5 h. The mixture was then taken up with Et_2O (20 mL), and the crude product obtained after the usual aqueous work-up (GP 2) was crystallized from CH_2Cl_2/pentane to give *O*-MOM protected [(difluoromethylcyclopropyl)alanyl]hormaomycin **57b** (8.0 mg, 90%) as a colorless glass, R_f = 0.36 acetone/hexanes 1:2. ^{1}H NMR (500 MHz, $CDCl_3$): δ = –0.46 to –0.36 (m, 2H), 0.14–0.27 (m, 2H), 0.44–0.56 (m, 2H), 0.79–0.93 (m, 3H), 0.88 (t, *J* = 7.3 Hz, 3H), 1.00 (d, *J* = 6.8 Hz, 3H), 1.04–1.16 (m, 1H), 1.19–1.29 (m, 2H), 1.27 (d, *J* = 7.1 Hz, 3H), 1.36 (d, *J* = 7.2 Hz, 3H), 1.55 (d, *J* = 6.9 Hz, 3H), 1.64 (d, *J* = 6.9Hz, 3H), 1.69–1.83 (m, 3H), 1.84–1.96 (m, 1H), 2.30–2.39 (m, 1H), 2.79–2.90 (m, 1H), 3.03 (s, 3H), 3.19–3.31 (m, 2H), 3.64–3.71 (m, 1H), 3.72 (s, 3H), 3.81–3.89 (m, 1H), 3.90–4.00 (m, 1H), 4.20–4.30 (m, 2H), 4.61–4.74 (m, 3H), 5.03 (td, *J* = 55.0 Hz, 5.0 Hz, 1H), 5.07–5.15 (m, 1H), 5.21–5.35 (m, 2H), 5.40–5.49 (m, 1H), 5.50 (s, 2H), 5.51–5.60 (m, 2H), 5.60–5.68 (m, 1H), 6.13 (d, *J* = 4.7Hz, 1H), 6.76–6.88 (m, 2H), 7.01–7.11 (m, 3H), 7.11–7.19 (m, 3H), 7.19–7.43 (m, 5H), 7.57 (d, *J* = 9.5Hz, 1H), 8.88 (d, *J* = 9.5Hz, 1H); ^{13}C NMR

(125.7 MHz, $CDCl_3$): δ = 6.78 (–, t, *J* = 3.4 Hz), 8.08 (–, t, *J* = 4.2 Hz), 10.11 (+, t, *J* = 4.4 Hz), 10.57 (+), 10.79 (+, t, *J* = 4.7 Hz), 13.11 (+), 13.35 (+), 14.19 (+), 15.27 (+), 17.34 (+), 18.15 (+), 19.91 (+, t, *J* = 26.9 Hz), 20.44 (+, t, *J* = 27.8 Hz), 24.29 (–), 26.79 (–), 29.96 (–), 35.45 (–), 36.12 (–), 36.87 (+), 38.36 (+), 39.45 (+), 43.43 (+), 50.59 (+), 52.65 (+), 54.77 (+), 59.32 (+), 59.54 (+), 59.89 (+), 61.54 (+), 70.07 (+), 104.24 (–), 106.15 (–), 111.11 (–), 116.61 (+, t, *J* = 237.5 Hz), 117.71 (+, t, *J* = 237.8 Hz), 119.43 (+), 121.88 (+), 126.87 (+), 127.21 (+), 127.29 (+), 127.48 (+), 127.55 (+), 128.32 (+), 128.43 (+), 128.69 (+), 142.21 (–), 142.45 (–), 158.31 (–), 168.98 (–), 169.77 (–), 170.31 (–), 170.79 (–), 171.33 (–), 171.42 (–), 171.79 (–); MS-ESI: (positive) *m/z* (%) 1205.5 (100, $[M+Na]^+$), (negative) *m/z* (%) 1181.6 (100, $[M–H]^-$).

*[(Difluoromethylcyclopropyl)alanyl]hormaomycin analogue (F_2Horm, **8b**)*: The *O*-MOM protected [(difluoromethylcyclopropyl)alanyl]hormaomycin **57b** (35 mg, 29.5 µmol) was deprotected using $MgBr_2•Et_2O$ (204 mg, 788 µmol) and EtSH (50 µL, 0.7 mmol) in CH_2Cl_2 (17 mL) according to GP 7 for 3 h. The mixture was taken up with EtOAc, and the crude product obtained after the usual aqueous work-up was crystallized from CH_2Cl_2/pentane to give **8b** (33 mg, 68%) as a colorless solid, which was finally purified by preparative HPLC. R_f = 0.24 acetone/hexanes 3:7; preparative HPLC: isocratic, 82% MeCN for 25 min, t_R = 15.4 min, purity > 98%; analytical HPLC: isocratic 60% MeCN for 10 min, then gradient 60% → 100% MeCN for 20 min, then isocratic 100% MeCN for 15 min t_R = 28.8 min, purity > 98%; $[\alpha]_D^{20}$ = +20.2 (*c* = 0.1, MeOH); ^{1}H NMR (500 Mhz, $CDCl_3$): δ = –0.47 to –0.37 (m, 2H), 0.15–0.26 (m, 2H), 0.45–0.55 (m, 2H), 0.80–0.92 (m, 3H), 0.87 (t, *J* = 7.3 Hz, 3H), 1.01 (d, *J* = 6.8 Hz, 3H), 1.05–1.15 (m, 1H), 1.26 (d, *J* = 7.1 Hz, 3H), 1.37 (d, *J* = 7.2 Hz, 3H), 1.56 (d, *J* = 6.9 Hz, 3H), 1.66 (d, *J* = 6.9Hz, 3H), 1.70–1.82 (m, 3H), 1.85–1.95 (m, 1H), 2.31–2.38 (m, 1H), 2.80–2.89 (m, 1H), 3.20–3.30 (m, 2H), 3.65–3.70 (m, 1H), 3.73 (s, 3H), 3.82–3.88 (m, 1H), 3.91–3.99 (m, 1H), 4.21–4.29 (m, 2H), 4.62–4.73 (m, 3H), 5.05 (td, *J* = 55.0 Hz, 5.0 Hz, 1H), 5.08–5.14 (m, 1H), 5.22–5.27 (m, 1H), 5.30–5.37 (m, 1H), 5.43–

5.48 (m, 1H), 5.52–5.59 (m, 2H), 5.60–5.67 (m, 1H), 6.12 (d, J = 4.7Hz, 1H), 6.77–6.87 (m, 2H), 7.02–7.10 (m, 3H), 7.11–7.17 (m, 3H), 7.20–7.28 (m, 3H), 7.24–7.43 (m, 2H), 7.55 (d, J = 9.5Hz, 1H), 8.86 (d, J = 9.5Hz, 1H); ^{13}C NMR (125.7 MHz, $CDCl_3$): δ = 6.82 (–, t, J = 3.4 Hz), 8.18 (–, t, J = 4.2 Hz), 10.01 (+, t, J = 4.4 Hz), 10.72 (+), 10.94 (+, t, J = 4.7 Hz), 13.31 (+), 13.50 (+), 14.09 (+), 15.07 (+), 17.45 (+), 18.55 (+), 19.69 (+, t, J = 26.9 Hz), 20.24 (+, t, J = 27.8 Hz), 24.94 (–), 26.90 (–), 29.68 (–), 35.51 (–), 36.00 (–), 36.72 (+), 38.62 (+), 39.54 (+), 43.14 (+), 50.96 (+), 54.70 (+), 59.26 (+), 59.41 (+), 59.97 (+), 61.44 (+), 70.11 (+), 106.05 (–), 111.15 (–), 116.70 (+, t, J = 237.5 Hz), 117.51 (+, t, J = 237.8 Hz), 119.36 (+), 121.83 (+), 126.72 (+), 126.96 (+), 127.31 (+), 127.51 (+), 127.53 (+), 128.25 (+), 128.37 (+), 128.64 (+), 142.14 (–), 142.54 (–), 158.11 (–), 168.84 (–), 169.99 (–), 170.17 (–), 170.92 (–), 171.32 (–), 171.43 (–), 171.90 (–); MS-ESI: (positive) *m/z* (%) 1161.5 (100, M+Na]$^+$), (negative) *m/z* (%) 1137.5 (100, M–H]$^-$).

(Monofluoromethylcyclopropyl)alanylhormaomycin analogue

N-Fmoc-(2R,1'R,2'R)-3-(2'-Fluoromethylcyclopropyl)alanine [*Fmoc-(R)mFmcpA-OH*, (2*R*,1'*R*,2'*R*)-**41c**]: Analogous to a procedure in [5] a solution of Fmoc-OSu (459 mg, 1.36 mmol) in acetone (7 mL) was added to a vigorously stirred solution of (2*R*,1'*R*,2'*R*)-3-(2'-fluoromethylcyclopropyl)alanine [(2*R*,1'*R*,2'*R*)-**9c**] (225 mg, 1.14 mmol) and $NaHCO_3$ (0.202 g, 2.40 mmol) in water (5 mL) (if a precipitate formed, acetone and/or water was added to obtain a homogeneous solution), and stirring was continued for an additional 3 h. Acetone was then removed under reduced pressure, and the pH of the residual aqueous solution was adjusted to 1 with aq. 1 M $KHSO_4$. The resulting emulsion was extracted with diethyl ether (30 mL), and the ethereal layer was back-extracted with aq. 3% $NaHCO_3$ (5 × 10 mL). The combined aqueous fractions were washed with diethyl ether (2 × 10 mL), acidified to pH 2 with aq. 1 M $KHSO_4$, and the resulting emulsion was extracted with diethyl ether (4 × 10 mL). The organic phase was washed with aq. 1 M $KHSO_4$ (2 × 10 mL), water (3 × 10 mL), brine (2 × 5 mL), dried, filtered and concentrated under reduced pressure. The residue was triturated

with cold pentane and filtered. The resulting extremely viscous oil was dried at 0.02 Torr for a prolonged time to give the target protected amino acid (2*R*,1'*R*,2'*R*)-**41c** (390 mg, 73%) as a colorless foam. R_f=0.08 (EtOAc/hexane 1:1); m. p. (softening) 60–67 °C; $[\alpha]_D^{20}$ = –57.6 (*c* = 0.33, $CHCl_3$); ^{1}H NMR (600 MHz, $CDCl_3$): 0.36–0.43 + 0.58–0.62 + 0.77–0.81 (3 × m, 1H), 0.88–0.92 + 0.97–1.09 (2 × m, 1H), 1.15–1.21 + 1.27–1.31 (2 × m, 1H), 1.33–1.55 + 1.81–1.87 (2 × m, 2 H), 3.73–3.78 + 4.50–4.61 (2 × m, 1H), 3.93–4.00 + 4.42–4.48 (2 × m, 2H), 4.16–4.23 (m, 1H), 5.55 (d, *J* = 7.7 Hz, 0.7H), 6.78 (d, *J* = 6.1 Hz, 0.3H), 7.28–7.33 (m, 2H), 7.33–7.38 (m, 2 H), 7.52 (t, J = 8.1 Hz, 0.6 H), 7.57 (t, J = 8.4 Hz, 1.4 H), 7.77 (d, *J* = 7.4 Hz, 2 H), 7.80–8.70 (bs, 1 H); ^{13}C NMR (125.7 MHz, $CDCl_3$): δ = 7.4 (+), 11.6 (–), 19.3 (+, d, *J* = 37 Hz), 34.5 (–), 47.0 (+), 53.8 (+), 67.7 (–), 101.6 (+, d, *J* = 272 Hz), 120.1 (+), 124.7 (+),127.6 (+), 128.0 (+), 143.1 (C_{quat}), 143.6 (C_{quat}), 156.5 (C_{quat}), 174.6 (C_{quat}); MS-ESI: (positive) *m/z* (%):825 (100, 2M+Na$]^+$), 424 (M+Na$]^+$), (negative) *m/z* (%): 801 (100, 2M–H$]^-$), 400 (19, M–H$]^-$), 204 (11, M–FmOH–H$]^-$), 196 (12, FmOH$]^-$).

*Fmoc-(R)mFmcpA-(*βMe)Phe-*Ile-ODCPM (***47c***)*: The dipeptide **42** (434 mg, 834 µmol) was taken up with EtOAc (20 mL) and hydrogenated over 10% Pd/C (250 mg) under ambient pressure of hydrogen for 2 h. The reaction mixture was filtered through a pad of Celite®, and concentrated under reduced pressure to give the deprotected dipeptide, which was directly used for the coupling with Fmoc-(*R*)mFmcpA-OH **41c** (360 mg, 860 µmol), using EDC (172 mg, 896 µmol), HOAt (120 mg, 883 µmol) and TMP (310 µL, 2.5 mmol) according to GP 2. The mixture was diluted with chloroform (50 mL) and subjected to the usual aqueous work-up according to GP 2 to give the pure tripeptide **47c** as a colorless solid (535 mg, 72%), R_f = 0.52; EtOAc/hexane 2:3; m. p. 158–161°C; $[\alpha]_D^{20}$ = –5.3 (*c* = 0.24, $CHCl_3$); ^{1}H NMR (500 MHz, $CDCl_3$): δ = 0.29–0.39 (m, 4H), 0.53–0.63 (m, 5H), 0.87 (d, *J* = 6.7 Hz, 3H), 0.92 (t, *J* = 7.4 Hz, 3H), 0.93–1.00 (m, 1H), 1.04–1.11 (m, 2H), 1.11–1.21 (m, 2H), 1.33–1.51 (m, 2H), 1.44 (d, *J* = 6.8 Hz, 3H), 1.59–1.68 (m, 1H), 1.78–1.87 (m, 2H), 3.20–3.29 (m, 1H), 3.68 (t, *J* = 8.4 Hz, 1H), 4.18 (t, *J* = 6.9 Hz, 1H), 4.32–4.40 (m, 3H), 4.40–4.51

(m, 1H), 4.68 (t, *J* = 8.0 Hz, 1H), 5.43 (d, *J* = 57.1 Hz, 2H); 5.67 (d, *J* = 7.7 Hz, 1H), 6.54 (d, *J* = 7.5 Hz, 1H), 7.12 (d, *J* = 8.1 Hz, 1H), 7.21–7.33 (m, 5H), 7.37 (t, *J* = 7.4 Hz, 2H), 7.43 (t, *J* = 7.4 Hz, 2H), 7.59 (t, *J* = 8.0 Hz, 2H), 7.80 (d, *J* = 7.5 Hz, 2H); ^{13}C NMR (125.7 MHz, $CDCl_3$) δ = 2.5, 2.9, 8.1, 11.2,14.3, 14.6, 15.0, 15.2, 25.4, 35.8, 38.3, 42.5, 47.7, 54.9, 56.6, 58.9, 67.8, 83.4, 99.9 (d, *J* = 256 Hz), 119.4, 124.9, 127.6, 127.5, 127.1, 128.4, 129.0, 141.8, 143.7, 143.8, 155.5, 169.4, 170.4, 170.6.

*Fmoc-(*βMe)Phe*-(R)mFmcpA-(*βMe)Phe*-Ile-ODCPM (***49c***)*: The tripeptide **47c** (394 mg, 500 µmol) was deprotected according to GP 1, and the resulting *C*-protected tripeptide was then directly coupled with Fmoc-*(*βMe)Phe-OH **46** (211 mg, 525 µmol) according to GP 2 using EDC (99 mg, 518 µmol), HOAt (70 mg, 512 µmol) and TMP (175 mg, 1440 µmol) in CH_2Cl_2 (3 mL). After 16 h, the reaction mixture was diluted with chloroform (50 mL) and subjected to the usual aqueous work-up according to GP 2 to give the crude tetrapeptide, which was twice recrystallized from THF/hexane to give the pure target tetrapeptide **49c** as an off-white solid (440 mg, 88%). R_f = 0.29 ($CHCl_3$/MeOH 70:1); m. p. 207–211 °C (decomp.); $[\alpha]_D^{20}$ = –28.3 (*c* = 0.23, THF); ^{1}H NMR (250 MHz, $CDCl_3$): δ = 0.22–0.36 (m, 4 H), 0.41 (t, *J* = 8.9 Hz, 3 H), 0.48–0.61 (m, 2 H), 0.62–0.71 (m, 1 H), 0.76 (d, *J* = 6.9 Hz, 3 H), 0.82 (t, *J* = 7.3 Hz, 3 H), 0.92–1.09 (m, 4 H), 1.09–1.18 (m, 1 H), 1.22–1.46 (m, 2 H) 1.26 (d, *J* = 6.9 Hz, 3 H), 1.39 (d, *J* = 6.6 Hz, 3 H), 1.71–1.89 (m, 1 H), 3.09–3.36 (m, 3 H), 3.81 (t, *J* = 8.4 Hz, 1 H), 4.01–4.12 (m, 2H), 4.15–4.33 (m, 3 H), 4.35–4.64 (m, 3 H), 4.62 (t, *J* = 8.3 Hz, 1 H), 5.95 (d, *J* = 7.1 Hz, 1 H), 6.40 (d, *J* = 7.4 Hz, 1 H), 7.08–7.45 (m, 16 H), 7.57 (t, *J* = 8.9 Hz, 2 H), 7.76 (d, *J* = 7.3 Hz, 2 H); ^{13}C NMR (62.9 MHz, $CDCl_3$): δ = 3.1 (+), 3.4 (–), 3.5 (–), 6.7 (–), 12.3 (+), 15.5 (+), 15.7 (+), 16.3 (+), 17.5 (+), 19.1 (+), 19.3 (–),35.4 (–), 40.0 (+), 42.3 (+), 44.4 (+), 48.5 (+), 52.7 (+), 57.1 (+), 59.4 (+), 60.0 (+), 67.7 (–), 83.3 (+), 101,1 (–, d, *J* = 261 Hz), 126.6 (+), 127.3 (+), 127.8 (+), 128.3 (+), 128.7 (+), 129.0 (+), 129.1 (+), 129.2 (+), 129.5 (+), 142.2 (C_{quat}), 144.2 (C_{quat}), 144.5 (C_{quat}), 145.8 (C_{quat}), 157.7 (C_{quat}), 171.5 (C_{quat}), 171.7 (C_{quat}), 171.9 (C_{quat}), 172.2 (C_{quat}).

*Boc-(4-Pe)Pro-[MeZ-a-Thr]-(*βMe)Phe*-(S)mFmcpA-(*βMe)Phe-*Ile-ODCPM* (**51c**): The tetrapeptide **49c** (332 mg, 350 µmol) was *N*-deprotected according to GP 1 and taken up with anhydrous CH_2Cl_2 (5 mL). A solution of the ester acid **53** (194 mg, 385 µmol), HATU (160 mg, 420 µmol) and HOAt (53 mg, 385µmol) in CH_2Cl_2 (3 mL) were added, and the reaction mixture was cooled to 4 °C. After this, a solution of DIEA (65 µL, 48 mg, 368 µmol) and TMP (140 µL, 127 mg, 1050 µmol) in CH_2Cl_2 (2 mL) was added at the same temperature within 5 min. The temperature was allowed to reach 20 °C, and stirring was continued for an additional 15 h. After aqueous work-up according to GP 2 and two recrystallizations from EtOAc/hexane (1:2), the target hexadepsipeptide **51c** (390 mg, 86%) was obtained as a colorless solid. R_f=0.45 (EtOAc/hexane 1:1); m. p. 133–137 °C; $[\alpha]_D^{20}$ = –21.0 (*c* = 0.22, THF); ^{1}H NMR (250 MHz, $CDCl_3$): δ = 0.22–0.70 (m, 12H), 0.77 (d, *J* = 6.9 Hz, 3H), 0.88 (t, *J* = 7.1 Hz, 3H), 0.99–1.18 (m, 5H), 1.18–1.44 (m, 1H), 1.22 (d, *J* = 6.6 Hz, 3H), 1.28 (d, *J* = 6.7 Hz, 3H), 1.33 (s, 9H), 1.41 (d, *J* = 6.7 Hz, 3H), 1.66 (d, *J* = 7.0 Hz, 3H), 1.77–1.97 (m, 2H), 2.31–2.44 (m, 1H), 2.38 (s, 3H), 3.10–3.43 (m, 4H), 3.66 (t, *J* = 8.6 Hz, 1H), 3.94 (t, *J* = 8.3 Hz, 1H), 4.02–4.31 (m, 3H), 4.31–4.54 (m, 4H), 4.65 (t, *J* = 9.6 Hz, 1H), 5.05–5.20 (m, 2H), 5.21–5.33 (m, 1H), 5.45–5.65 (m, 2H), 6.56 (d, *J* = 7.7 Hz, 1H), 6.68 (d, *J* = 8.8 Hz, 1H), 6.86 (d, *J* = 8.4 Hz, 1H), 7.15–7.40 (m, 14H), 7.47 (d, *J* = 10.1 Hz, 1H), 7.77 (d, *J* = 8.1 Hz, 1H); ^{13}C NMR (62.9 MHz, $CDCl_3$): δ = 2.2 (–), 2.4 (–), 2.8 (–), 5.1 (+), 11.5 (+), 13.0 (+), 14.1 (+), 14.4 (+), 15.7 (+), 17.5 (+),18.6 (+), 19.3 (+), 21.0 (+),25.3 (–), 28.3 (+), 36.0 (–), 36.4 (+), 37.0 (+), 40.5 (+), 42.4 (+), 50.2 (+), 52.8 (+), 56.3 (–), 59.0 (–), 59.9 (+), 61.1 (+), 61.4 (+), 62.0 (+), 66.9 (–), 70.3 (+), 80.2 (C_{quat}), 83.4 (+), 116.2 (+, d, *J* = 283 Hz), 126.8 (+), 127.0 (+), 127.1 (+), 127.5 (+), 127.9 (+), 128.3 (+), 128.6 (+), 128.7 (+), 128.8 (+), 128.9 (+), 133.1 (C_{quat}), 137.3 (C_{quat}), 141.4 (C_{quat}), 141.7 (C_{quat}), 154.6 (C_{quat}), 155.5 (C_{quat}), 170.2 (C_{quat}), 170.3 (C_{quat}), 170.9 (C_{quat}), 171.7 (C_{quat}), 173.1 (C_{quat}), 174.6 (C_{quat}).

N-MeZ-protected cyclohexadepsipeptide **52c** *(Cyclo-$F_1$6-MeZ)*: The hexadepsipeptide **51c** (300 mg, 247 µmol) was deprotected at the ends by treatment with 2 M HCl solution in ethyl

acetate (5 mL). The reaction mixture was stirred for 20 min in a dark place (aluminum foil jacket) at ambient temperature, and all volatiles were removed *in vacuo* without any heating. The residue was triturated with anhydrous diethyl ether (10 mL) to give the hydrochloride of the deprotected material as a colorless solid (232 mg, 220 µmol, 89%). The ends-deprotected hexadepsipeptide, HATU (110 mg, 288 µmol) and HOAt (33 mg, 244 µmol) were dissolved in cold (4 °C, internal temperature) anhydrous CH_2Cl_2 (2.5 L), and a solution of DIEA (120 µL, 93 mg, 720 µmol) in CH_2Cl_2 (100 mL) was added dropwise within 1 h, the cooling bath was removed and the mixture was stirred for 2 h at ambient temperature. The mixture was cooled again to 4 °C (internal temperature), then second portions each of HATU (110 mg, 288 µmol) and HOAt (33 mg, 244 µmol) were added, followed by dropwise addition of a solution of DIEA (120 µL, 93 mg, 720 µmol) in CH_2Cl_2 (100 mL) within 1 h. The cooling bath was removed, and the mixture was stirred at ambient temperature for 18 h. The mixture was concentrated under reduced pressure, subjected to aqueous work-up according to GP 2 to give the crude protected cyclohexadepsipeptide (180 mg, 73%) which was finally purified by HPLC to give pure product **52c** (132 mg, 49%).

N-Teoc-(2S,1'S,2'R)-3-(2'-Fluoromethylcyclopropyl)alanine [Teoc-(S)mFmcA-OH, **53c***]*: A solution of TeocOSu (43 mg, 164 µmol) in acetone (1 mL) was added to a vigorously stirred solution of (2*S*,1'*S*,2'*R*)-3-(2'-fluoromethylcyclopropyl)alanine [(2*S*,1'*S*,2'*R*)-**9c**] (27 mg, 137 µmol) and $NaHCO_3$ (24 mg, 286 µmol) in water (1 mL) (if an emulsion formed, acetone and/or water was added to obtain a homogeneous solution), and stirring was continued for another 2 h. *N*,*N*-Dimethylaminopropylamine (8 µL, 6.4 mg, 52 µmol) was then added. After an additional 10 min, acetone was removed under reduced pressure, and the pH of the residual water solution was adjusted to 2–3 with aq. 1 M $KHSO_4$ solution. The resulting emulsion was extracted with diethyl ether (50 mL), and the ethereal layer was washed with aq. 1 M $KHSO_4$ (2 × 10 mL), water (3 × 10 mL), brine (2 × 5 mL), dried over $MgSO_4$, filtered and concentrated under reduced pressure. The residual oil was dried overnight *in vacuo* to give the glass-like product **53c** (38 mg, 71%). R_f=0.24 [EtOAc/hexane 1:3 (2% AcOH)];

$[\alpha]_D^{20}$ = +28.2 (c = 0.34, $CHCl_3$); ^{1}H NMR (250 MHz, $CDCl_3$): δ = 0.03 (s, 9 H), 0.99 (dd, J = 9.5 Hz, 7.3 Hz, 2 H), 1.05–1.13 (m, 1 H), 1.56–1.89 (m, 2 H), 2.01–2.22 (m, 2 H), 4.16–4.24 (m, 3 H), 4.32–4.54 (m, 1 H), 5.35–5.43 (m, 1 H), 7.00–7.30 (bs, 1 H); ^{13}C NMR (62.9 MHz, $CDCl_3$): δ = –1.9 (+), 3.3 (+), 10.1 (–), 12.0 (+), 17.7 (–), 34.3 (–), 52.3 (+), 64.6 (–), 105.1 (d, J = 275 Hz), 154.7 (C_{quat}), 175.2 (C_{quat}).

*Teoc-(S)mFmcpA-Cyclo-$F_1$6 (***55c***)*: The *N*-MeZ-protected cyclohexadepsipeptide **52c** (25 mg, 25 µmol) was deprotected with 10% anisole in TFA (1.1 mL) in the dark at ambient temperature for 2 h. After evaporation of TFA, the residue was treated with toluene (5 mL), concentrated under reduced pressure, and the crude product was dried *in vacuo* at ambient temperature for 2 h. A solution of Teoc-(*S*)mFmcpA-OH **53c**, HATU (29 mg, 75 µmol) and HOAt (10 mg, 75 µmol) in CH_2Cl_2 (1.5 mL) was added at 4 °C, followed by a solution of DIEA (3 5 mg, 27 µmol) and TMP (27 mg, 225 µmol) in CH_2Cl_2 (1.5 mL), and the mixture was stirred at ambient temperature for 15 h. The reaction mixture was then diluted with diethyl ether (50 mL), and the crude product obtained after the usual aqueous work-up (GP 2) was purified by crystallization from CH_2Cl_2/pentane to give Teoc-(*S*)mFmcpA-Cyclo-$F_1$6 (**55c**) (29 mg, 89%) as a colorless solid (R_f = 0.43, acetone/hexane 1:2) which was used for the next step without any characterization.

MOM-O-Protected [(monofluoromethylcyclopropyl)alanyl]hormaomycin (MOM-O-F_1Horm, **57c***)*: The Teoc group was cleaved off from compound **55c** (8.0 mg, 7.08 µmol) by stirring with TFA (0.6 mL) within 1 h. The mixture was concentrated under reduced pressure at 20 °C and then taken up with toluene (3 × 15 mL), which was distilled off to remove the last traces of TFA. The resulting deprotected depsipeptide was coupled with *O*-MOM protected acid **54** (2.9 mg, 14.10 µmol) using HATU (5.4 mg, 14.20 µmol), DIEA (0.92 mg, 7.12 µmol) and TMP (5.14 mg, 42.42 µmol) in CH_2Cl_2 (1 mL) according to GP 6 for 2.5 h. The mixture was then taken up with Et_2O (20 mL), and the crude product obtained after the usual aqueous work-up (GP 2) was crystallized from CH_2Cl_2/pentane to give *O*-MOM protected

[(monofluoromethylcyclopropyl)alanyl]hormaomycin **57c** (8.0 mg, 93%) as a colorless glass (R_f = 0.36, acetone/hexanes 1:2) and was used for the next step without any characterization.

[(Monofluoromethylcyclopropyl)alanyl]hormaomycin analogue (F_1Horm, **8c***)*: The *O*-MOM protected [(monofluoromethylcyclopropyl)alanyl]hormaomycin **57c** (8.0 mg, 6.82 µmol) was deprotected using $MgBr_2{\cdot}Et_2O$ (52 mg, 201.36 µmol) and EtSH (0.10 mL, 1.9 mmol) in CH_2Cl_2 (10 mL) according to GP 7 within 3 h. The mixture was taken up with EtOAc, and the crude product obtained after the usual aqueous work-up was crystallized from CH_2Cl_2/pentane to give **8c** (5.5 mg, 78%, 50% over 5 steps from **52c**) as a colorless solid, which was finally purified by preparative HPLC. R_f = 0.27 (acetone/hexanes 3:7); analytical HPLC: column B, isocratic, 65% MeCN in H_2O for 15 min, then gradient 65→99% MeCN in H_2O for 5 min, then isocratic, 99% MeCN, flow rate = 0.5 mL/min, t_R = 14.54 min, purity > 92%; preparative HPLC: isocratic, 62% MeCN in H_2O (+ 0.1% TFA) for 7 min, then gradient 65→99% MeCN in H_2O (+ 0.1% TFA) for 10 min, then isocratic, 99% MeCN in H_2O (+ 0.1% TFA), flow rate = 18 mL/min, t_R = 12.54 min; $[\alpha]_D^{20}$ = +20.2 (*c* = 0.1, MeOH); ^{1}H NMR (600 MHz, $CDCl_3$): δ = –0.69 to –0.60 (m, 1H), –0.22 to 0.11 (m, 1H), 0.22–0.33 (m, 1H), 0.50–0.55 (m, 1H), 0.90 (t, *J* = 7.4 Hz, 3H), 0.93–0.99 (m, 1H), 1.03 (d, *J* = 6.9 Hz, 3H), 1.02–1.15 (m, 1H), 1.18–1.33 (m, 1H), 1.33 (d, *J* = 7.0 Hz, 3H), 1.41 (d, *J* = 7.3 Hz, 3H), 1.47–1.55 (m, 1H), 1.55 (d, *J* = 6.9 Hz, 3H), 1.51–1.60 (m, 1H), 1.66 (dd, *J* = 6.9 Hz, 1.6 Hz, 3H), 1.77–1.83 (m, 2H), 1.83–1.90 (m, 3H), 2.22–2.30 (m, 1H), 2.80–2.84 (m, 1H), 2.92–3.00 (m, 1H), 3.12–3.22 (m, 2H), 3.33–3.43 (m, 1H), 3.60–3.67 (m, 1H), 3.90–4.24 (m, 7H), 4.34 (dd, *J* = 10.6 Hz, 10.6 Hz, 1H), 4.48 (dd, *J* = 9.4 Hz, 4.5 Hz, 1H), 4.60 (dd, *J* = 9.3 Hz, 2.3 Hz, 1H), 4.76 (dd, *J* = 9.0, 9.0 Hz, 1H), 5.05–5.11 (m, 1H), 5.25–5.29 (m, 1H), 5.42 (qd, *J* = 6.9 Hz, 2.4 Hz, 1H), 5.57–5.64 (m, 1H), 6.11 (d, *J* = 4.7 Hz, 1H), 6.58 (d, *J* = 6.5 Hz, 1H), 6.81 (d, *J* = 9.3 Hz, 1H), 6.84 (d, *J* = 4.7 Hz, 1H), 7.00–7.08 (m, 1H), 7.08–7.19 (m, 5H), 7.19–7.26 (m, 7H), 8.08 (d, *J* = 9.1 Hz, 1H), 9.03 (d, *J* = 9.3 Hz, 1H); ^{13}C NMR (150.8 MHz, $CDCl_3$): δ = 1.50 (+), 3.24 (+), 13.33 (–), 14.94 (–), 16.99 (+), 17.41 (+), 17.74 (+), 20.00 (+), 21.66 (+), 24.90 (–),

26.88 (+), 33.02 (+), 35.03 (–), 35.51 (–), 36.66 (–), 37.97 (+), 39.24 (+), 41.75 (+), 50.99 (+), 51.79 (+), 52.78 (–), 54.61 (+), 54.93 (+), 58.11 (+), 59.12 (+), 59.86 (+), 60.04 (+), 61.37 (+), 69.07 (+), 98.6 (+, d, *J* = 252 Hz), 103.59 (+), 101.1 (d, *J* = 257 Hz), 109.85 (+), 119.86 (C_{quat}), 121.55 (C_{quat}), 126.98 (+), 127.17 (+), 127.44 (+), 127.47 (+), 127.67 (+), 128.33 (+), 128.49 (+), 128.64 (+), 141.55 (C_{quat}), 142.11 (C_{quat}), 159.27 (C_{quat}), 168.54 (C_{quat}), 168.73 (C_{quat}), 169.75 (C_{quat}), 170.74 (C_{quat}), 171.26 (C_{quat}), 171.55 (C_{quat}), 172.86 (C_{quat}); MS-ESI: positive, *m/z* = 1125 (100, M+Na]$^+$); negative, *m/z* = 1101 (100, M–H]$^-$).

Crystal Structure Analyses (Figures 1 and 2): Crystals suitable for X-ray diffractometry of compounds were obtained by slow evaporation of their solutions in $CHCl_3$, [(*R*,*R*,*R*)-**28**] or MeOH, [(2*S*,1'*R*,2'*S*)-**26a**, (2*S*,1'*R*,2'*S*)-**26b**, (2*R*,1'*R*,2'*S*)-**26b**, and (2*S*,3*S*)-**32**]. The X-ray single crystal data have been collected on a Bruker SMART CCD 6000 (compounds **28** and **32**), a Bruker SMART 1000 (compound **26a**) and a Rigaku R-AXIS Spider IP [compounds (2*S*,1'*R*,2'*S*)-**26b** and (2*R*,1'*R*,2'*S*)-**26b**] diffractometers (graphite monochromator, $\lambda_{MoK\alpha}$, λ=0.71073Å) equipped with a Cryostream (Oxford Cryosystems) open-flow nitrogen cryostats at the temperatures 120.0(2)K or 120.0(2)K, respectively. All structures were solved by direct method and refined by full matrix least squares on F2 for all data using SHELXTL software [9]. Crystal data and parameters of refinement are summarized in Table 1.

Table 1: Crystal and data collection parameters for compounds (2*S*,1'*R*,2'*S*)-**26a**, (2*S*,1'*R*,2'*S*)-**26b**, (2*R*,1'*R*,2'*S*)-**26b**, (*R*,*R*,*R*)-**28** and (2*S*,3*S*)-**32**.

Compound	(2*S*,1'*R*,2'*S*)-**26a**	(2*S*,1'*R*,2'*S*)-**26b**	(2*R*,1'*R*,2'*S*)-**26b**	(*R*,*R*,*R*)-**28**	(2*S*,3*S*)-**32**
Formula	$C_{32}H_{30}F_3N_3NiO_3$	$C_{32}H_{31}F_2N_3NiO_3$ × CH_3OH × H_2O	$C_{32}H_{31}F_2N_3NiO_3$ × 0.5 H_2O × 1.5 CH_3OH	$C_{29}H_{29}N_3NiO_4$ × 3 $CHCl_3$	$C_{35}H_{33}N_3NiO_3$ × C H_3OH
Molecular mass	620.30	651.36	659.38	900.37	634.40
Crystal system	Tetragonal	Monoclinic	Triclinic	Orthorhombic	Orthorhombic
Space group	$P\,4_32_12$	$P\,2_1$	$P\,1$	$P\,2_12_12_1$	$P\,2_12_12_1$
Crystal size [mm]	0.52 × 0.14 × 0.10	0.33 × 0.22 × 0.08	0.30 × 0.19 × 0.07	0.46 × 0.04 × 0.02	00.44 × 0.05 × 0.03
a [Å]	9.9745(2)	9.2565(6)	11.2796(7)	10.3057(3)	8.5832(5)
b [Å]	9.9745(2)	11.8858(6)	11.5766(8)	16.3778(5)	15.6673(9)
c [Å]	57.255(2)	14.020(1)	14.0282(8)	22.3882(7)	23.0536(14)
α [°]	90.0	90.0	100.16(2)	90.0	90.0
β [°]	90.0	90.67(3)	109.83(2)	90.0	90.0
γ [°]	90.0	90.0	106.99(2)	90.0	90.0
V [$Å^3$]	5696.4(2)	1542.4(5)	1568.78(17)	3778.8(2)	3100.1(3)
Z	8	2	2	4	4
F(000)	2576	682	692	1832	1336
D [g cm^{-3}]	1.447	1.403	1.396	1.583	1.359
μ [mm^{-1}]	0.740	0.687	0.676	1.191	0.670
Θ_{max} [°]	25.49	29.50	29.50	27.50	27.50
Refl. collected	35391	16960	25002	39822	30404
Refl. independent	5151	8238	14723	8679	7118
R_{int}	0.0644	0.0657	0.0555	0.0734	0.1278
R_1 [I ≥ 2σ(I)]	0.0480	0.0487	0.0635	0.0523	0.0412
wR_2(all data)	0.0968	0.1203	0.1494	0.1319	0.0785
No. of parameters refined	379	532	822	442	530
GOOF	1.144	1.009	1.071	1.056	0.880
Absolute structure parameter	0.03(2)	0.008(11)	–0.007(12)	–0.01(2)	–0.003(13)
Largest diff. peak and hole, e·$Å^{-3}$	0.466, –0.706	0.7332, –0.498	0.5886, –0.688	0.943, –0.977	0.432, –0.449

CCDC-991863 [(2*S*,1'*R*,2'*S*)-**26a**], -991864 [(2*S*,1'*R*,2'*S*)-**26b**], -991865 [(2*R*,1'*R*,2'*S*)-**26b**], -991862 [(*R*,*R*,*R*)-**28**], -991866 [(2*S*,3*S*)-**32**], -1018305 [(2S,3R)-**32**-*m*-Cl] and -1018304 [(2S,3R)-**32**-*p*-F] contain the supplementary crystallographic data for this paper. These data can be obtained free of charge via www.ccdc.cam.ac.uk/conts/retrieving.html (or from the Cambridge Crystallographic Data Centre, 12, Union Road, Cambridge CB21EZ, UK; fax: (+44)1223-336-033; or deposit@ccdc.cam.ac.uk).

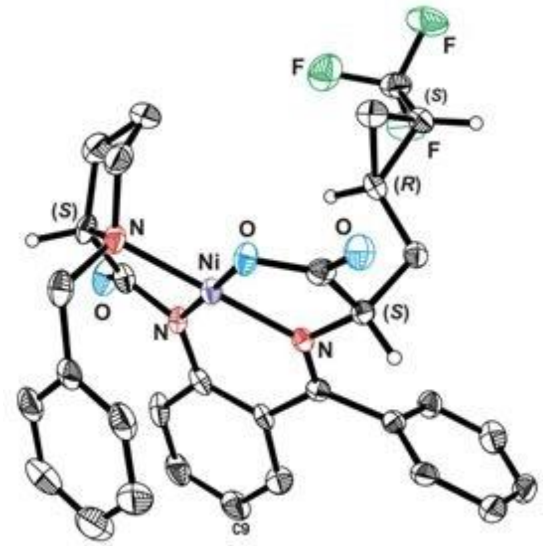

(2*S*,1'*R*,2'*S*)-**26a**

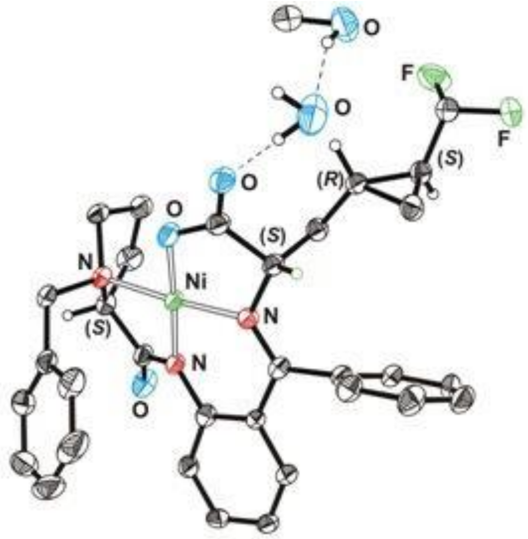

(2*S*,1'*R*,2'*S*)-**26b**

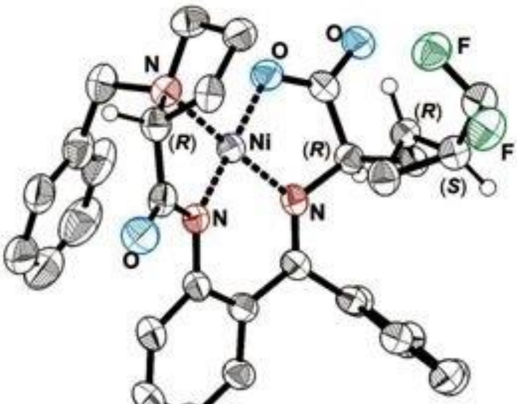

(2*R*,1'*R*,2'*S*)-**26b**

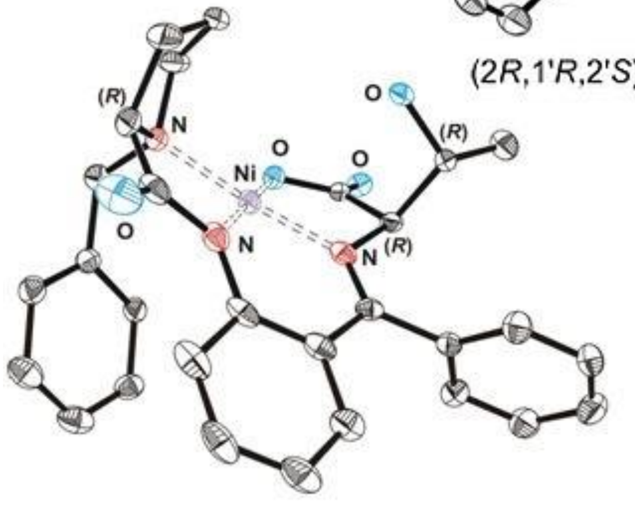

(*R*,*R*,*R*)-**28**

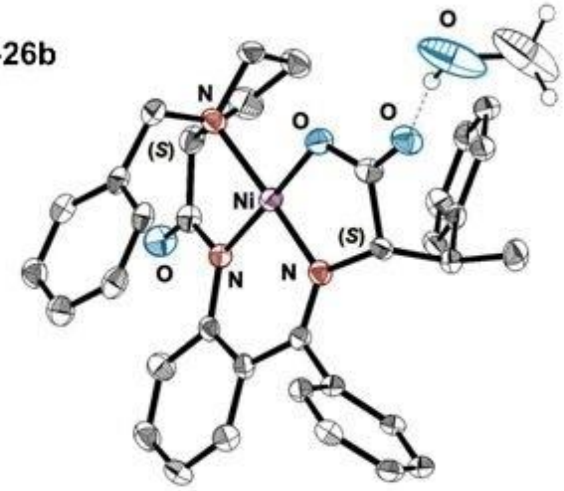

(2*S*,3*S*)-**32**

Figure 1: Structure and absolute configurations of the Belokon'-type nickel(II)-complexes (2*S*,1'*R*,2'*S*)-**26a**, (2*S*,1'*R*,2'*S*)-**26b**, (2*R*,1'*R*,2'*S*)-**26b**, (*R*,*R*,*R*)-**28** and (2*S*,3*S*)-**32** in the crystals. Hydrogen atoms are omitted for clarity.

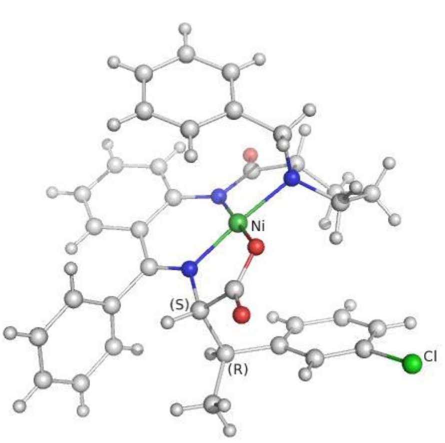

(2*S*,3*R*)-**32**-*m*-Cl

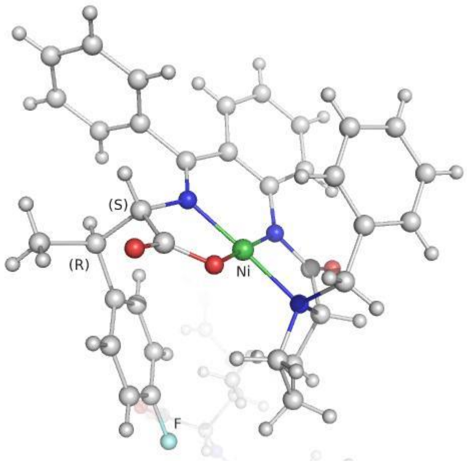

(2*S*,3*R*)-**32**-*p*-F

Figure 2: Structure and absolute configurations of the Belokon'-type nickel(II)-complexes (2*S*,3*R*)-**32**-*m*-Cl and (2*S*,3*R*)-**32**-*p*-F in the crystals.

Table 2: Crystal and data collection parameters for compounds (2*S*,3*R*)-**32**-*m*-Cl and (2*S*,3*R*)-**32**-*p*-F.

Compound	(2*S*,3*R*)-**32**-*m*-Cl	(2*S*,3*R*)-**32**-*p*-F
Formula	$C_{35}H_{32}ClN_3NiO_3$	$C_{35}H_{32}FN_3NiO_3$
Molecular mass	636.80	620.35
Crystal system	Orthorhombic	Orthorhombic
Space group	$P\ 2_1\ 2_1\ 2_1$	$P\ 2_1\ 2_1\ 2_1$
Crystal size [mm]	0.40 × 0.25 × 0.03	0.25 × 0.15 × 0.10
a [Å]	8.0379(16)	11.146(2)
b [Å]	15.338(3)	19.625(4)
c [Å]	24.070(5)	c = 26.276(5)
α [°]	90.0	90.0
β [°]	90.0	90.0
γ [°]	90.0	90.0
V [Å^3]	2967.4(10)	5748(2)
Z	4	8
F(000)	1328	2592
D [g cm^{-3}]	1.425	1.434
μ [mm^{-1}]	2.103	1.372
Θ_{max} [°]	61.04	60.02
Refl. collected	21464	40395
Refl. independent	4364	7958
R_{int}	0.0350	0.0404
R_1 [I ≥ 2σ(I)]	0.0215	0.0253
wR_2(all data)	0.0525	0.0628
No. of parameters refined	389	777
GOOF	1.040	1.024
Absolute structure parameter	–0.008(12)	–0.032(14)
Largest diff. peak and hole, e·Å^{-3}	0.135, –0.204	0.371, –0.244

^{1}H-, ^{13}C-NMR, MS- and (in part) IR-spectra as well as specific rotations of representative compounds

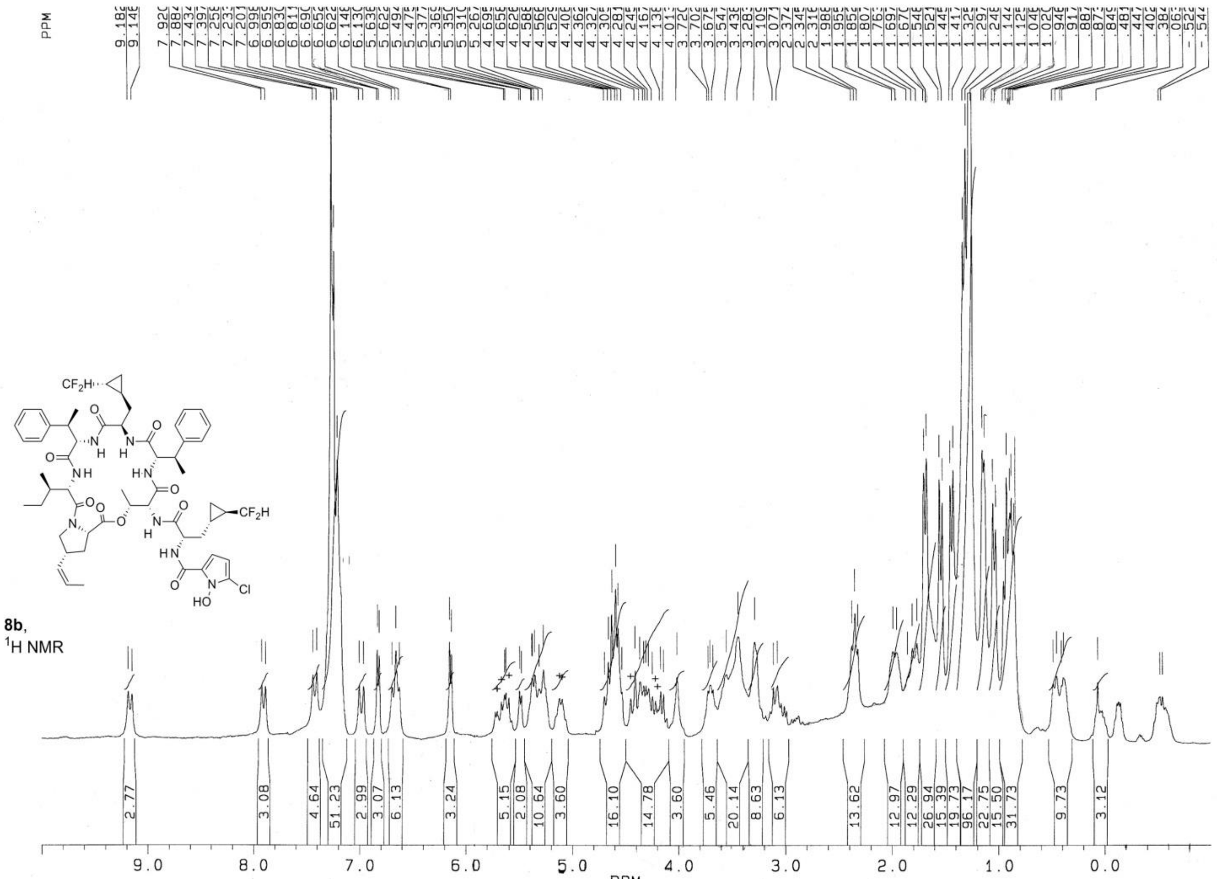

8b,
^{1}H NMR

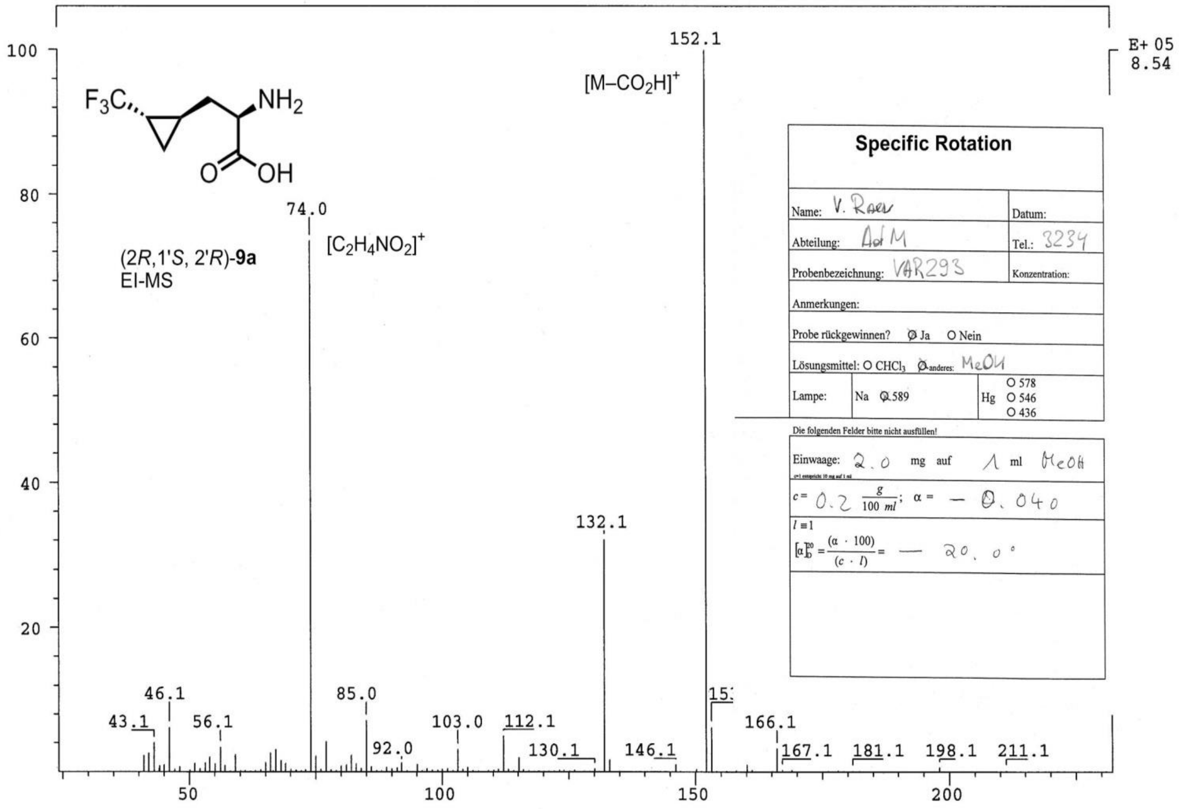
(2R,1'S, 2'R)-9a
EI-MS
152.1
[M–CO2H]+
74.0
[C2H4NO2]+
132.1
46.1
43.1
56.1
85.0
92.0
103.0
112.1
130.1
146.1
166.1
167.1
181.1
198.1
211.1
E+ 05
8.54
Specific Rotation
Name: V. Raev
Abteilung: AdM
Tel.: 3234
Probenbezeichnung: VAR293
Lösungsmittel: MeOH
Lampe: Na 589
Einwaage: 2.0 mg auf 1 ml MeOH
c = 0.2 g/100 ml; α = – 0.040
l = 1
[α]D20 = (α · 100)/(c · l) = – 20.0°

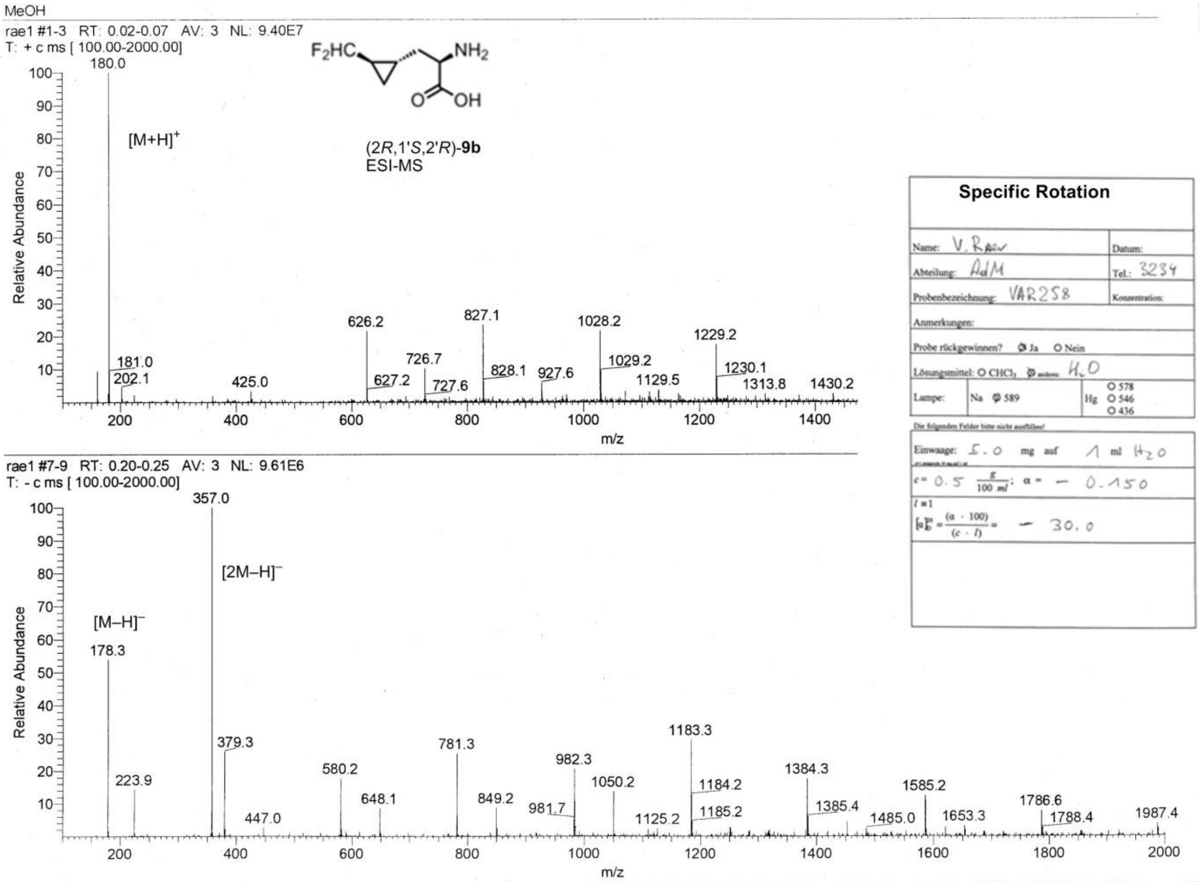
MeOH
rae1 #1-3 RT: 0.02-0.07 AV: 3 NL: 9.40E7
T: + c ms [100.00-2000.00]
(2R,1'S,2'R)-9b
ESI-MS
180.0
[M+H]+
181.0
202.1
425.0
626.2
627.2
726.7
727.6
827.1
828.1
927.6
1028.2
1029.2
1129.5
1229.2
1230.1
1313.8
1430.2
Relative Abundance
m/z
rae1 #7-9 RT: 0.20-0.25 AV: 3 NL: 9.61E6
T: - c ms [100.00-2000.00]
357.0
[2M–H]−
[M–H]−
178.3
223.9
379.3
447.0
580.2
648.1
781.3
849.2
981.7
982.3
1050.2
1125.2
1183.3
1184.2
1185.2
1384.3
1385.4
1485.0
1585.2
1653.3
1786.6
1788.4
1987.4
Specific Rotation
Name: V. Raev
Abteilung: AdM
Tel.: 3234
Probenbezeichnung: VAR258
Lösungsmittel: H2O
Lampe: Na 589
Einwaage: 5.0 mg auf 1 ml H2O
c = 0.5 g/100 ml; α = – 0.150
l = 1
[α]D20 = (α · 100)/(c · l) = – 30.0

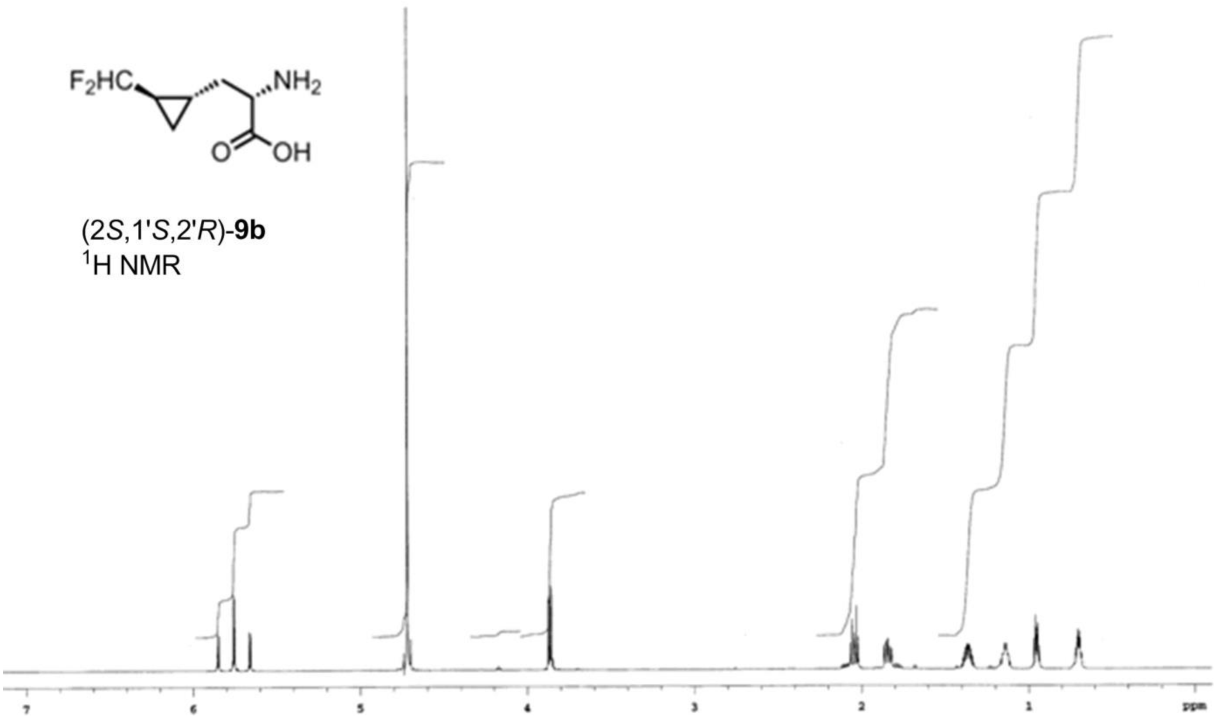
F2HC
NH2
O
OH
(2S,1'S,2'R)-9b
1H NMR
7
6
5
4
3
2
1
ppm

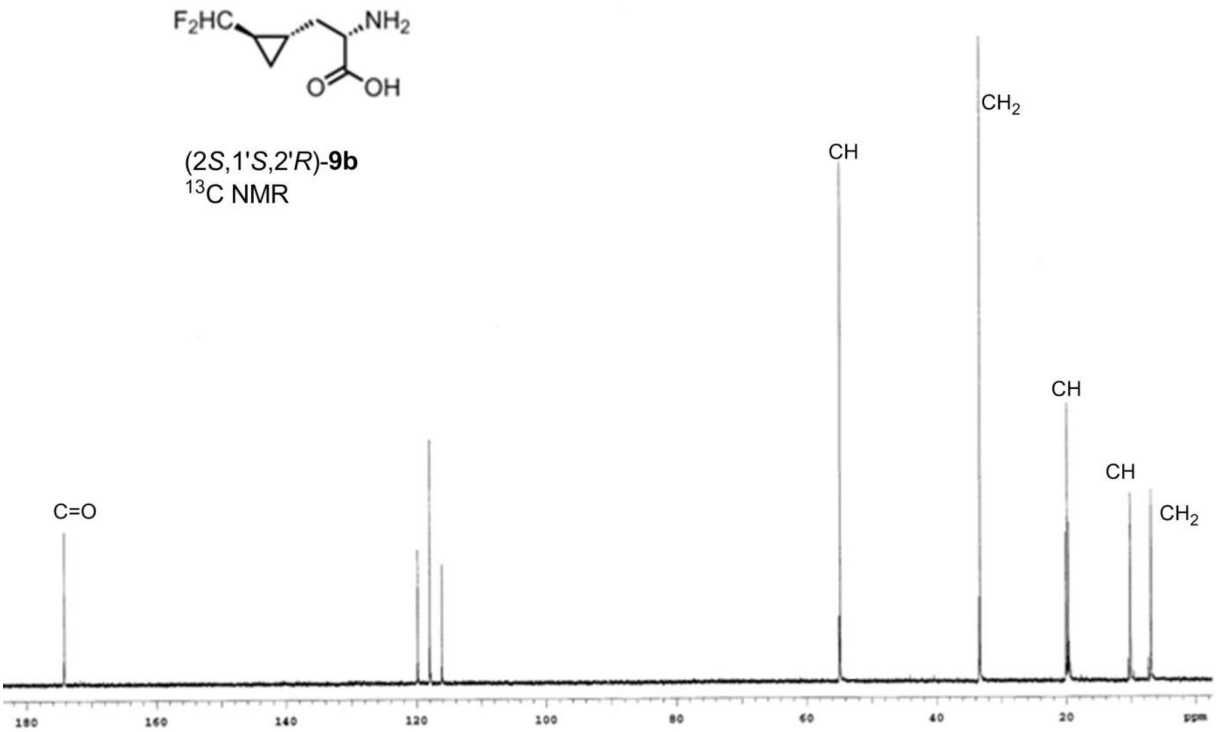
F2HC
NH2
O
OH
(2S,1'S,2'R)-9b
13C NMR
CH2
CH
CH
CH
CH2
C=O
180
160
140
120
100
80
60
40
20
ppm

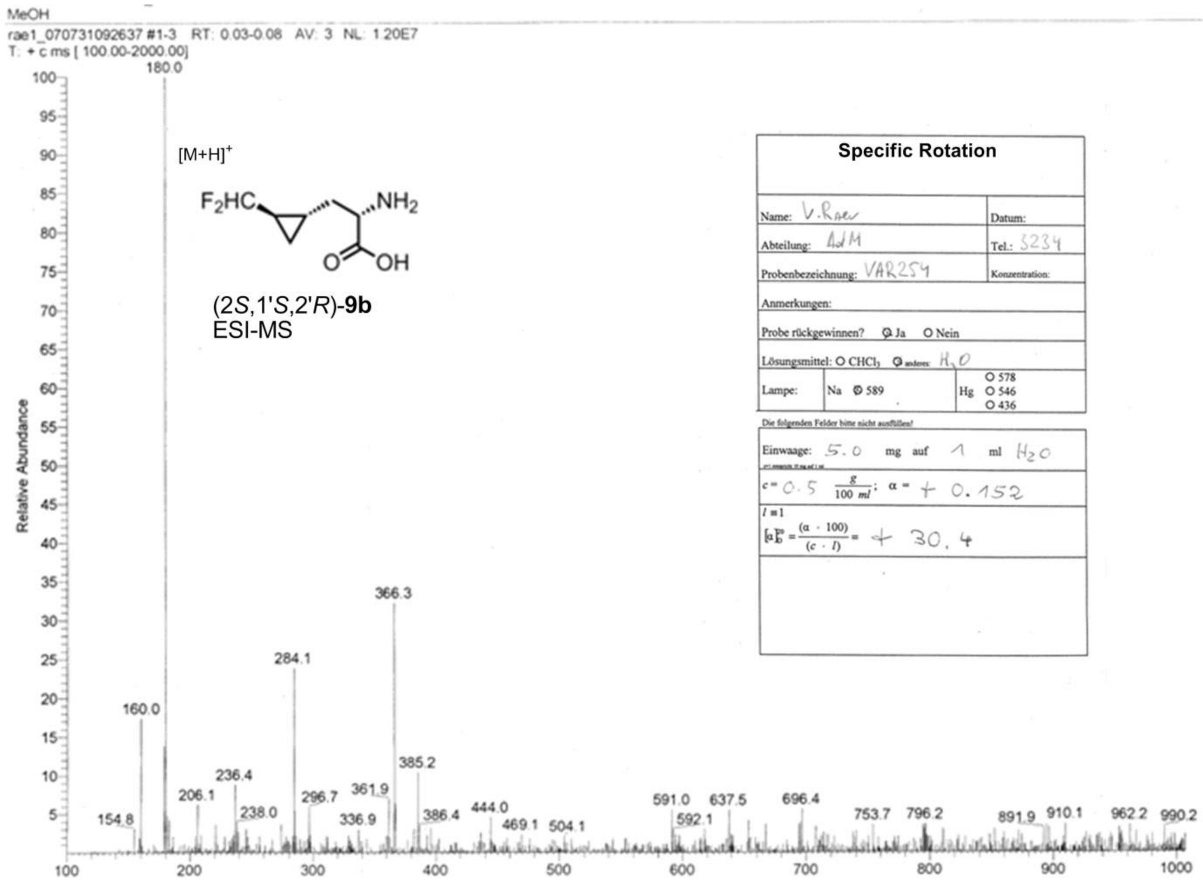
MeOH
rae1_070731092637 #1-3 RT: 0.03-0.08 AV: 3 NL: 1.20E7
T: + c ms [100.00-2000.00]
[M+H]+
(2S,1'S,2'R)-9b
ESI-MS
Relative Abundance
Specific Rotation
Name: V. Raev
Datum:
Abteilung: AdM
Tel.: 5234
Probenbezeichnung: VAR254
Konzentration:
Anmerkungen:
Probe rückgewinnen? Ja Nein
Lösungsmittel: CHCl3 anderes: H2O
Lampe: Na 589 Hg 578 546 436
Die folgenden Felder bitte nicht ausfüllen!
Einwaage: 5.0 mg auf 1 ml H2O
c = 0.5 g/100 ml; α = + 0.152
l = 1
[α]D = (α · 100)/(c · l) = + 30.4
180.0
366.3
284.1
160.0
385.2
236.4
206.1
296.7
361.9
154.8
238.0
336.9
386.4
444.0
469.1
504.1
591.0
592.1
637.5
696.4
753.7
796.2
891.9
910.1
962.2
990.2

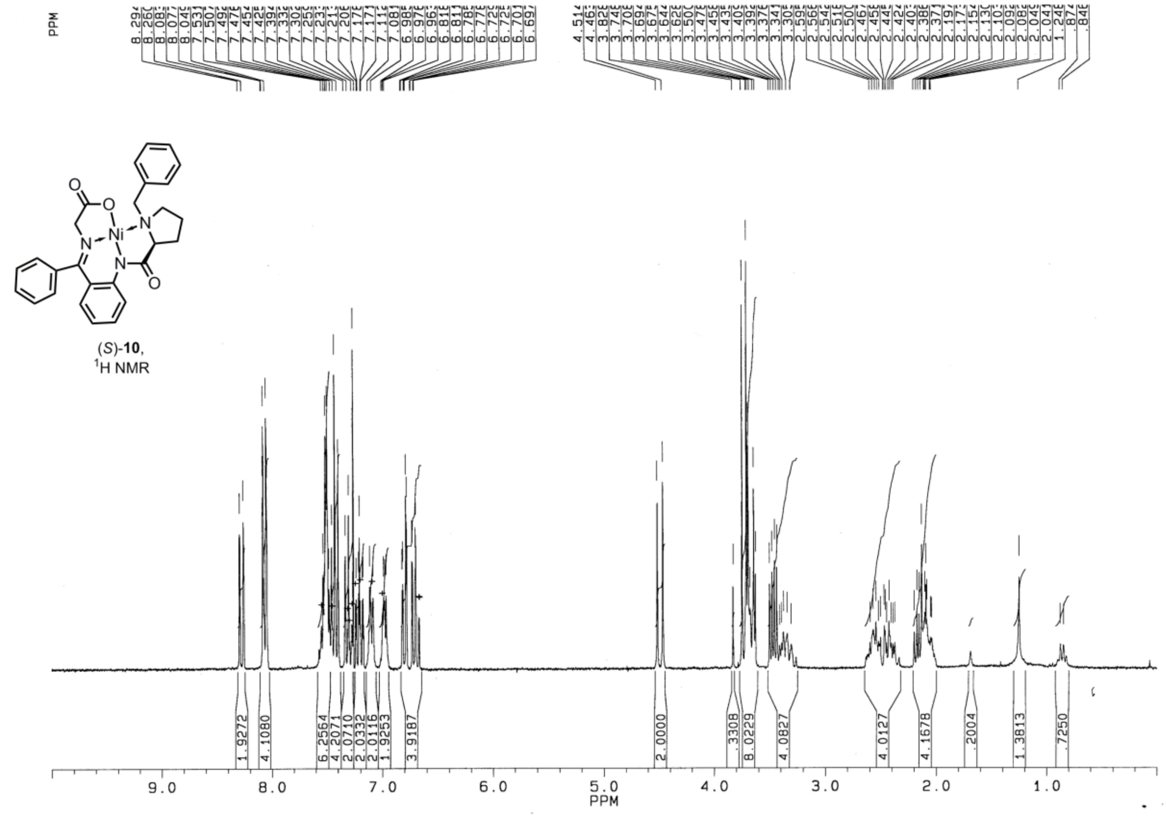
PPM
(S)-10,
1H NMR
1.9272
4.1080
6.2564
4.2071
2.0710
2.0332
2.0116
1.9253
3.9187
2.0000
.3308
8.0229
4.0827
4.0127
4.1678
.2004
1.3813
.7250
PPM

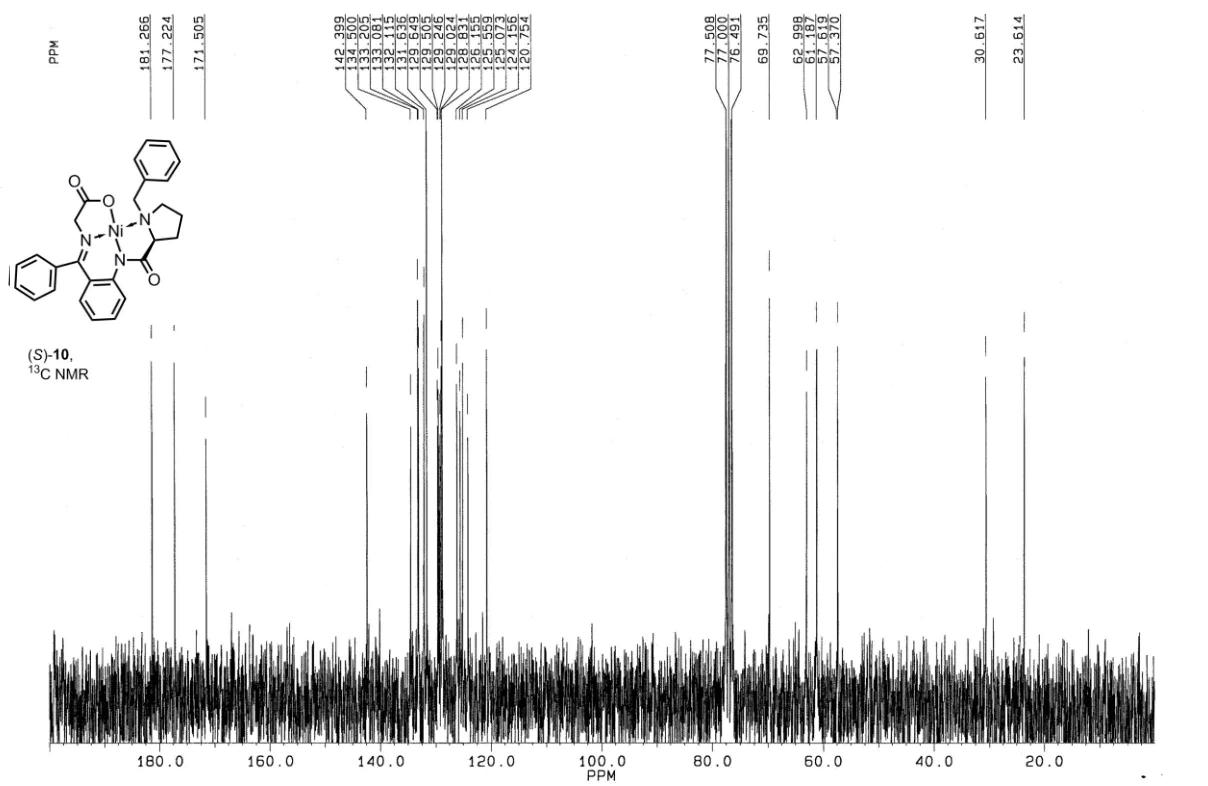

(*S*)-**10**,
^{13}C NMR

rac-**11a**
^{1}H NMR

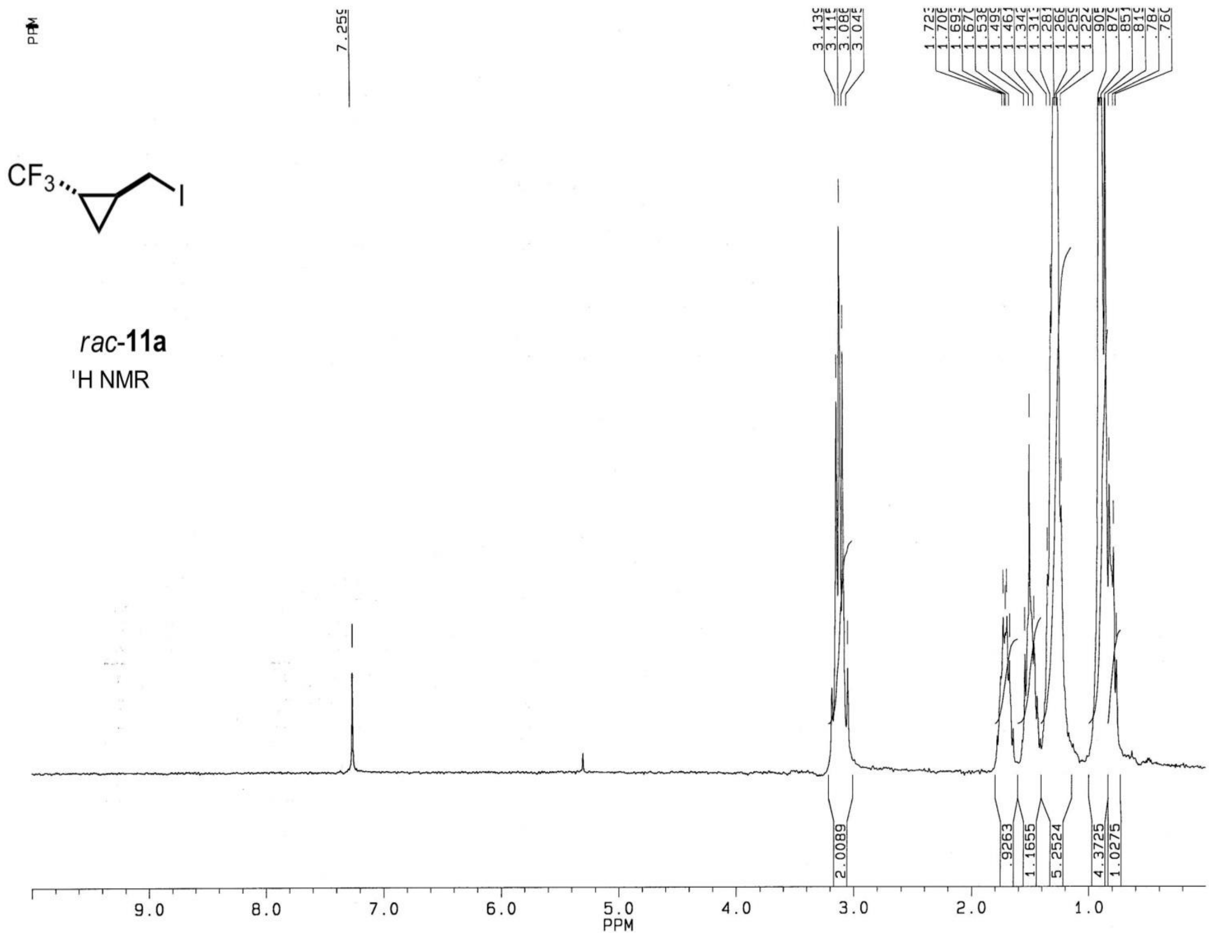

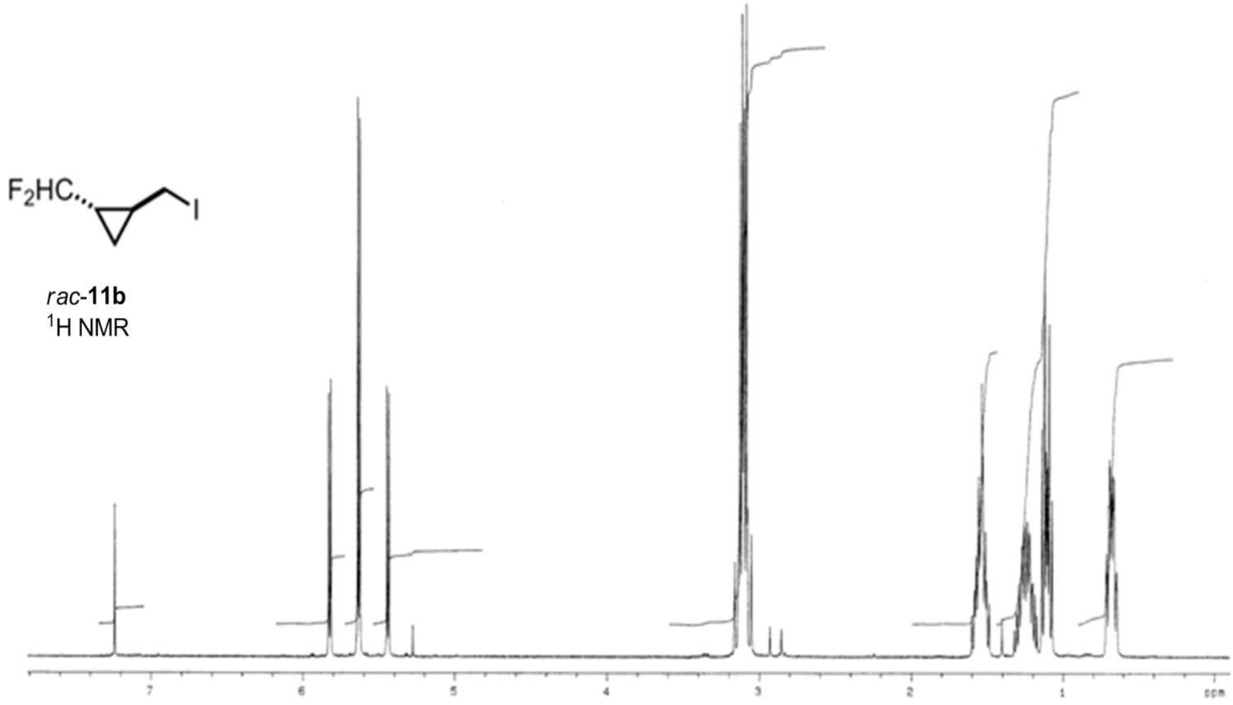

F2HC
I
rac-11b
1H NMR
7
6
5
4
3
2
1
ppm

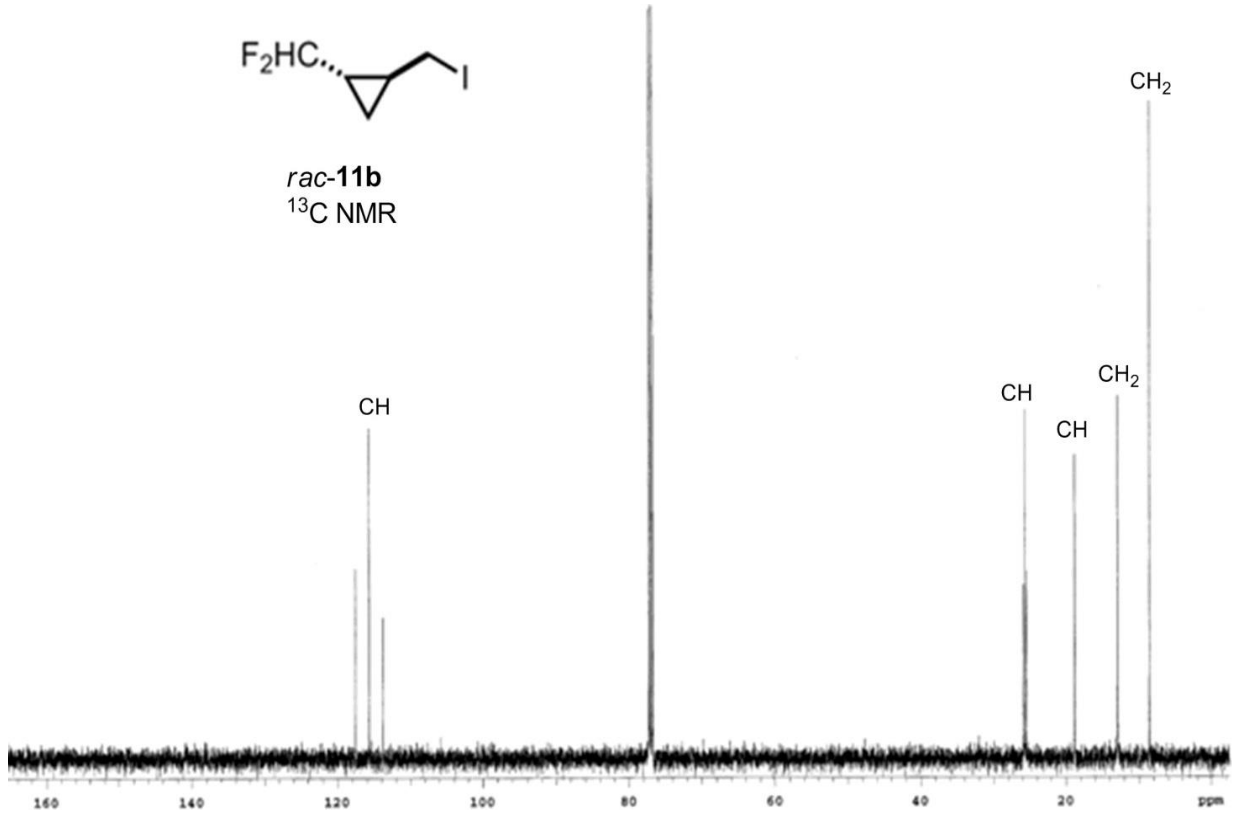

F2HC
I
rac-11b
13C NMR
CH2
CH
CH
CH2
CH
160
140
120
100
80
60
40
20
ppm

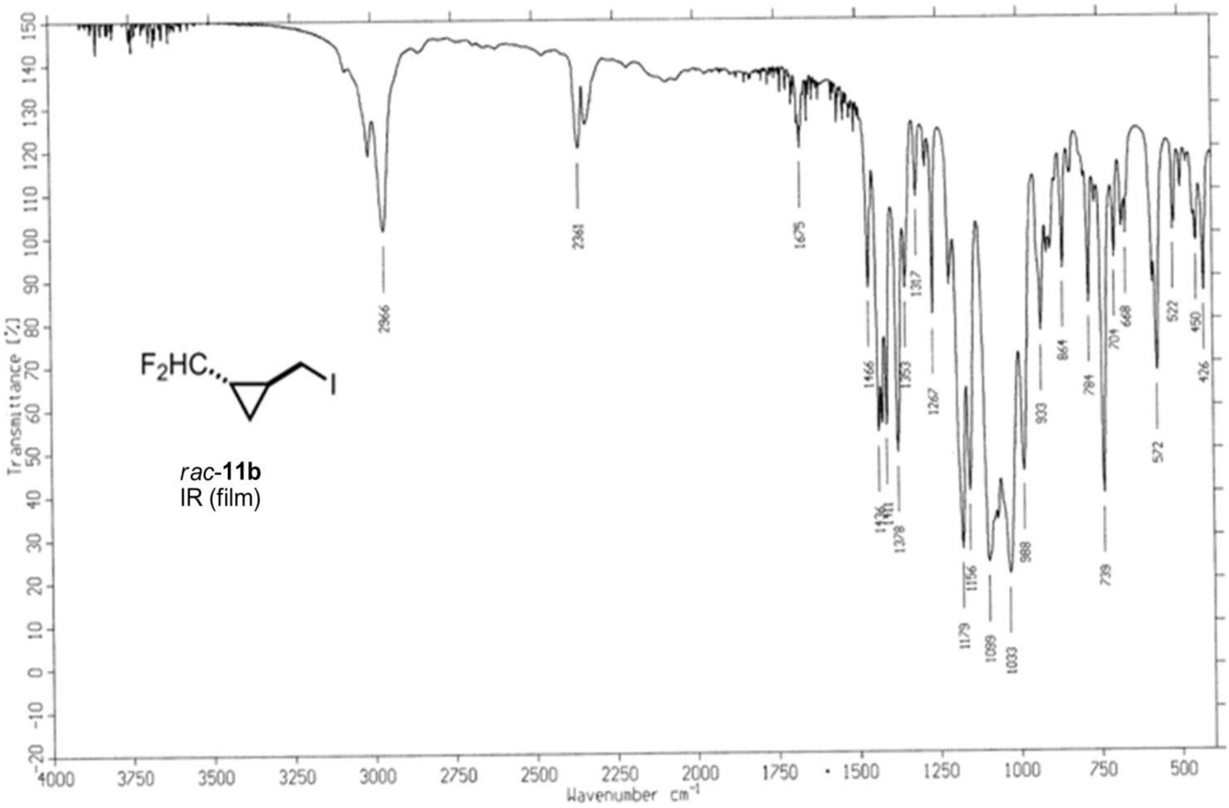
F2HC
I
rac-11b
IR (film)
Transmittance [%]
Wavenumber cm-1
2966
2361
1675
1466
1436
1411
1378
1353
1317
1267
1179
1156
1099
1033
988
933
864
784
739
704
668
572
522
450
426

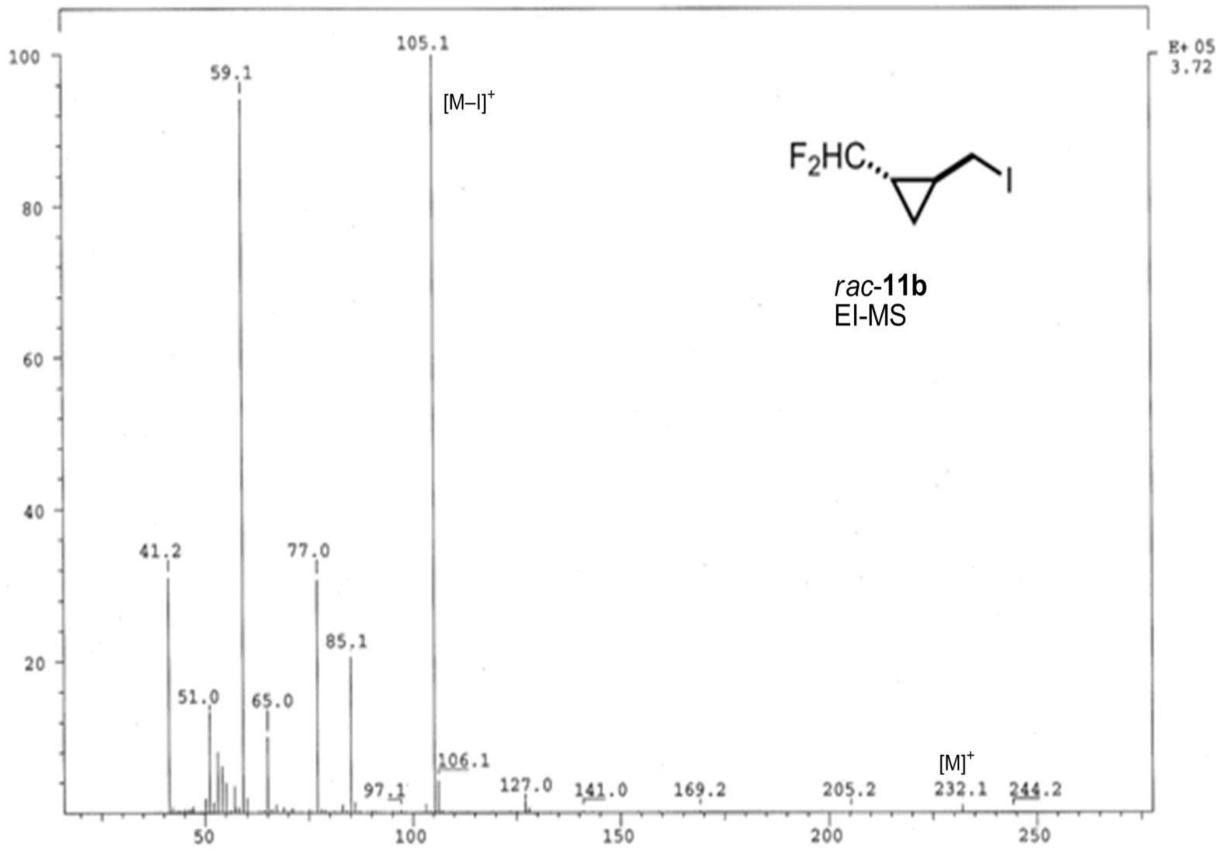
105.1
[M–I]+
59.1
E+ 05
3.72
F2HC
I
rac-11b
EI-MS
41.2
77.0
85.1
51.0
65.0
106.1
97.1
127.0
141.0
169.2
205.2
[M]+
232.1
244.2

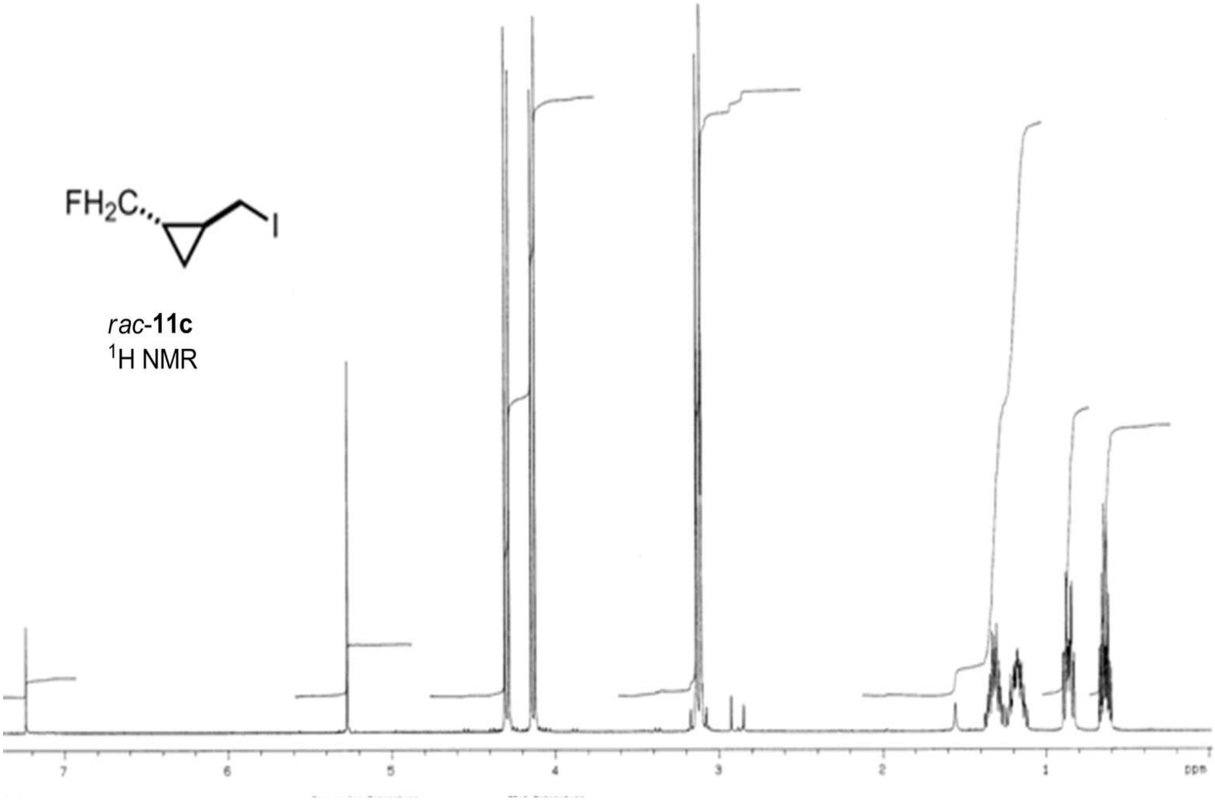

rac-**11c**
^{1}H NMR

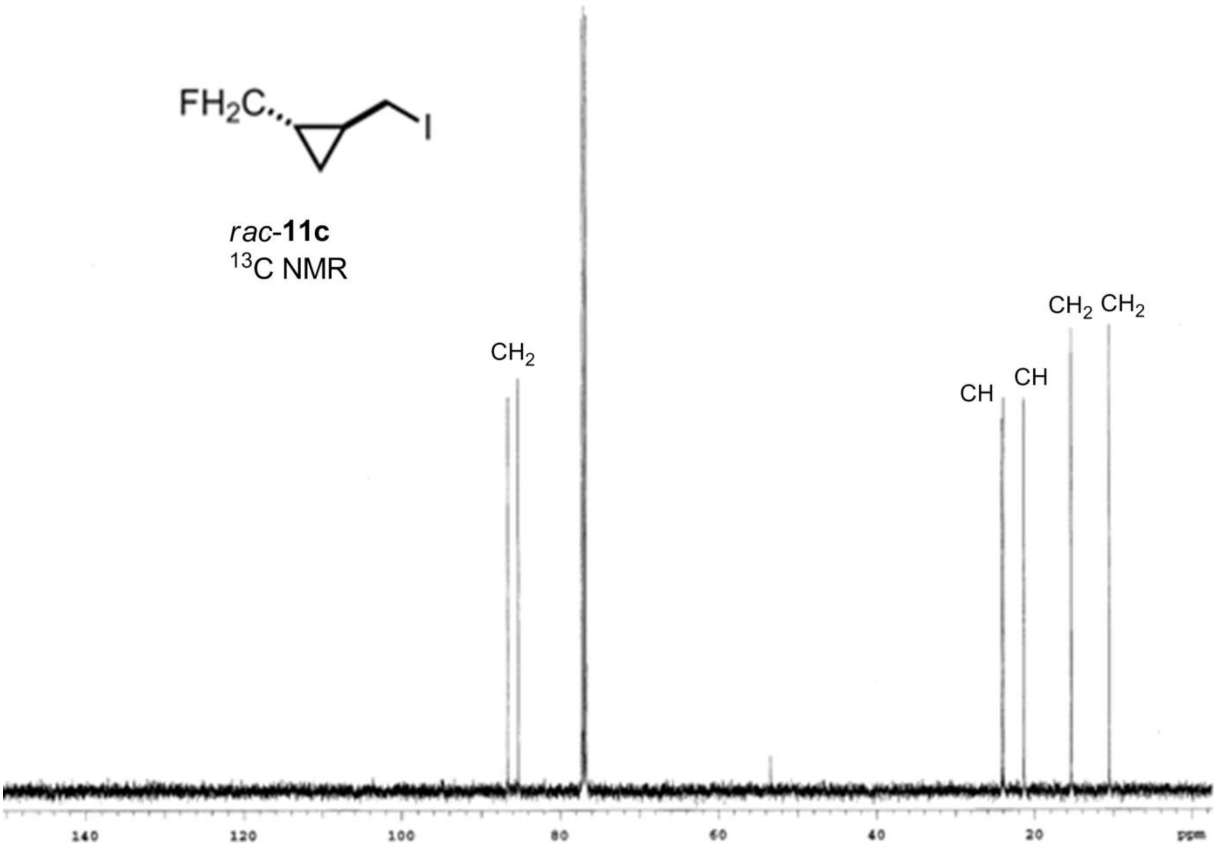

rac-**11c**
^{13}C NMR

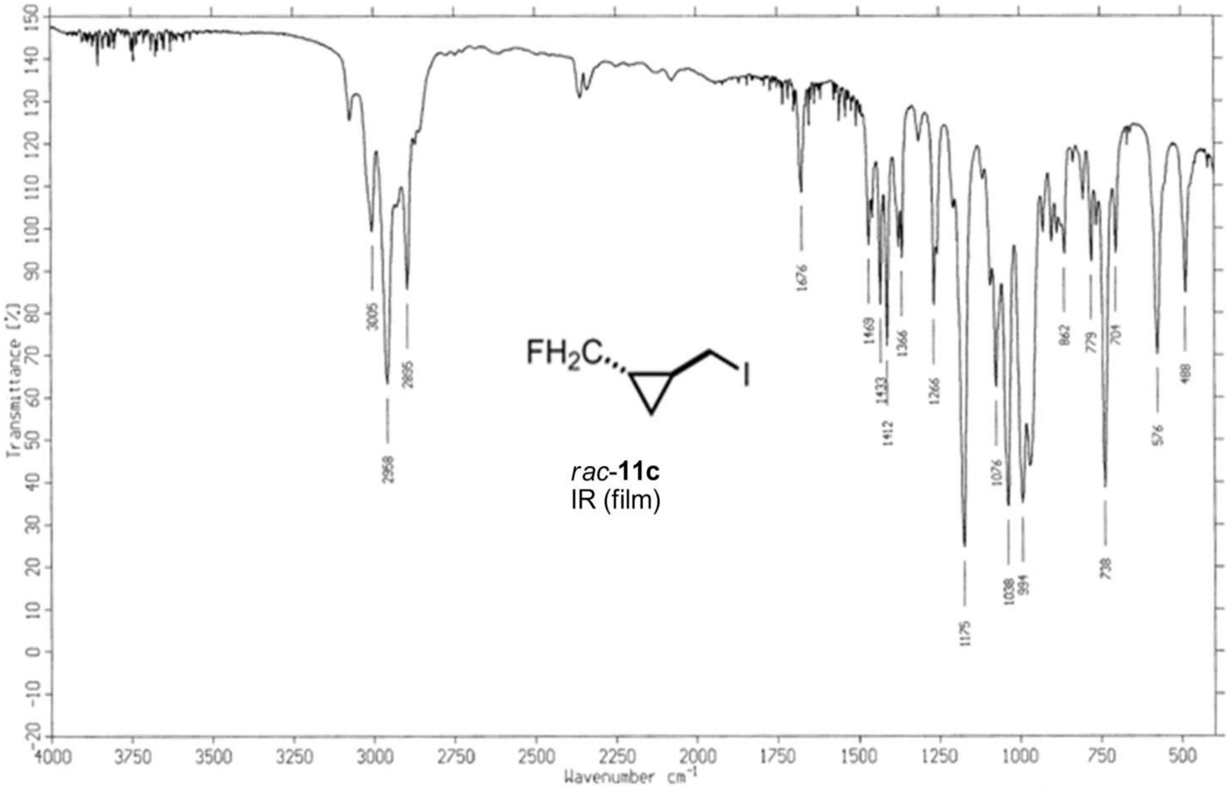

rac-**11c**
IR (film)

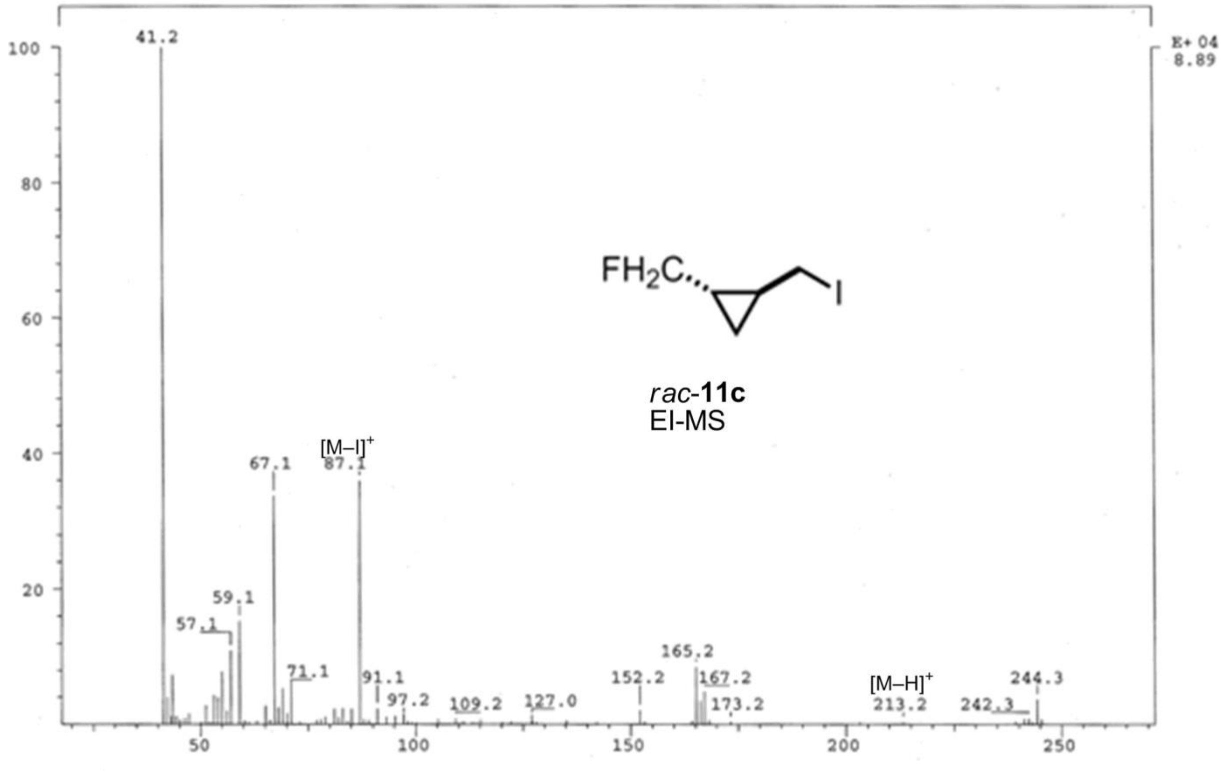

rac-**11c**
EI-MS

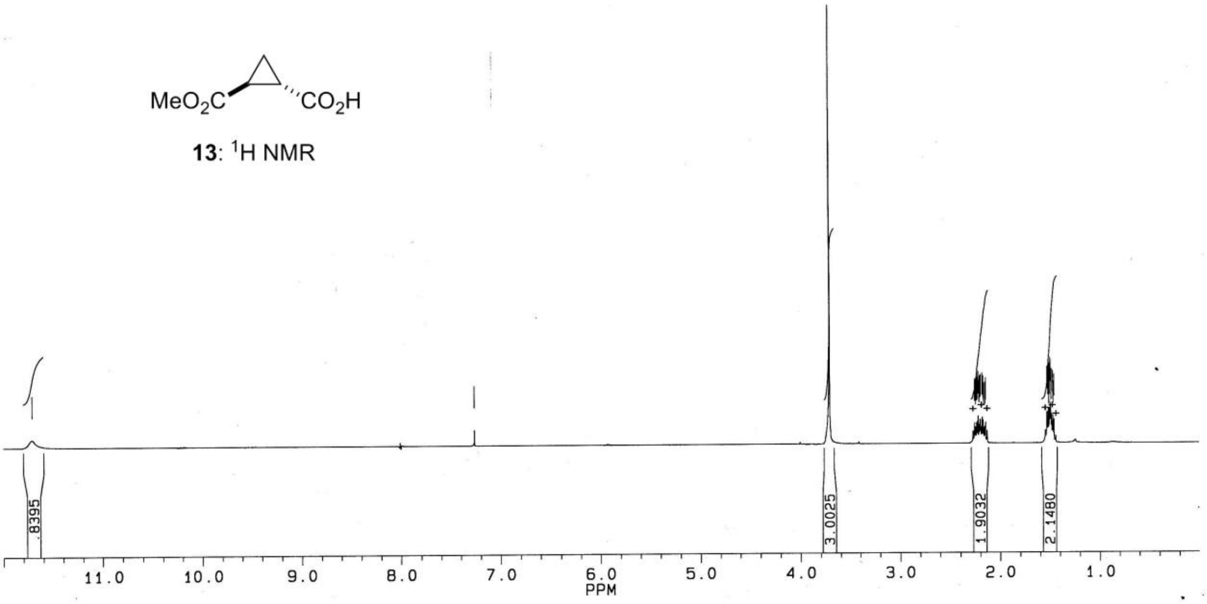
MeO2C CO2H
13: 1H NMR
.8395
3.0025
1.9032
2.1480
11.0 10.0 9.0 8.0 7.0 6.0 5.0 4.0 3.0 2.0 1.0
PPM

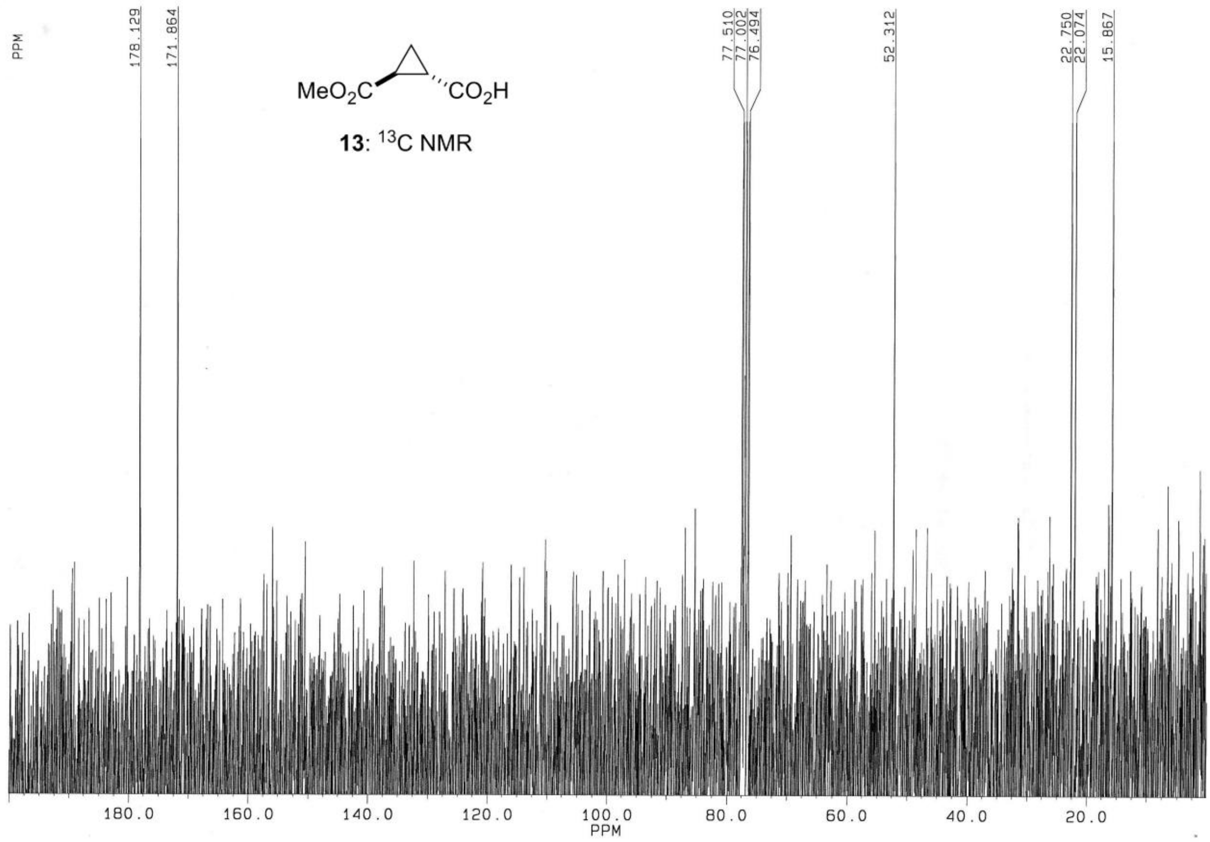
PPM
178.129
171.864
77.510
77.002
76.494
52.312
22.750
22.074
15.867
MeO2C CO2H
13: 13C NMR
180.0 160.0 140.0 120.0 100.0 80.0 60.0 40.0 20.0
PPM

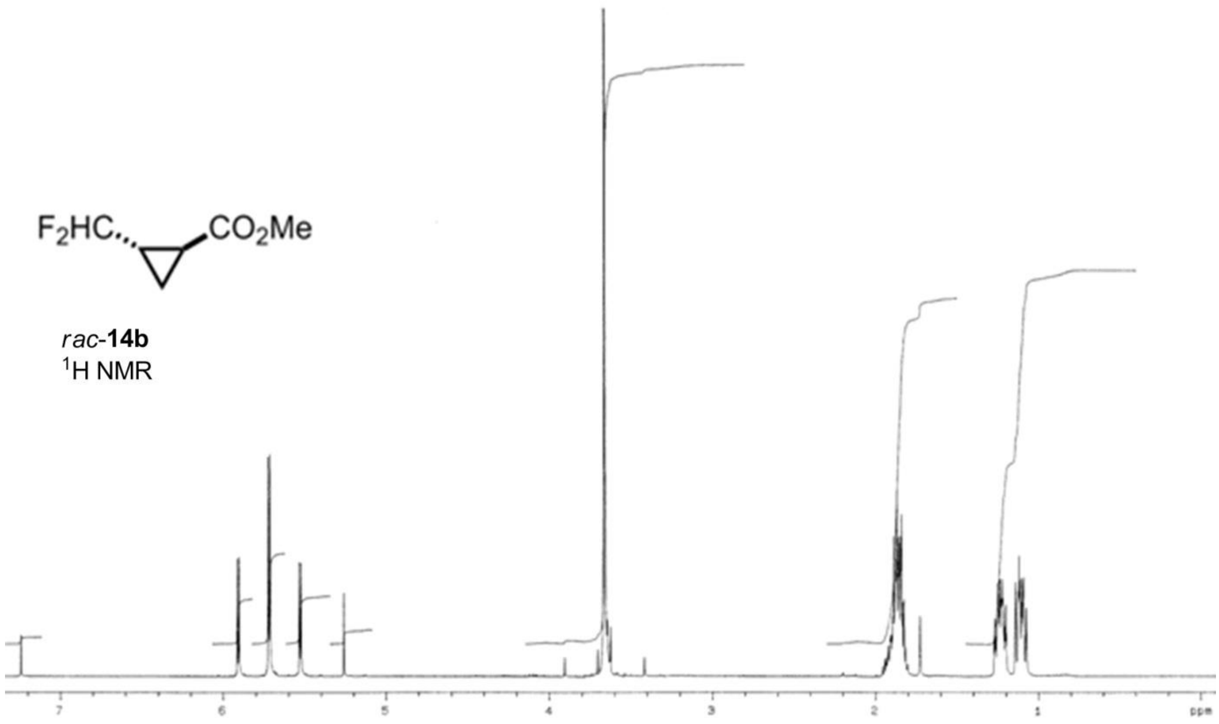
F2HC CO2Me
rac-14b
1H NMR
7
6
5
4
3
2
1
ppm

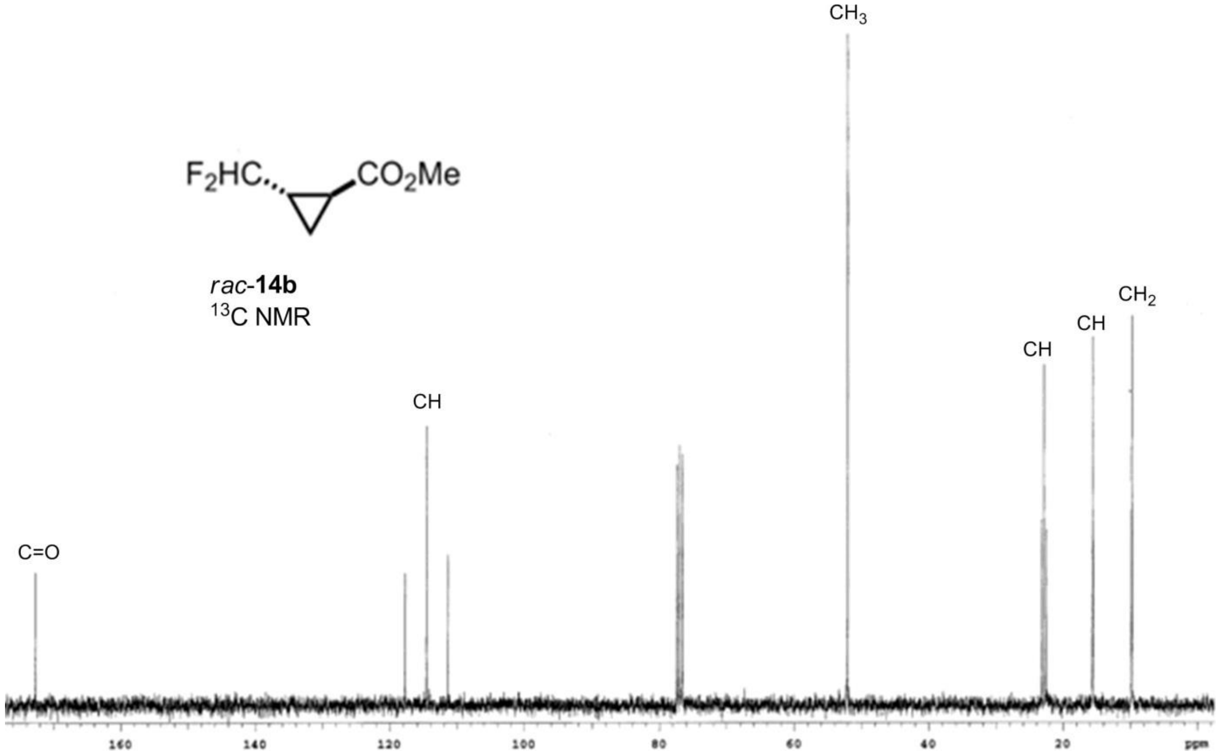
CH3
F2HC CO2Me
rac-14b
13C NMR
CH2
CH
CH
CH
C=O
160
140
120
100
80
60
40
20
ppm

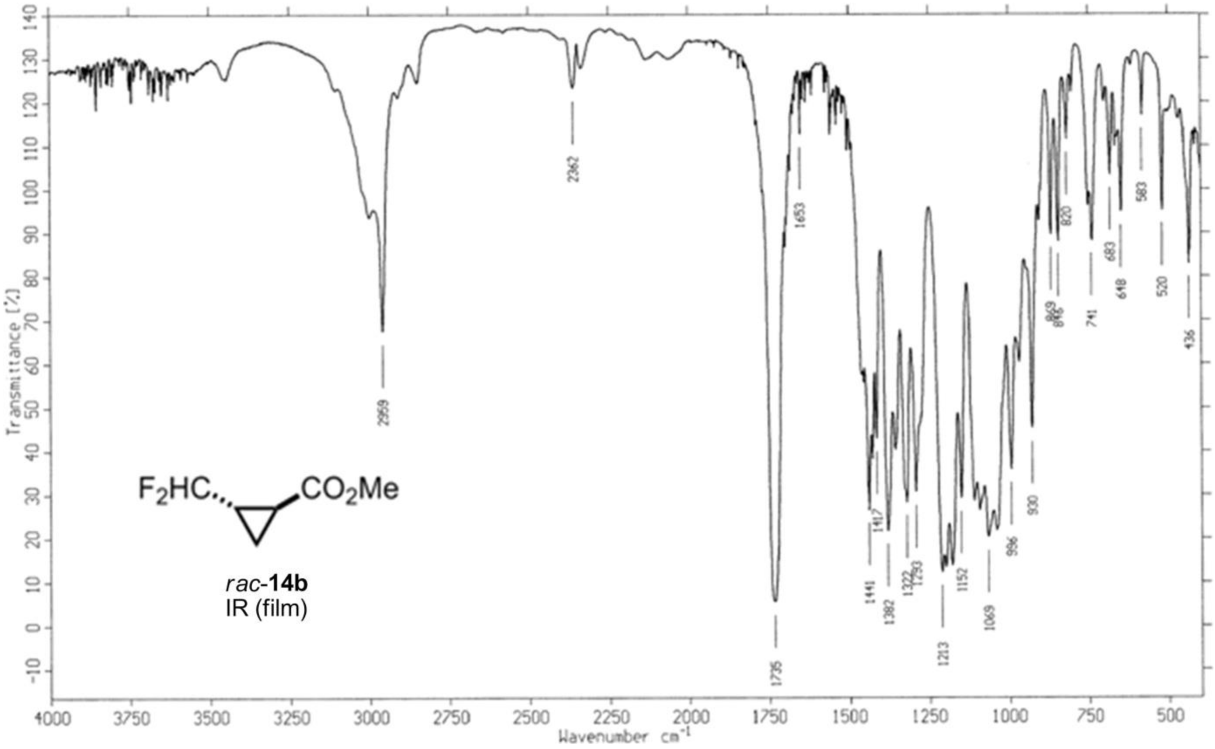
Transmittance [%]
Wavenumber cm-1
2959
2362
1735
1653
1441
1417
1382
1322
1293
1213
1152
1069
996
930
869
846
820
741
683
648
583
520
436
F2HC
CO2Me
rac-14b
IR (film)

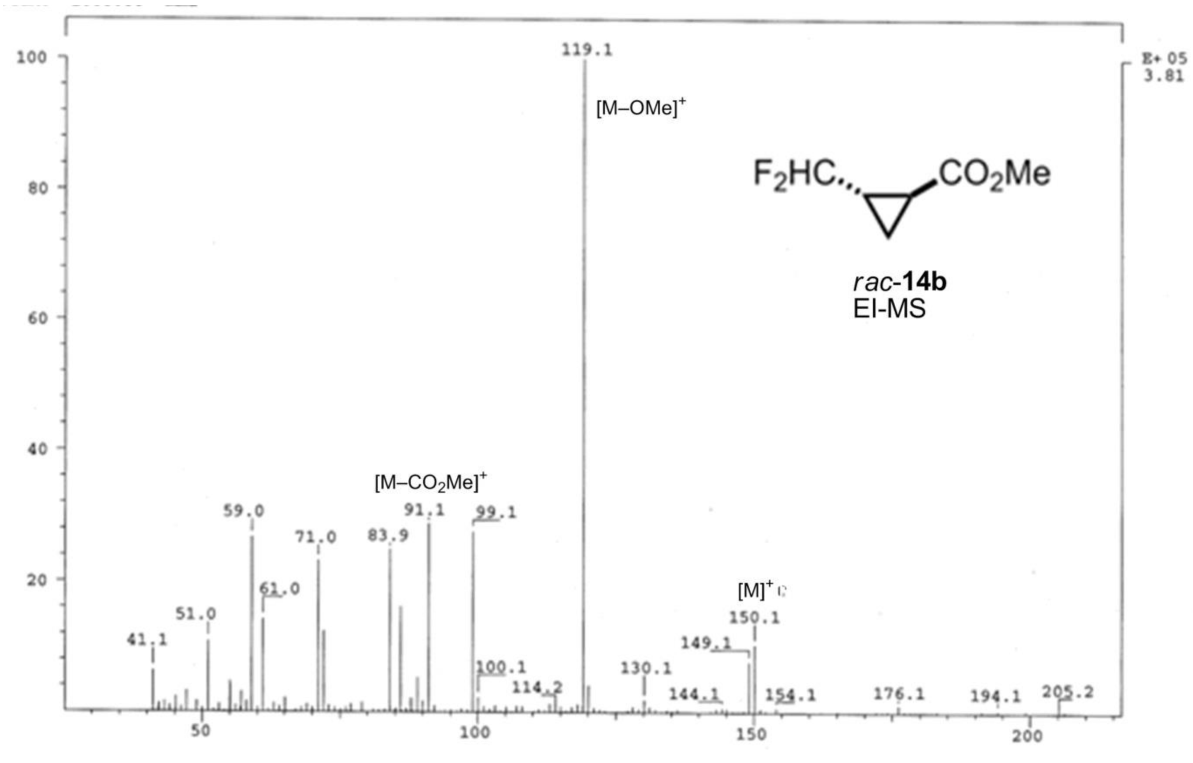
119.1
[M–OMe]+
E+ 05
3.81
F2HC
CO2Me
rac-14b
EI-MS
[M–CO2Me]+
59.0
91.1
99.1
71.0
83.9
61.0
51.0
41.1
[M]+
150.1
149.1
100.1
114.2
130.1
144.1
154.1
176.1
194.1
205.2

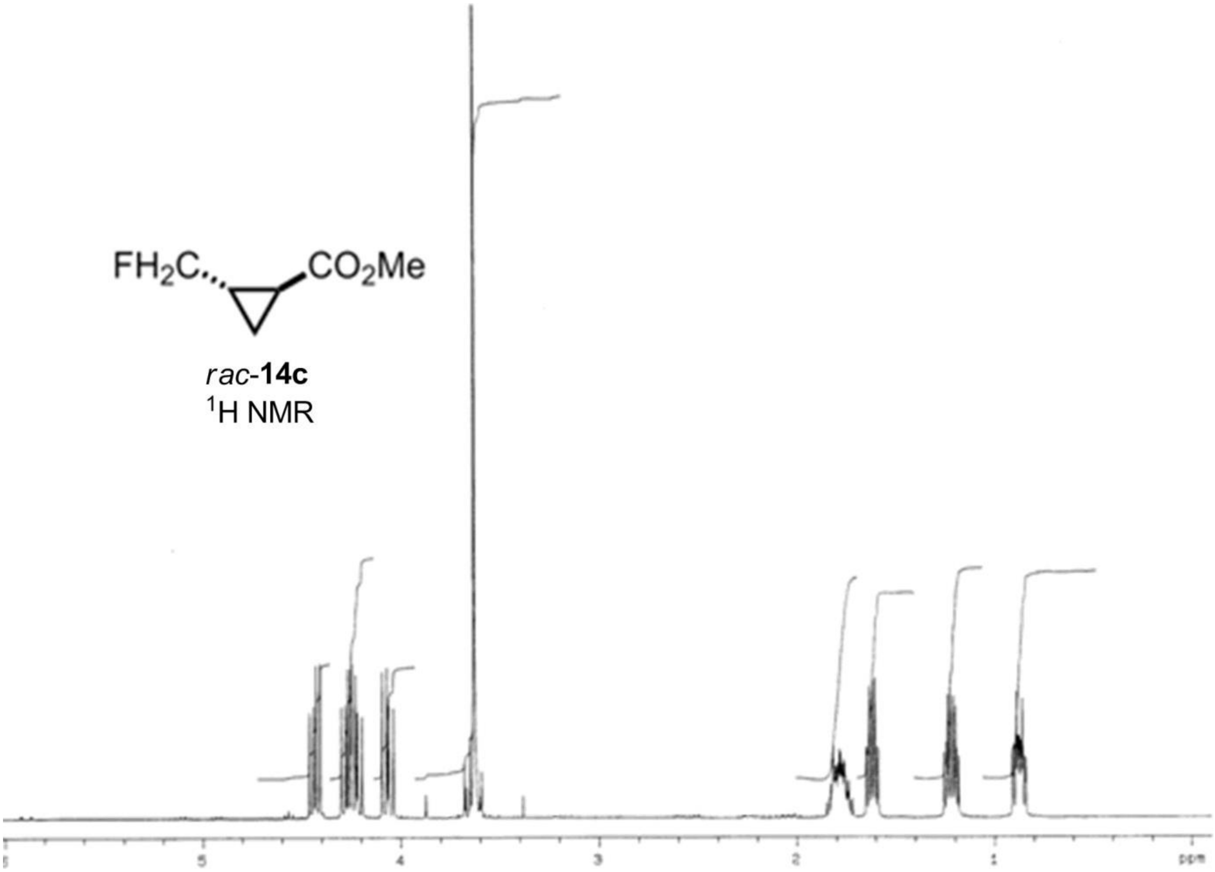

FH2C CO2Me
rac-14c
1H NMR
5
4
3
2
1
ppm

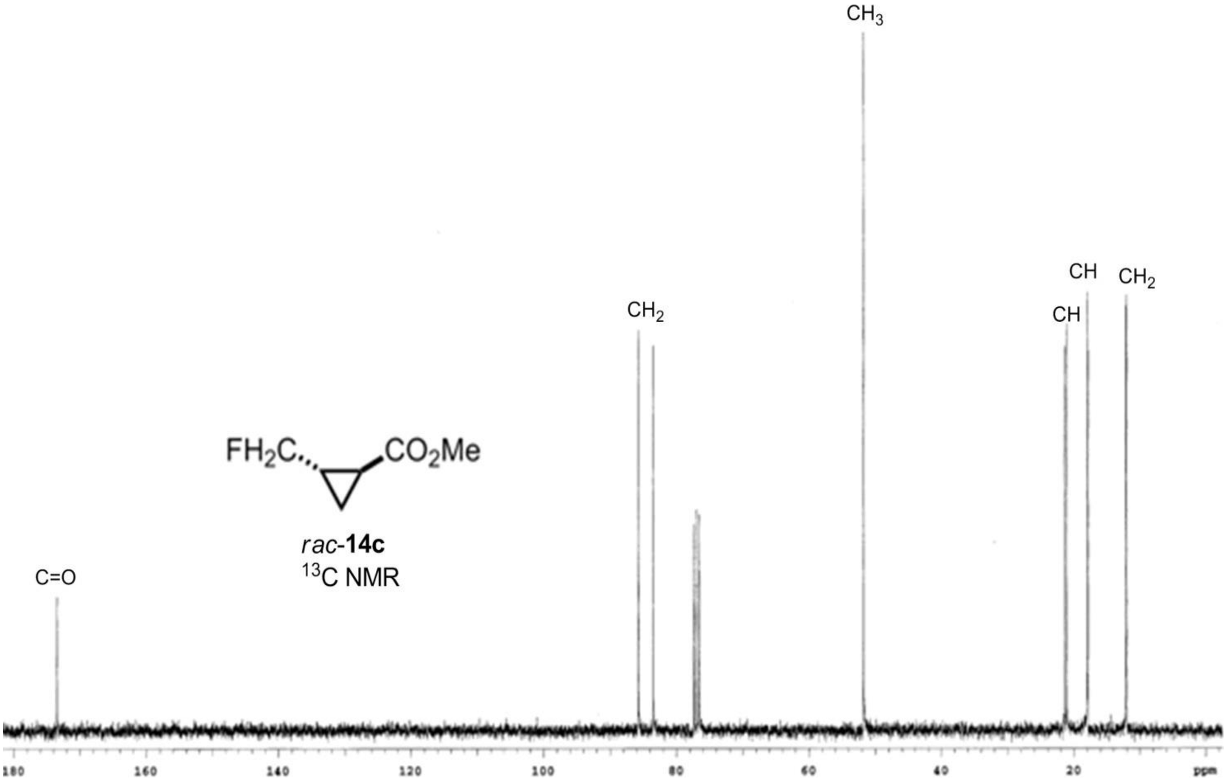

CH3
CH
CH2
CH2
CH
FH2C CO2Me
rac-14c
13C NMR
C=O
180
160
140
120
100
80
60
40
20
ppm

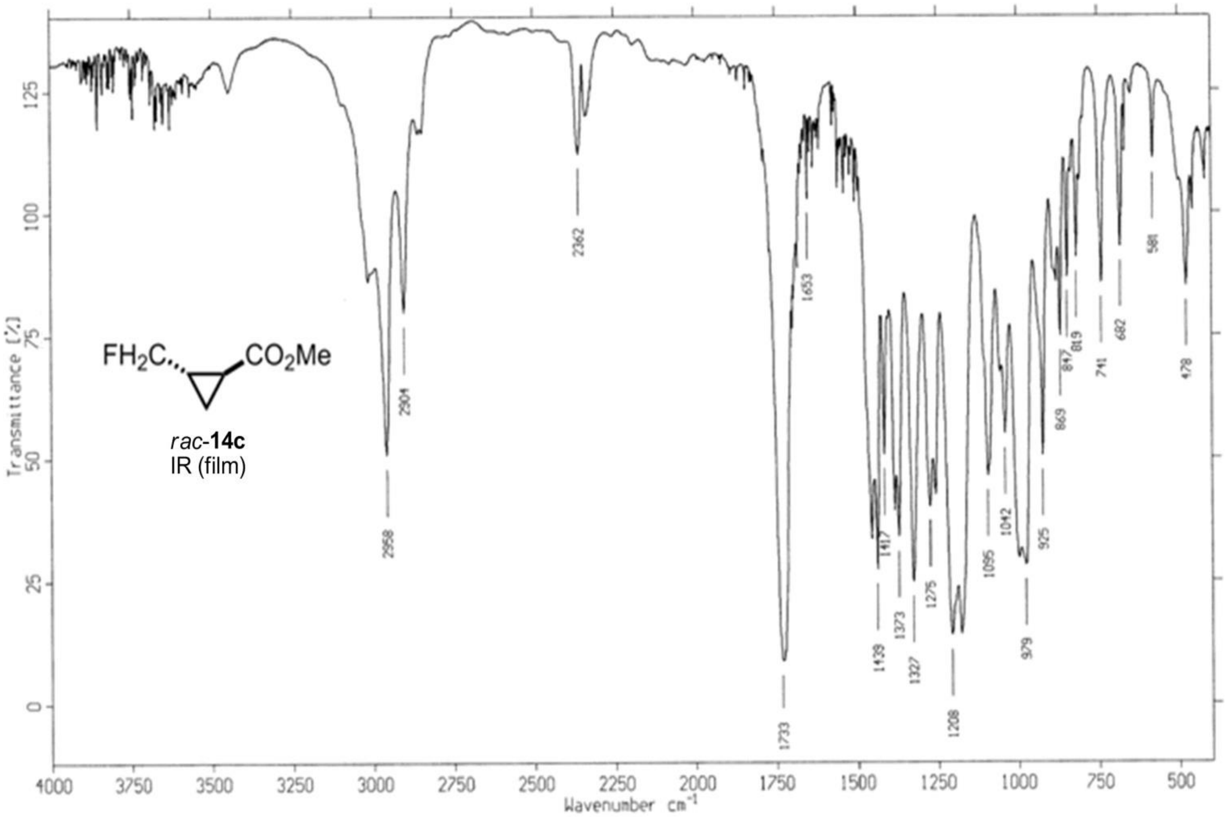
FH2C CO2Me
rac-14c
IR (film)
Transmittance [%]
Wavenumber cm-1
2958
2904
2362
1733
1653
1439
1417
1373
1327
1275
1208
1095
1042
979
925
869
847
819
741
682
581
478

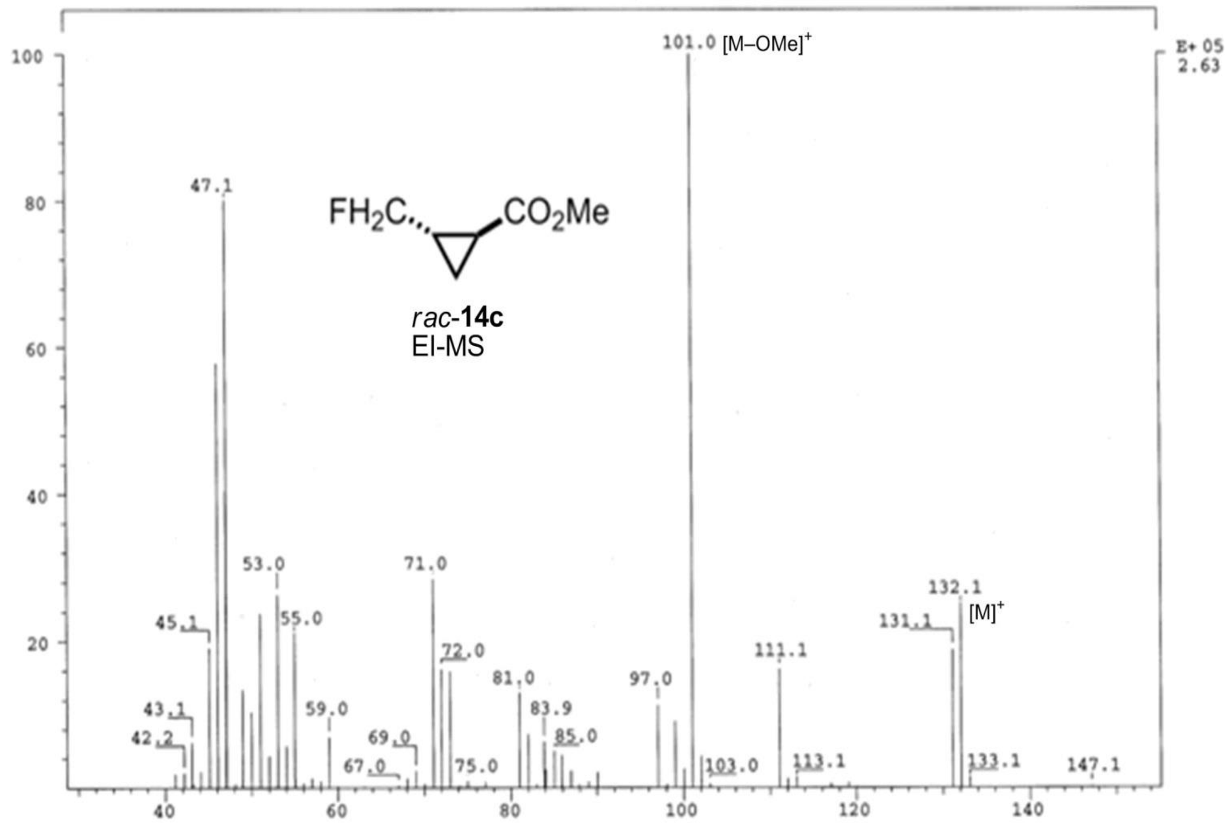
FH2C CO2Me
rac-14c
EI-MS
101.0 [M–OMe]+
E+ 05
2.63
47.1
53.0
71.0
132.1
[M]+
131.1
45.1
55.0
72.0
111.1
81.0
97.0
43.1
59.0
83.9
42.2
69.0
85.0
67.0
75.0
103.0
113.1
133.1
147.1

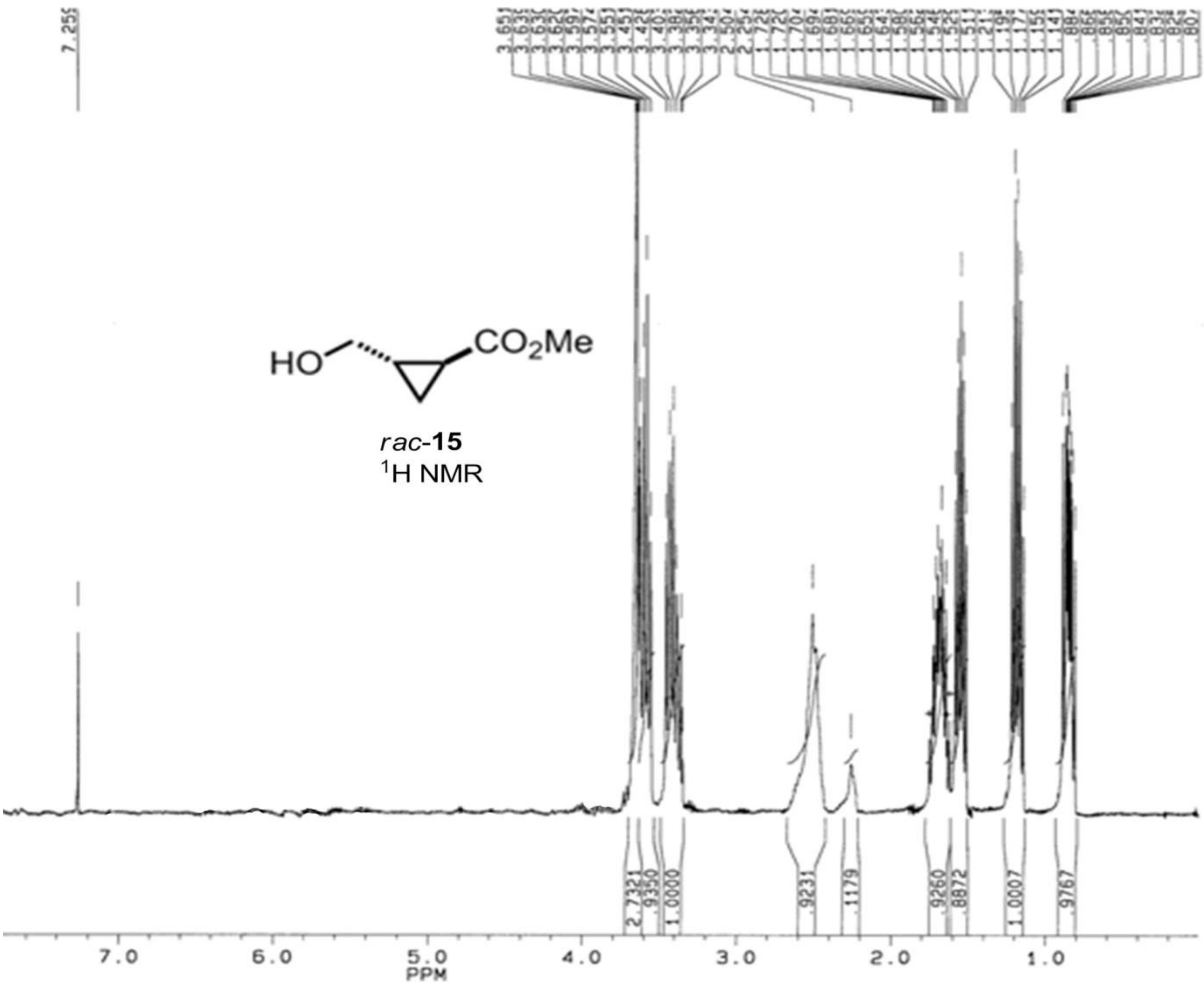
7.255
HO
CO2Me
rac-15
1H NMR
2.7321
.9350
1.0000
.9231
.1179
.9260
.8872
1.0007
.9767
7.0
6.0
5.0
PPM
4.0
3.0
2.0
1.0

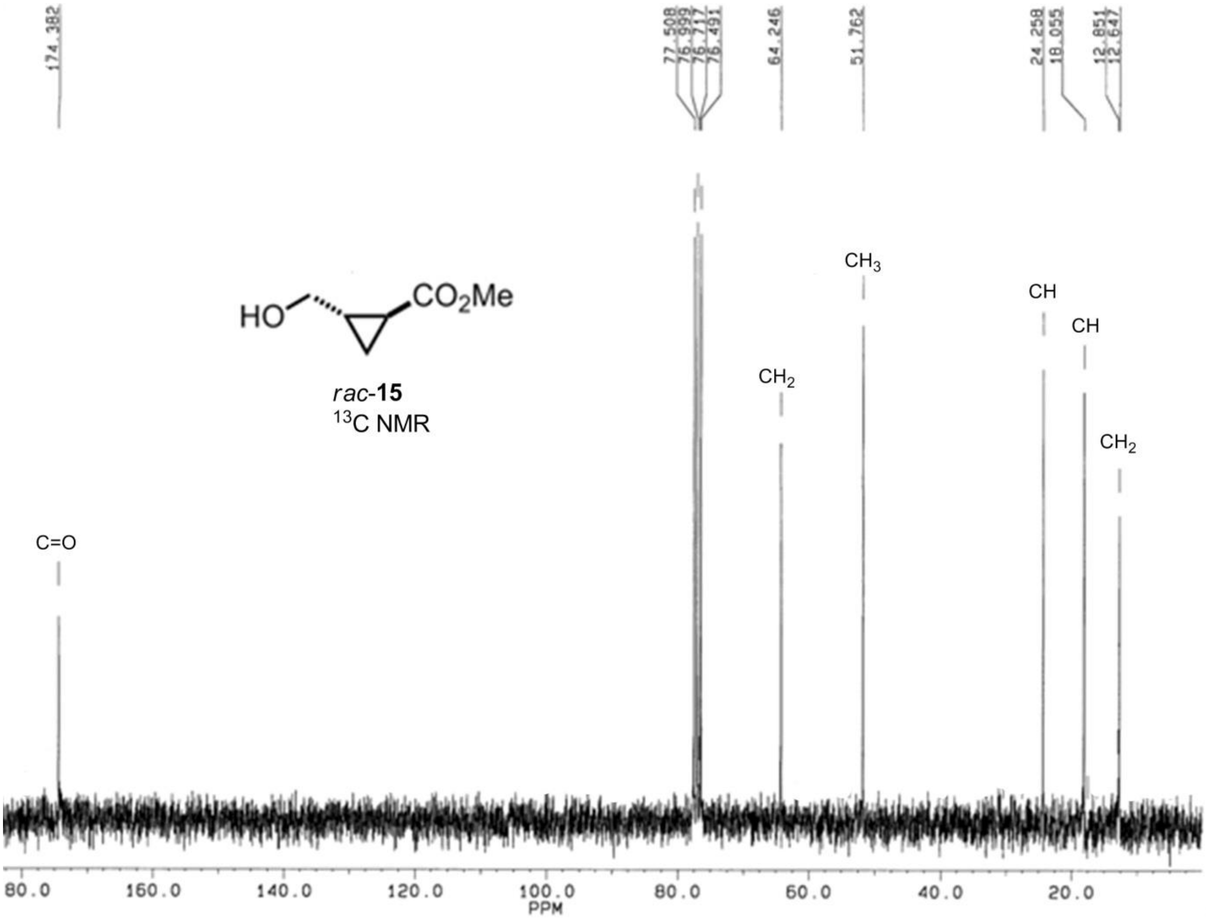
174.382
77.508
76.999
76.717
76.491
64.246
51.762
24.258
18.055
12.851
12.647
HO
CO2Me
rac-15
13C NMR
C=O
CH2
CH3
CH
CH
CH2
80.0
160.0
140.0
120.0
100.0
PPM
80.0
60.0
40.0
20.0

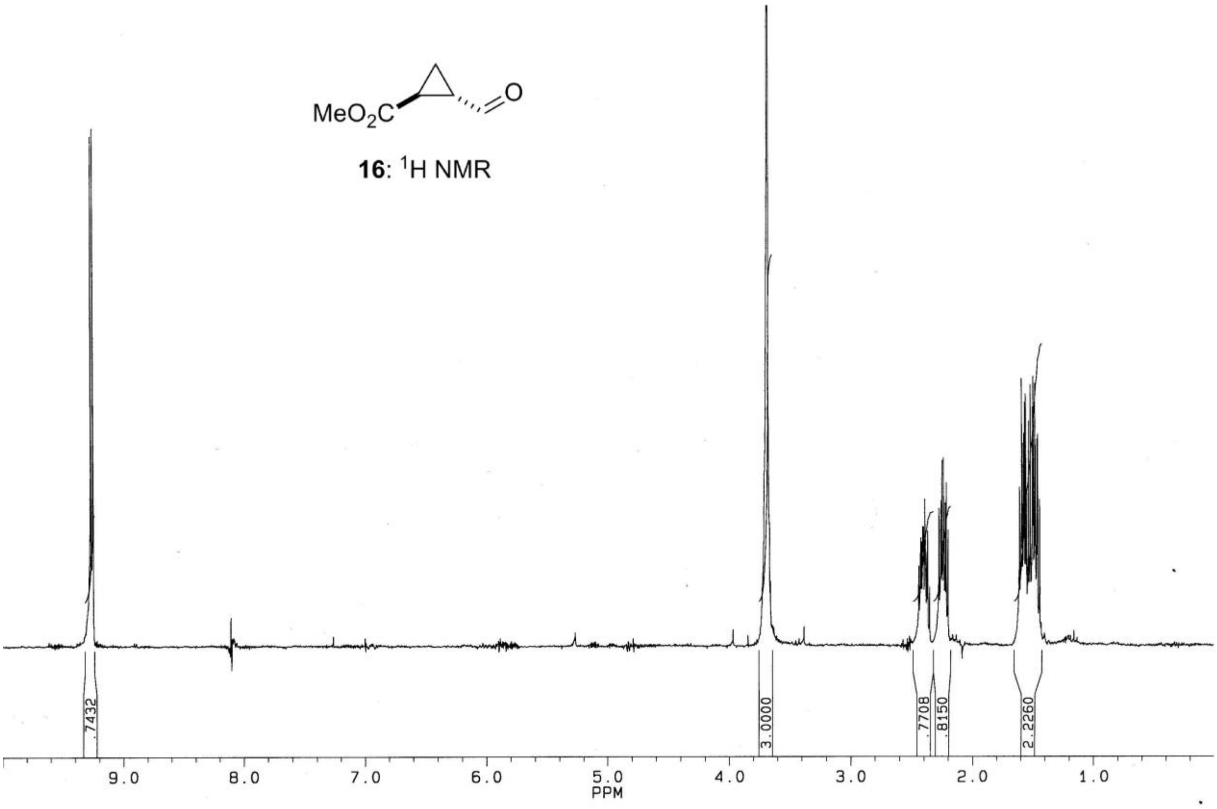
MeO2C
O
16: 1H NMR
.7432
3.0000
.7708
.8150
2.2260
9.0
8.0
7.0
6.0
5.0
PPM
4.0
3.0
2.0
1.0

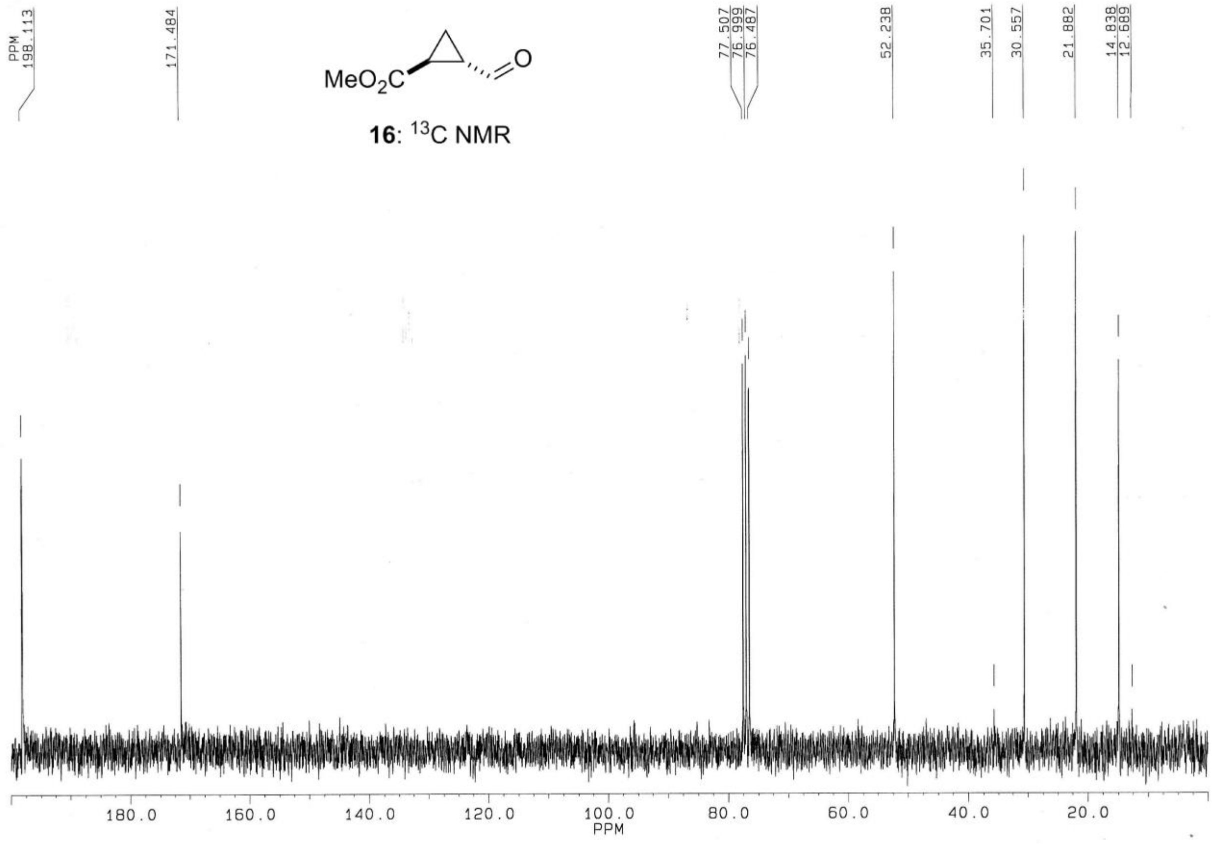
PPM
198.113
171.484
77.507
76.999
76.487
52.238
35.701
30.557
21.882
14.838
12.689
MeO2C
O
16: 13C NMR
180.0
160.0
140.0
120.0
100.0
PPM
80.0
60.0
40.0
20.0

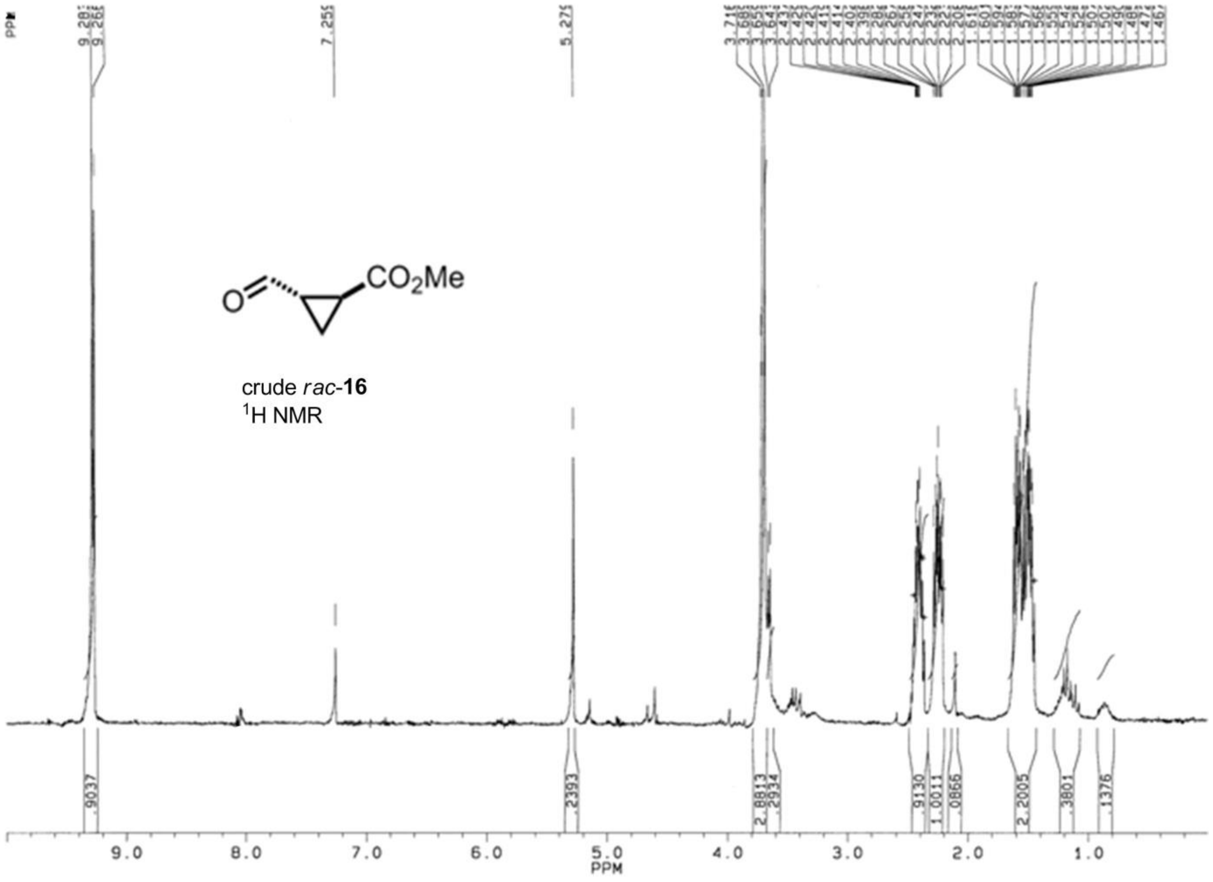
CO2Me
crude rac-16
1H NMR
PPM

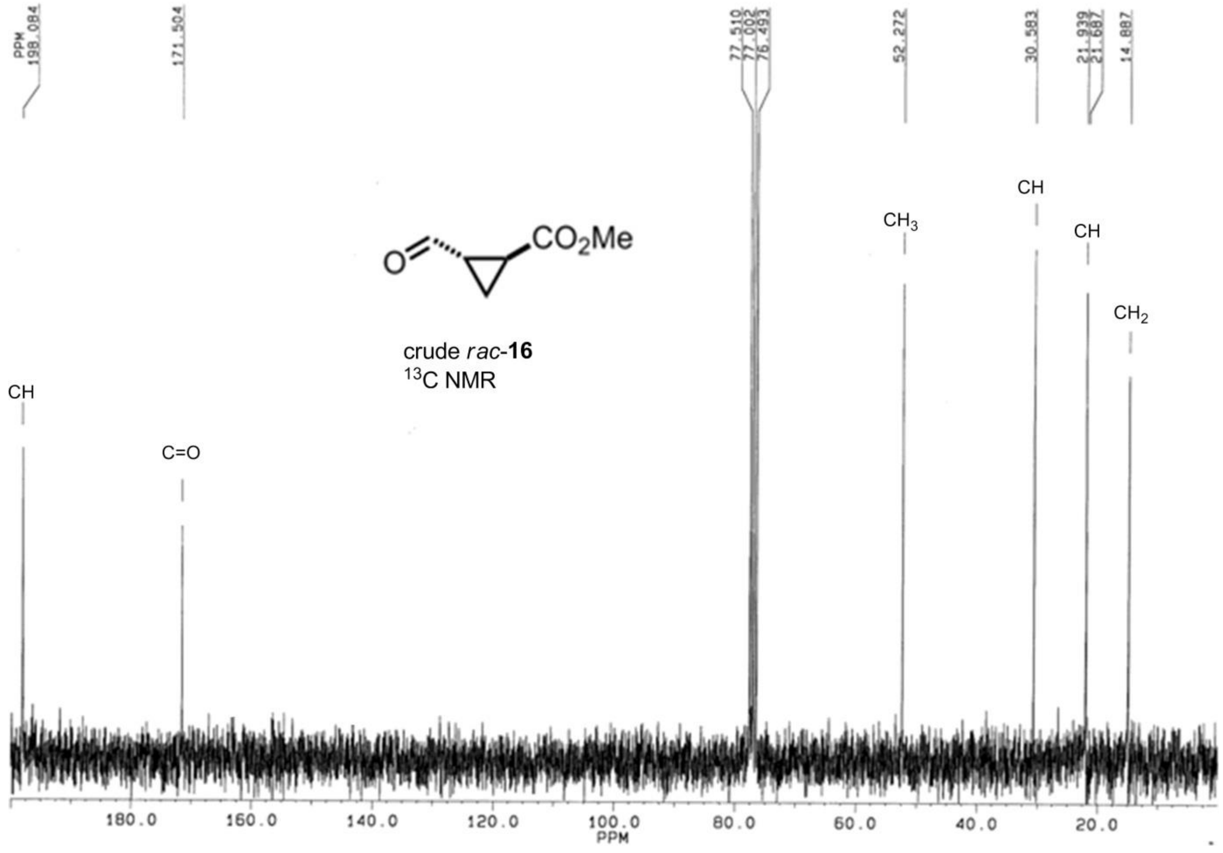
CO2Me
crude rac-16
13C NMR
CH
C=O
CH3
CH
CH
CH2
PPM

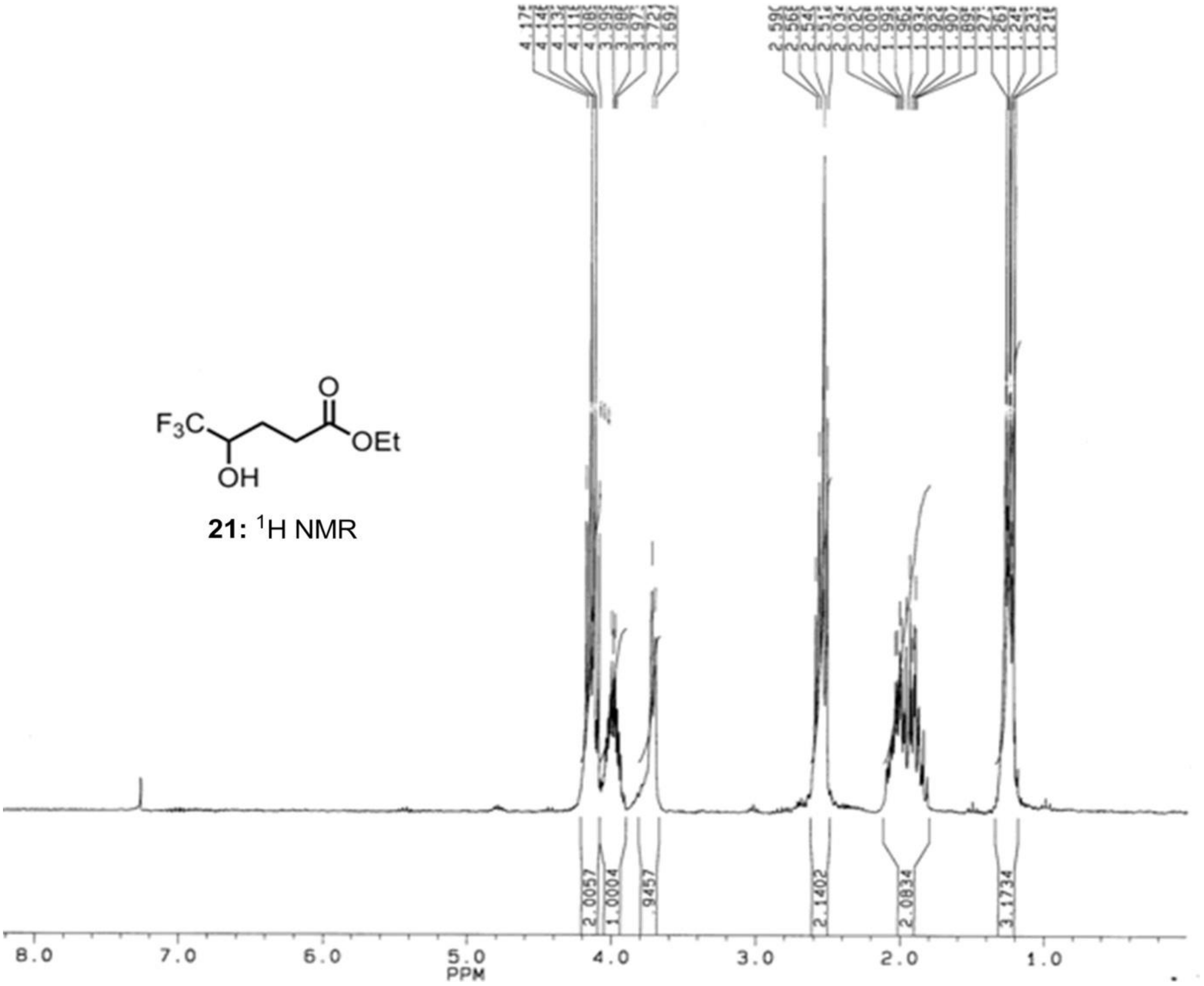

F3C
O
OEt
OH
21: 1H NMR
2.0057
1.0004
.9457
2.1402
2.0834
3.1734
8.0
7.0
6.0
5.0
PPM
4.0
3.0
2.0
1.0

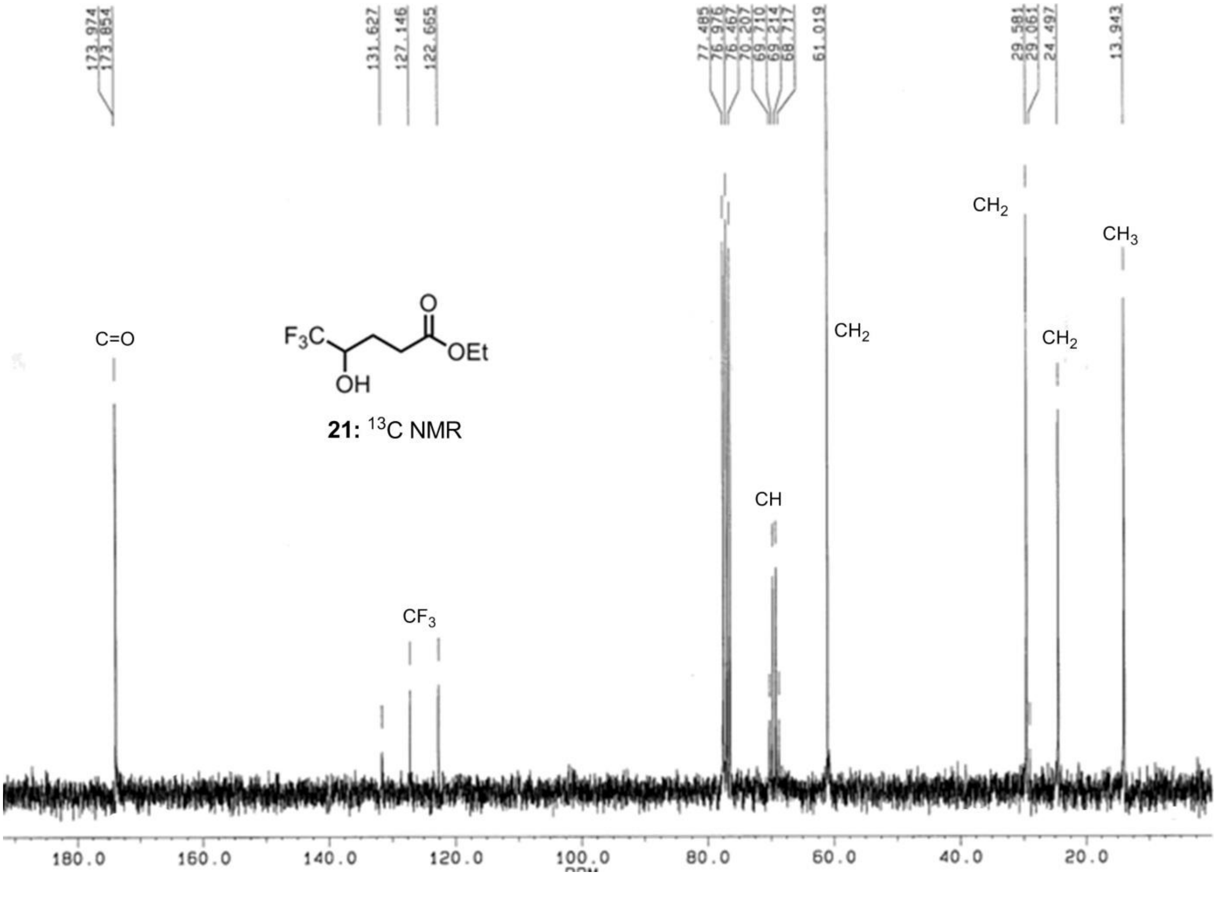

173.974
173.854
131.627
127.146
122.665
77.485
76.976
76.467
70.267
69.719
69.214
68.717
61.019
29.581
29.061
24.497
13.943
C=O
F3C
O
OEt
OH
21: 13C NMR
CF3
CH
CH2
CH2
CH2
CH3
180.0
160.0
140.0
120.0
100.0
80.0
60.0
40.0
20.0

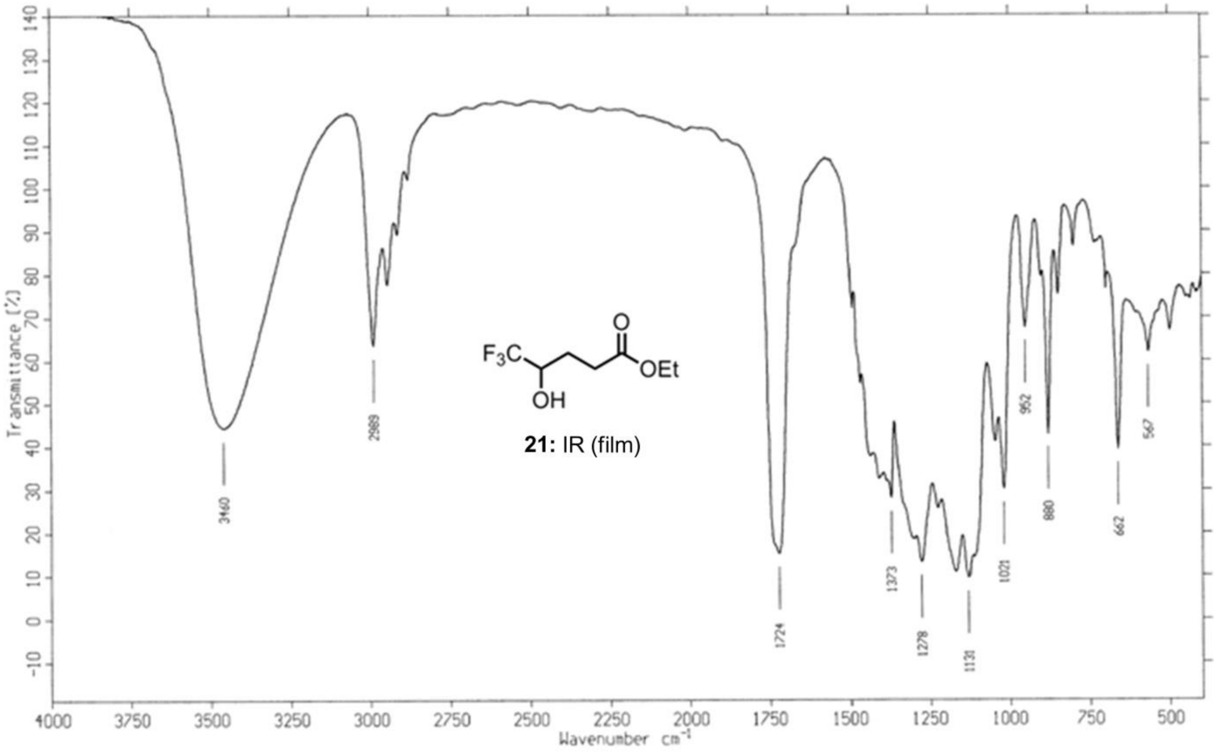
Transmittance [%]
Wavenumber cm⁻¹
3460
2989
1724
1373
1278
1131
1021
952
880
662
567
F_3C
OH
O
OEt
21: IR (film)

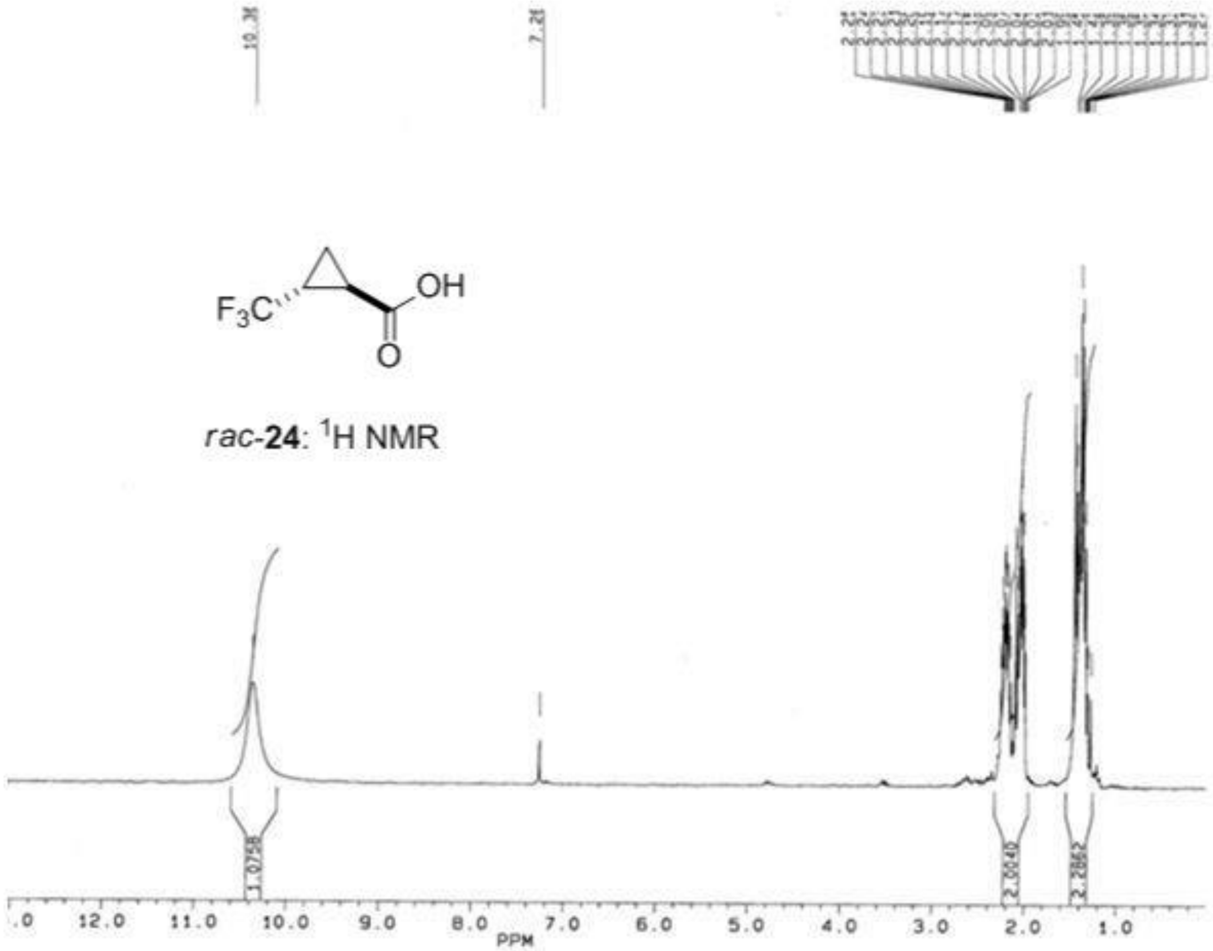
10.36
7.26
F_3C
OH
O
rac-24: ^{1}H NMR
1.0758
2.0040
2.2862
PPM

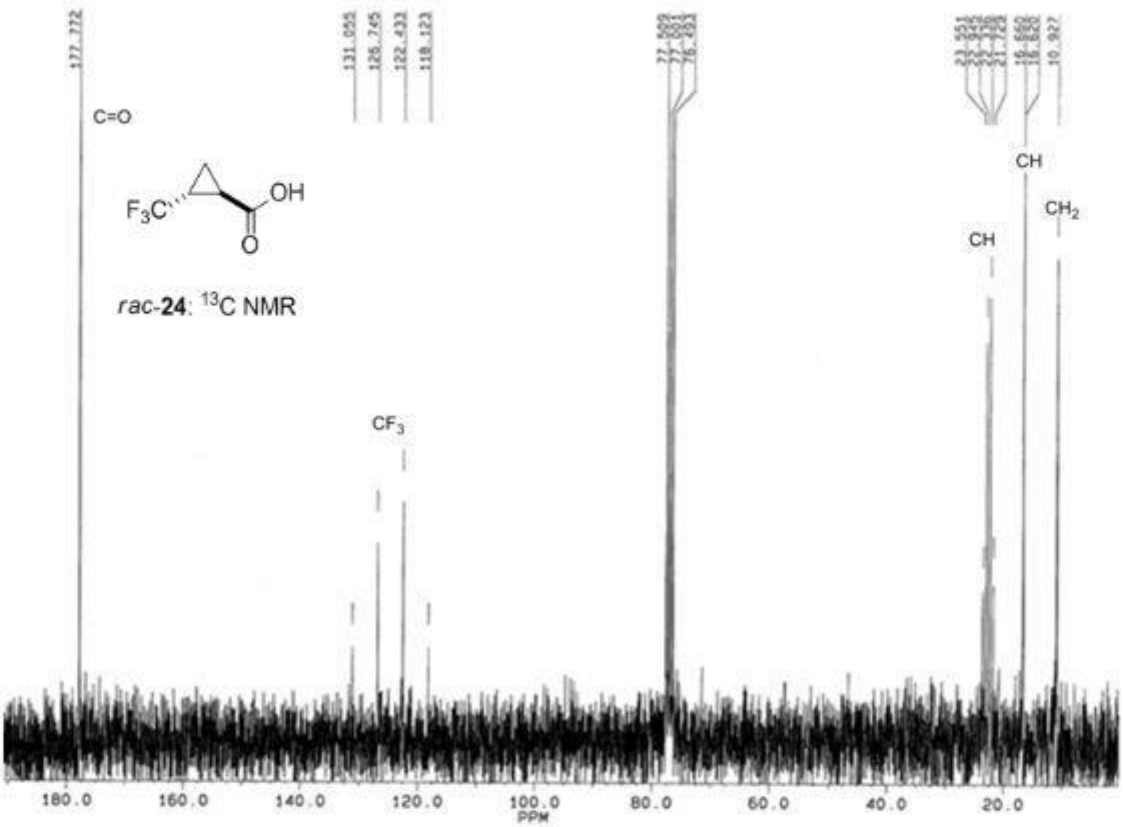

177.772
131.055
126.745
122.433
118.123
C=O
OH
F3C
O
rac-24: 13C NMR
CF3
CH
CH2
CH
10.927
180.0
160.0
140.0
120.0
100.0
PPM
80.0
60.0
40.0
20.0

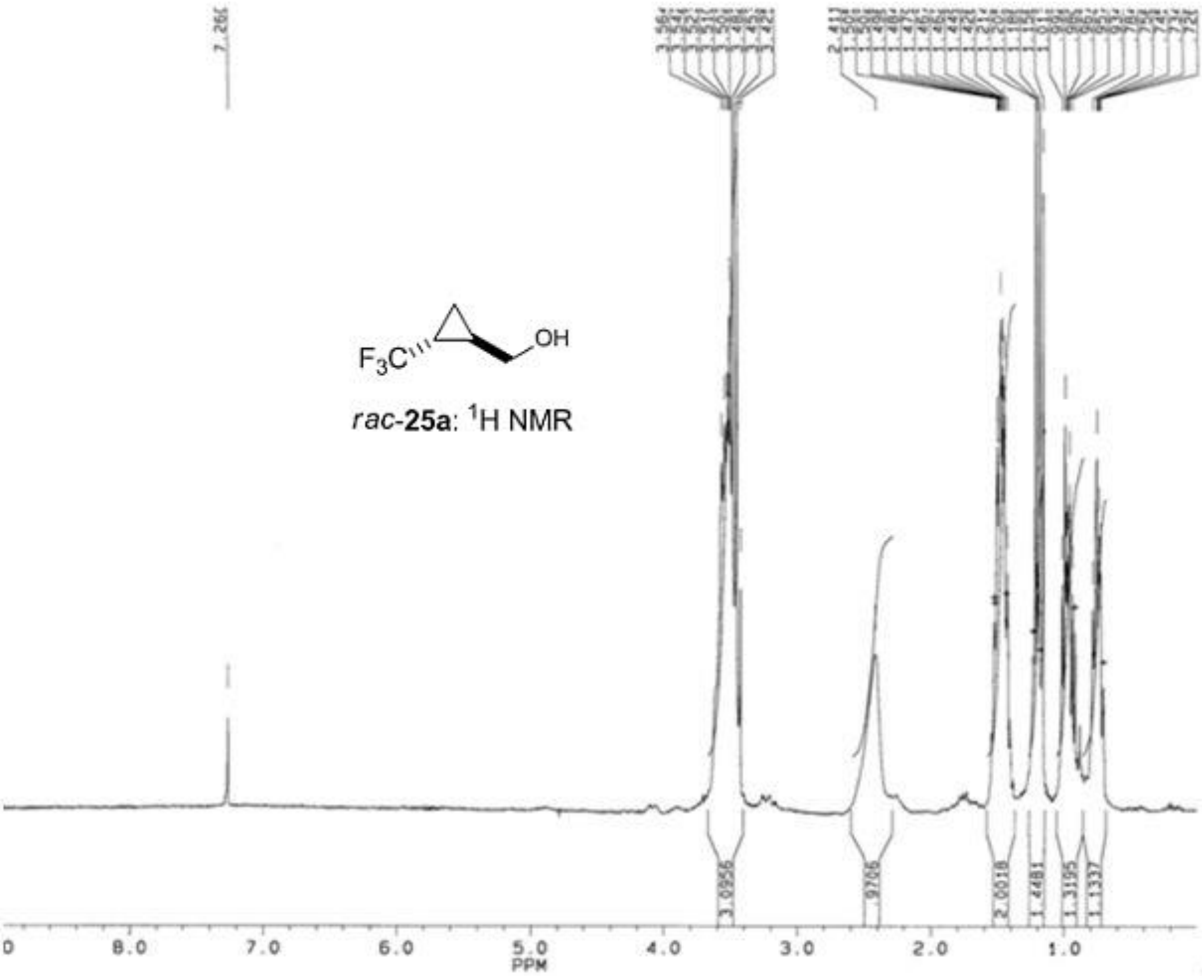

7.260
F3C
OH
rac-25a: 1H NMR
3.0956
9706
2.0018
1.4481
1.3195
1.1337
8.0
7.0
6.0
5.0
PPM
4.0
3.0
2.0
1.0

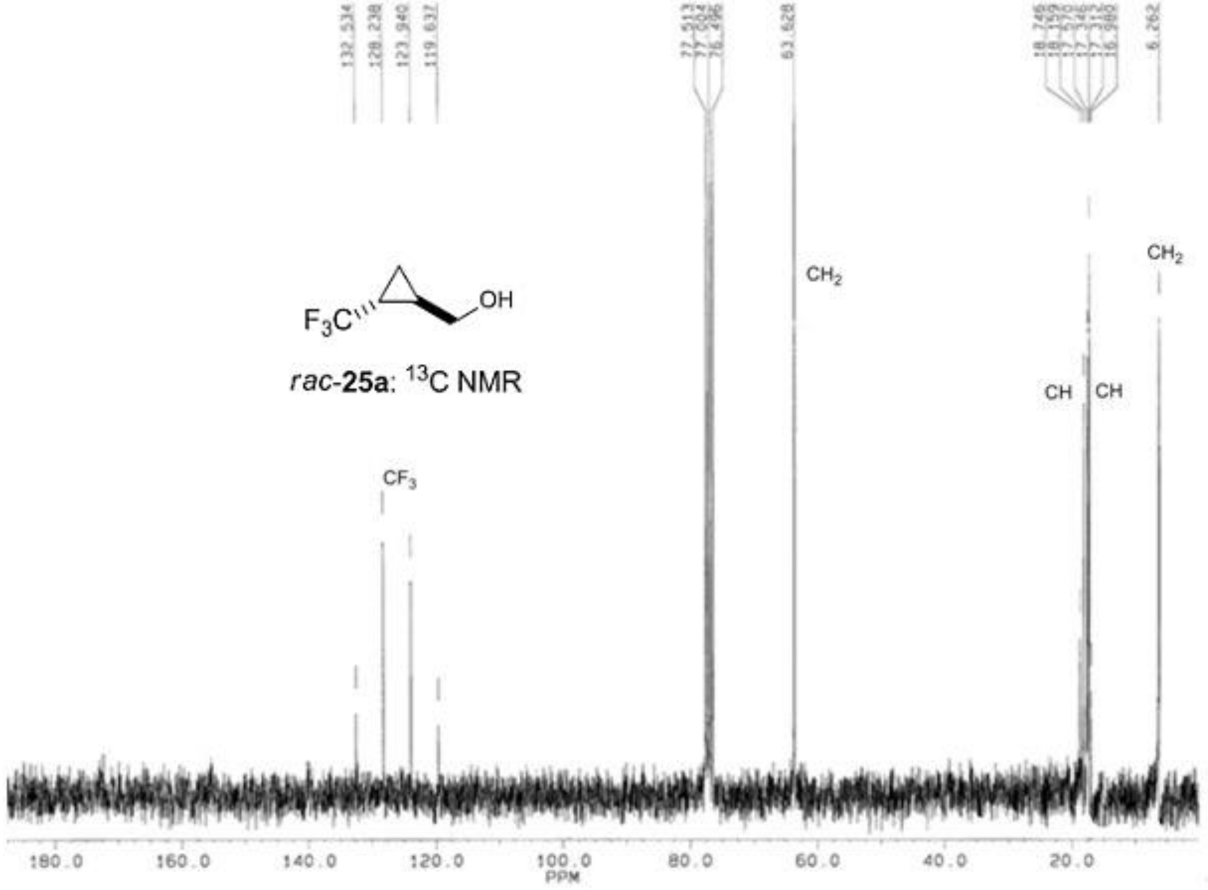
132.534
128.238
123.940
119.637
77.513
77.004
76.496
63.628
18.746
18.159
17.570
17.346
17.315
16.980
6.262
CH2
CH2
F_3C
OH
rac-**25a**: ^{13}C NMR
CH
CH
CF_3
180.0
160.0
140.0
120.0
100.0
PPM
80.0
60.0
40.0
20.0

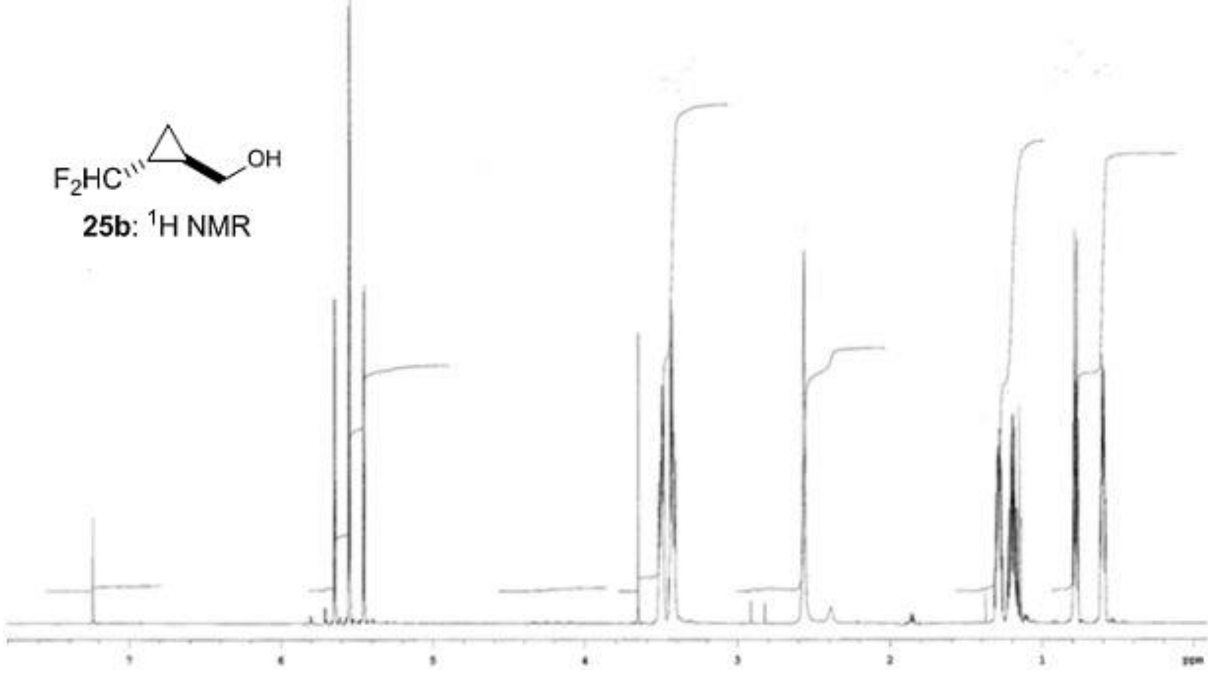
F_2HC
OH
25b: ^{1}H NMR

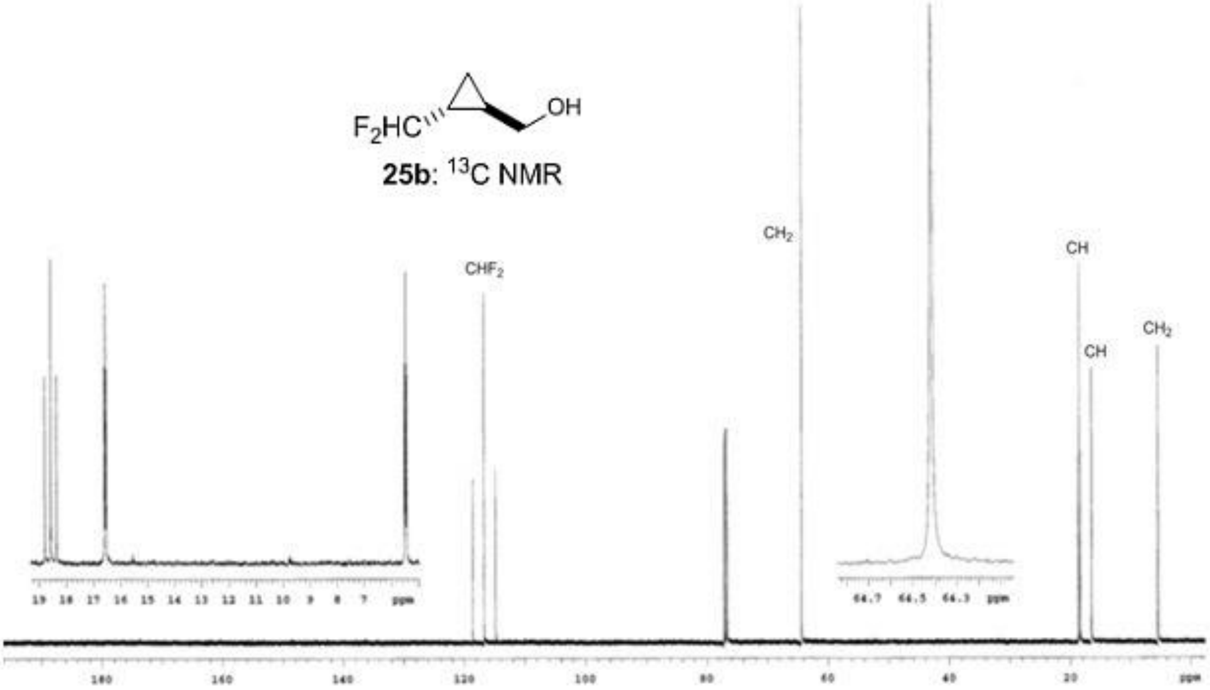
F2HC
OH
25b: 13C NMR
CHF2
CH2
CH
CH
CH2
ppm

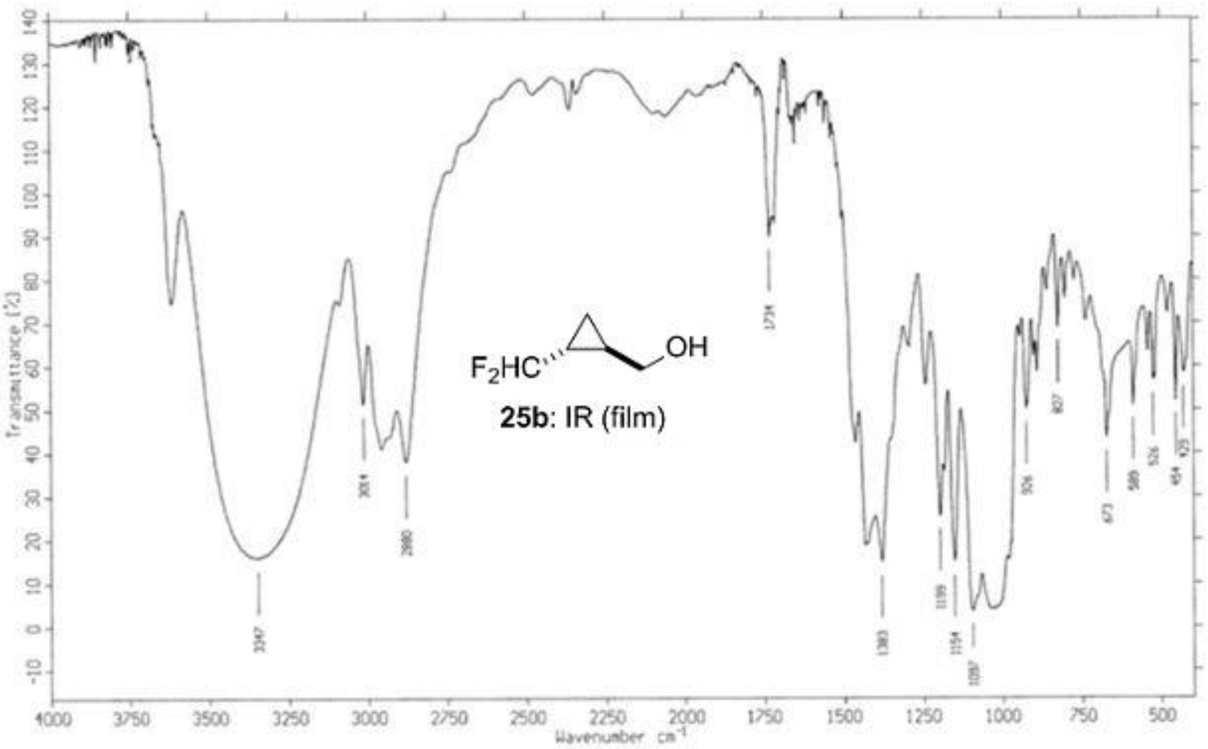
F2HC
OH
25b: IR (film)
Transmittance [%]
Wavenumber cm-1
3347
3004
2880
1724
1383
1195
1154
1097
926
837
673
585
526
454
425

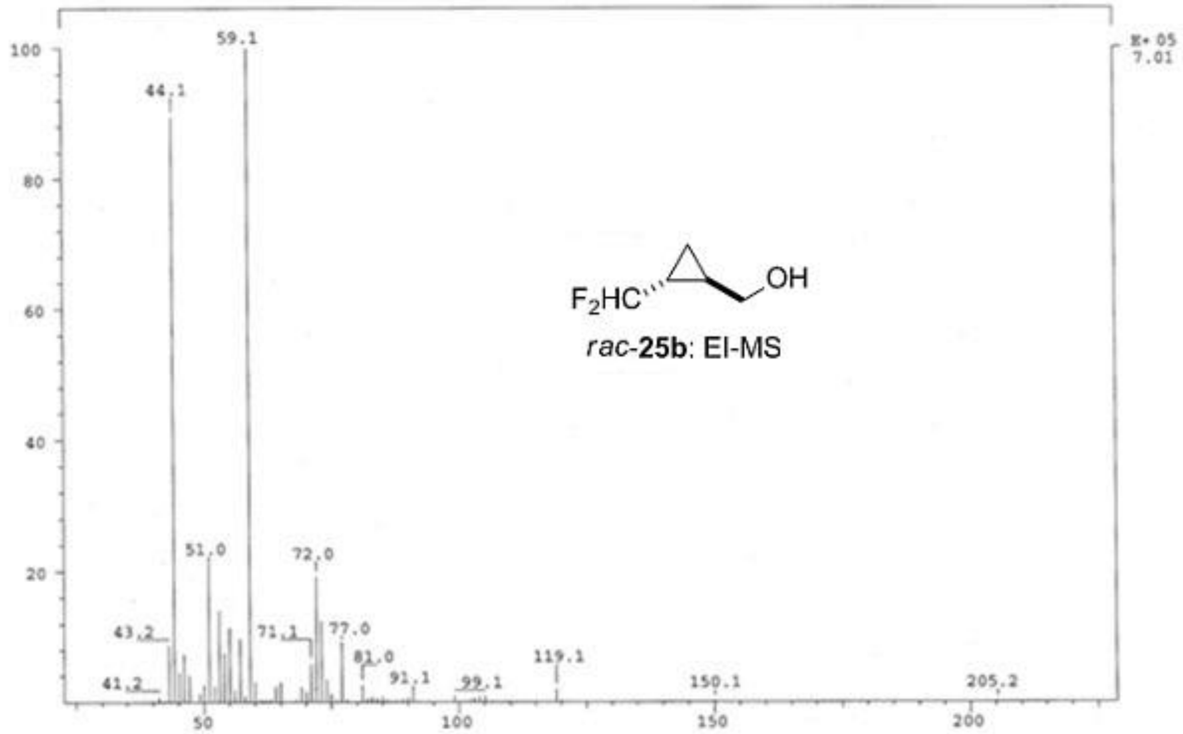

100
80
60
40
20
59.1
44.1
51.0
72.0
43.2
71.1
77.0
81.0
41.2
91.1
99.1
119.1
150.1
205.2
50
100
150
200
E+ 05
7.01
F2HC
OH
rac-25b: EI-MS

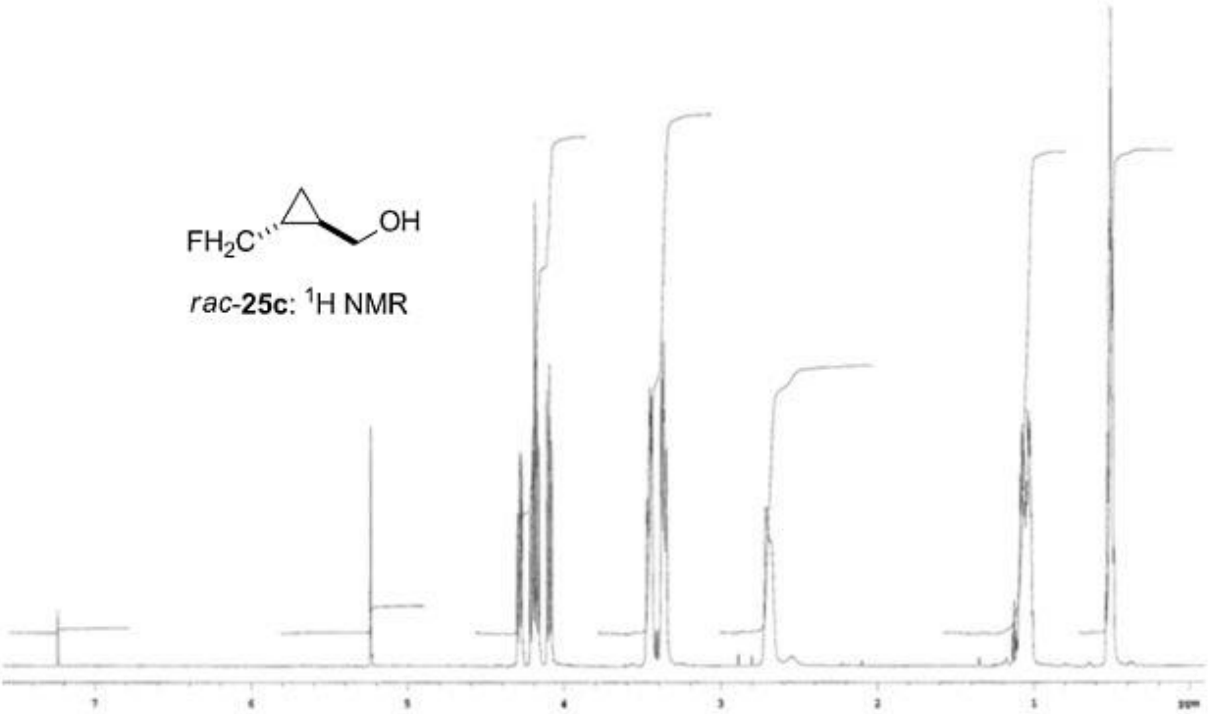

FH2C
OH
rac-25c: 1H NMR

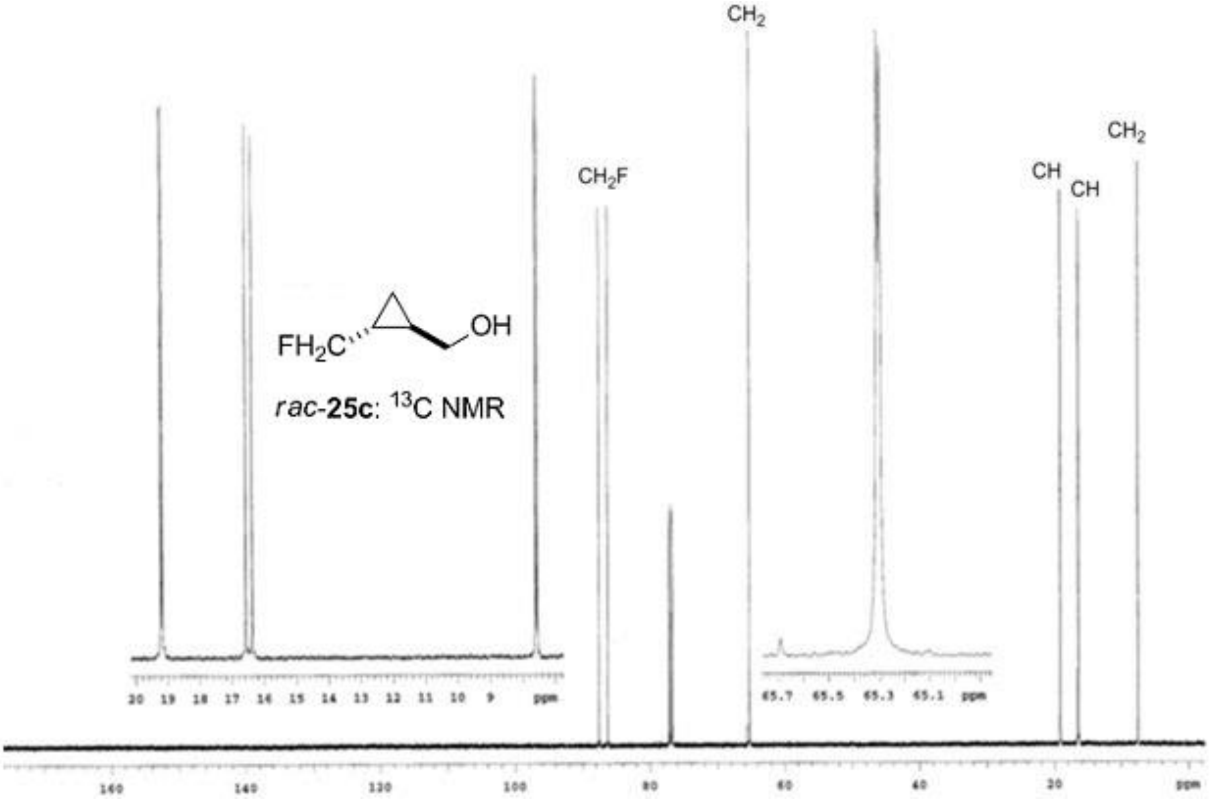
CH2
CH2F
CH
CH
CH2
FH2C
OH
rac-25c: 13C NMR
ppm
ppm
ppm

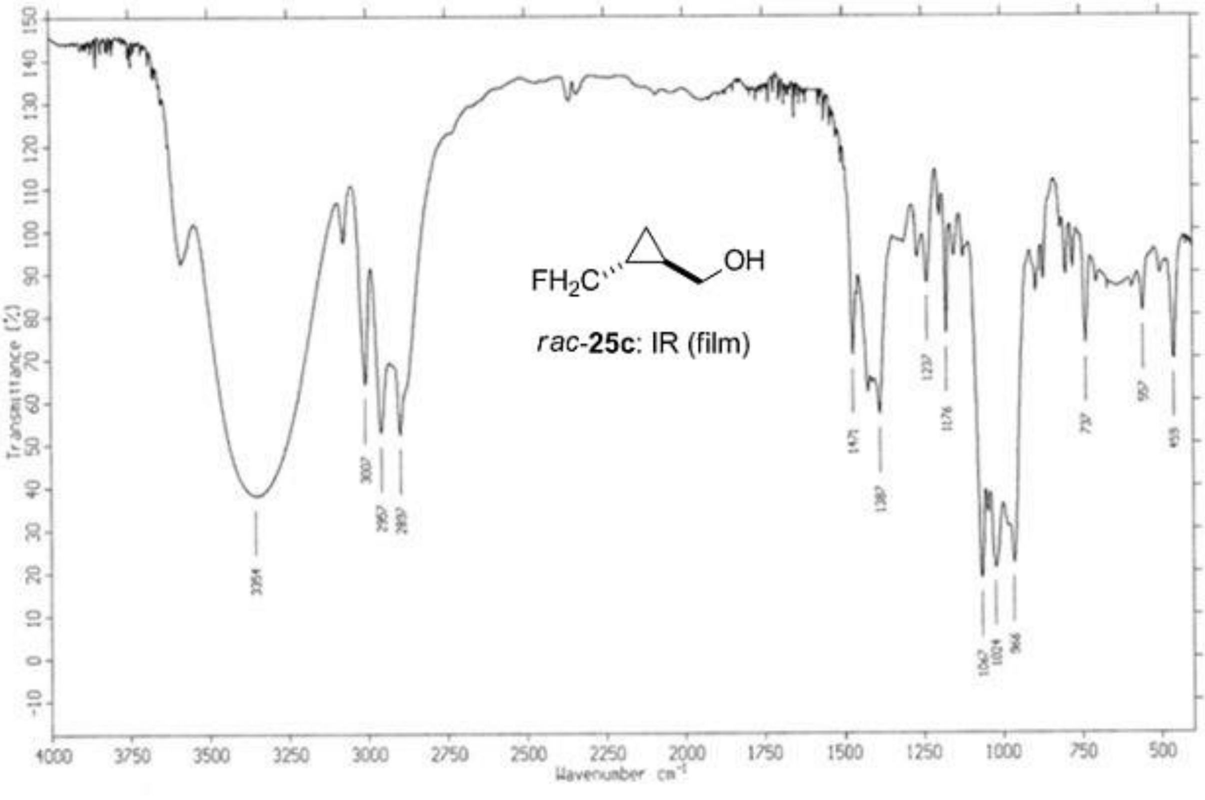
FH2C
OH
rac-25c: IR (film)
Transmittance (%)
Wavenumber cm-1
3354
3007
2957
2897
1471
1387
1237
1176
1067
1024
968
737
557
459

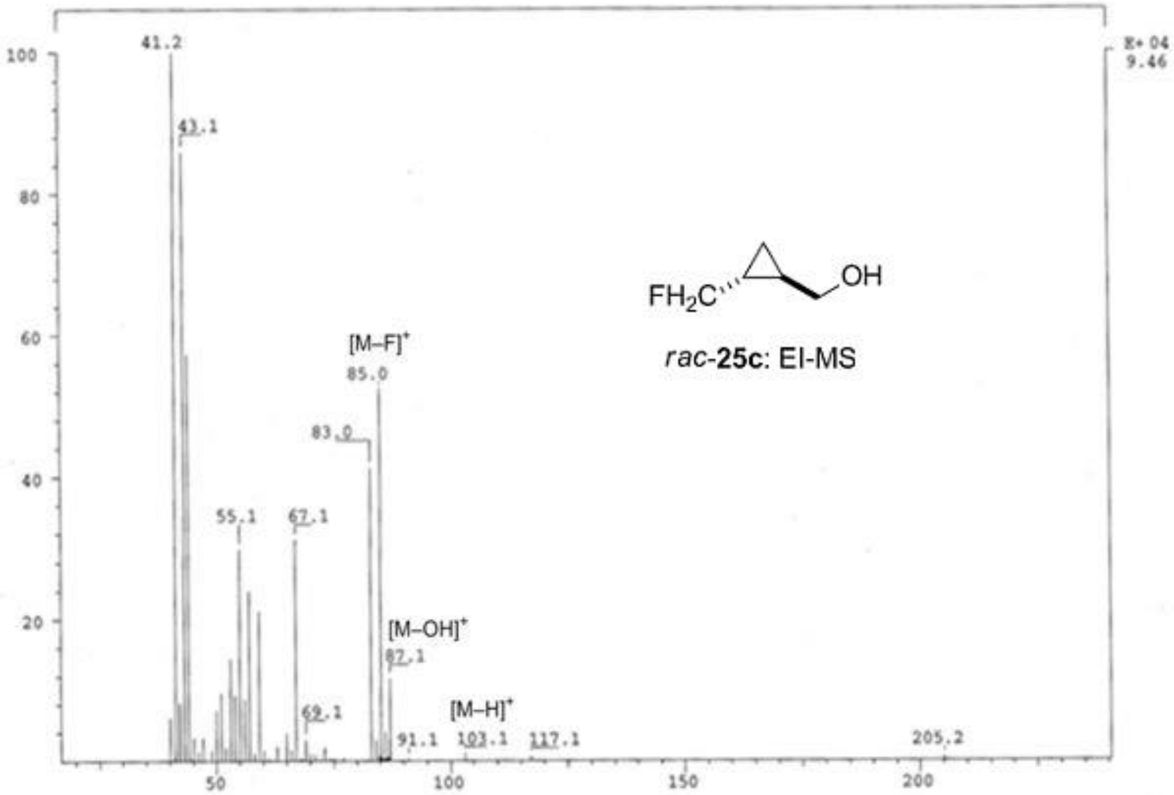

rac-25c: EI-MS
FH2C
OH
[M–F]+
85.0
83.0
41.2
43.1
55.1
67.1
[M–OH]+
87.1
69.1
[M–H]+
91.1
103.1
117.1
205.2
E+ 04
9.46
100
80
60
40
20
50
100
150
200

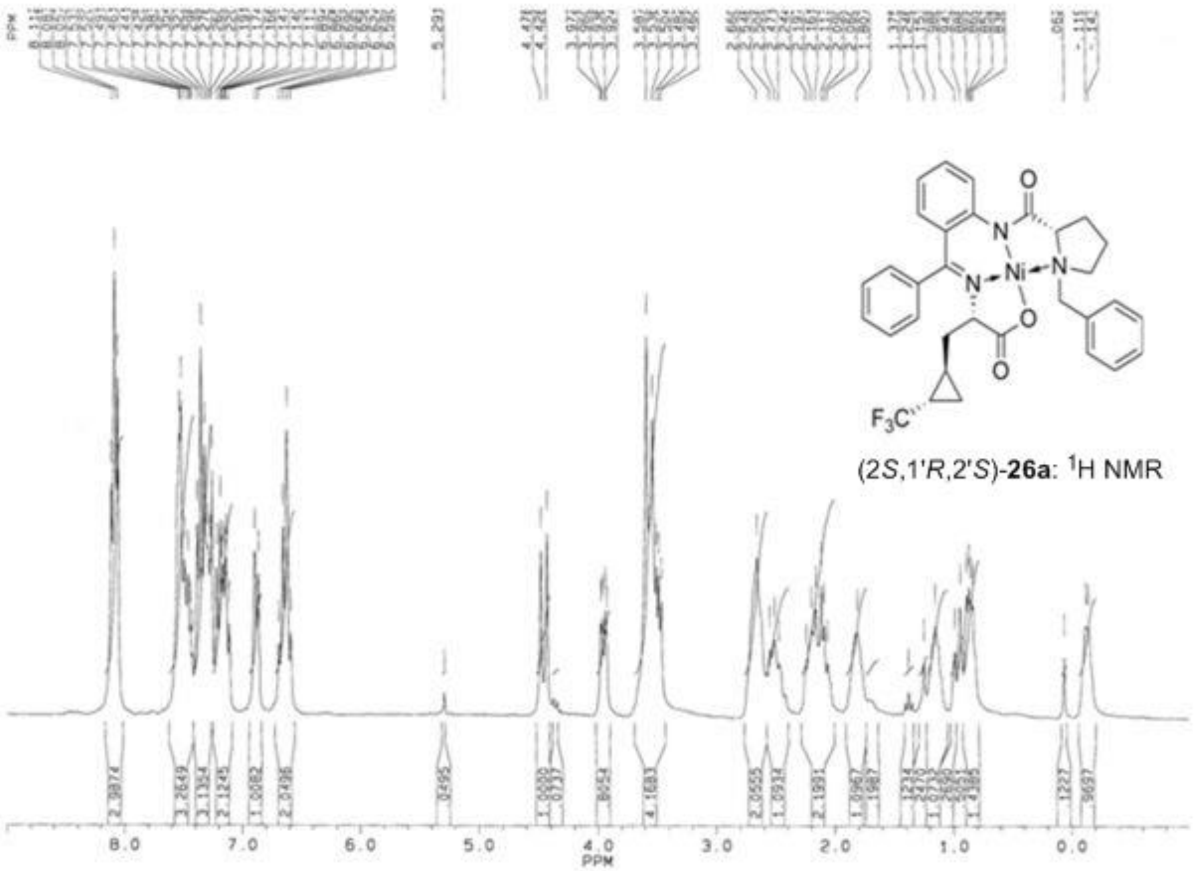

(2S,1'R,2'S)-26a: 1H NMR
F3C
Ni
N
O
PPM
8.0
7.0
6.0
5.0
4.0
3.0
2.0
1.0
0.0

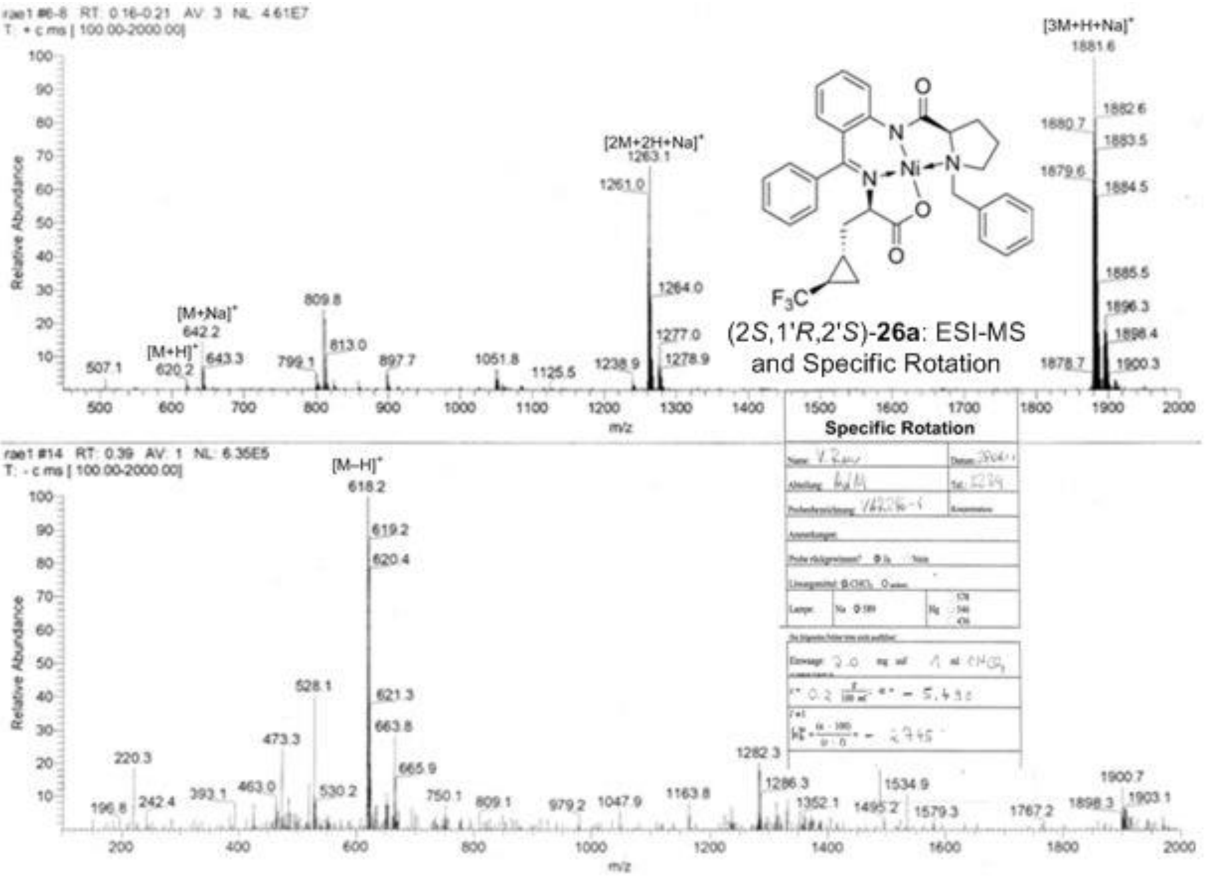

(2S,1'R,2'S)-**26a**: ESI-MS and Specific Rotation

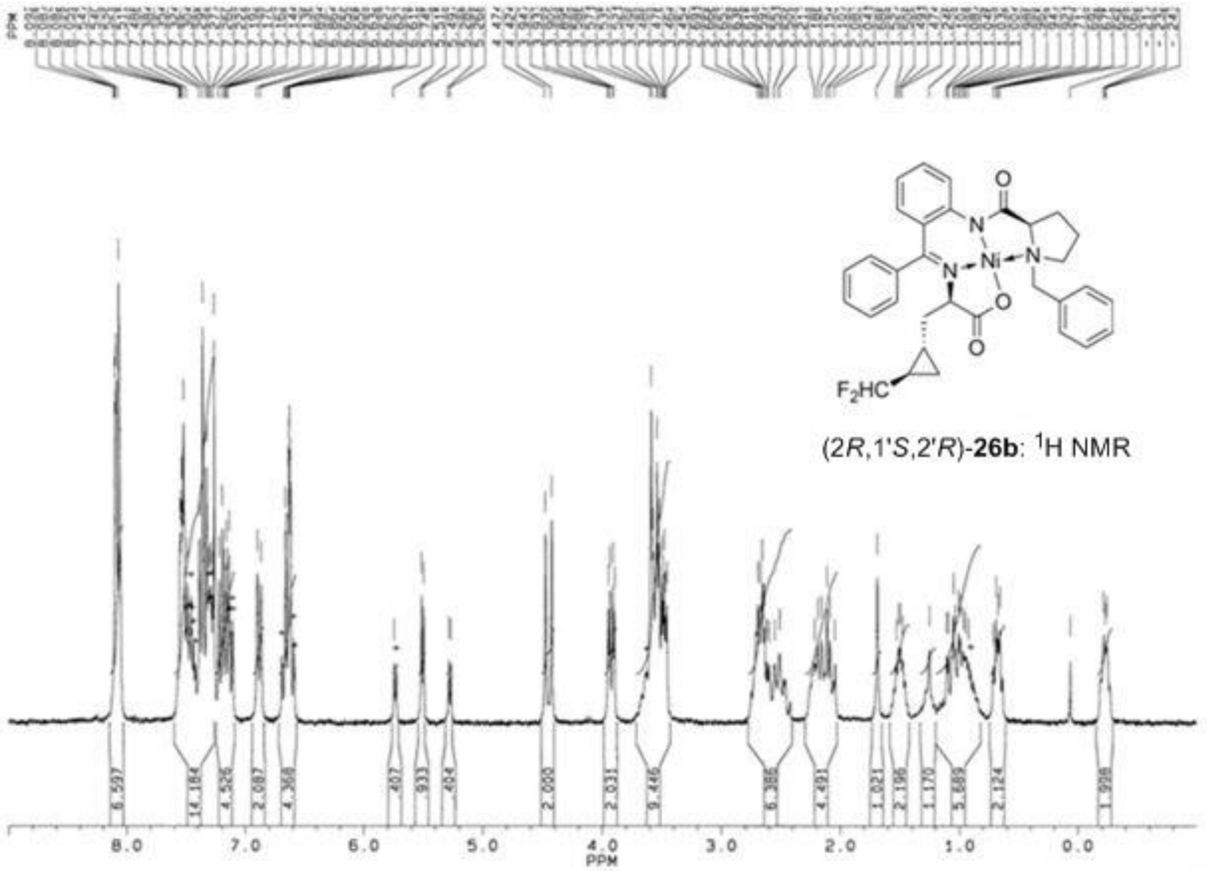

(2R,1'S,2'R)-**26b**: ^{1}H NMR

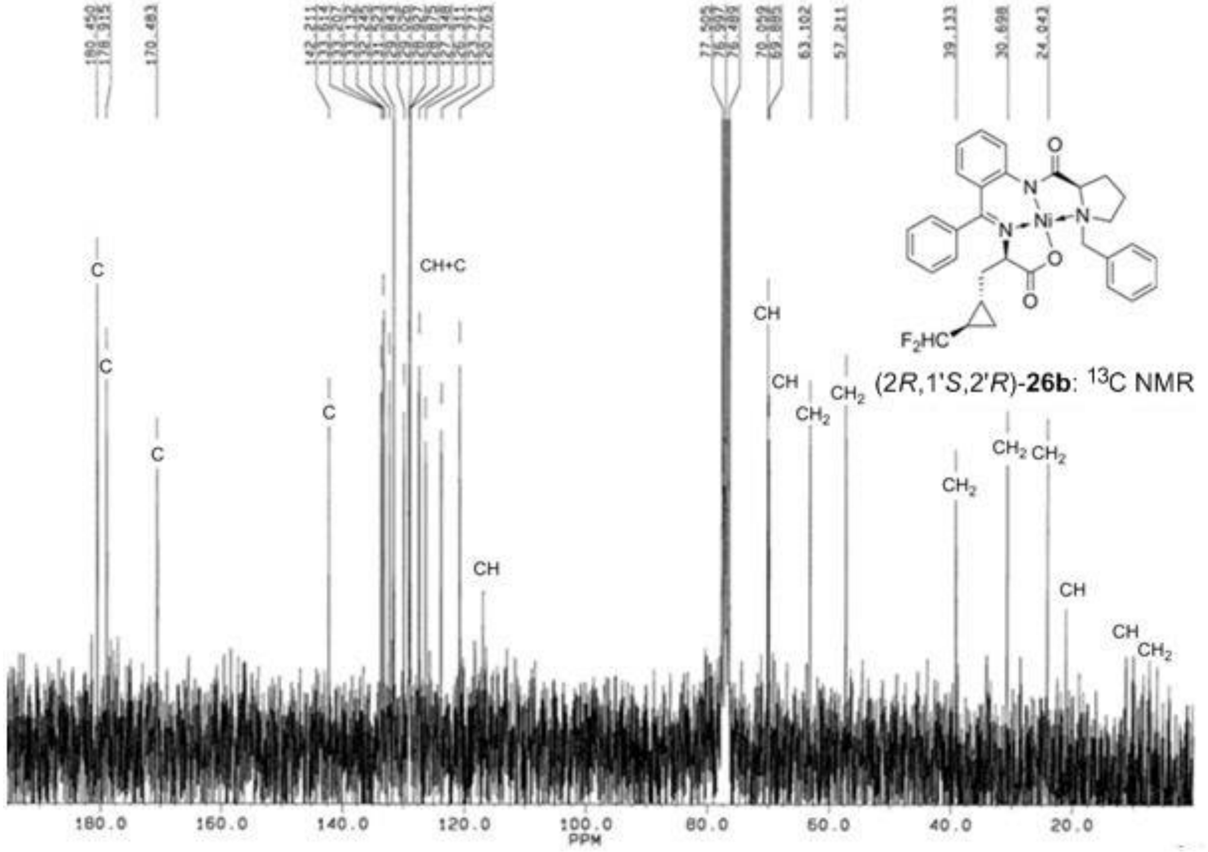
180.450
178.913
170.483
63.102
57.211
39.133
30.698
24.043
C
CH+C
CH
CH$_2$
(2*R*,1'*S*,2'*R*)-**26b**: ^{13}C NMR
180.0
160.0
140.0
120.0
100.0
PPM
80.0
60.0
40.0
20.0

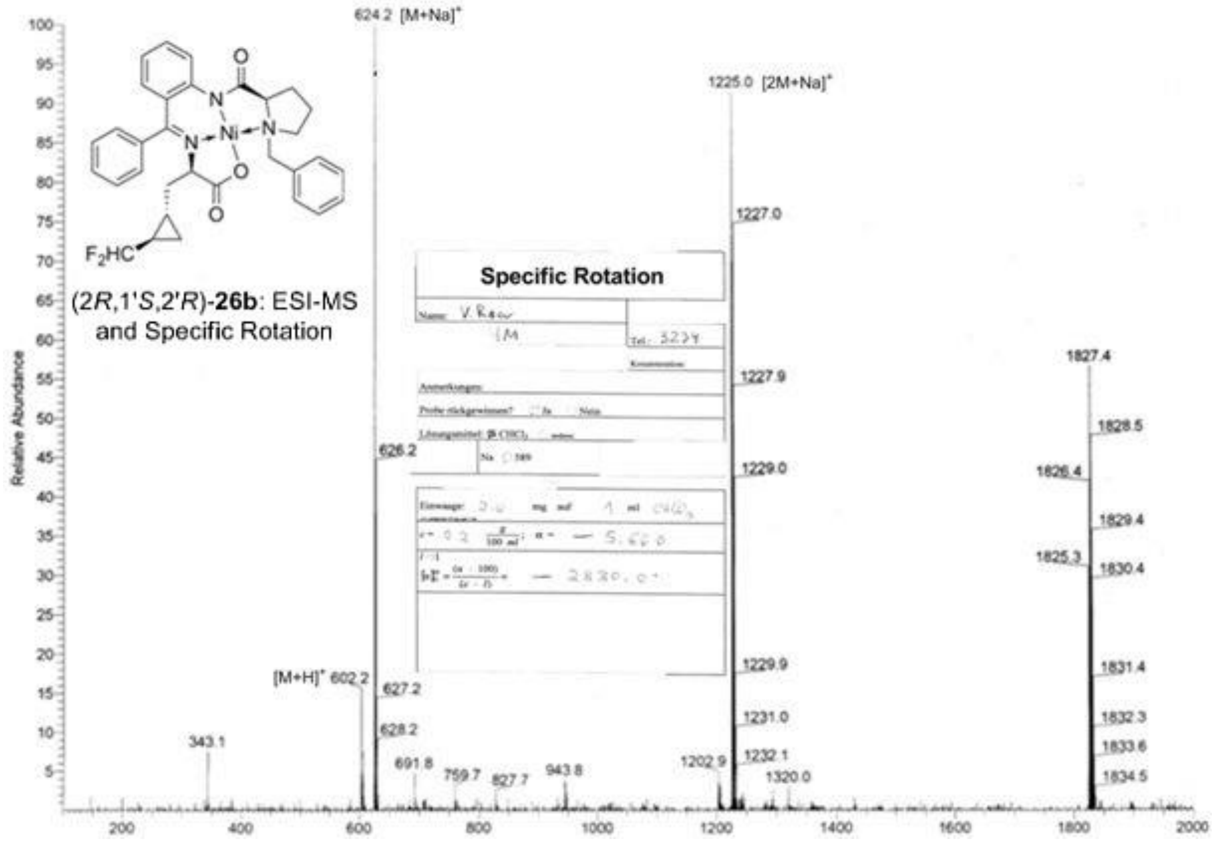
(2*R*,1'*S*,2'*R*)-**26b**: ESI-MS and Specific Rotation
624.2 [M+Na]$^+$
1225.0 [2M+Na]$^+$
[M+H]$^+$ 602.2
343.1
626.2
627.2
628.2
691.8
759.7
827.7
943.8
1202.9
1227.0
1227.9
1229.0
1229.9
1231.0
1232.1
1320.0
1827.4
1828.5
1826.4
1829.4
1825.3
1830.4
1831.4
1832.3
1833.6
1834.5
Relative Abundance
Specific Rotation

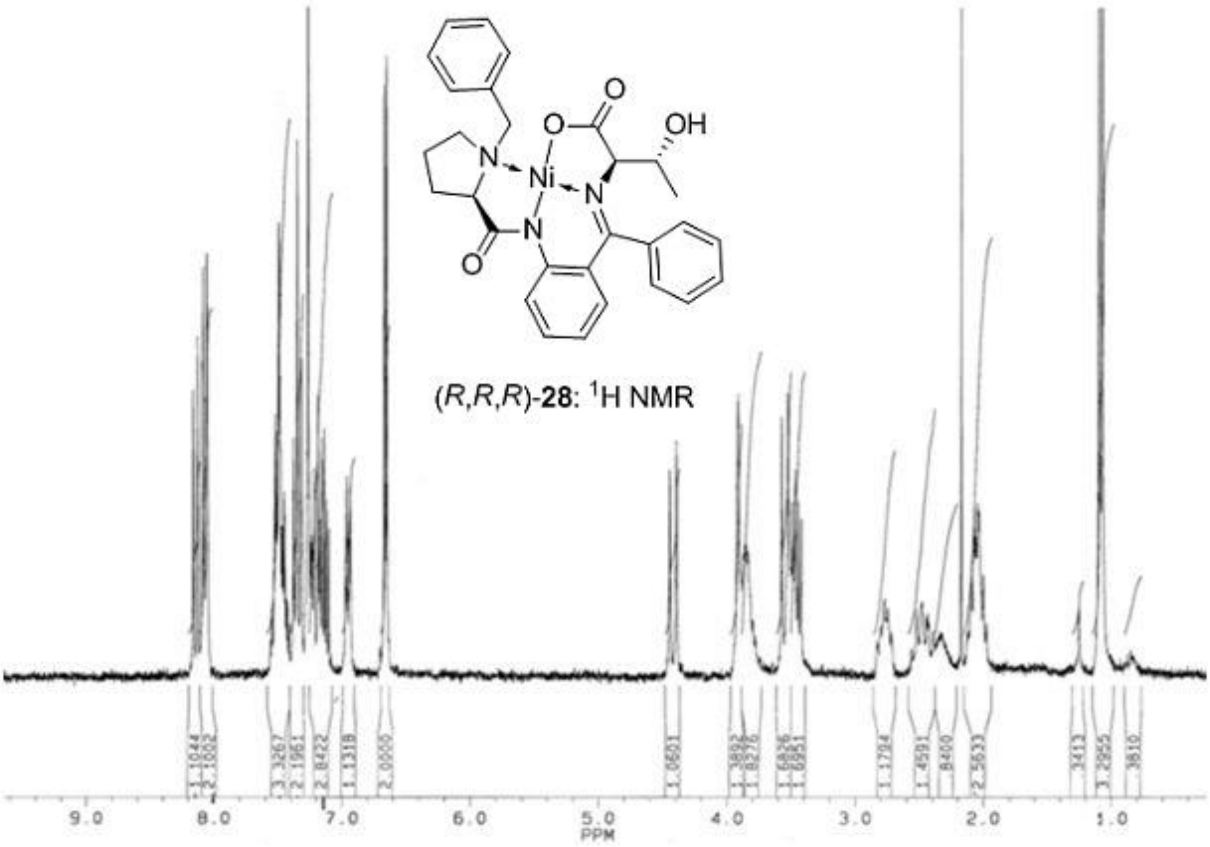
OH
O
Ni
N
(R,R,R)-28: 1H NMR
9.0
8.0
7.0
6.0
5.0
PPM
4.0
3.0
2.0
1.0

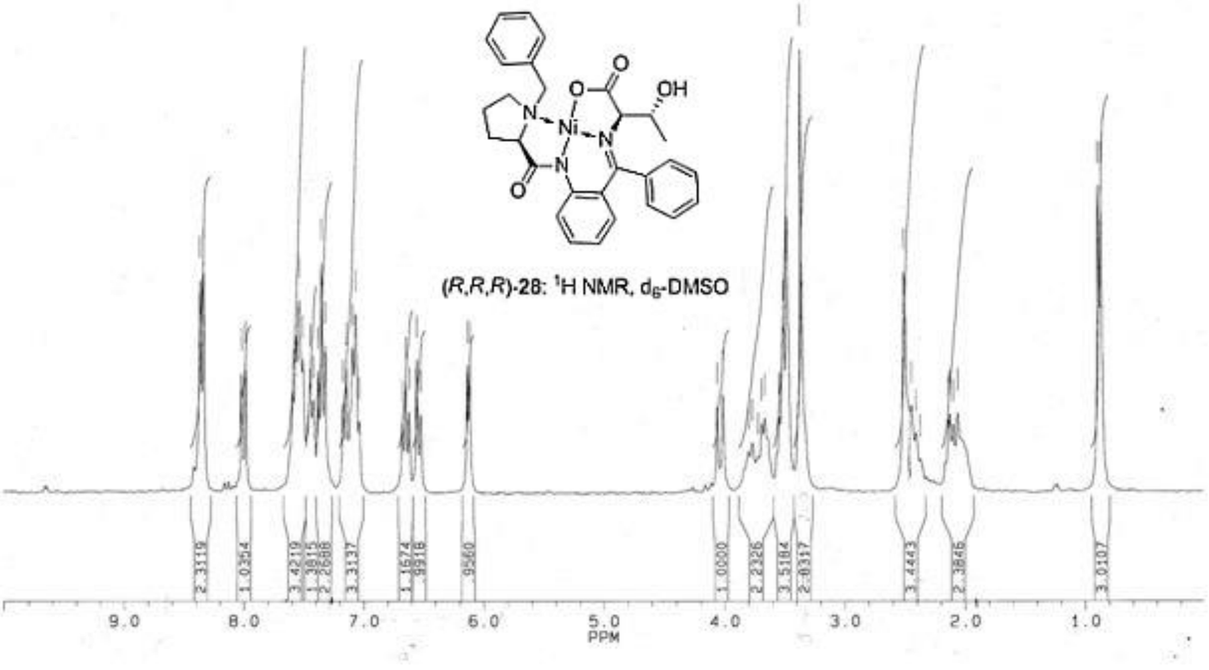
OH
O
Ni
N
(R,R,R)-28: 1H NMR, d6-DMSO
9.0
8.0
7.0
6.0
5.0
PPM
4.0
3.0
2.0
1.0

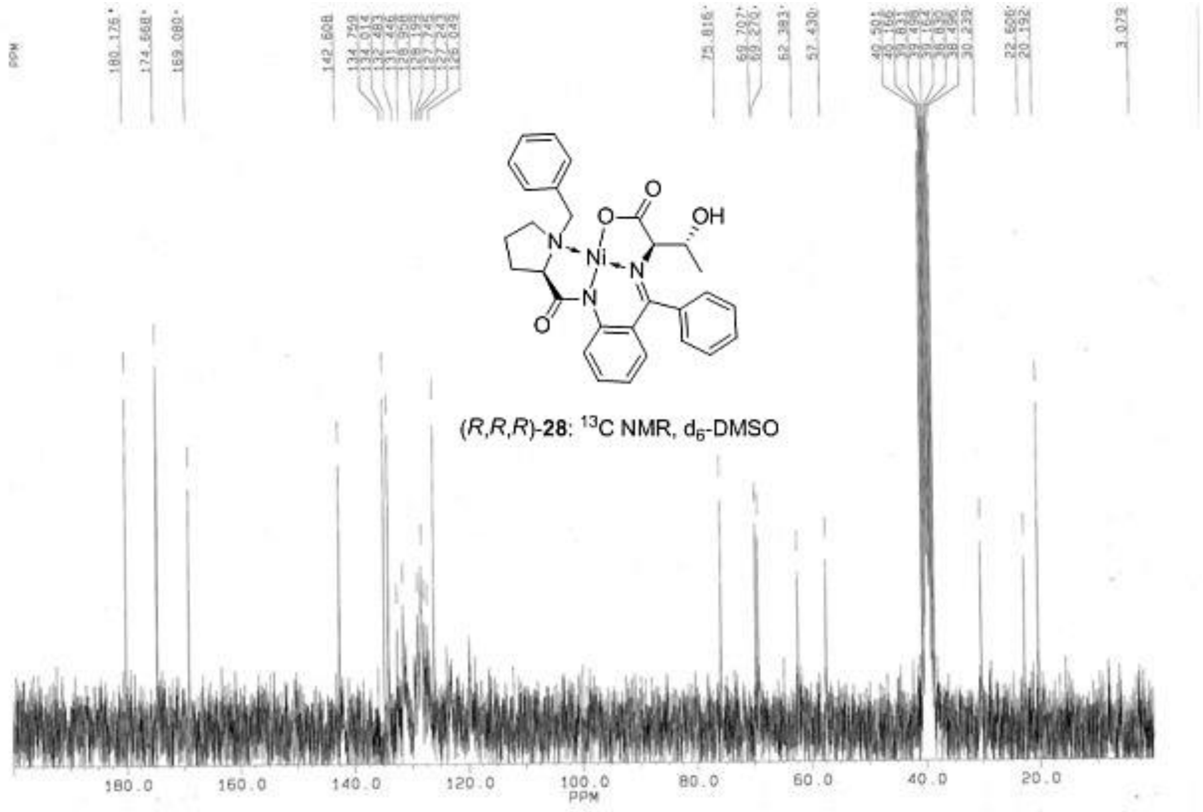
(R,R,R)-28: 13C NMR, d6-DMSO

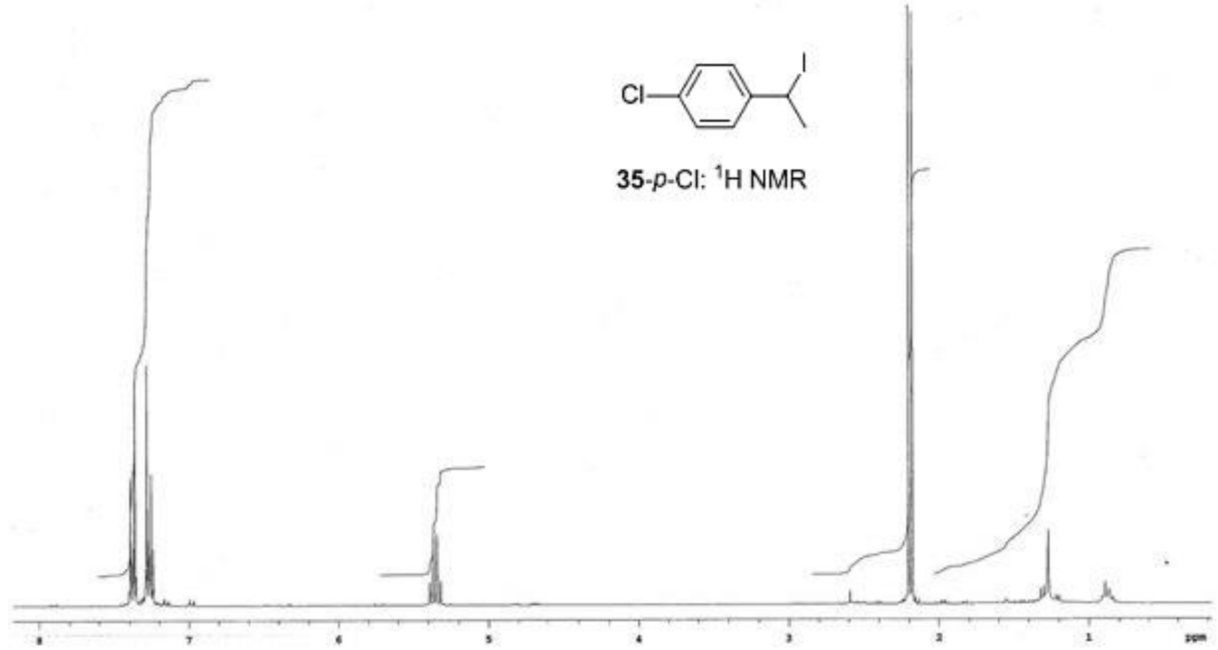
35-p-Cl: 1H NMR

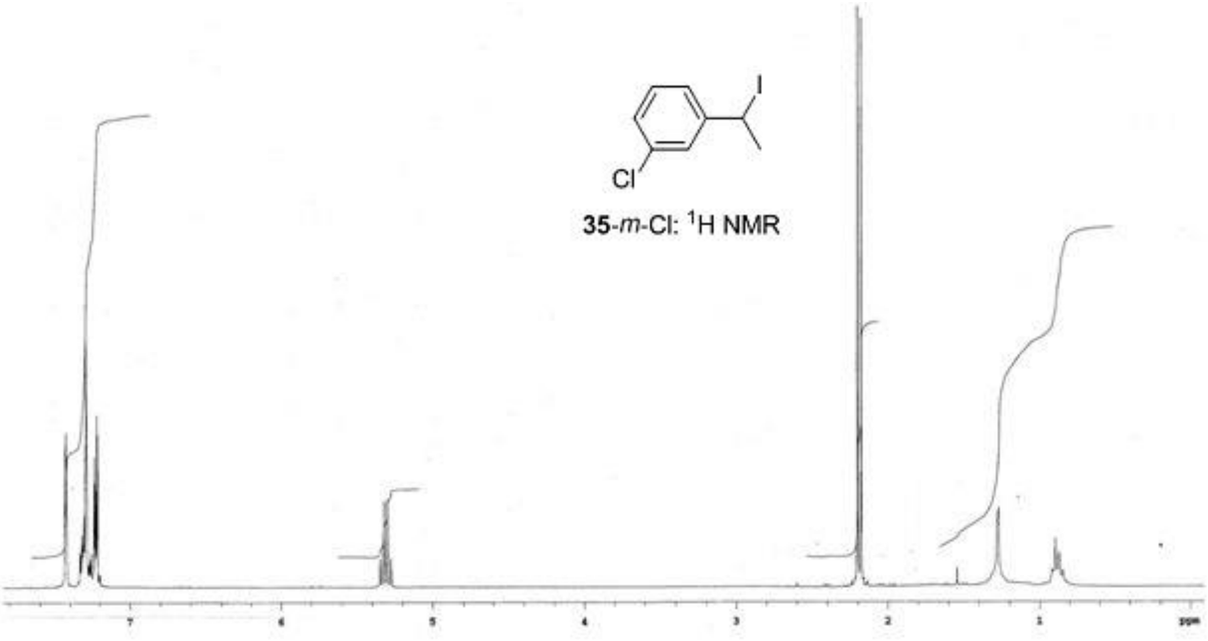

I
Cl
35-m-Cl: 1H NMR
7
6
5
4
3
2
1
ppm

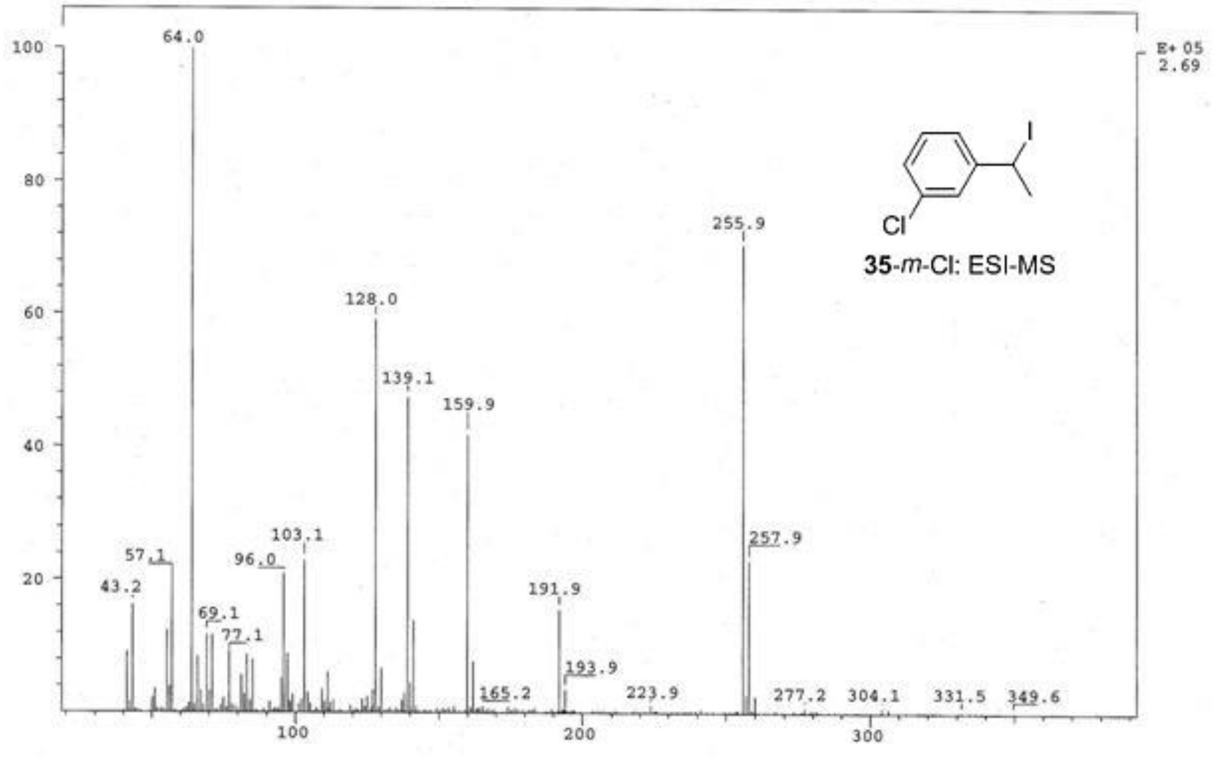

100
80
60
40
20
64.0
E+ 05
2.69
I
Cl
35-m-Cl: ESI-MS
255.9
128.0
139.1
159.9
103.1
57.1
96.0
257.9
43.2
69.1
77.1
191.9
193.9
165.2
223.9
277.2
304.1
331.5
349.6
100
200
300

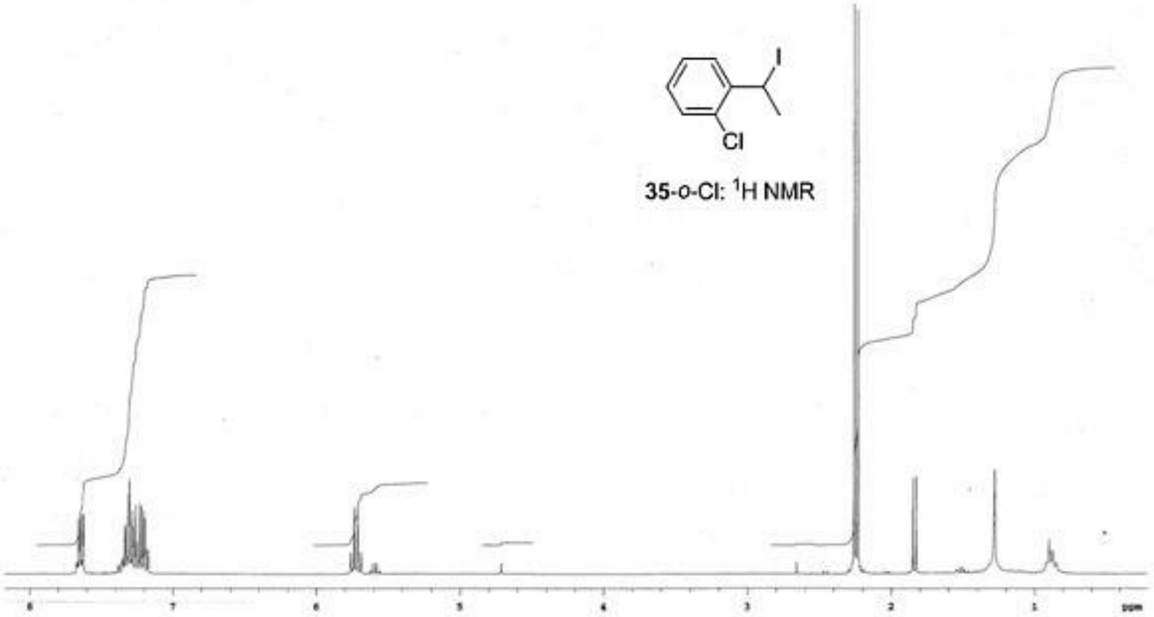

Cl
I
35-o-Cl: 1H NMR
ppm

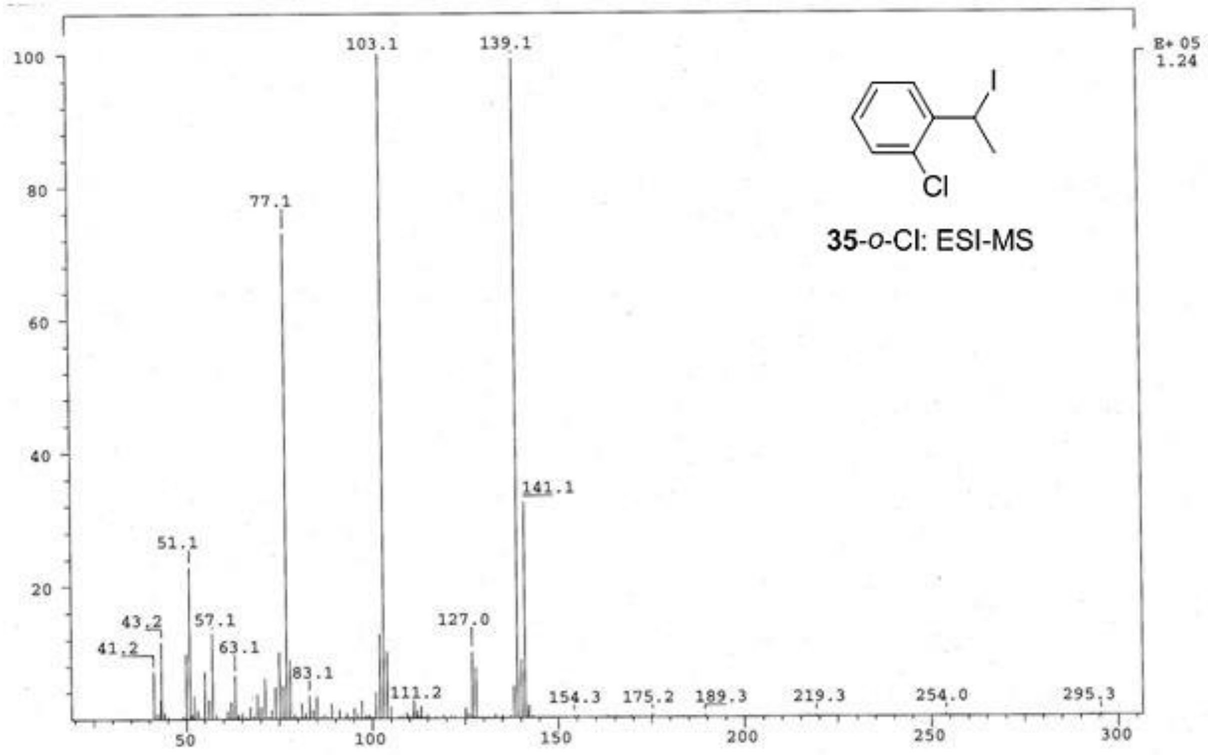

I
Cl
35-o-Cl: ESI-MS
100
80
60
40
20
103.1
139.1
77.1
141.1
51.1
43.2
41.2
57.1
63.1
83.1
111.2
127.0
154.3
175.2
189.3
219.3
254.0
295.3
50
100
150
200
250
300
E+ 05
1.24

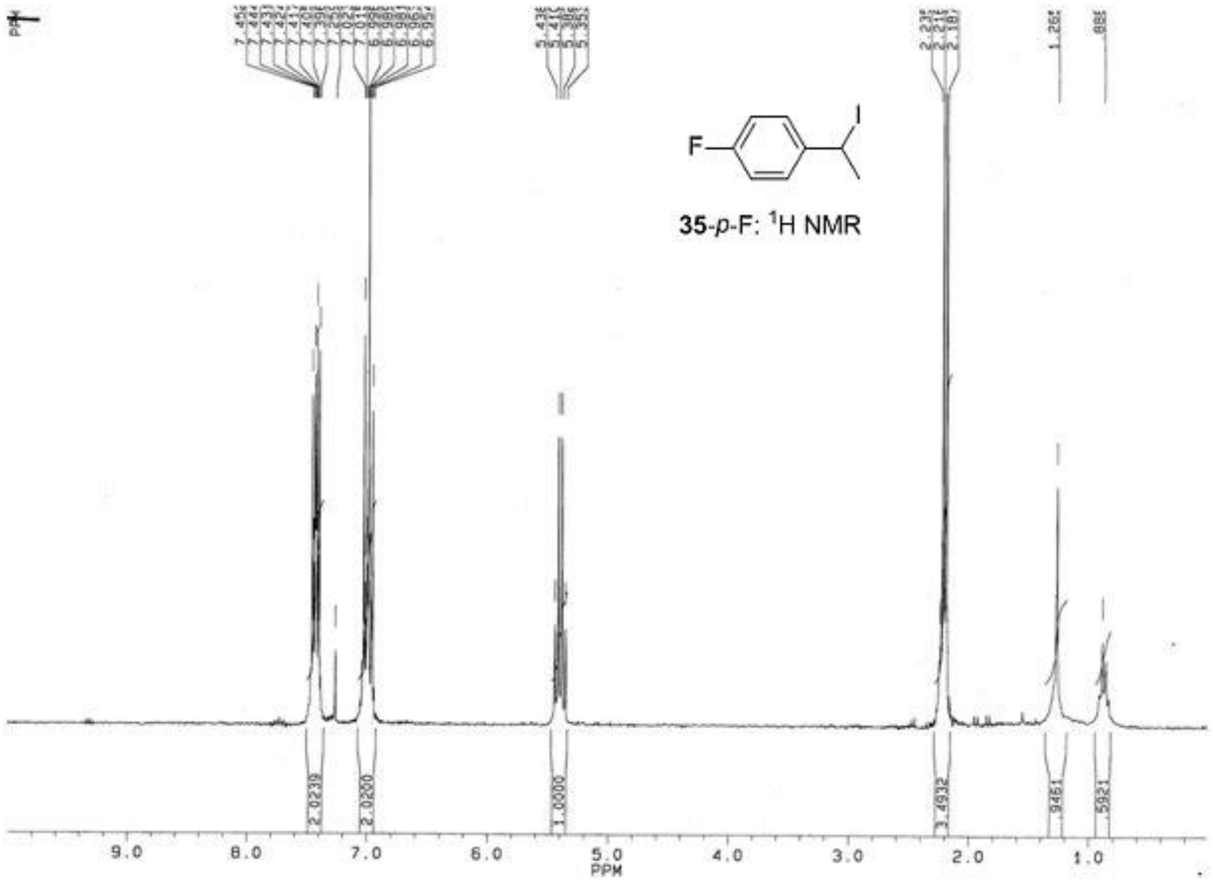
35-p-F: 1H NMR
2.0239
2.0200
1.0000
3.4932
9.0
8.0
7.0
6.0
5.0
PPM
4.0
3.0
2.0
1.0

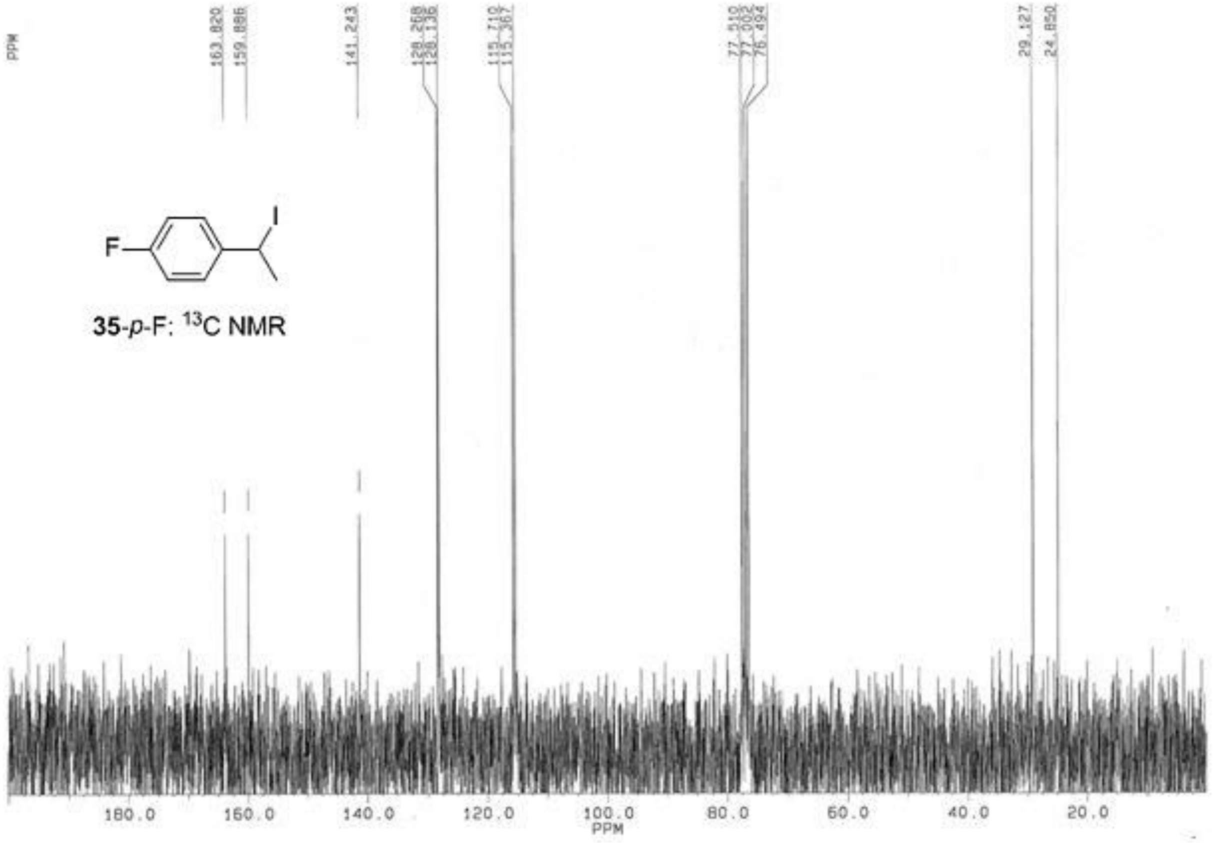
163.820
159.886
141.243
115.710
115.367
29.127
24.850
35-p-F: 13C NMR
180.0
160.0
140.0
120.0
100.0
PPM
80.0
60.0
40.0
20.0

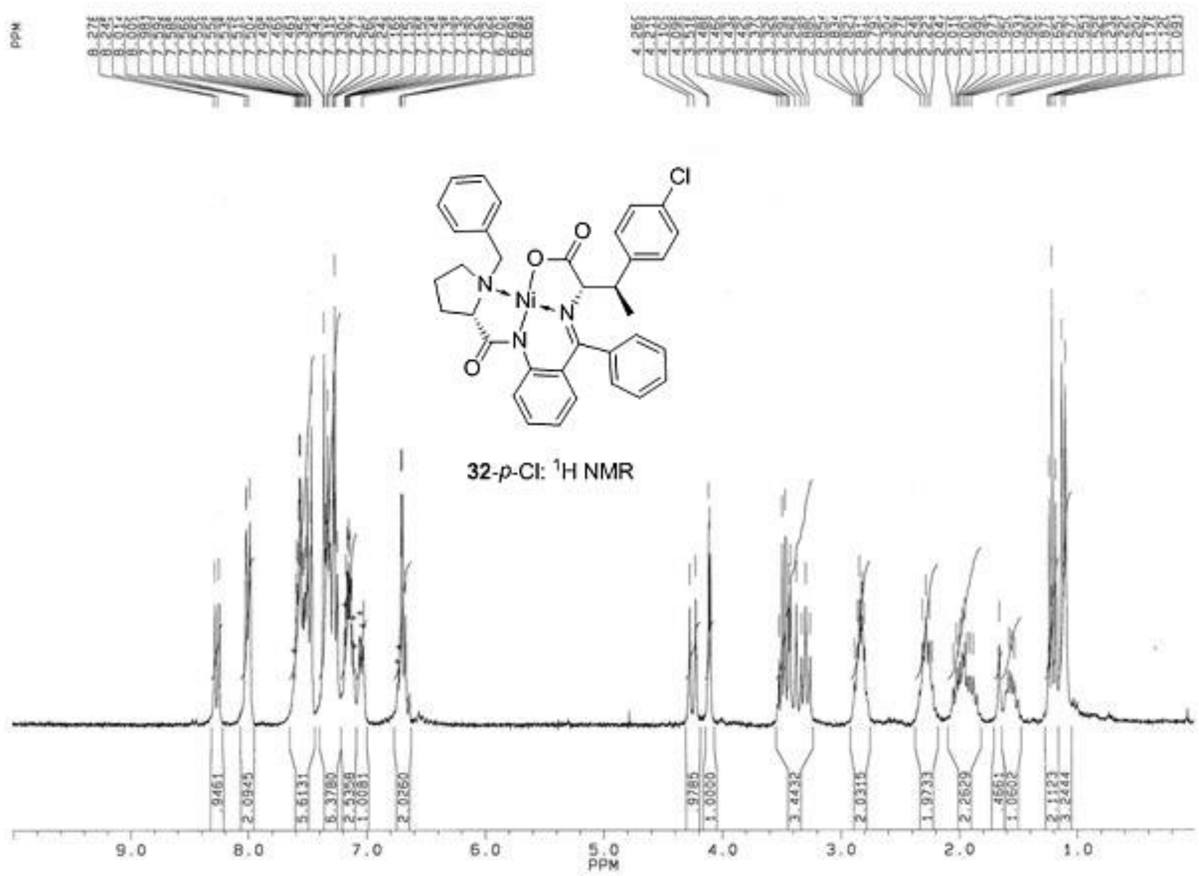
32-p-Cl: 1H NMR

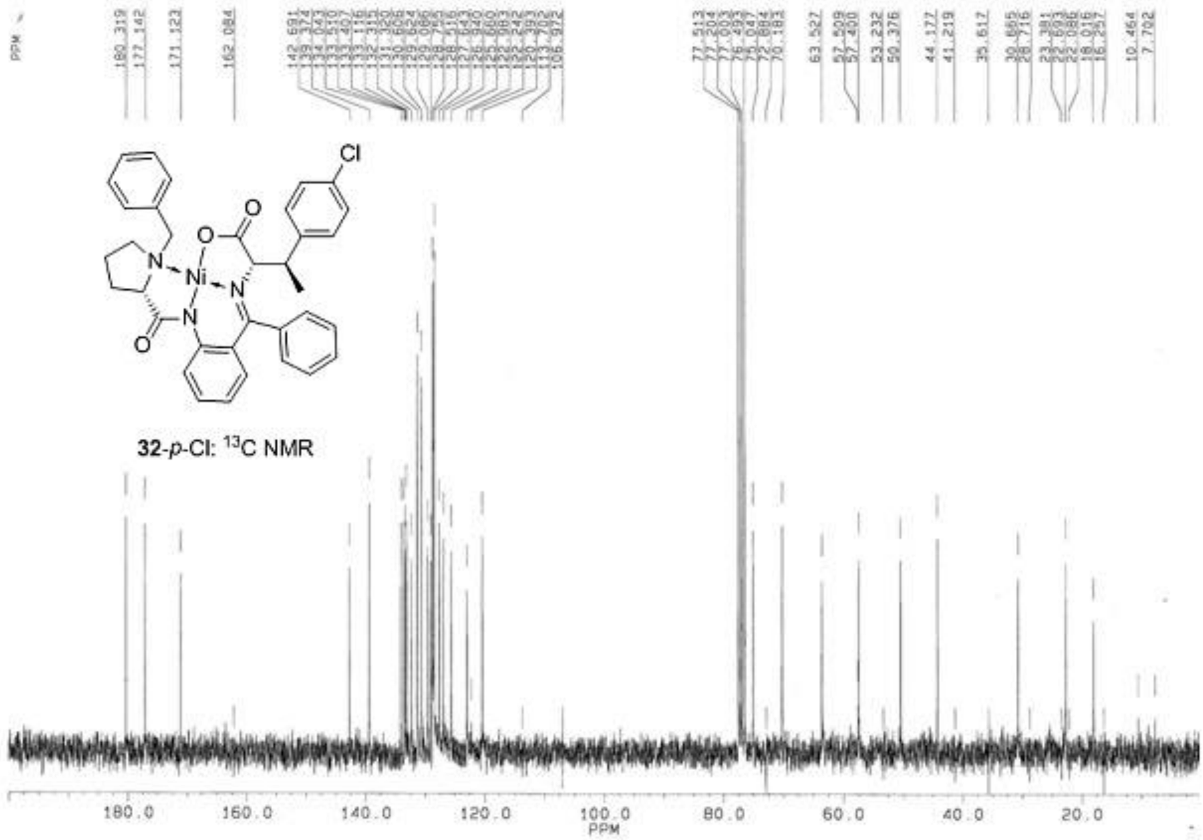
32-p-Cl: 13C NMR

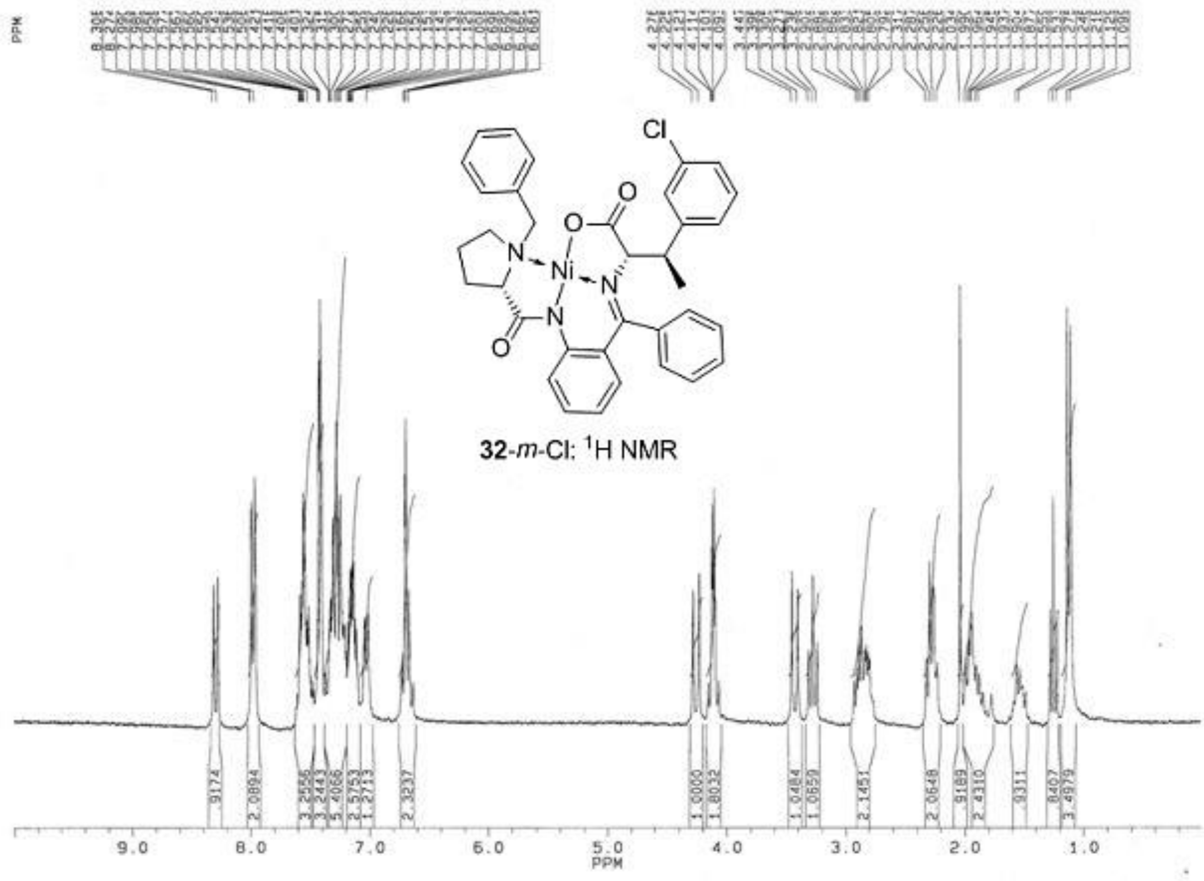
32-m-Cl: ¹H NMR
9.0 8.0 7.0 6.0 5.0 4.0 3.0 2.0 1.0
PPM

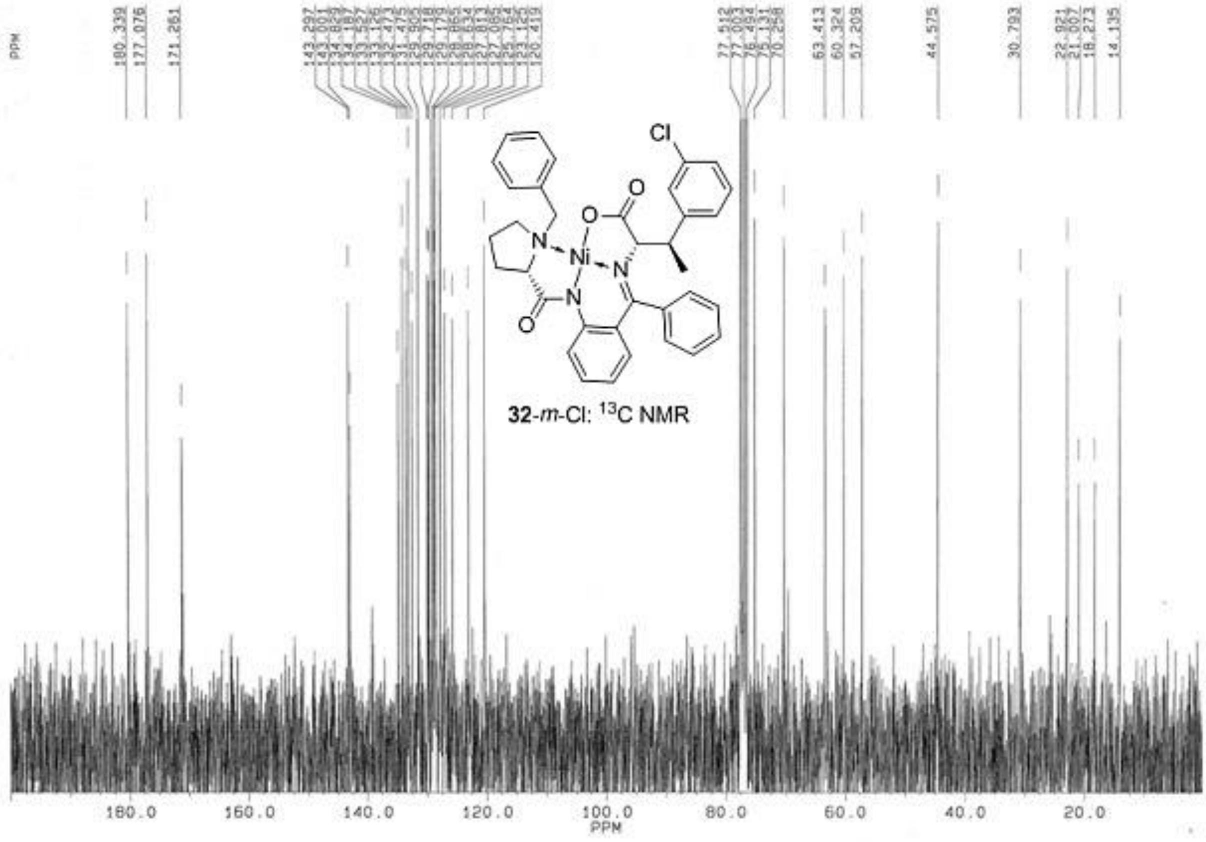
32-m-Cl: ¹³C NMR
180.0 160.0 140.0 120.0 100.0 80.0 60.0 40.0 20.0
PPM

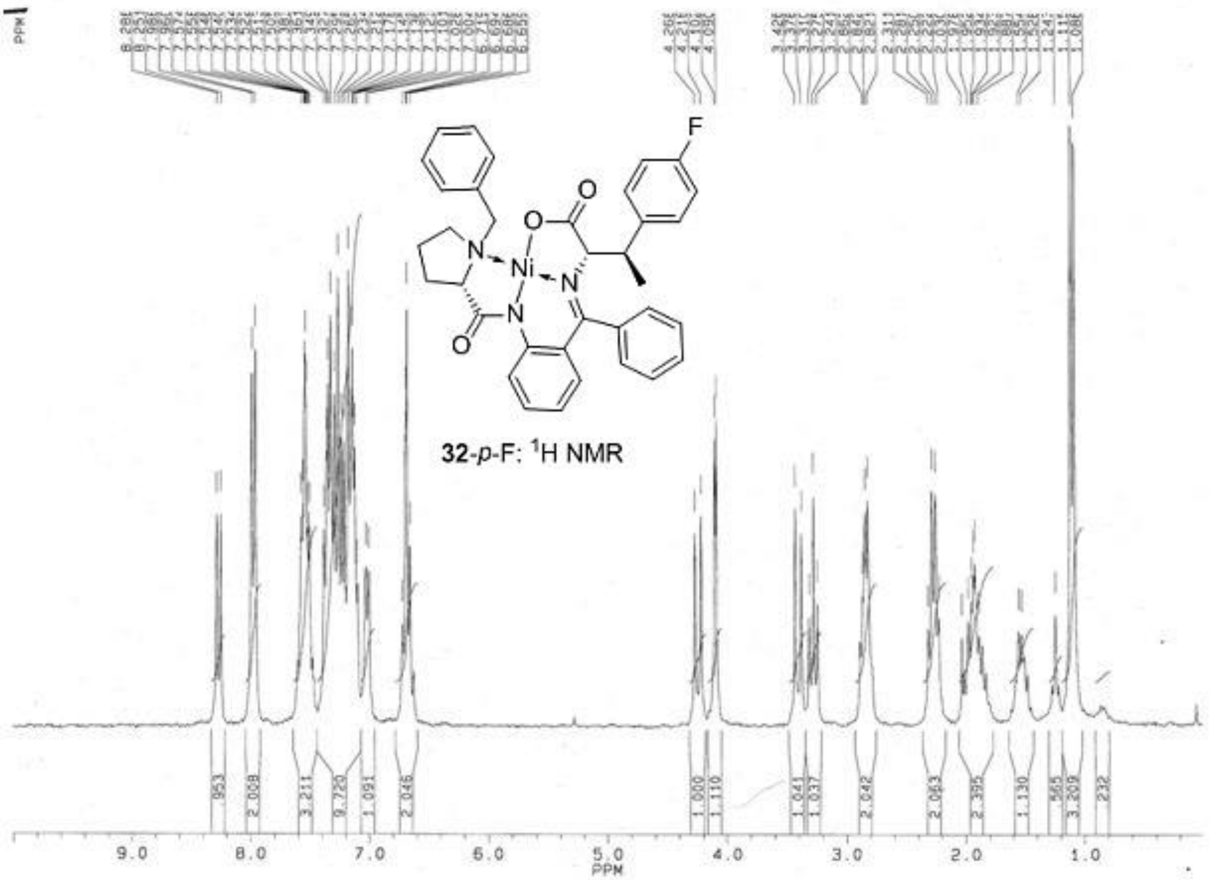
32-p-F: ^{1}H NMR

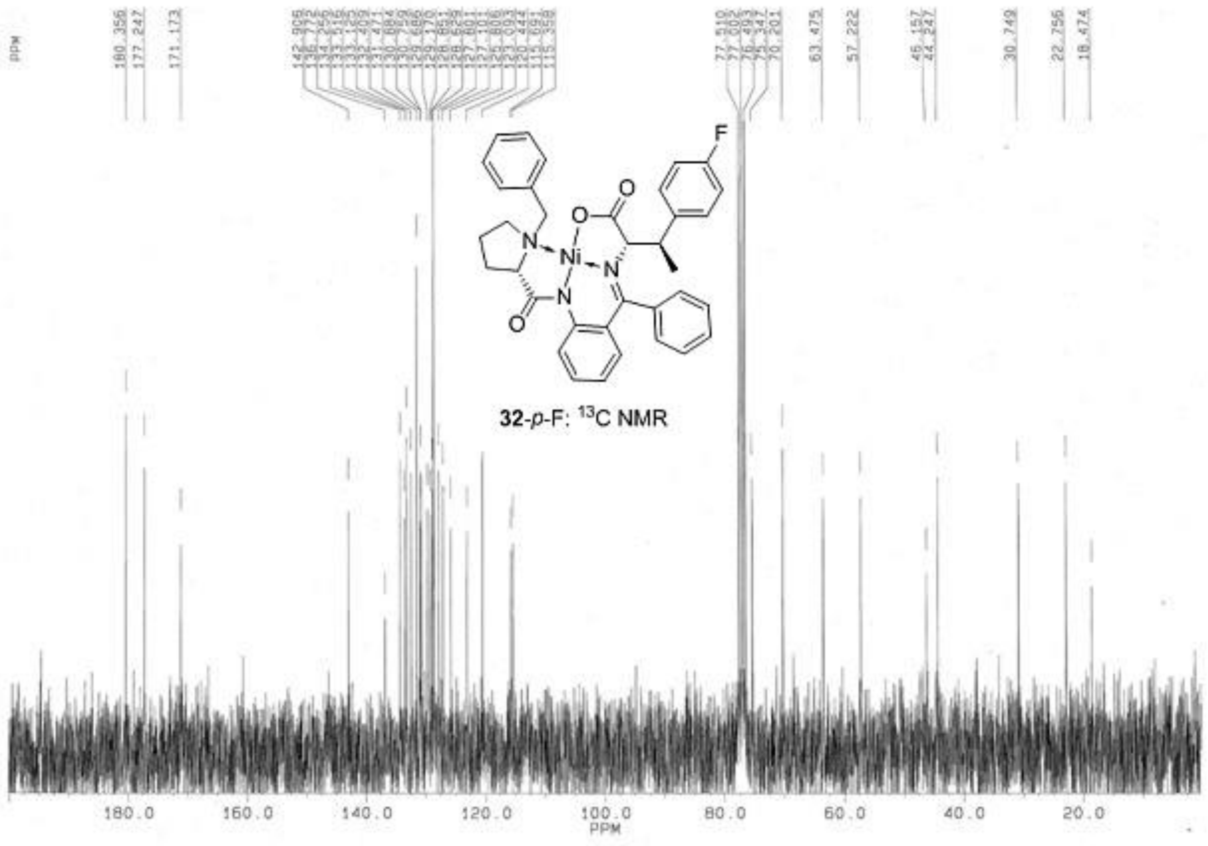
32-p-F: ^{13}C NMR

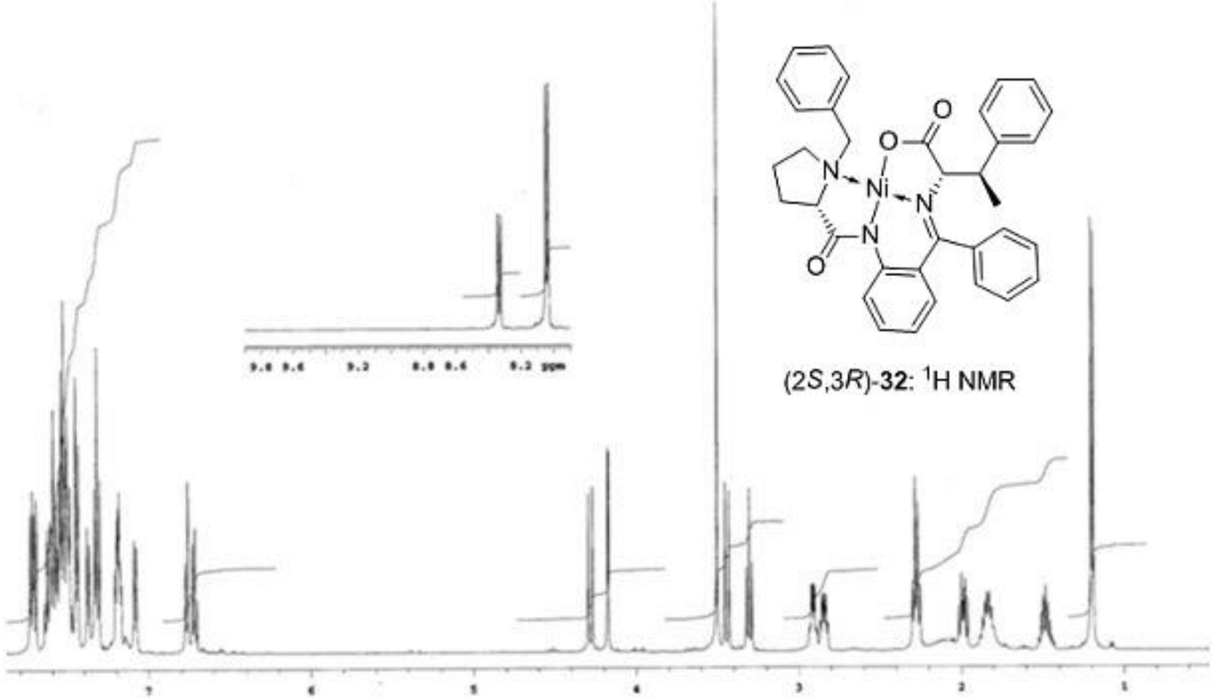

(2S,3R)-32: 1H NMR
7
6
5
4
3
2
1

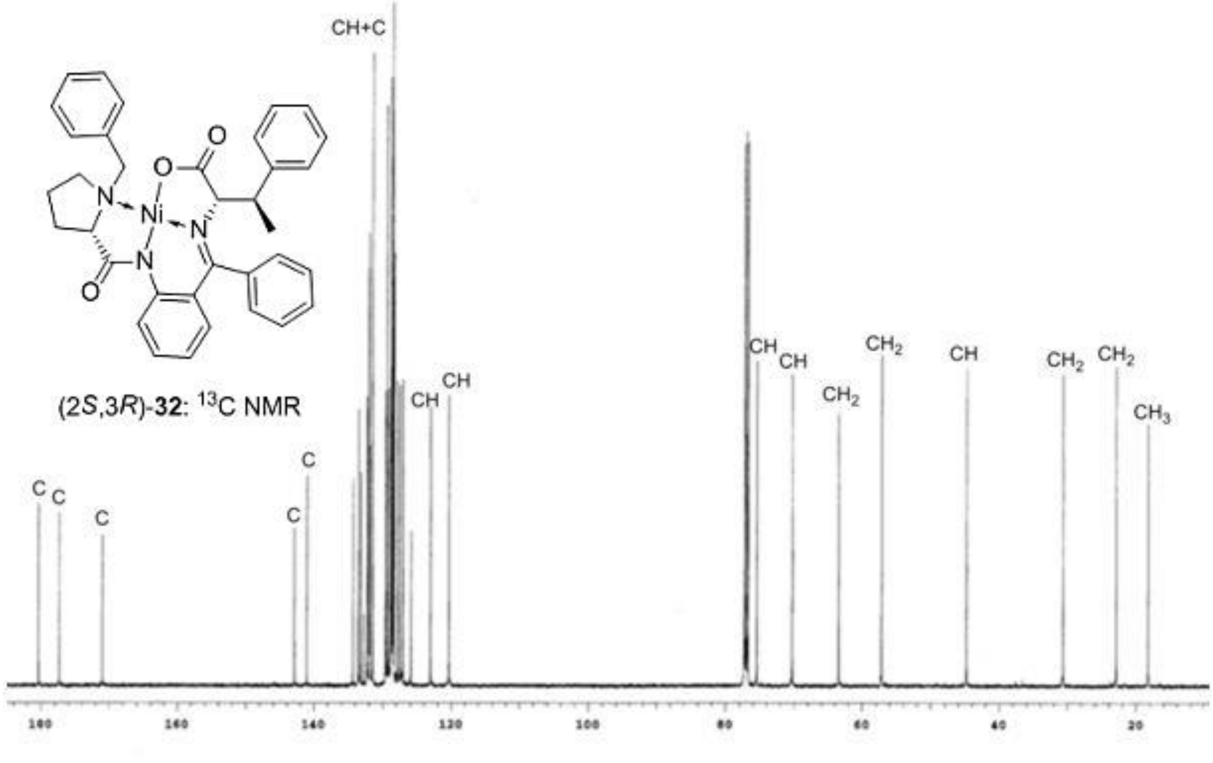

(2S,3R)-32: 13C NMR
CH+C
C C
C
C
C
CH CH
CH CH
CH2
CH2
CH
CH2 CH2
CH3
180
160
140
120
100
80
60
40
20

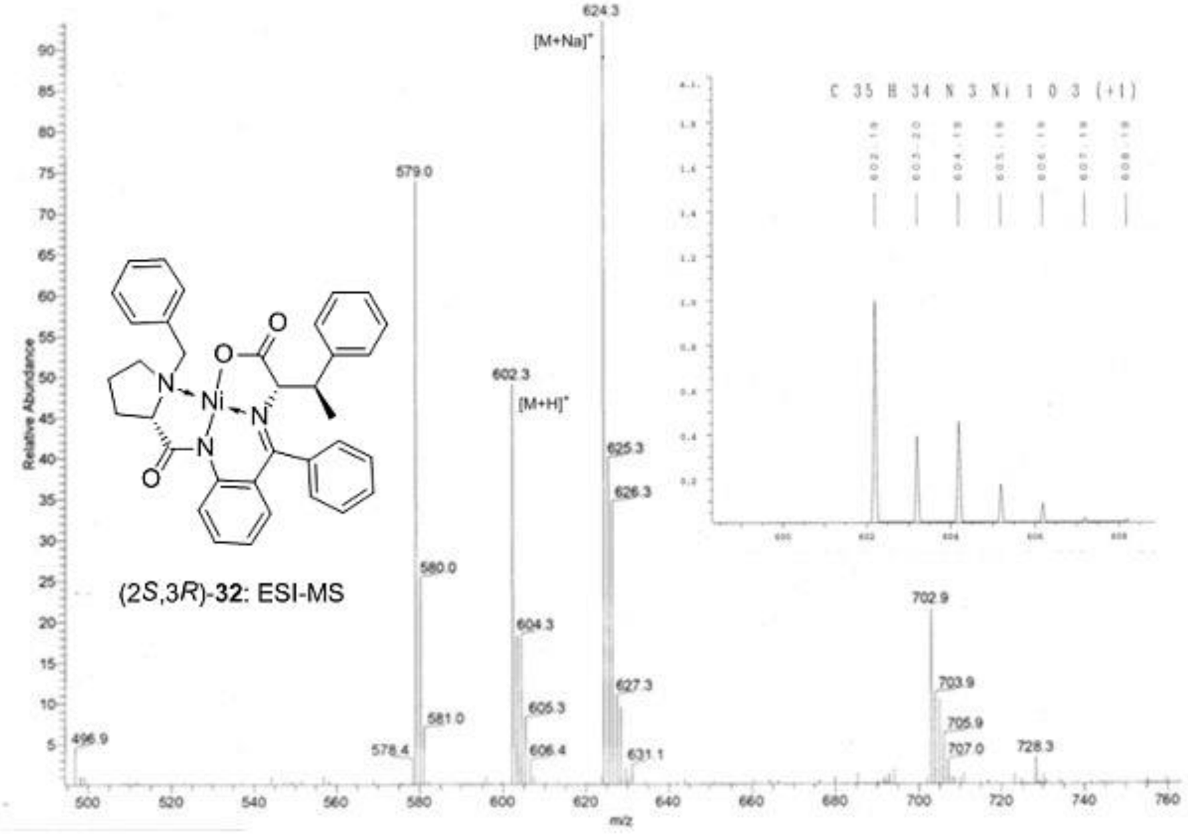
(2S,3R)-32: ESI-MS
Relative Abundance
m/z
624.3
[M+Na]+
579.0
580.0
581.0
578.4
602.3
[M+H]+
604.3
605.3
606.4
625.3
626.3
627.3
631.1
496.9
702.9
703.9
705.9
707.0
728.3
C 35 H 34 N 3 Ni 1 O 3 (+1)
602.19
603.20
604.19
605.19
606.19
607.19
608.19
O
N
Ni
N
N
O

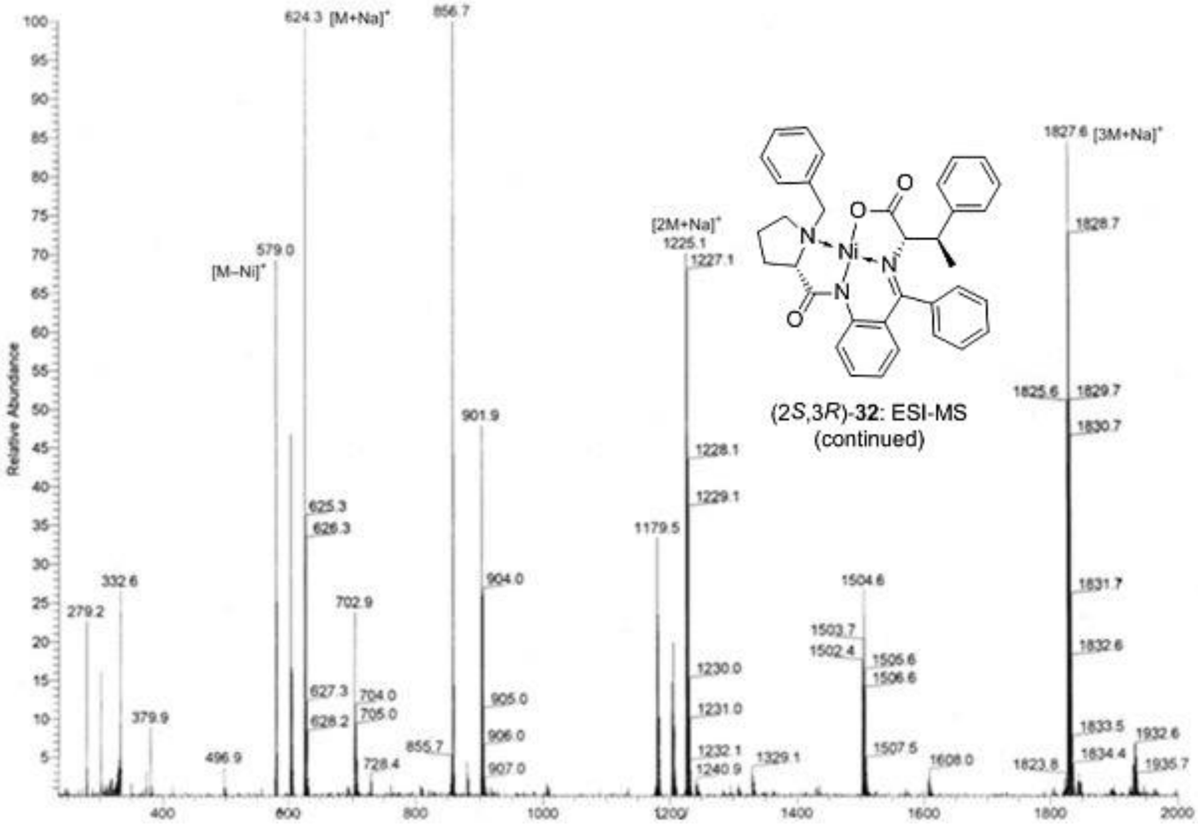
(2S,3R)-32: ESI-MS
(continued)
Relative Abundance
624.3 [M+Na]+
856.7
579.0
[M–Ni]+
901.9
904.0
905.0
906.0
907.0
625.3
626.3
627.3
628.2
702.9
704.0
705.0
728.4
855.7
332.6
279.2
379.9
496.9
[2M+Na]+
1225.1
1227.1
1228.1
1229.1
1230.0
1231.0
1232.1
1240.9
1179.5
1329.1
1504.6
1503.7
1502.4
1505.6
1506.6
1507.5
1608.0
1827.6 [3M+Na]+
1828.7
1825.6
1829.7
1830.7
1831.7
1832.6
1833.5
1834.4
1823.8
1932.6
1935.7

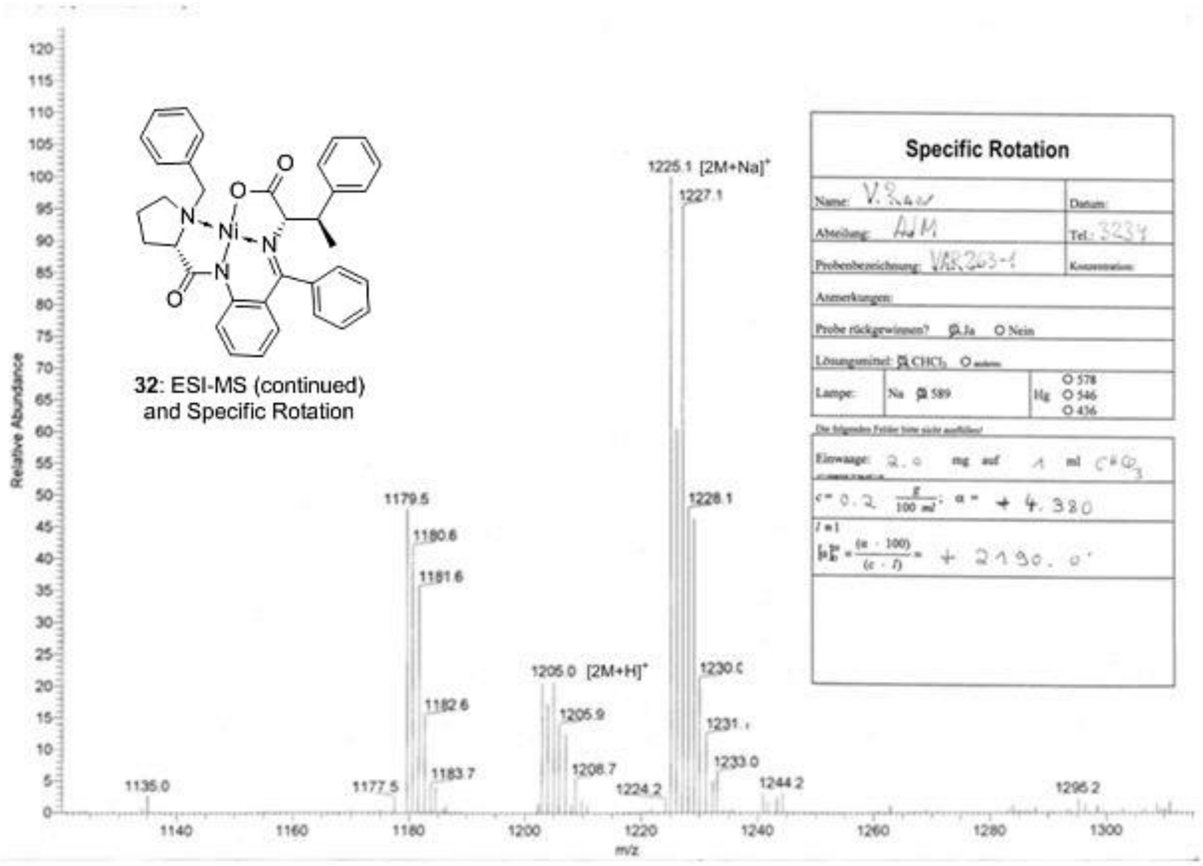

32: ESI-MS (continued) and Specific Rotation

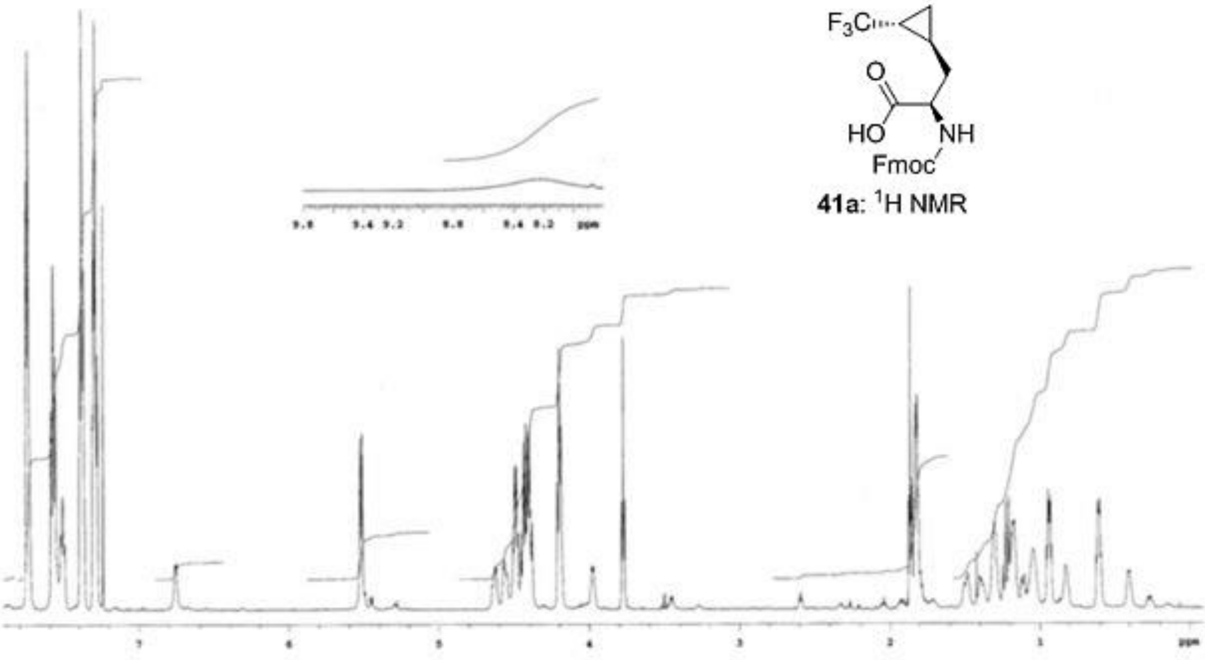

41a: ^{1}H NMR

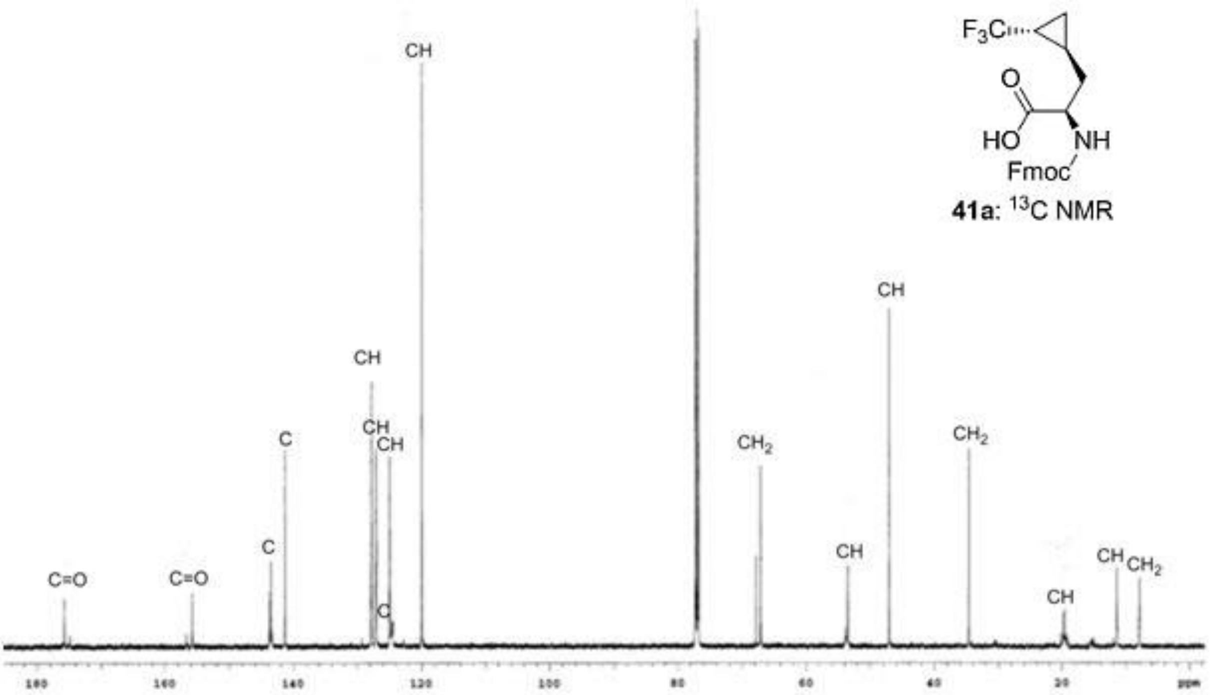
F3C
O
HO
NH
Fmoc
41a: 13C NMR
CH
CH
CH
CH
C
C
C
C=O
C=O
CH2
CH
CH
CH2
CH
CH
CH2
ppm

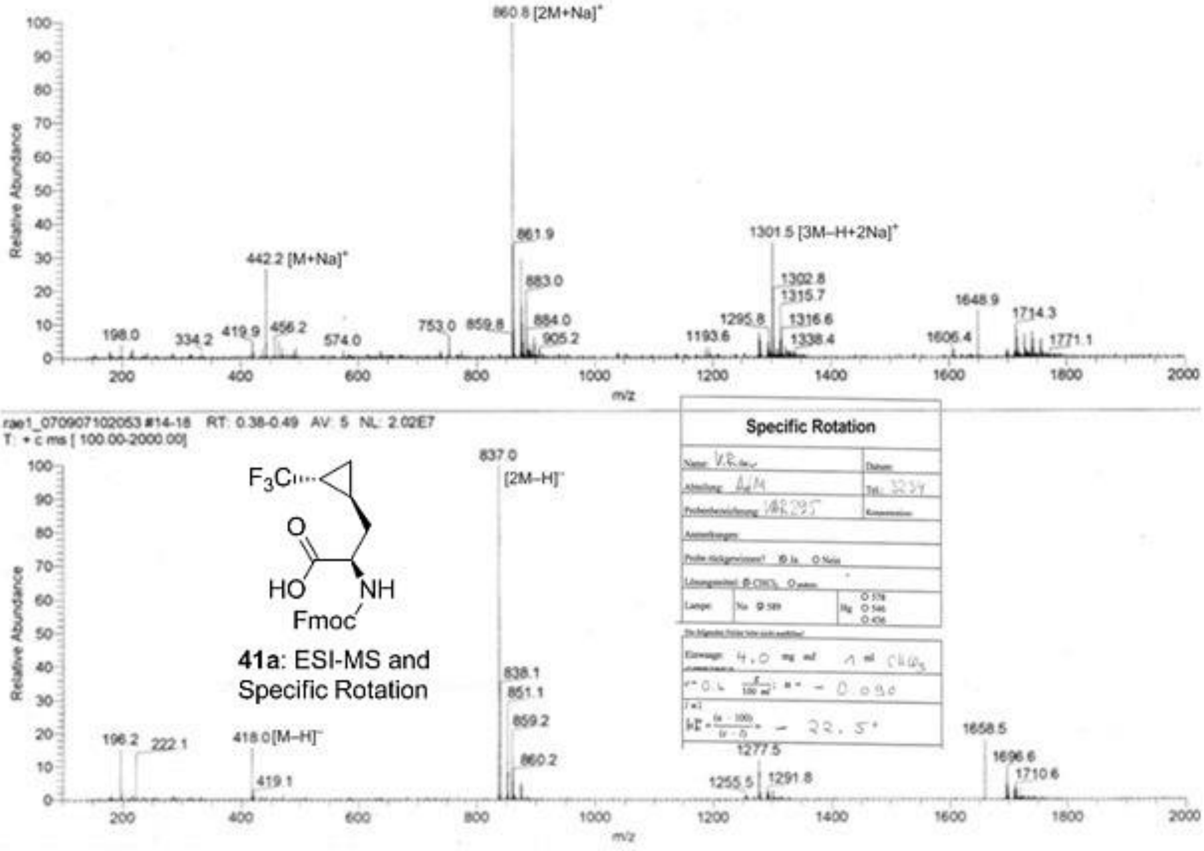
860.8 [2M+Na]+
442.2 [M+Na]+
1301.5 [3M−H+2Na]+
861.9
883.0
884.0
905.2
198.0
334.2
419.9
456.2
574.0
753.0
859.8
1193.6
1295.8
1302.8
1315.7
1316.6
1338.4
1606.4
1648.9
1714.3
1771.1
Relative Abundance
m/z
rae1_070907102053 #14-18 RT: 0.38-0.49 AV: 5 NL: 2.02E7
T: + c ms [100.00-2000.00]
F3C
O
HO
NH
Fmoc
41a: ESI-MS and Specific Rotation
837.0
[2M−H]−
418.0 [M−H]−
196.2
222.1
419.1
838.1
851.1
859.2
860.2
1255.5
1277.5
1291.8
1658.5
1696.6
1710.6
Specific Rotation

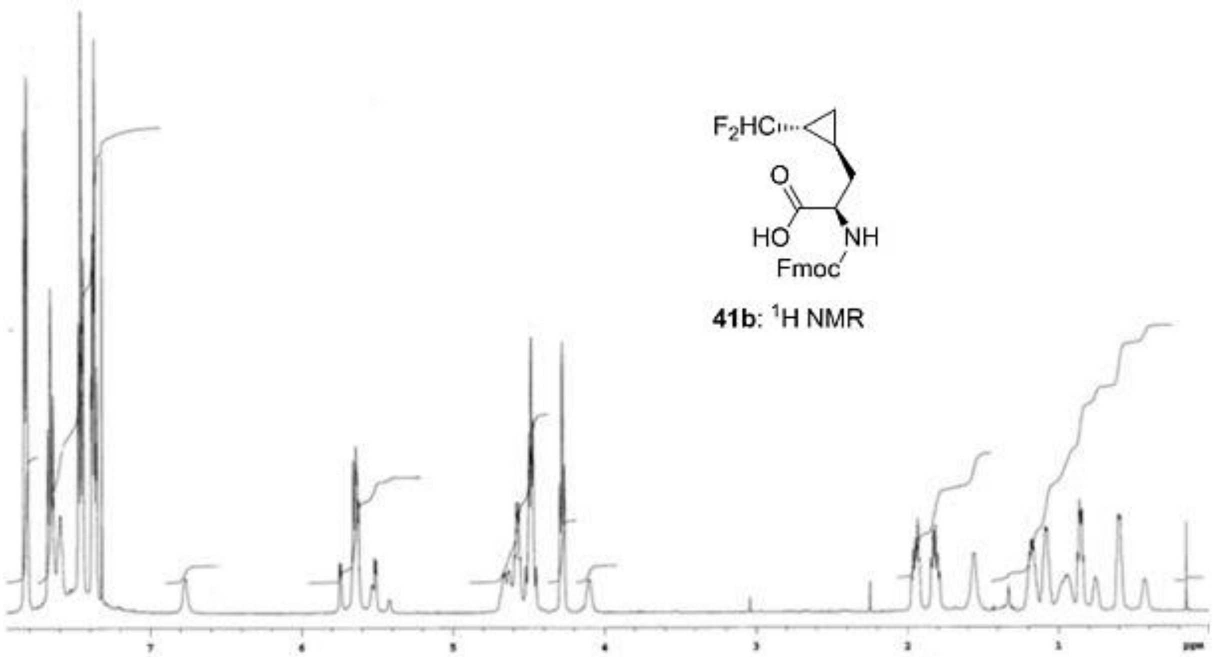
F2HC
O
HO
NH
Fmoc
41b: 1H NMR
7
6
5
4
3
2
1
ppm

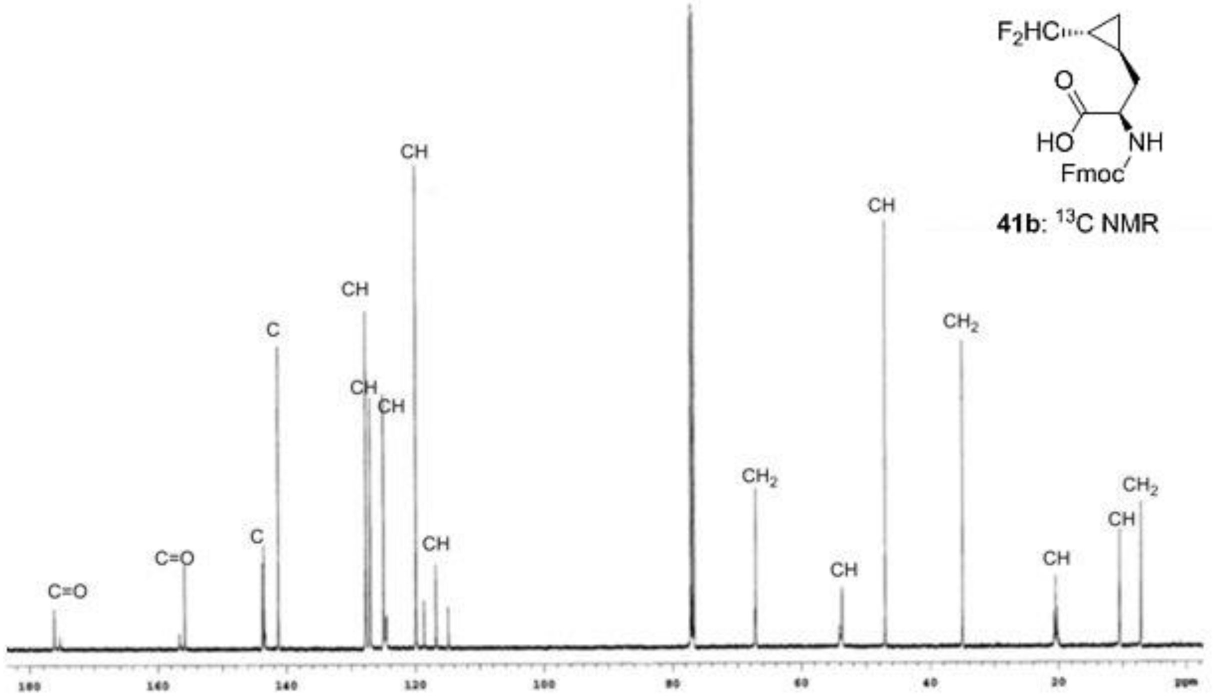
F2HC
O
HO
NH
Fmoc
41b: 13C NMR
C=O
C=O
C
C
CH
CH
CH
CH
CH
CH2
CH
CH
CH2
CH
CH
CH2
160
140
120
100
80
60
40
20
ppm

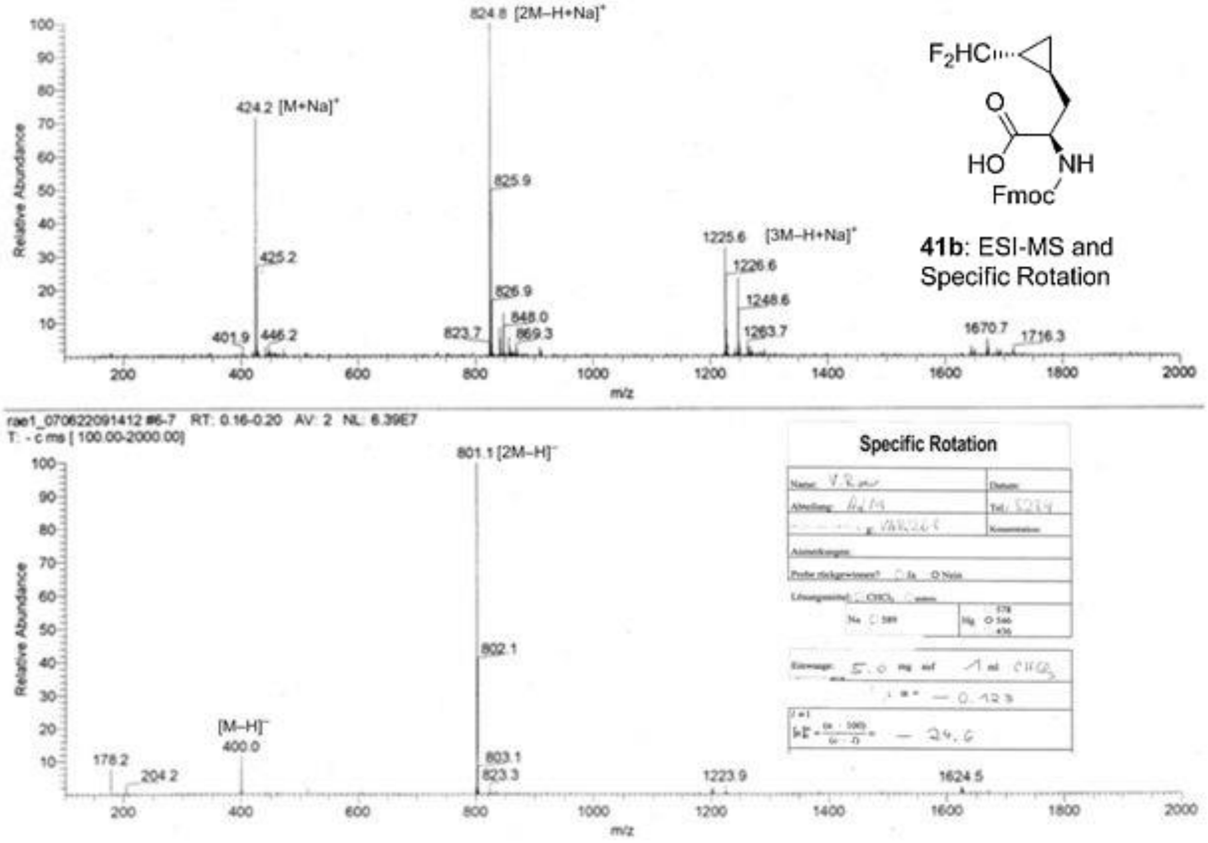
824.8 [2M–H+Na]+
424.2 [M+Na]+
1225.6 [3M–H+Na]+
41b: ESI-MS and Specific Rotation
801.1 [2M–H]−
[M–H]−
400.0
Specific Rotation
Relative Abundance
m/z

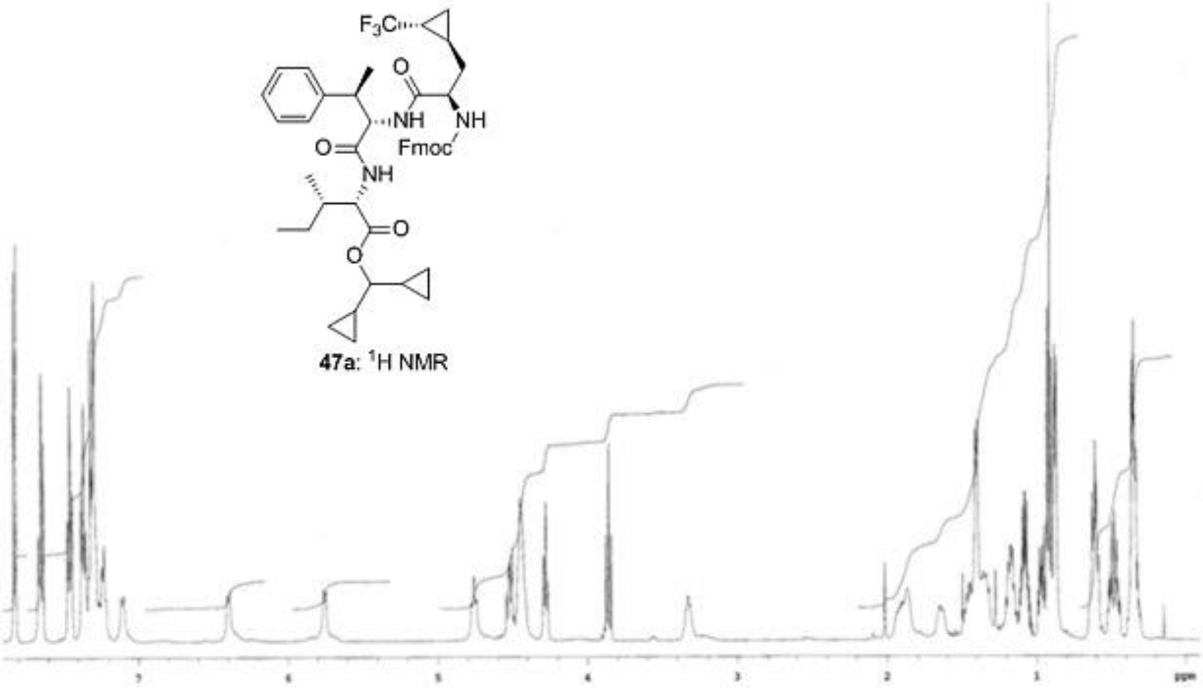
47a: 1H NMR
ppm

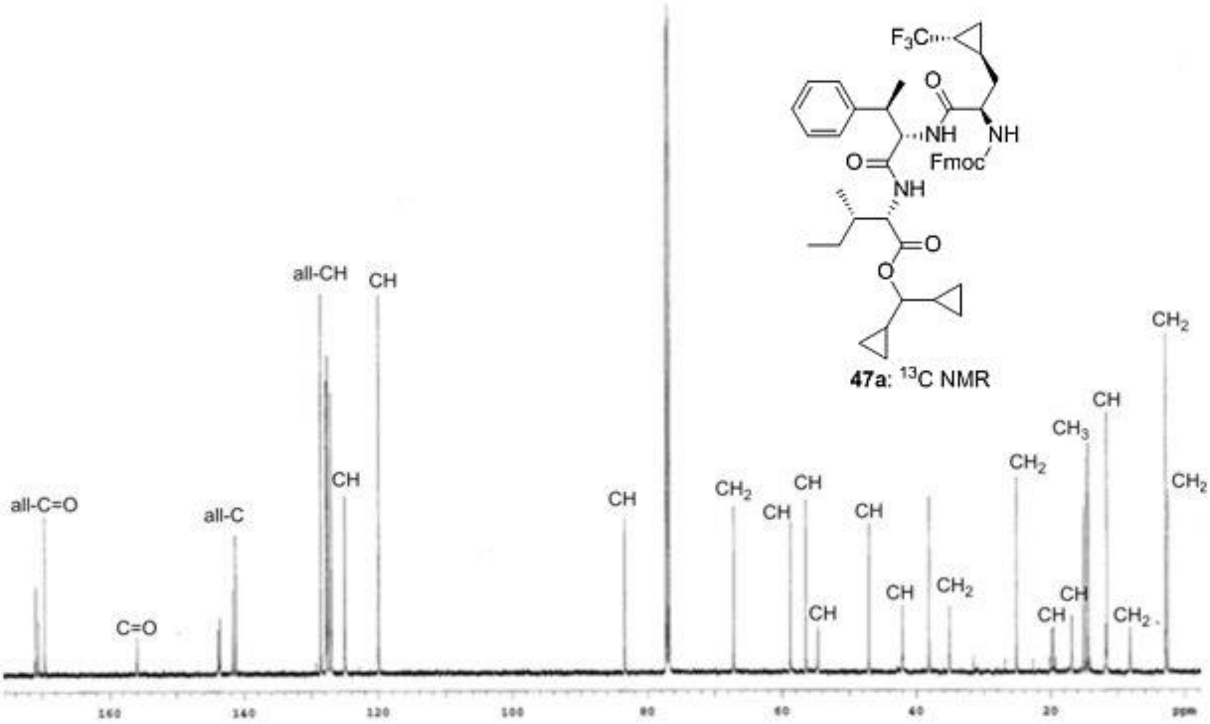
F3C
O
NH
NH
Fmoc
O
NH
O
O
47a: 13C NMR
all-C=O
C=O
all-C
all-CH
CH
CH
CH
CH2
CH
CH
CH
CH
CH
CH2
CH2
CH3
CH
CH
CH
CH2
CH2
CH2
160
140
120
100
80
60
40
20
ppm

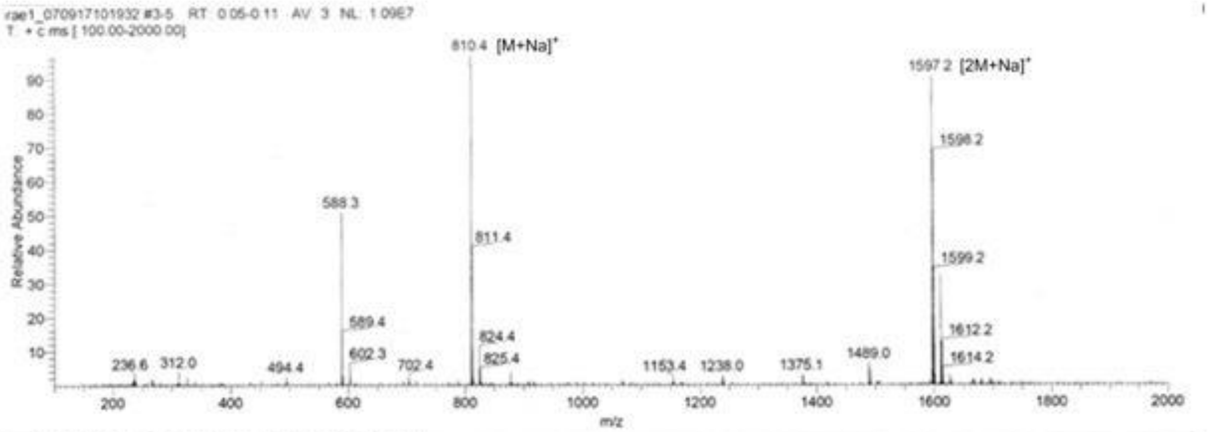
rae1_070917101932 #3-5 RT: 0.05-0.11 AV: 3 NL: 1.09E7
T: + c ms [100.00-2000.00]
810.4 [M+Na]+
1597.2 [2M+Na]+
1598.2
588.3
811.4
1599.2
589.4
824.4
1612.2
602.3
1614.2
236.6
312.0
494.4
702.4
825.4
1153.4
1238.0
1375.1
1489.0
Relative Abundance
m/z

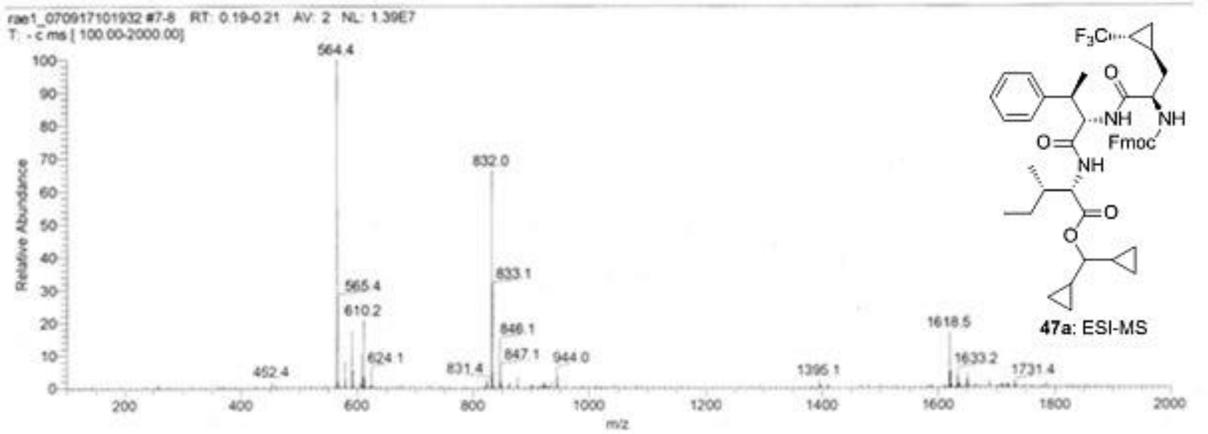
rae1_070917101932 #7-8 RT: 0.19-0.21 AV: 2 NL: 1.39E7
T: - c ms [100.00-2000.00]
564.4
832.0
833.1
565.4
610.2
846.1
624.1
847.1
944.0
831.4
452.4
1395.1
1618.5
1633.2
1731.4
F3C
O
NH
NH
Fmoc
O
NH
O
O
47a: ESI-MS
Relative Abundance
m/z

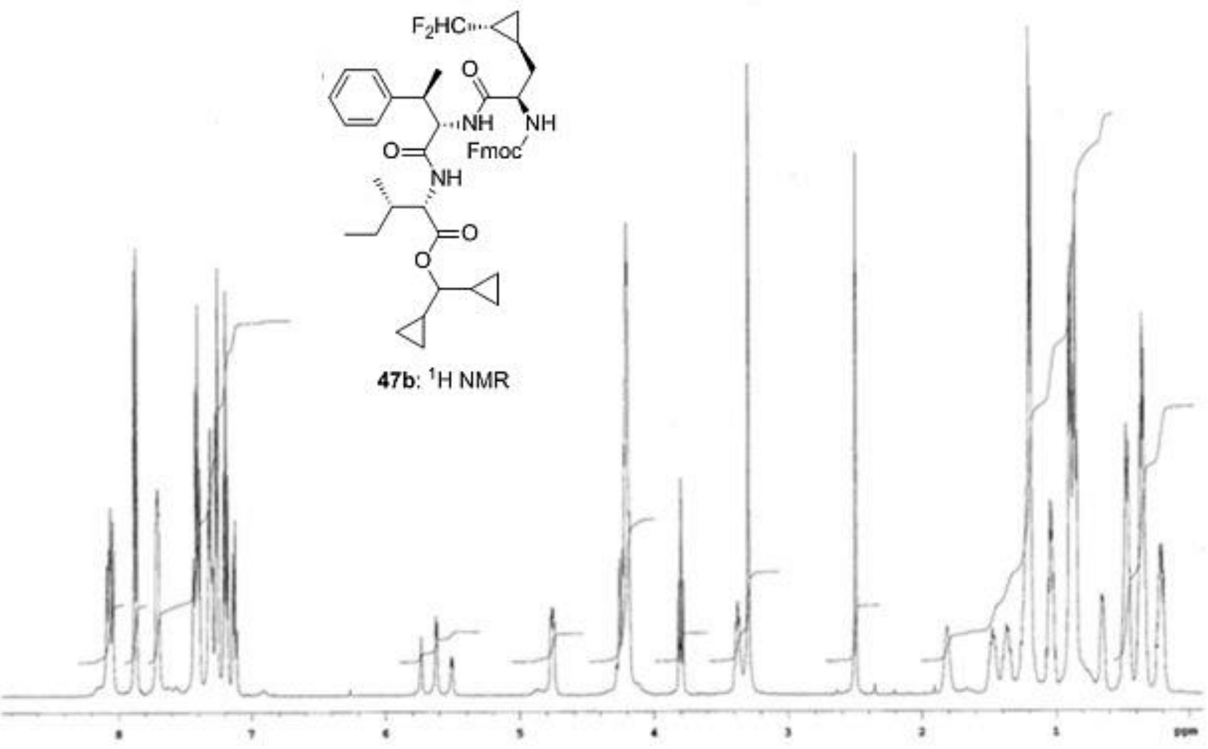

47b: ¹H NMR

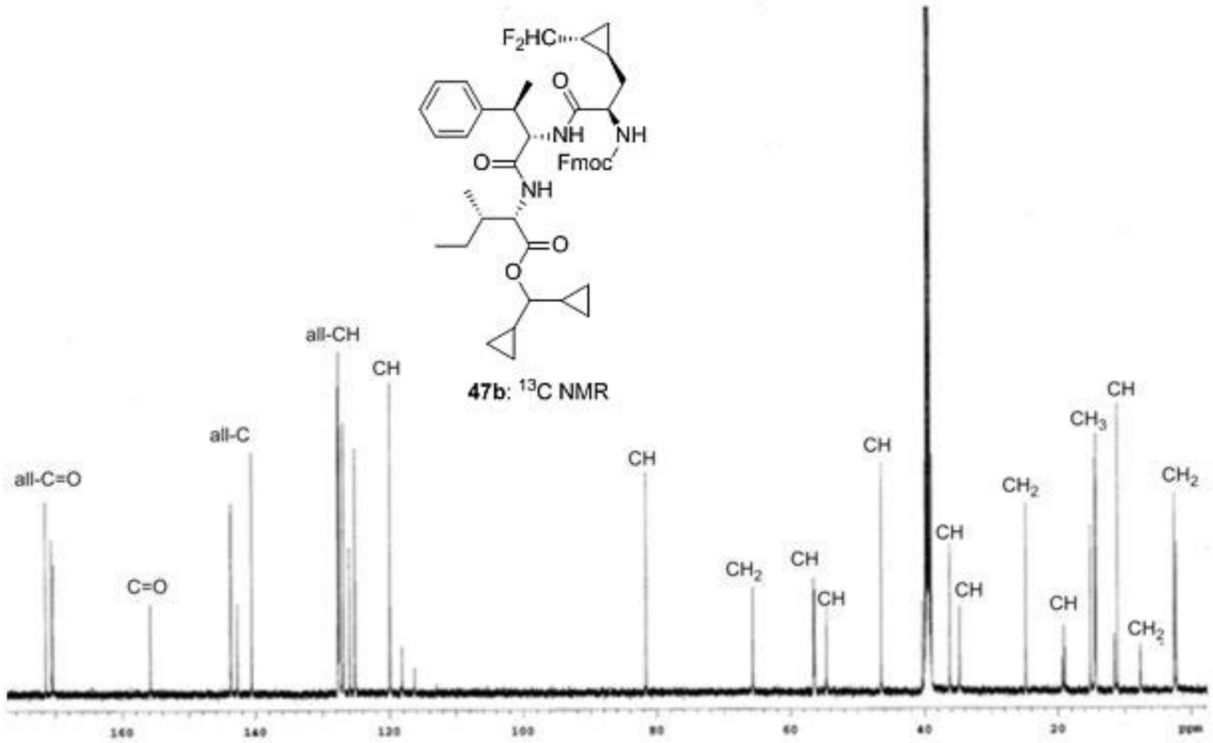

47b: ¹³C NMR

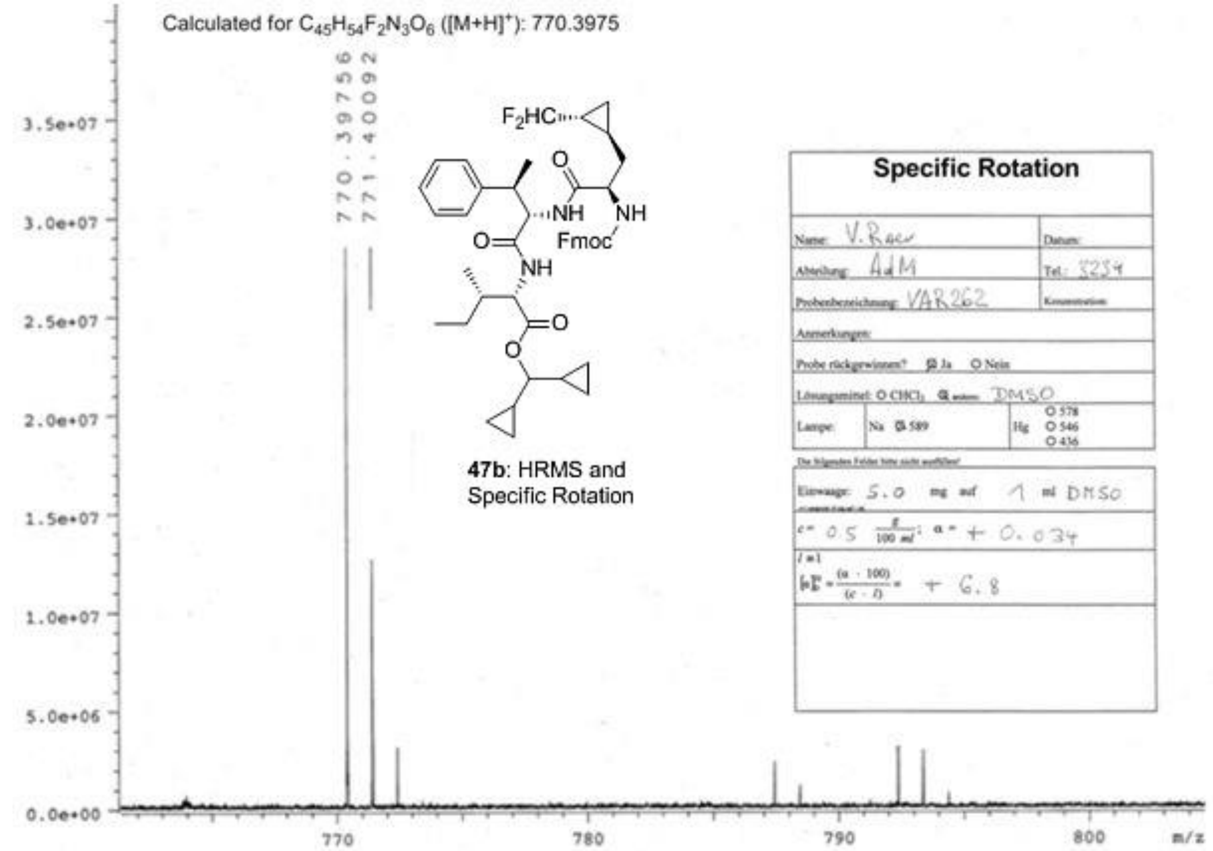

47b: HRMS and Specific Rotation

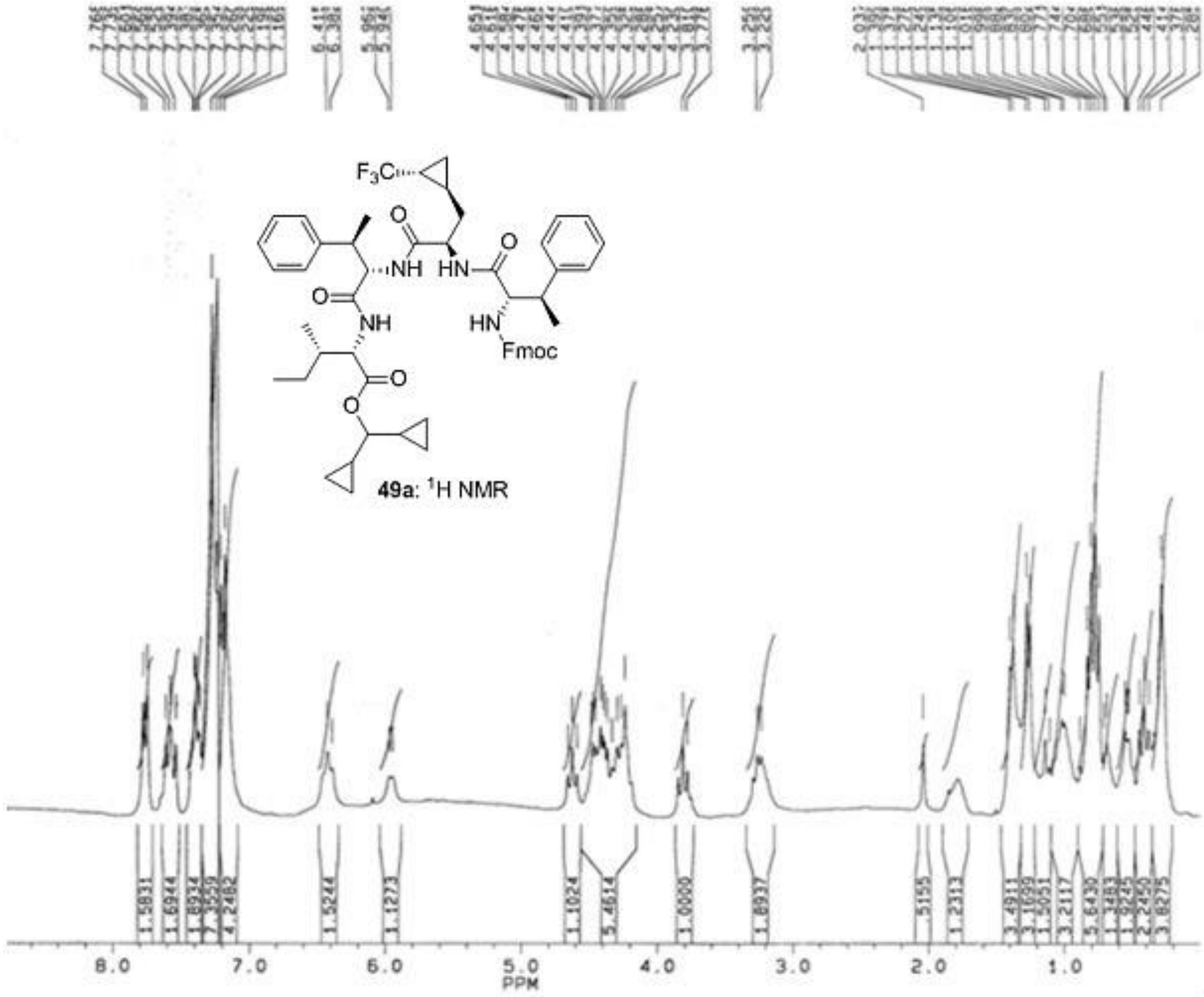

49a: ^{1}H NMR

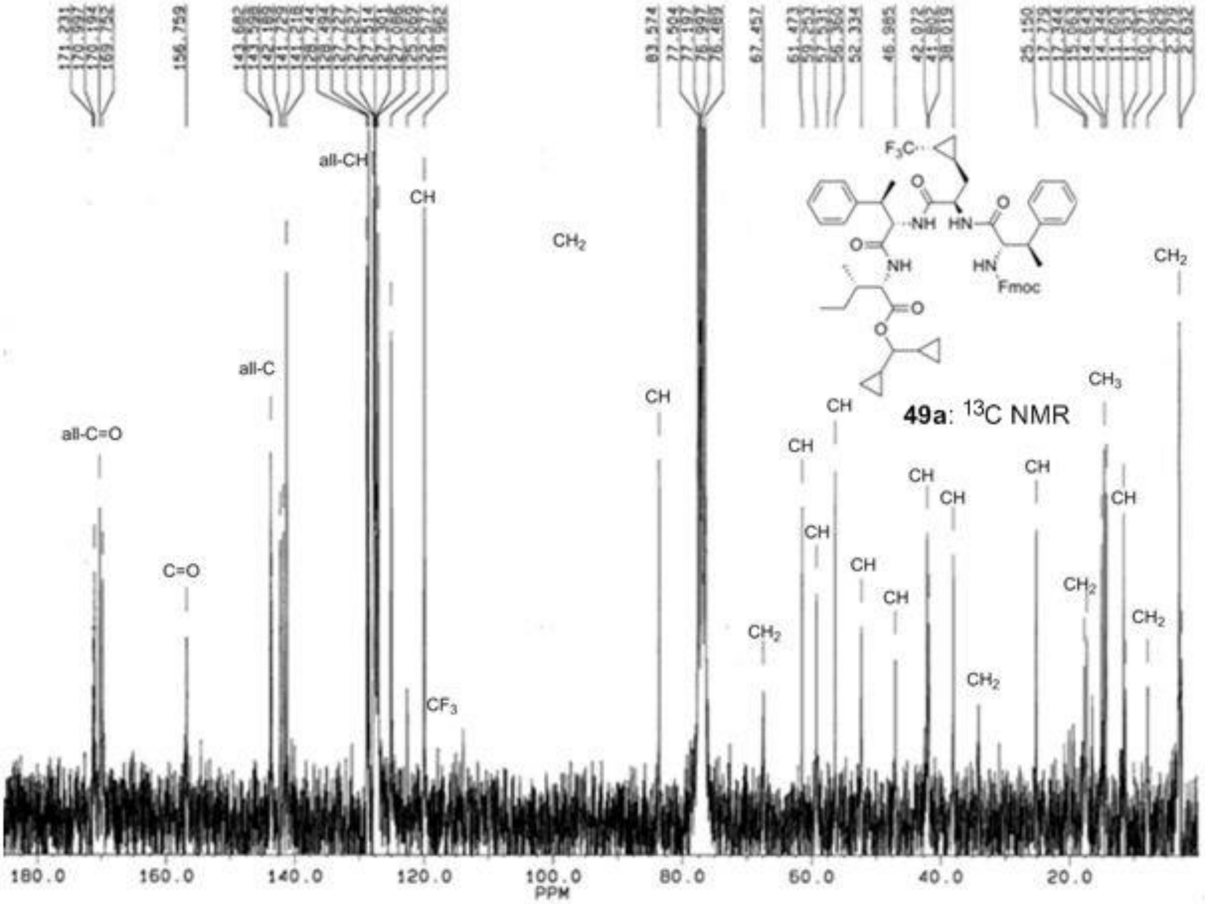

all-C=O
C=O
all-C
all-CH
CH
CF3
CH2
CH
CH
CH
CH
CH
CH
CH
CH
CH
CH2
CH2
CH3
CH2
CH2
CH2
CH
F3C
NH
HN
O
NH
HN
Fmoc
O
O
49a: 13C NMR
180.0
160.0
140.0
120.0
100.0
PPM
80.0
60.0
40.0
20.0

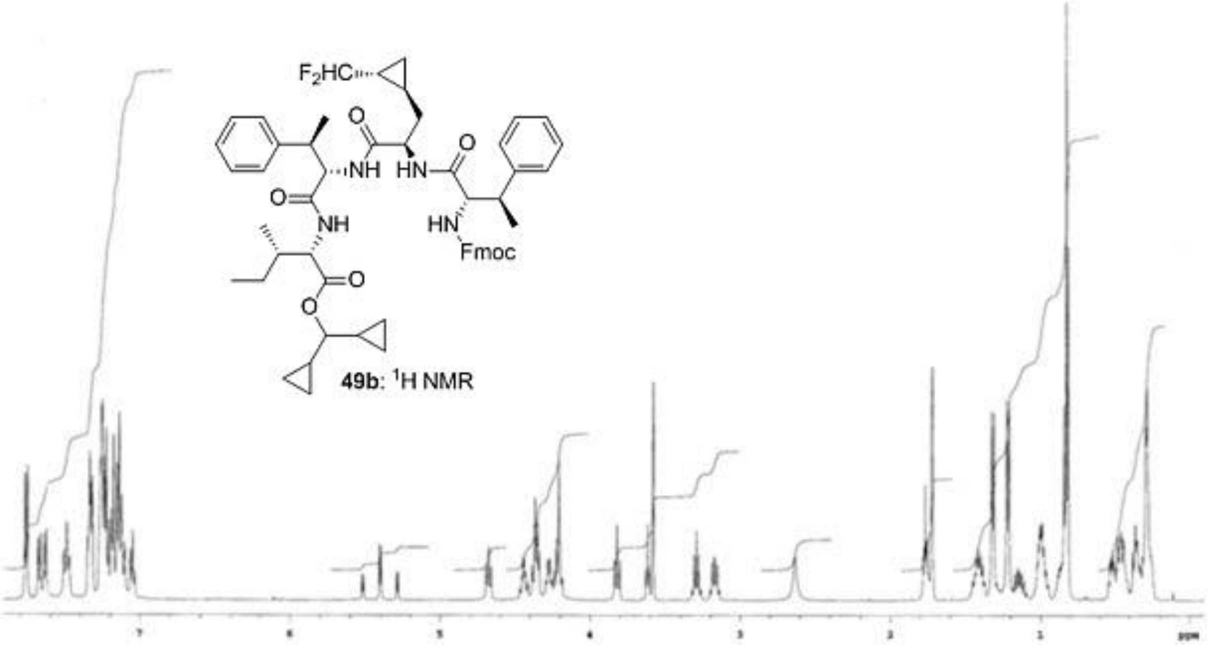

F2HC
O
O
NH
HN
O
NH
HN
Fmoc
O
O
49b: 1H NMR

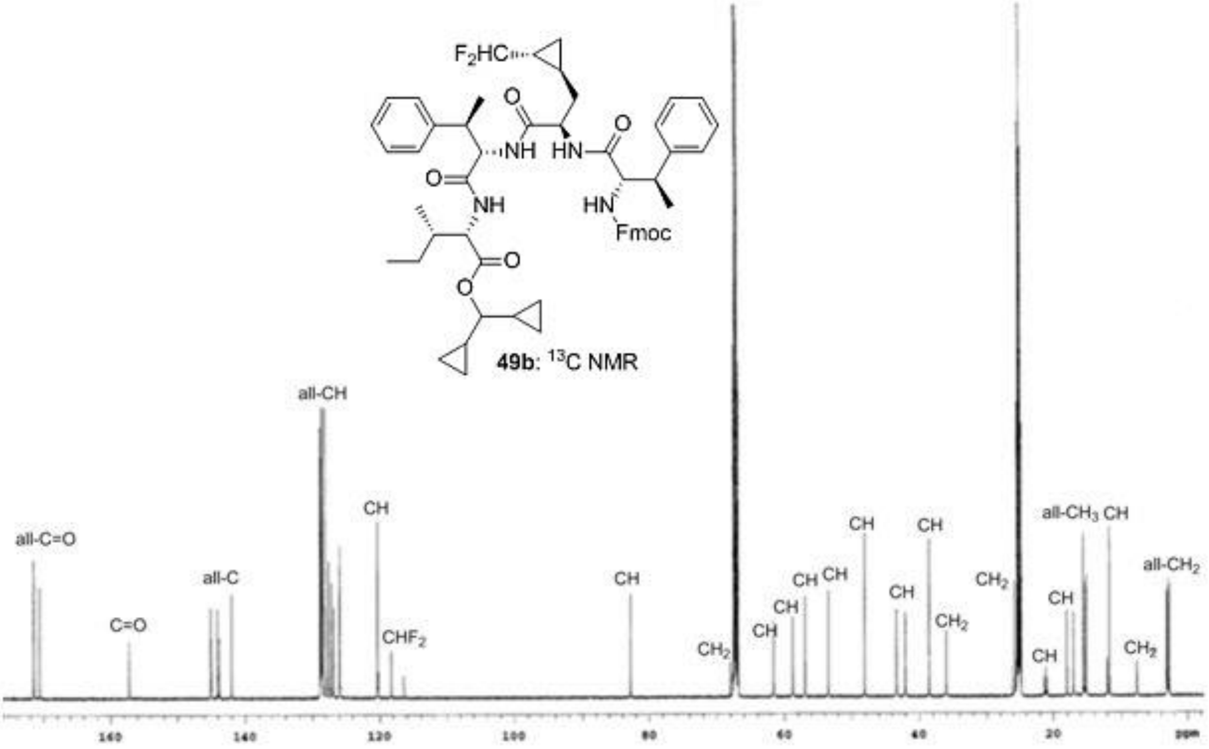

F2HC
NH
HN
O
Fmoc
49b: 13C NMR
all-C=O
C=O
all-C
all-CH
CH
CHF2
CH2
all-CH3
all-CH2
140
120
100
80
60
40
20
ppm

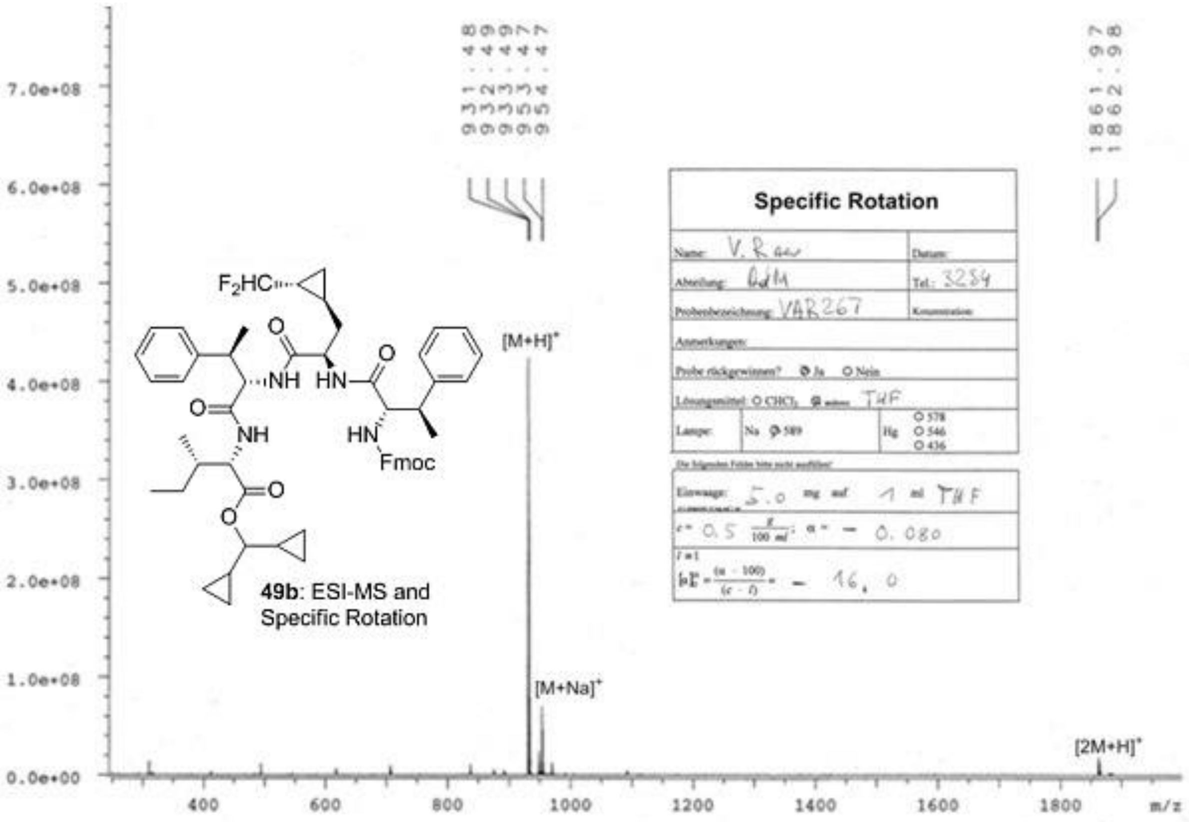

931.48
932.49
933.49
935.47
945.47
1861.97
1862.98
7.0e+08
6.0e+08
5.0e+08
4.0e+08
3.0e+08
2.0e+08
1.0e+08
0.0e+00
[M+H]+
[M+Na]+
[2M+H]+
400
600
800
1000
1200
1400
1600
1800
m/z
F2HC
NH
HN
Fmoc
49b: ESI-MS and Specific Rotation
Specific Rotation
Name: V. Rau
Datum:
Abteilung: AdM
Tel.: 3234
Probenbezeichnung: VAR267
Konzentration:
Anmerkungen:
Probe rückgewinnen? Ja Nein
Lösungsmittel: CHCl3 THF
Lampe: Na 589 Hg 578 546 436
Einwaage: 5.0 mg auf 1 ml THF
c = 0.5 g/100 ml; α = − 0.080
l = 1
[α] = (α · 100)/(c · l) = − 16.0

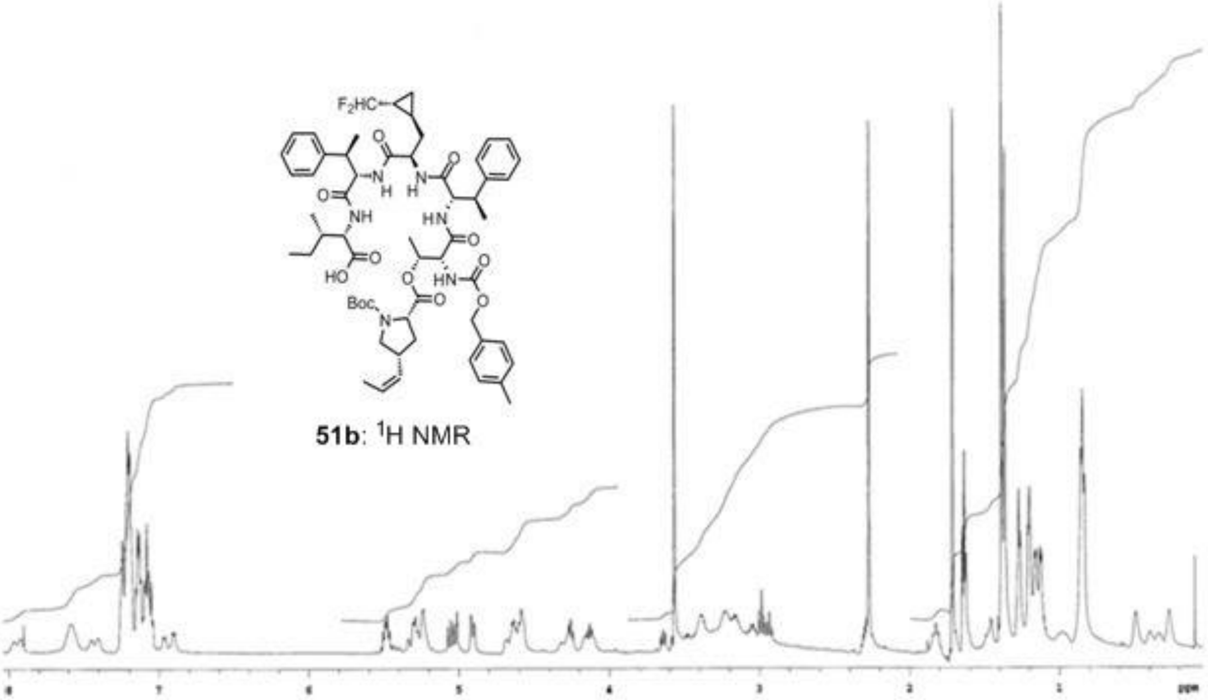

51b: ^{1}H NMR

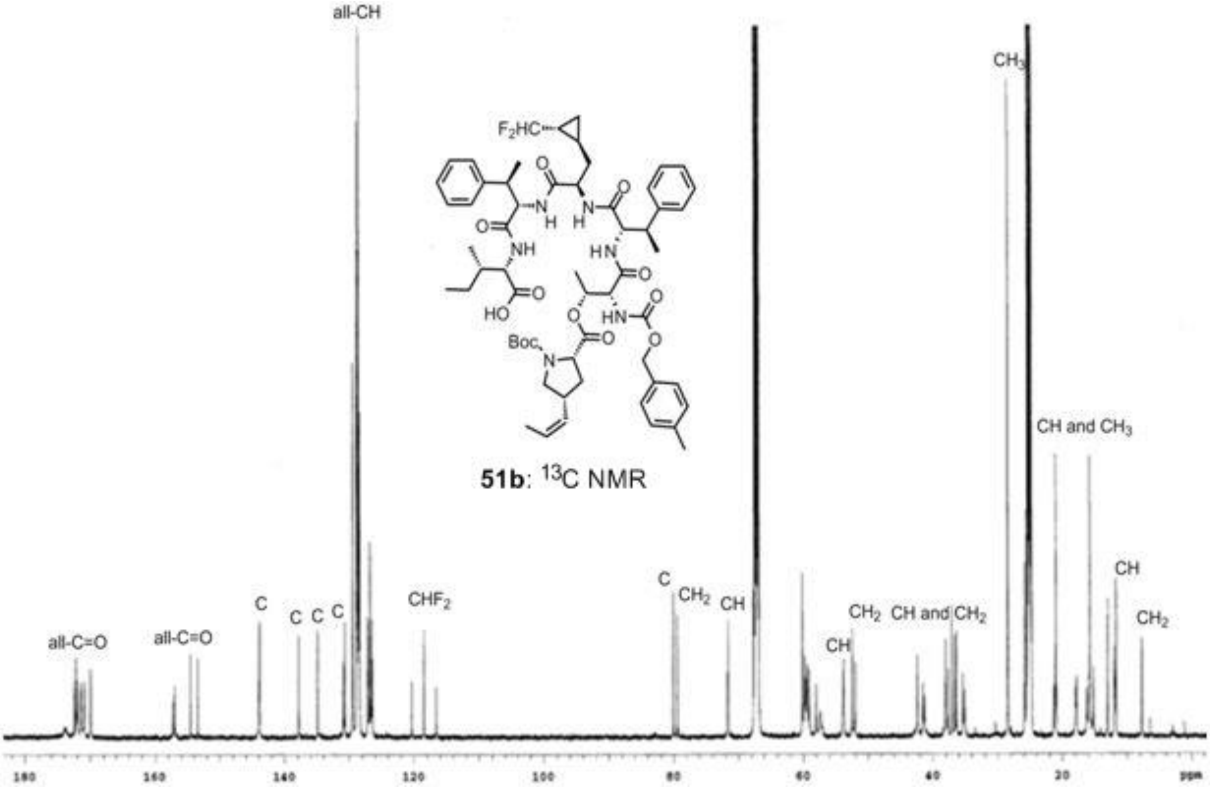

51b: ^{13}C NMR

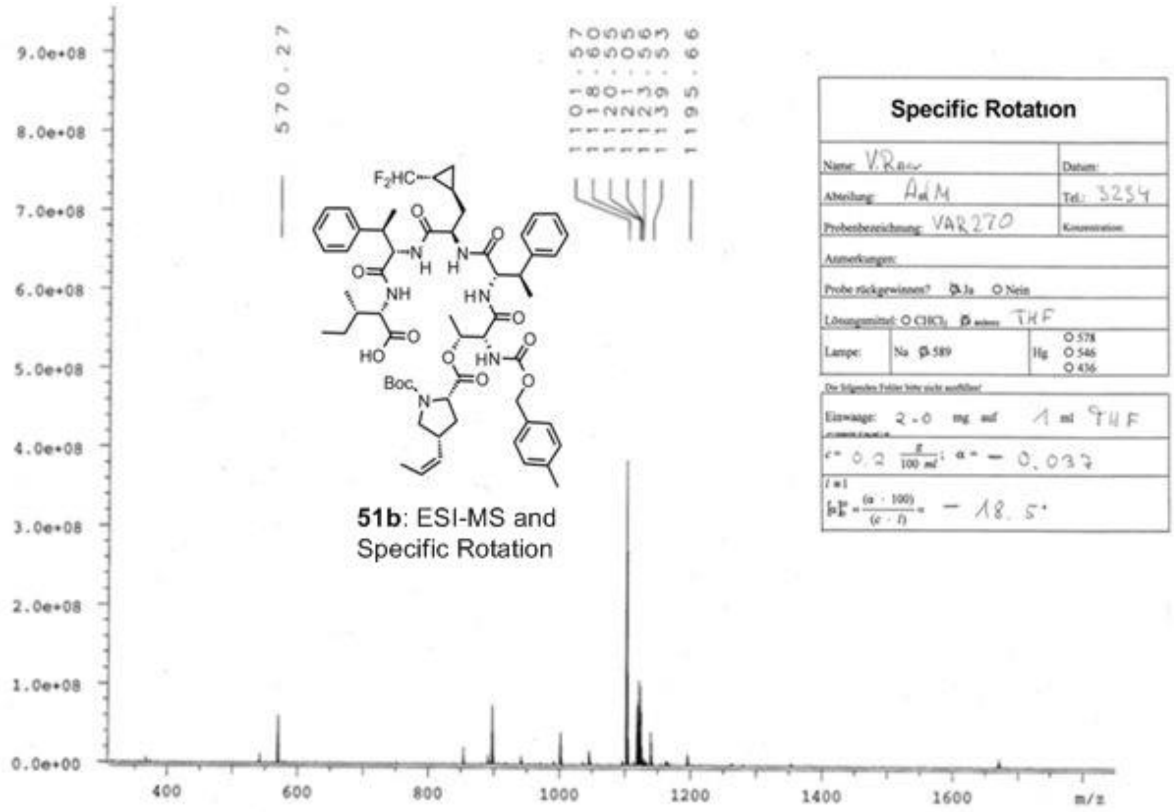

51b: ESI-MS and Specific Rotation

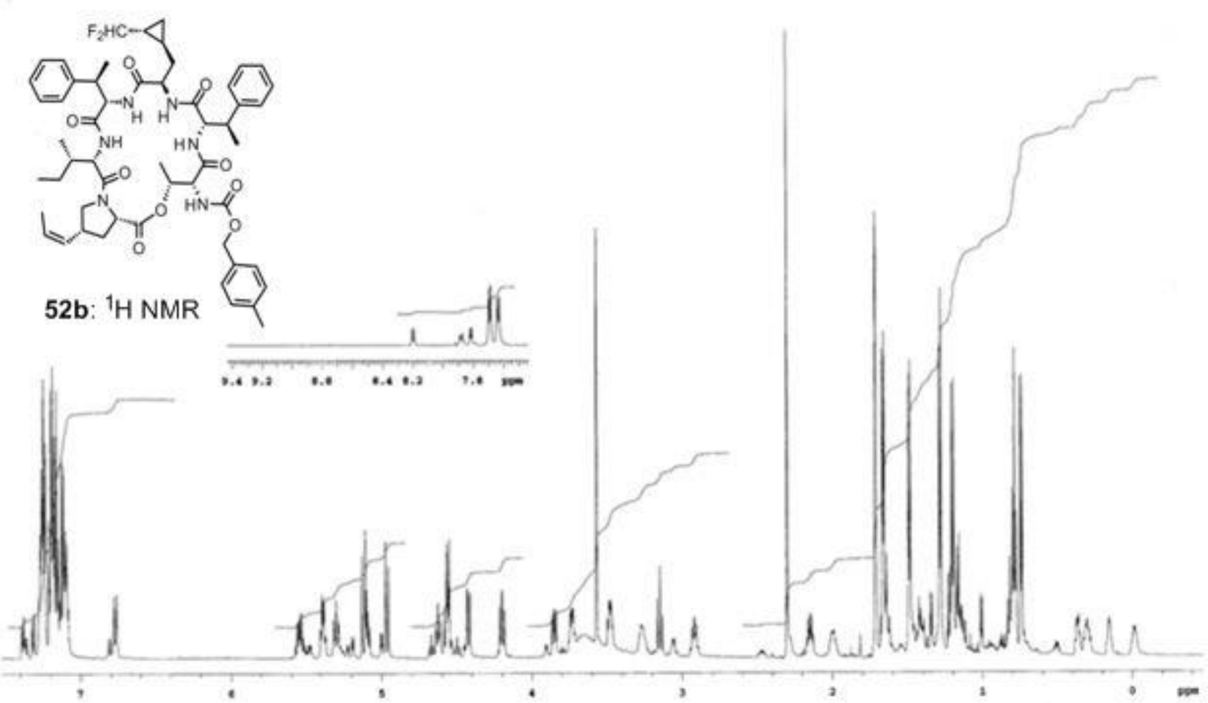

52b: ^{1}H NMR

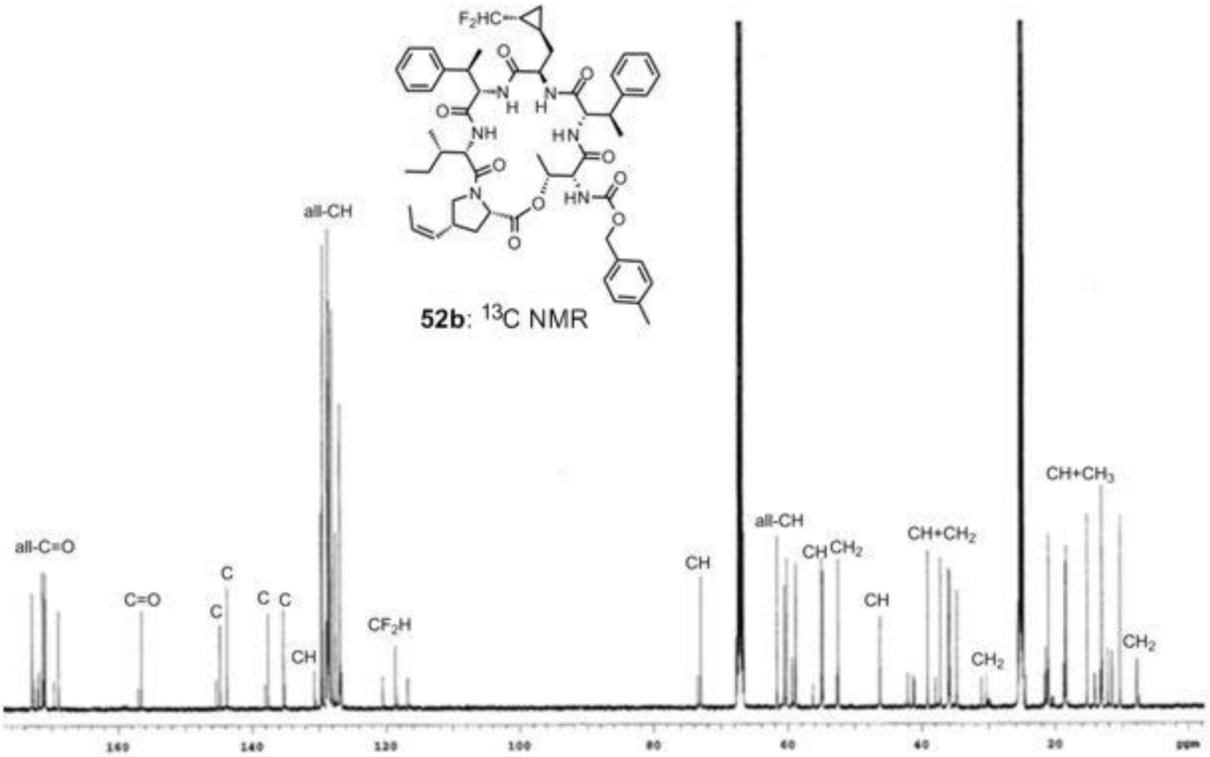
52b: 13C NMR
all-C=O
C=O
C
C
C
C
CH
all-CH
CF2H
CH
all-CH
CH
CH2
CH
CH+CH2
CH2
CH+CH3
CH2
ppm

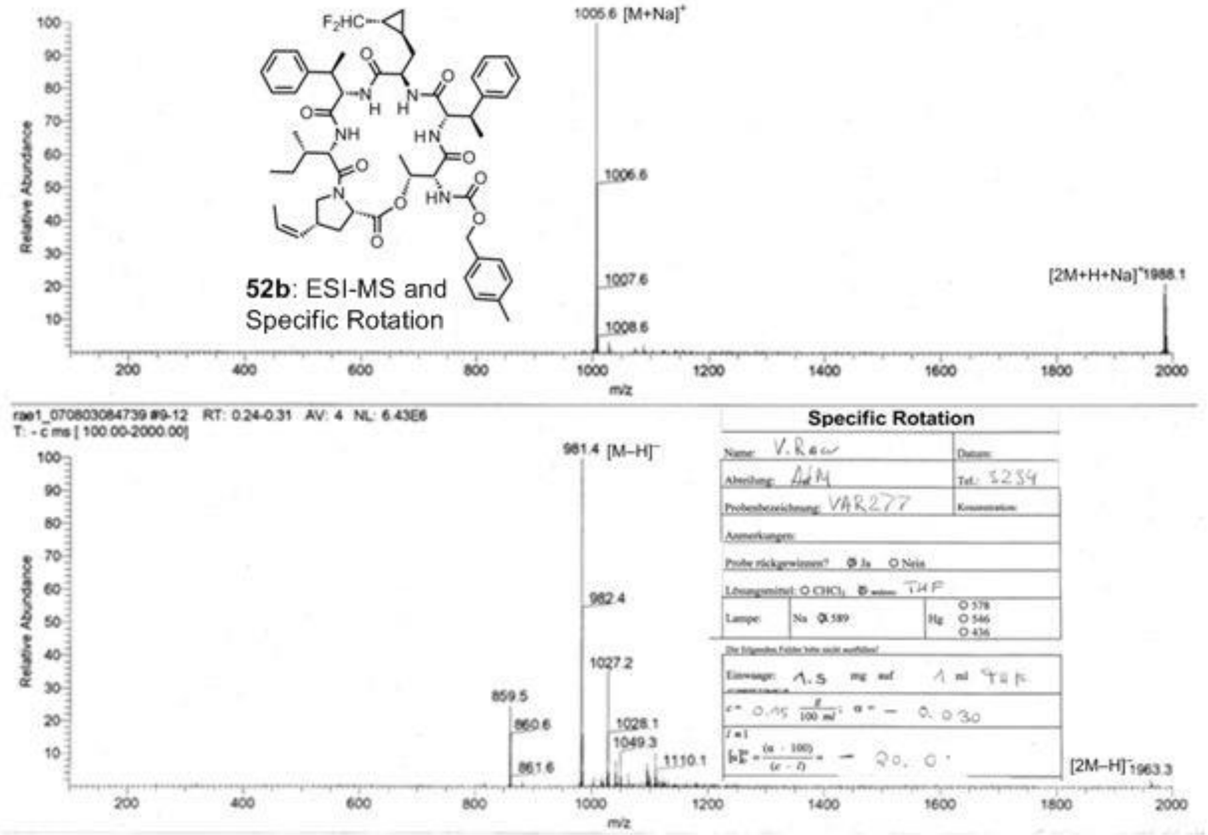
52b: ESI-MS and Specific Rotation
1005.6 [M+Na]+
1006.6
1007.6
1008.6
[2M+H+Na]+1988.1
Relative Abundance
m/z
rae1_070803084739 #9-12 RT: 0.24-0.31 AV: 4 NL: 6.43E6
T: - c ms [100.00-2000.00]
981.4 [M–H]−
982.4
1027.2
859.5
860.6
861.6
1028.1
1049.3
1110.1
[2M–H]−1963.3
Specific Rotation
Name: V. Rew
Abteilung: AdM
Tel.: 5234
Probenbezeichnung: VAR277
Lösungsmittel: THF
Einwaage: 1.5 mg auf 1 ml THF
c = 0.15
α = − 0.030
[α] = − 20.0

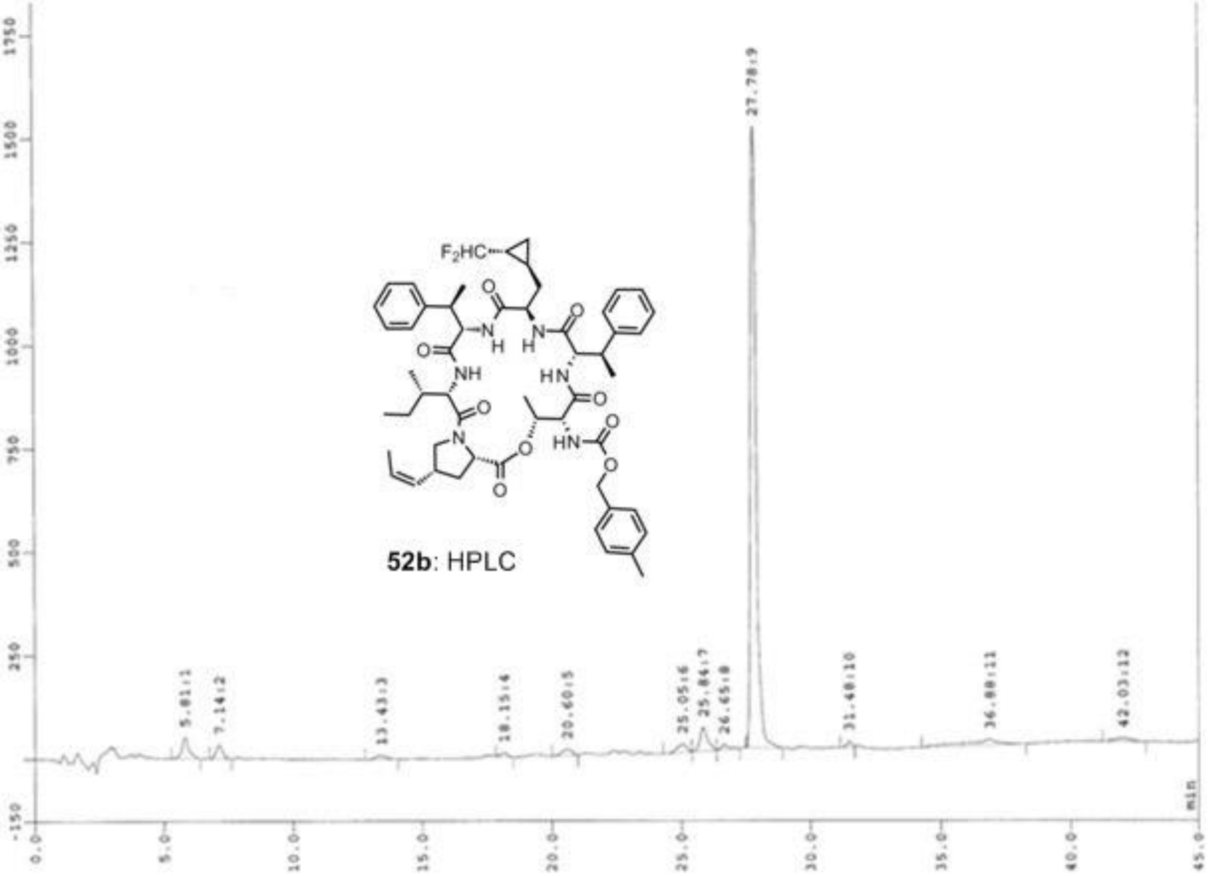
52b: HPLC

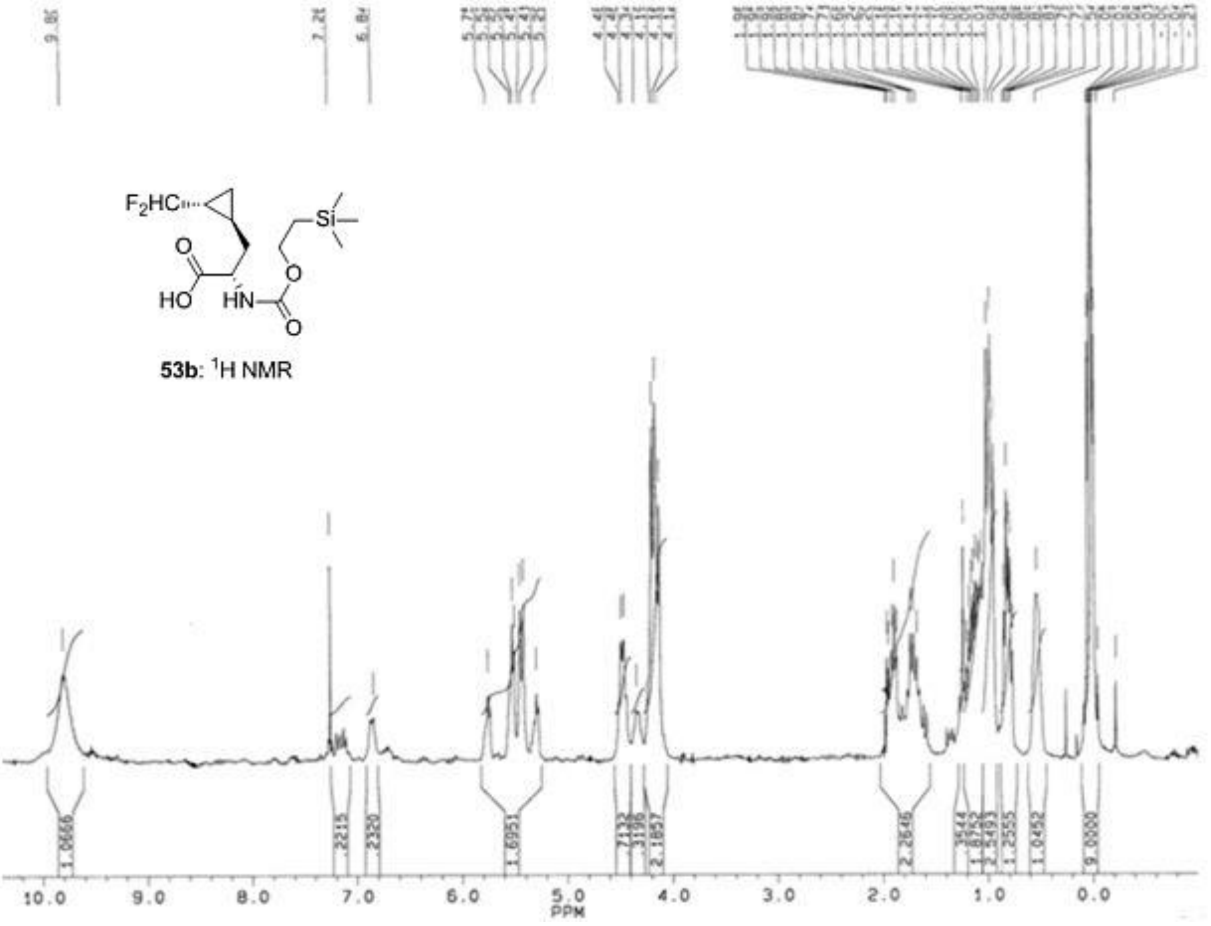
53b: ^{1}H NMR

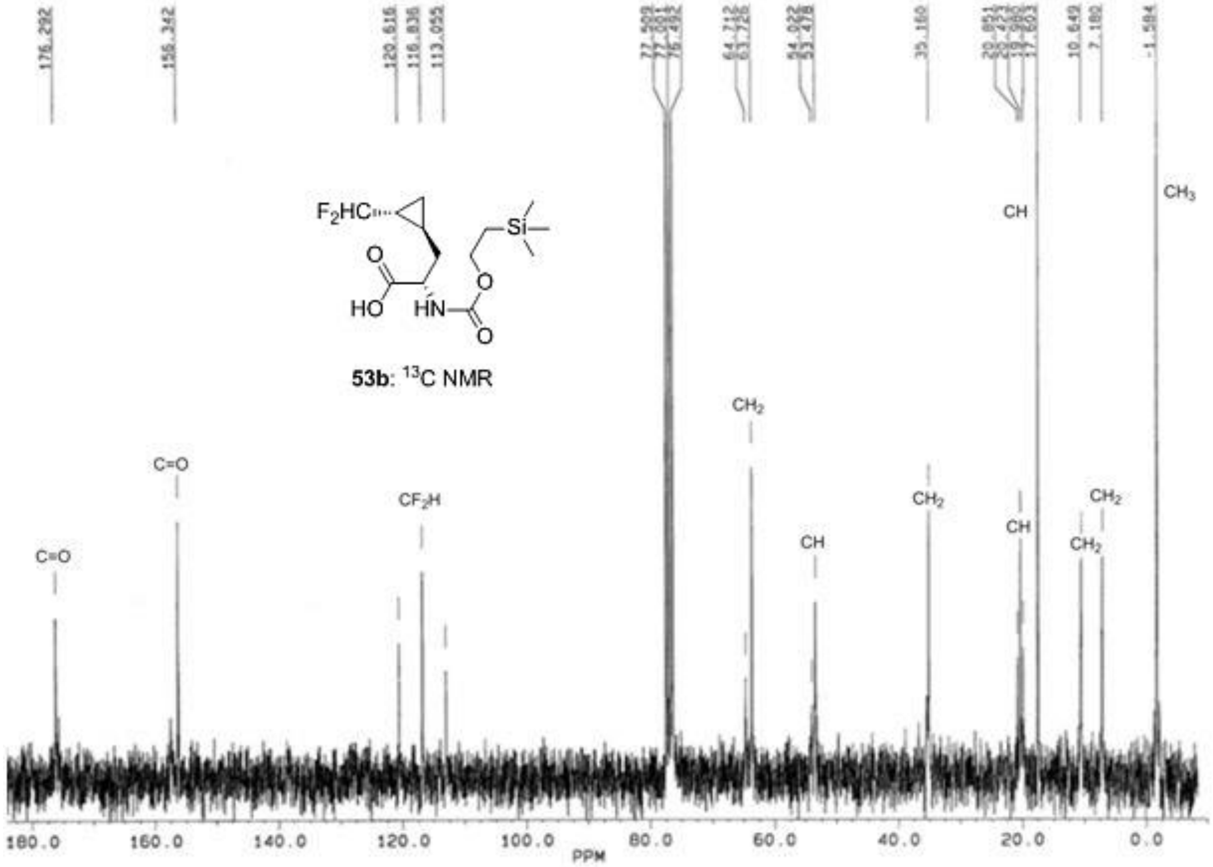
176.292
156.342
120.616
116.836
113.055
64.712
35.160
10.649
7.180
-1.584
53b: ^{13}C NMR
C=O
C=O
CF$_2$H
CH$_2$
CH
CH$_2$
CH
CH
CH$_2$
CH$_2$
CH$_3$
PPM

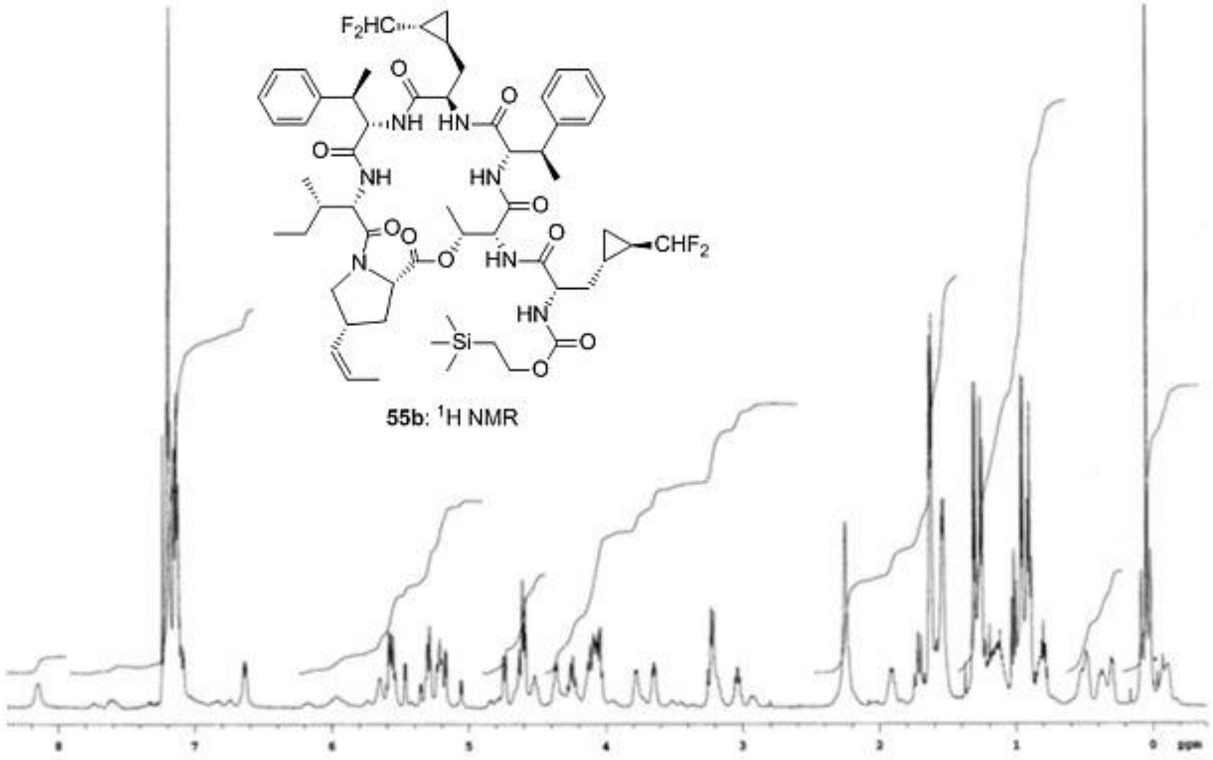
55b: ^{1}H NMR
ppm

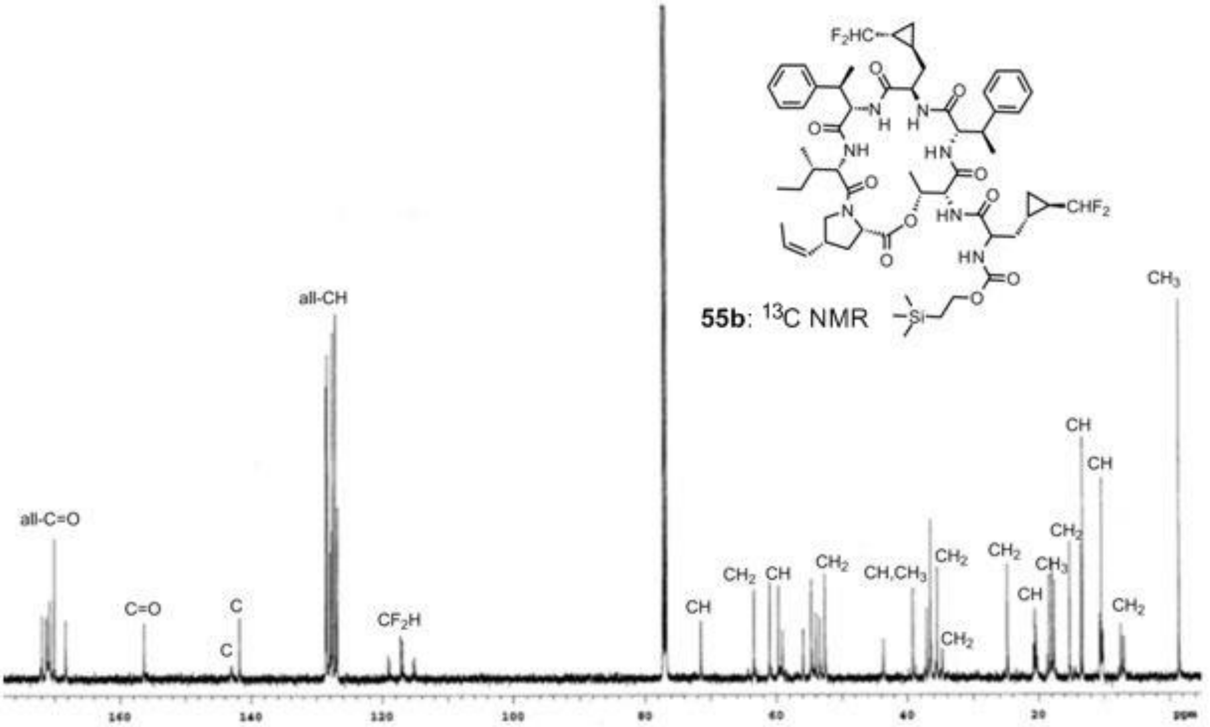

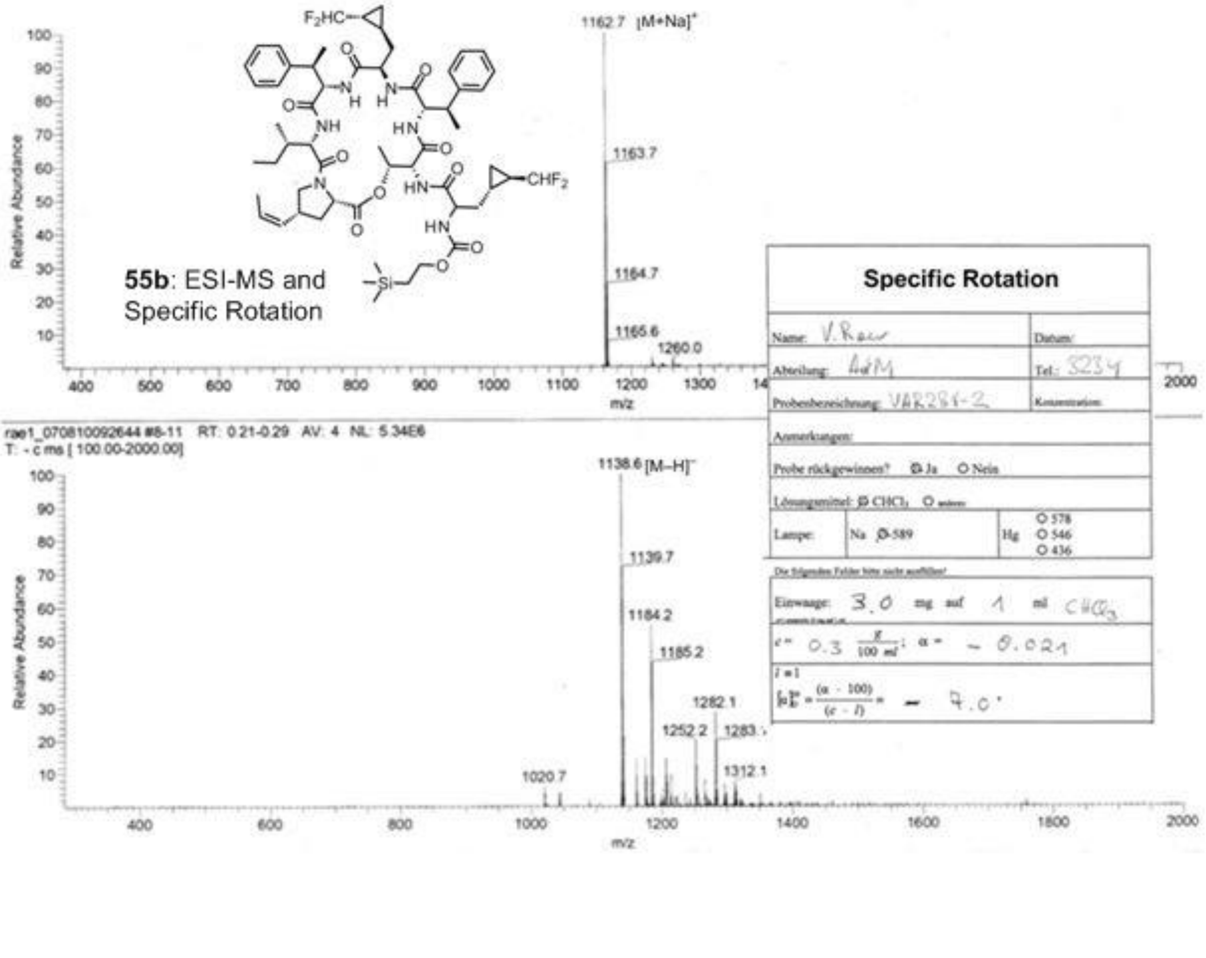

Specific Rotation

Name: V. Rau	Datum:
Abteilung: AdM	Tel.: 3234
Probenbezeichnung: VAR281-2	Konzentration:
Anmerkungen:	
Probe rückgewinnen? ☒ Ja ○ Nein	
Lösungsmittel: ☒ CHCl₃ ○ anderes	
Lampe: Na ☒ 589	Hg ○ 578 ○ 546 ○ 436

Die folgenden Felder bitte nicht ausfüllen!

Einwaage: 3.0 mg auf 1 ml CHCl₃	
c = 0.3 $\frac{g}{100\ ml}$; α = – 0.021	
l = 1	
$[\alpha]_D^{20} = \frac{(\alpha \cdot 100)}{(c \cdot l)}$ = – 7.0°	

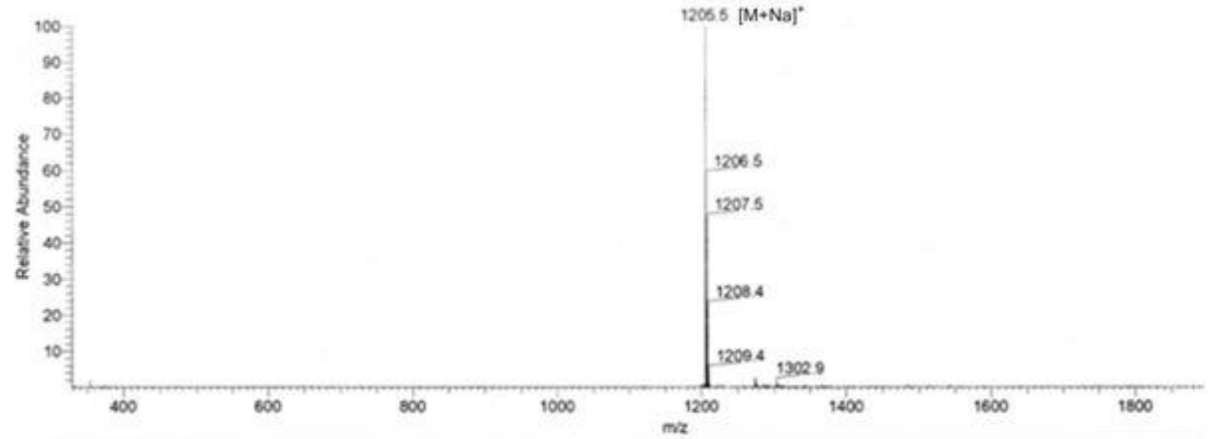

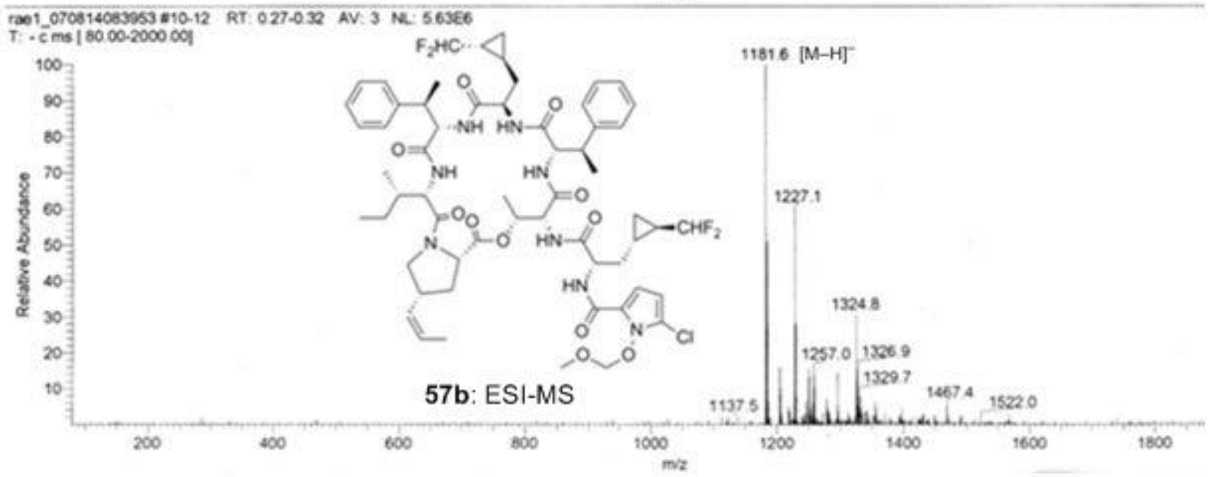

References

1 Hilt, G.; Arndt, M.; Khlebnikov, A. F.; Kozhushkov, S. I.; de Meijere, A. *Eur. J. Org. Chem.* **2012**, 3112–3121.

2 Khlebnikov, A. F.; Kozhushkov, S. I.; Yufit, D. S.; Schill, H.; Reggelin, M.; Spohr, V.; de Meijere, A. *Eur. J. Org. Chem.* **2012**, 1530–1545.

3 Brown, P.; Burdon, J.; Smith, T. J.; Tatlow, J. C. *Tetrahedron* **1960**, *10*, 164–170.

4 Zlatopolskiy, B. D.; Kroll, H.-P.; Melotto, E.; de Meijere, A. *Eur. J. Org. Chem*. **2004**, 4492–4502.

5 Zlatopolskiy, B. D.; de Meijere, A. *Chem. Eur. J*. **2004**, *10*, 4718–4727.

6 Kozhushkov, S. I.; Brandl, M.; Yufit, D. S.; Machinek, R.; de Meijere, A. *Liebigs Ann./Recueil* **1997**, 2197–2204.

7 Bahutski, V. *Dissertation*, Universität Göttingen, **2003**.

8 Baldwin, J. E.; Chang, G. E. C. *Tetrahedron*, **1982**, *36*, *6*, 625–835.

9 Sheldrick, G. M. *Acta Cryst.* **2008**, *A64*, 112–122.
